# New 2-aryl-7,8-dimethoxy-3,4-dihydroisoquinolin-2-ium salts as potential antifungal agents: synthesis, bioactivity and structure-activity relationships

**DOI:** 10.1038/s41598-017-07303-8

**Published:** 2017-08-08

**Authors:** Lifei Zhu, Bohang Zhou, Bingyu Zhang, Mingxuan Xu, Huiling Geng, Le Zhou

**Affiliations:** College of Chemistry & Pharmacy, Northwest A&F University, Yangling, 712100 Shaanxi Province People’s Republic of China

## Abstract

The title compounds can be considered as simple analogues of quaternary benzo[c]phenanthridine alkaloids (QBAs). In order to develop potent QBA-like antifungal agents, as our continuing study, a series of new title compounds were synthesized and evaluated for bioactivity against five plant pathogenic fungi by the mycelium growth rate method in this study. The SAR were also derived. The majority of the compounds showed good to excellent inhibition activity with average EC_50_ values of 7.87–20.0 μM for the fungi, superior to sanguinarine and cherythrine (two QBAs) and the commercial fungicide azoxystrobin. Part of the compounds were more active than commercial fungicides thiabendazole or carbendazim against *F. solani*, *F. graminearum* and *C. gloeosporioides*. Six compounds with average EC_50_ of 3.5–5.1 μg/mL possessed very great potential for development of new antifungal agents. SAR found that substitution patterns of the two aryl-rings significantly affect the activity. There exists a complex interaction effect between substituents of the two aryl-rings on the activity. Generally, the presence of electron-withdrawing groups on the C-ring can significantly increase the activity. These findings will be of great importance for the design of more potent antifungal isoquinoline agents.

## Introduction

Most of plant diseases are caused by plant pathogenic fungi^1^. Plant mycoses not only often result in the loss of crop yield and quality, but also are a food safety problem because some of plant pathogenic fungi can produce mycotoxins harmful to animal and human health^2^. Therefore, various fungicides have been extensively used to manage and treat plant mycosis in current agriculture. However, the persistent and incorrect use of some commercial fungicides had led to some increasingly serious problems, such as hereditable resistance, cross-resistance or environmental pollution^3^. Therefore, it is necessary and urgent to develop novel antifungal agents, especially environmentally friendly plant fungicides. In the past decades, natural product-based antimicrobial agents have attracted attention from researchers due to their lower environmental and mammalian toxicity^4^.

**Figure 1:**
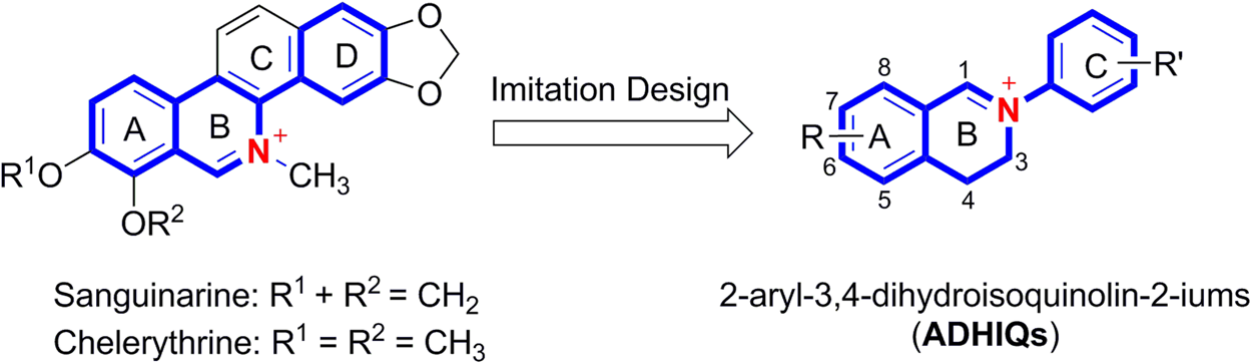
Structures of sanguinarine, chelerythrine and ADHIQs.

2-Aryl-3,4-dihydroisoquinolin-2-ium salts (ADHIQs) (Fig. 1) can be considered as a class of structurally simple analogues of sanguinarine (SA) or chelerythrine (CH) with diverse biological effects, two quaternary benzo[c]phenanthridine alkaloids (QBAs). Our previous study proved that like SA and CH^5–8^, ADHIQs generally possessed excellently antifungal^9–13^, acaricidal^14, 15^ and anticancer activities^5, 16^, and also showed high safety to plant growth^17, 18^. These results show that ADHIQs are of great potential as new or secondary lead compounds to develop QBA-like antifungal agents.

In addition, our study also found that the C=N^+^ moiety in ADHIQs is a determinant for their bioactivities including antifungal properties^5, 9^, and the antifungal activity of ADHIQs is closely related to the electron density distribution in its conjugated system, especially in the C=N^+^ bond^9–13^. The substitution patterns of the two aryl-rings can significantly impact the activity. Meanwhile, there exists a complex effect between substituents of the two aryl-rings on the antifungal activity. These findings encouraged us to further extend the modification of 2-aryl-3,4-dihydroisoquinolin-2-ium compounds with the aim of finding more potent antifungal agents. Considering the structural similarity to chelerythrine containing 7,8-dimethyoxy groups, in the present study, we synthesized a series of new ADHIQs with 7,8-dimethyoxy groups. Herein, we report their synthesis, bioactivity against plant pathogenic fungi and structure-activity relationship.

## Results

### Synthesis

**Figure 2:**
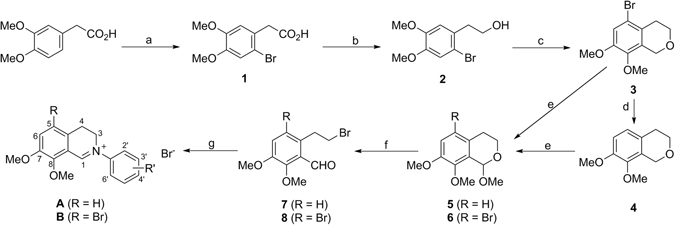
Synthetic route of compounds **A** and **B**. Reagents and conditions: (**a**) dry Br_2_, dry CH_2_Cl_2_; (**b**) NaBH_4_, I_2_, dry THF, 0 to 40 °C; (**c**) (HCHO)_n_, TFA, r.t.; (**d**) n-BuLi, dry THF, −78 °C; (**e**) DDQ, dry MeOH, dry CH_2_Cl_2_; (**f**) TMSBr, Bu_4_NBr, dry toluene, 80 °C; (**g**) Ar-NH_2_, dioxane.

The synthetic route of compounds **A** and **B** is outlined in Fig. 2. Commercially available 2-(3,4-dimethoxyphenyl)acetic acid as a starting material reacted with dry Br_2_ in anhydrous dichloromethane to yield compound **1** in 94% yield. Compound **1** was reduced with NaBH_4_ in the presence of equimolar iodine to obtain crude **2**. Compound **3** was obtained by Oxa-Pictet-Spengler reaction of **2** without purification and paraformaldehyde in trifluoroacetic acid in 75% total yield for two steps. Compound **3** was treated with *n*-butyllithium, and followed by the treatment of water to give **4** in 79% yield.

For key intermediates **7** and **8**, we initially attempted to obtain them by our reported method^9^, i.e., 1-bromination of **3** or **4** with Br_2_ followed by the treatment of aqueous hydrobromic acid. However, the treatment of hydrobromic acid did not give the desired compounds **7** or **8** but unidentified polymers. A similar case was also observed in the preparation of 2-(2-bromoethyl)-4,5-dimethoxy(or 4,5-methylenedioxy)benzaldehyde from the corresponding isochroman^13, 19^. Subsequently, **3** or **4** was oxidized with 2,3-dichloro-5,6-dicyano-p-benzoquinone (DDQ) in CH_2_Cl_2_ containing MeOH to provide acetals **5** or **6**, respectively. Compound **5** or **6** was treated with Bu_4_NBr and TMSBr in dry toluene to yield key intermediates **7** or **8**. Finally, **7** or **8** reacted with various primary aromatic amines to yield two series of the target compounds **A** and **B**. It was worth mentioning that for some primary aromatic amines with a strong electron-withdrawing group such as nitro, trifluoromethyl or cyano, we did not obtain the desired target compounds with enough purity by the reaction above.

The structures of all synthesized compounds were confirmed by NMR and MS. All target compounds **A** and **B** presented some similar spectral features due to the structural similarity. In NMR spectra, each of the compounds **A** or **B** revealed one singlet signal in the range of *δ*_H_ 9.17 to 9.63 ppm and *δ*_C_ 159.9 to 170.7 ppm due to the iminium moiety (C=N^+^), double triplet signals of a A_2_X_2_ system at *δ*_H_ ca. 3.4 (2H, t, *J* = ca. 7.8 Hz) and ca. 4.5 (2H, t, *J* = ca. 7.8 Hz) and two carbon signals at *δ*_C_ ca. 26.0 and ca. 63.0 due to the CH_2_CH_2_ unity, and signals of two methoxy groups at *δ*_H_ ca. 3.9 (3H, s) and ca. 4.1 (3H, s) and *δ*_C_ ca. 53.0 and ca. 57.5. In ESI-MS or HR-ESI-MS spectra, **A** and **B** showed one corresponding characteristic ion peak at m/z [M−Br]^+^. Due to the same synthetic method and structural similarity, only part of the compounds were performed for HR-MS and analysized for bromine ion. In the negative ESI-MS spectrum of **A1**, the ion peaks at m/z [^79^Br]^−^ and [^81^Br]^−^ were observed, showing the presence of bromine ion.

### Antifungal Activity

Based on the mycelia growth rate method reported previously by us^9^, all compounds **A** and **B** were initially screened for antifungal activity *in vitro* at 150 μM (52–76 μg/mL) against five plant pathogenic fungi (*Fusarium solani*, *Fusarium graminearum*, *Valsa mali*, *Curvularia lunata* and *Colletotrichum gloeosporioides*). Sanguinarine (SA) and chelerythrine (CH), two natural model compounds, were used as reference controls. Thiabendazole (TBZ), carbendazim (CBD) and azoxystrobin (ASB), three commercial fungicide standards, were used as positive controls. The results are listed in Table 1.

**Table 1:** Antifungal activity of compounds **A** and **B** at 150 μM.

Compounds			Average inibition rate ± SD (%)^a^					Mean (%)^b^
No.	R	R′	*F. solani*	*F. graminearum*	*V. mali*	*C. lunata*	*C. gloeosporioides*	
**A1**	H	H	77.9 ± 1.7 qr	75.0 ± 2.7 hi	60.7 ± 0.7 t	71.7 ± 0.9 l	88.5 ± 1.4 fg	**74.8**
**A2**	H	2′-F	83.7 ± 2.0 kl	99.6 ± 0.7 ab	84.6 ± 3.7 ij	83.7 ± 1.5 hi	100.0 ± 0.0 a	**90.3**
**A3**	H	3′-F	90.7 ± 0.0 defg	100.0 ± 0.0 a	82.7 ± 0.8 jklm	85.7 ± 0.9 fghi	100.0 ± 0.0 a	**91.8**
**A4**	H	4′-F	83.5 ± 1.7 kl	85.2 ± 1.4 f	72.7 ± 1.2 r	71.7 ± 2.5 l	94.3 ± 1.4 cd	**81.5**
**A5**	H	2′-Cl	93.4 ± 0.7 bcd	100.0 ± 0.0 a	86.9 ± 0.8 gh	89.7 ± 1.5 abcd	100.0 ± 0.0 a	**94.0**
**A6**	H	3′-Cl	82.1 ± 0.7 lm	100.0 ± 0.0 a	84.6 ± 0.0 ij	87.2 ± 0.9 defg	97.5 ± 2.9 ab	**90.3**
**A7**	H	4′-Cl	94.2 ± 0.0 bc	99.2 ± 0.7 ab	86.9 ± 2.1 gh	86.7 ± 0.0 efg	100.0 ± 0.0 a	**93.4**
**A8**	H	2′-Br	95.3 ± 0.0 ab	100.0 ± 0.0 a	87.9 ± 2.1 efgh	91.1 ± 0.0 ab	100.0 ± 0.0 a	**94.9**
**A9**	H	3′-Br	92.2 ± 0.7 cde	99.6 ± 0.7 ab	83.2 ± 0.0 jkl	87.2 ± 1.7 defg	98.7 ± 1.1 a	**92.2**
**A10**	H	4′-Br	91.8 ± 2.0 cdef	91.7 ± 4.0 de	87.4 ± 1.4 fgh	91.6 ± 0.9 a	98.1 ± 1.9 ab	**92.1**
**A11**	H	2′-I	94.0 ± 1.3 bc	96.5 ± 1.2 bc	94.2 ± 1.4 b	90.2 ± 0.0 abc	95.9 ± 1.4 bc	**94.2**
**A12**	H	3′-I	87.4 ± 1.4 hij	98.2 ± 0.8 ab	84.6 ± 0.8 ij	88.7 ± 1.8 bcde	66.2 ± 2.9 mn	**85.0**
**A13**	H	4′-I	85.4 ± 1.1 jk	71.9 ± 0.0 k	88.0 ± 1.9 efgh	87.5 ± 0.9 cdefg	90.2 ± 0.0 ef	**84.6**
**A14**	H	2′,6′-diF	78.7 ± 2.1 opqr	100.0 ± 0.0 a	79.6 ± 1.4 nop	87.7 ± 0.0 cdefg	100.0 ± 0.0 a	**89.2**
**A15**	H	2′,4′-diCl	88.6 ± 0.7 ghi	99.6 ± 0.8 ab	89.6 ± 0.8 def	88.7 ± 0.9 bcde	88.5 ± 0.0 fg	**91.0**
**A16**	H	3′-NO_2_	89.4 ± 1.2 efgh	99.6 ± 0.8 ab	90.0 ± 2.1 de	85.6 ± 0.9 fghi	100.0 ± 0.0 a	**92.9**
**A17**	H	2′-CF_3_	79.8 ± 1.1 mnopq	78.9 ± 2.0 g	80.6 ± 0.7 mno	91.3 ± 0.9 ab	90.2 ± 0.0 ef	**84.1**
**A18**	H	3′-CF_3_	81.3 ± 1.3 lmnop	85.2 ± 0.7 f	74.8 ± 1.9 q	85.3 ± 1.6 fghi	93.4 ± 1.4 cd	**84.0**
**A19**	H	3′-CN	87.8 ± 0.7 ghij	99.6 ± 0.8 ab	77.7 ± 0.8 p	85.1 ± 0.9 ghi	100.0 ± 0.0 a	**90.0**
**A20**	H	2′-CH_3_	76.8 ± 1.4 qr	60.9 ± 1.6 m	22.3 ± 1.4 x	65.1 ± 1.8 m	51.0 ± 1.1 o	55.2
**A21**	H	3′-CH_3_	85.0 ± 2.5 jk	85.5 ± 2.1 f	57.3 ± 1.6 u	72.8 ± 1.8 kl	92.4 ± 0.0 de	**78.6**
**A22**	H	4′-CH_3_	76.8 ± 3.8 qr	83.2 ± 0.8 f	57.7 ± 1.4 u	78.5 ± 0.0 j	65.6 ± 1.9 n	**72.3**
**A23**	H	2′-OMe	70.4 ± 1.8 s	52.6 ± 3.4 o	27.6 ± 0.0 w	50.0 ± 1.5 o	38.6 ± 0.0 pq	47.8
**A24**	H	3′-OMe	70.8 ± 1.1 s	72.7 ± 2.0 ij	51.8 ± 1.5 v	74.5 ± 0.9 k	73.5 ± 2.1 ij	68.7
**A25**	H	4′-OMe	54.5 ± 3.1 u	56.0 ± 2.0 n	28.1 ± 0.8 w	57.8 ± 3.1 n	39.2 ± 2.8 p	47.1
**A26**	H	2′-OH	38.9 ± 1.4 v	65.4 ± 1.3 l	10.1 ± 0.8 z	34.8 ± 1.7 p	21.1 ± 2.1 s	34.1
**A27**	H	3′-OH	31.1 ± 1.2 w	47.4 ± 0.0 p	14.0 ± 0.8 y	27.9 ± 0.0 r	25.3 ± 2.1 r	29.2
**A28**	H	4′-OH	17.5 ± 2.4 x	11.1 ± 2.0 q	7.5 ± 0.8 a	14.2 ± 2.3 s	4.8 ± 1.0 t	11.0
**B1**	Br	H	81.1 ± 1.3 lmnop	90.9 ± 1.5 de	83.3 ± 0.0 jk	91.8 ± 0.0 a	99.3 ± 1.2 a	**81.7**
**B2**	Br	2′-F	86.1 ± 1.3 ijk	93.4 ± 0.9 cd	88.5 ± 0.7 efg	91.8 ± 1.6 a	99.3 ± 1.2 a	**88.8**
**B3**	Br	3′-F	81.1 ± 1.3 lmnop	89.3 ± 1.5 e	81.8 ± 1.4 klmn	89.6 ± 1.0 abcd	86.4 ± 2.4 g	**80.1**
**B4**	Br	4′-F	86.1 ± 1.3 ijk	93.9 ± 0.0 cd	85.7 ± 0.0 hi	86.9 ± 0.0 defg	100.0 ± 0.0 a	**84.4**
**B5**	Br	3′-Cl	81.9 ± 0.7 lmn	70.1 ± 2.3 jk	81.4 ± 0.7 klmn	87.4 ± 1.0 cdefg	69.4 ± 2.0 kl	**73.4**
**B6**	Br	4′-Cl	76.1 ± 1.3 r	61.4 ± 0.9 m	81.0 ± 0.0 lmn	86.3 ± 1.0 efgh	75.5 ± 0.0 i	**70.1**
**B7**	Br	3′-Br	78.6 ± 0.0 pqr	61.4 ± 0.9 m	81.4 ± 0. 7 klmn	85.3 ± 1.6 fghi	64.0 ± 1.2 n	**69.8**
**B8**	Br	4′-Br	79.0 ± 3.2 nopqr	63.5 ± 3.0 lm	78.6 ± 1.2 op	83.1 ± 1.0 i	78.9 ± 1.2 h	**71.4**
**B9**	Br	4′-I	76.8 ± 0.7 qr	69.1 ± 2.4 k	68.6 ± 1.4 s	79.9 ± 3.4 j	71.3 ± 1.4 jk	**69.7**
**B10**	Br	2′-OMe	81.7 ± 1.7 lmno	98.8 ± 0.0 ab	78.5 ± 1.4 op	88.0 ± 1.9 cdef	88.5 ± 2.8 fg	**81.2**
**Sanguinarine (SA)**			89.0 ± 0.1 fghi	76.3 ± 2.4 gh	91.5 ± 0.1 cd	90.2 ± 0.7 abc	100.0 ± 0.0 a	89.4
**Chelerythrine (CH)**			90.3 ± 2.0 efgh	74.7 ± 1.7 hi	93.0 ± 0.1 bc	87.2 ± 0.0 defg	52.8 ± 2.4 o	79.6
**Thiabendazole (TBZ)**			85.2 ± 3.2 jk	95.0 ± 3.5 c	98.3 ± 1.4 a	16.2 ± 0.8 s	100.0 ± 0.0 lm	78.9
**Carbendazim (CBD)**			97.2 ± 0.0 a	100.0 ± 0.0 a	94.6 ± 0.0 b	30.8 ± 3.2 q	100.0 ± 0.0 a	84.5
**Azoxystrobin (ASB)**			64.0 ± 0.2 t	63.4 ± 3.1 lm	52.4 ± 0.4 v	48.4 ± 1.7 o	36.4 ± 1.4 q	52.9

^a^The difference between the data with the different letters within a column is significant for the same fungi (*P* < 0.05). ^b^Mean: the average values of inhibition rates of the same compound on the five test fungi.

Gratifyingly, almost all the compounds presented a certain inhibition activity against each fungus. For average inhibition rate of the same compound on all the five test fungi, among all the 38 tested compounds, 33 compounds gave average inhibition rates of >55%, superior to ASB (52%); 22 compounds showed average inhibition rates of >80%, higher than both TBZ (78.9%) and CH (79.6%); 12 compounds displayed average inhibition rates of 90.0% to 94.9%, more active than CBD (84.5%) and SA (89.4%). It was worth noting that except for **A26**–**A28**, all compounds were much more active than three commercial fungicides against *C. lunata*. On the other hand, for each fungus, the vast majority of the compounds showed the higher activity than ASB. Based on the average inhibition rate of all compounds **A** or **B** on the same fungus, it was found that the order of susceptibility of five test fungi to compounds **A** was *F. graminearum* (82.7%) > *C. gloeosporioide*s (79.0%) ≈ *F. solani* (77.8%) > *C. lunata* (75.4%) > *V. mali* (66.2%) while compounds **B** was *C. lunata* (87.0%) > *C. gloeosporioides* (83.3%) > *F. solani* (80.9%) = *V. mali* (80.9%) > *F. graminearum* (79.2%).

In order to more clearly know the difference among the activity of the more active compounds in Table 1, we further determined their activity at lower concentrations (75.0 μM) (26–38 μg/mL) against the same fungi. The results are listed in Table 2. Excitingly, 27 out of the 33 tested compounds showed the higher activity with average inhibition rates of 62.5% to 84.1% for the five test fungi.

**Table 2:** Antifungal activity of compounds **A** and **B** at 75 μM.

Compounds			Average inibition rate ± SD (%)^a^					Mean (%)^b^
No.	R	R′	*F. solani*	*F. graminearum*	*V. mali*	*C. lunata*	*C. gloeosporioides*	
**A1**	H	H	58.1 ± 2.0 r	43.6 ± 0.0 s	26.6 ± 1.2 q	48.0 ± 1.0 j	25.7 ± 1.0 t	40.4
**A2**	H	2′-F	79.8 ± 1.8 def	69.4 ± 1.9 hij	71.0 ± 1.4 ef	73.7 ± 1.8 ef	97.7 ± 2.0 a	**78.3**
**A3**	H	3′-F	76.7 ± 1.2 hij	66.9 ± 0.6 jklm	71.8 ± 1.4 cde	69.6 ± 2.0 g	68.4 ± 3.5 jk	**70.7**
**A4**	H	4′-F	78.7 ± 0.7 efgh	67.8 ± 2.0 jkl	47.7 ± 1.2 n	60.8 ± 1.0 h	40.4 ± 3.5 r	**59.1**
**A5**	H	2′-Cl	85.7 ± 2.4 b	65.3 ± 3.4 lm	73.9 ± 2.5 bcd	76.6 ± 1.0 cde	93.0 ± 3.5 bc	**78.9**
**A6**	H	3′-Cl	79.8 ± 0.7 def	80.3 ± 2.4 e	72.6 ± 0.0 cde	77.2 ± 1.8 cd	70.2 ± 3.5 ij	**76.0**
**A7**	H	4′-Cl	81.4 ± 0.0 cd	82.8 ± 1.9 d	73.0 ± 1.9 cde	74.3 ± 2.7 def	93.0 ± 0.0 bc	**80.9**
**A8**	H	2′-Br	86.1 ± 2.3 b	74.5 ± 1.1 g	73.4 ± 0.7 cde	74.3 ± 2.0 def	89.5 ± 0.0 cd	**79.6**
**A9**	H	3′-Br	80.2 ± 0.7 de	70.8 ± 0.5 hi	71.0 ± 2.1 ef	82.3 ± 0.9 b	71.4 ± 2.3 hij	**75.2**
**A10**	H	4′-Br	78.3 ± 0.7 efgh	68.1 ± 2.3 ijk	71.5 ± 0.8 de	79.3 ± 0.9 bc	85.7 ± 2.3 e	**76.6**
**A11**	H	2′-I	91.3 ± 1.4 a	71.4 ± 1.4 h	81.8 ± 1.4 a	87.4 ± 0.9 a	76.2 ± 0.0 fg	**81.6**
**A12**	H	3′-I	77.1 ± 1.4 ghij	66.0 ± 1.9 klm	68.7 ± 1.6 fg	82.3 ± 0.9 b	59.1 ± 2.0 no	**70.6**
**A13**	H	4′-I	81.4 ± 1.8 cd	70.8 ± 1.4 hi	74.3 ± 0.8 bc	81.8 ± 0.0 b	89.6 ± 2.3 cd	**79.6**
**A14**	H	2′,6′-diF	71.2 ± 1.4 lm	77.4 ± 0.0 f	68.7 ± 2.1 fg	77.3 ± 1.5 cd	96.1 ± 0.0 ab	**78.1**
**A15**	H	2′,4′-diCl	81.4 ± 0.7 cd	84.9 ± 1.0 cd	83.2 ± 0.0 a	85.4 ± 0.9 a	87.0 ± 2.3 de	**84.1**
**A16**	H	3′-NO_2_	77.9 ± 1.4 efghi	78.0 ± 1.4 ef	72.0 ± 2.8 cde	80.8 ± 0.9 b	92.9 ± 3.0 bc	**80.3**
**A17**	H	2′-CF_3_	78.8 ± 0.7 efgh	64.4 ± 1.9 mn	65.9 ± 1.6 hi	69.8 ± 2.8 g	65.8 ± 3.3 kl	**68.9**
**A18**	H	3′-CF_3_	77.6 ± 0.7 fghi	85.6 ± 1.0 bc	66.4 ± 0.0 gh	77.2 ± 1.1 cd	63.3 ± 2.2 lm	**74.0**
**A19**	H	3′-CN	77.6 ± 1.9 fghi	92.8 ± 1.0 a	54.7 ± 0.8 m	74.1 ± 1.9 ef	93.7 ± 2.2 b	**78.6**
**A20**	H	2′-CH_3_	60.4 ± 0.0 qr	26.8 ± 2.6 t	9.0 ± 1.6 r	30.7 ± 3.0 k	24.1 ± 3.3 t	30.2
**A21**	H	3′-CH_3_	61.2 ± 0.7 qr	52.2 ± 1.9 q	24.3 ± 0.0 q	46.3 ± 3.2 j	29.8 ± 0.0 s	42.8
**A22**	H	4′-CH_3_	68.6 ± 1.4 no	52.2 ± 1.0 q	31.3 ± 1.4 p	53.7 ± 1.9 i	32.3 ± 1.1 s	47.6
**A24**	H	3′-OMe	49.5 ± 3.0 s	47.0 ± 2.3 r	36.0 ± 0.8 o	53.9 ± 1.8 i	48.8 ± 2.1 q	47.0
**B1**	Br	H	74.7 ± 0.7 jk	87.8 ± 1.9 b	65.4 ± 1.6 hij	76.5 ± 1.1 cde	74.1 ± 1.1 gh	**75.7**
**B2**	Br	2′-F	79.6 ± 1.4 defg	86.7 ± 0.0 bc	76.2 ± 1.4 b	79.6 ± 1.9 bc	84.8 ± 0.0 e	**81.4**
**B3**	Br	3′-F	83.3 ± 1.9 c	86.1 ± 1.0 bc	66.8 ± 0.8 gh	77.8 ± 0.0 c	77.9 ± 1.1 f	**78.4**
**B4**	Br	4′-F	72.5 ± 1.2 kl	71.1 ± 1.1 h	65.4 ± 2.5 hij	72.9 ± 0.9 f	73.2 ± 1.8 ghi	**71.0**
**B5**	Br	3′-Cl	70.1 ± 1.2 mn	60.4 ± 0.0 op	63.4 ± 0.7 ijk	69.5 ± 0.9 g	56.6 ± 2.1 op	**64.0**
**B6**	Br	4′-Cl	68.5 ± 0.7 no	60.4 ± 0.0 op	61.3 ± 0.7 k	69.5 ± 0.9 g	61.9 ± 2.1 mn	**64.3**
**B7**	Br	3′-Br	69.7 ± 0.7 mno	59.4 ± 0.0 p	63.0 ± 2.1 jk	70.0 ± 0.9 g	54.2 ± 1.0 p	**63.2**
**B8**	Br	4′-Br	67.3 ± 1.4 op	59.8 ± 0.5 op	63.0 ± 0.0 jk	69.5 ± 3.4 g	61.3 ± 1.0 mn	**64.2**
**B9**	Br	4′-I	65.7 ± 0.7 p	62.3 ± 1.9 no	58.0 ± 0.0 l	70.0 ± 0.9 g	56.6 ± 1.0 op	**62.5**
**B10**	Br	2′-OMe	75.7 ± 0.7 ij	71.7 ± 0.0 h	64.6 ± 1.4 hij	77.8 ± 0.0 c	78.6 ± 0.0 f	**73.7**

^a^The difference between the data with the different lowercase letters within a column is significant (*P* < 0.05). ^b^Mean: the average values of inhibition rates of the same compound on the five test fungi.

Subsequently, the compounds with inhibition rates of >50% in Table 2 were further assayed for median effective concentrations (EC_50_) against each fungus to explore their antifungal potential in more detail and SAR. Compound **A1** without substituents on the C-ring, SA and CH were used as reference controls. TBZ and CBD were used as positive controls. The results are listed in Table 3.

**Table 3:** EC_50_ values of compounds **A** and **B** against five fungi.

Compounds			EC_50_ μM (μg/mL)					Mean (μM)^a^
No.	R	R′	*F. solani*	*F. graminearum*	*V. mali*	*C. lunata*	*C. gloeosporioides*	
**A1**	H	H	49.8 (17.3)	84.2 (29.3)	112 (39.0)	73.1 (25.5)	104 (36.3)	84.6
**A2**	H	2′-F	6.98 (2.55)	21.1 (7.71)	19.0 (6.95)	23.7 (8.66)	24.0 (8.76)	19.0
**A3**	H	3′-F	23.1 (8.43)	23.9 (8.73)	19.4 (7.07)	22.9 (8.36)	15.6 (5.69)	21.0
**A4**	H	4′-F	19.9 (7.28)	27.0 (9.87)	85.8 (31.3)	42.3 (15.4)	>75^b^	
**A5**	H	2′-Cl	2.38 (0.91)	18.9 (7.24)	16.4 (6.28)	18.0 (6.89)	15.8 (6.04)	14.3
**A6**	H	3′-Cl	14.6 (5.60)	17.7 (6.76)	23.7 (9.07)	8.02 (3.07)	20.9 (8.01)	17.0
**A7**	H	4′-Cl	9.27 (3.55)	10.8 (4.12)	18.7 (7.14)	8.07 (3.09)	19.2 (7.35)	13.2
**A8**	H	2′-Br	**6.08** (**2.60**)	**5.79** (**2.47**)	**11.1** (**4.75**)	**13.8** (**5.90**)	**15.9** (**6.78**)	**10.5**
**A9**	H	3′-Br	7.55 (3.23)	11.3 (4.81)	22.8 (9.76)	10.5 (4.47)	20.4 (8.73)	14.5
**A10**	H	4′-Br	**6.48** (**2.77**)	**3.35** (**1.43**)	**23.6** (**10.1**)	**2.76** (**1.18**)	**16.0** (**6.84**)	**10.4**
**A11**	H	2′-I	**1.83** (**0.87**)	**10.8** (**5.13**)	**13.8** (**6.53**)	**7.55** (**3.58**)	**16.3** (**7.72**)	**10.1**
**A12**	H	3′-I	6.05 (2.87)	11.2 (5.33)	20.1 (9.52)	14.1 (6.68)	26.3 (12.5)	15.6
**A13**	H	4′-I	**2.30** (**1.09**)	**12.0** (**5.71**)	**18.9** (**8.94**)	**7.43** (**3.52**)	**13.2** (**6.28**)	**10.8**
**A14**	H	2′,6′-diF	8.45 (3.25)	12.5 (4.81)	17.7 (6.81)	11.1 (4.26)	18.8 (7.22)	13.7
**A15**	H	2′,4′-diCl	**1.74** (**0.72**)	**4.86** (**2.03**)	**12.9** (**5.40**)	**6.96** (**2.90**)	**12.9** (**5.36**)	**7.87**
**A16**	H	3′-NO_2_	25.3 (9.97)	12.7 (5.00)	31.1 (12.2)	18.3 (7.20)	15.1 (5.96)	20.5
**A17**	H	2′-CF_3_	10.2 (4.26)	28.7 (11.9)	17.6 (7.32)	17.9 (7.44)	19.6 (8.17)	18.8
**A18**	H	3′-CF_3_	19.9 (8.28)	13.9 (5.80)	25.9 (10.8)	12.6 (5.25)	23.5 (9.78)	19.2
**A19**	H	3′-CN	12.9 (4.80)	21.9 (8.16)	44.1 (16.4)	32.7 (12.2)	27.4 (10.2)	27.8
**A20**	H	2′-CH_3_	60.6 (22.0)	>75^b^	>75^b^	>75^b^	>75^b^	
**A21**	H	3′-CH_3_	39.3 (14.2)	62.1 (22.5)	>75^b^	75.5 (27.3)	>75^b^	
**A22**	H	4′-CH_3_	38.2 (13.9)	64.0 (23.2)	>75^b^	60.5 (21.9)	>75^b^	
**A24**	H	3′-OMe	89.2 (33.7)	78.4 (29.7)	>75^b^	68.7 (26.0)	79.6 (30.1)	
**B1**	Br	H	6.18 (2.64)	15.0 (6.39)	23.7 (10.1)	14.2 (6.05)	22.6 (9.67)	16.3
**B2**	Br	2′-F	**9.38** (**4.18**)	**10.2** (**4.56**)	**12.2** (**5.42**)	**9.45** (**4.21**)	**14.2** (**6.32**)	**11.1**
**B3**	Br	3′-F	16.7 (7.41)	17.2 (7.67)	23.3 (10.4)	15.0 (6.67)	25.3 (11.3)	19.5
**B4**	Br	4′-F	10.5 (4.70)	27.7 (12.3)	17.7 (7.87)	14.2 (6.33)	22.4 (9.98)	18.5
**B5**	Br	3′-Cl	11.1 (5.12)	16.3 (7.52)	22.5 (10.4)	13.2 (6.09)	35.7 (16.5)	19.8
**B6**	Br	4′-Cl	14.2 (6.57)	21.2 (9.77)	22.0 (10.1)	16.4 (7.57)	28.7 (13.3)	20.5
**B7**	Br	3′-Br	9.27 (4.69)	13.9 (7.02)	22.7 (11.5)	24.6 (12.5)	39.8 (20.1)	22.1
**B8**	Br	4′-Br	10.2 (5.14)	21.7 (11.0)	23.3 (11.8)	23.9 (12.1)	25.4 (12.8)	20.9
**B9**	Br	4′-I	27.9 (15.4)	40.7 (22.5)	65.9 (36.4)	62.8 (34.8)	59.6 (33.0)	51.4
**B10**	Br	2′-OMe	12.7 (5.81)	19.4 (8.89)	32.7 (15.0)	29.3 (13.4)	28.9 (13.2)	24.6
**Sanguinarine (SA)**			21.6 (9.90)	30.0 (13.8)	35.8 (16.4)	27.6 (12.7)	37.0 (17.0)	30.4
**Chelerythrine (CH)**			23.2 (11.0)	47.3 (22.5)	31.8 (15.1)	23.4 (11.1)	141 (67.0)	53.3
**Thiabendazole (TBZ)**			12.5 (2.50)	3.00 (0.60)	11.7 (2.40)	403 (81.1)	2–4 (0.80)	
**Carbendazim (CBD)**			4.34 (0.83)	3.83 (0.73)	4.08 (0.78)	≈300 (≈57.4)	<2 (<0.38)	

^a^Mean: the average EC_50_ values of the same compound against the five fungi. ^b^Estimated EC_50_ values based on the results in Tables 1 and 2.

Except for **A1**, all the compounds presented the good to excellent activity on each fungus. Among them, 26 compounds showed the higher average activity with average EC_50_ values of 7.87 to 27.8 μM for the five fungi than both SA (EC_50_ = 30.4 μM) and CH (EC_50_ = 53.3 μM), and of which **A15** showed the highest average activity. Compared with commercial fungicides TBZ (EC_50_ = 403 μM) and CBD (EC_50_ ≈ 300 μM), all the compounds showed the much higher activity (EC_50_ = 2.76–75.5 μM) against *C. lunata*. For *F. solani*, 17 compounds (EC_50_ = 1.74–11.1 μM) were more active than TBZ (EC_50_ = 12.5 μM) but lower than CBD (EC_50_ = 4.34 μM). As far as *F. graminearum* was concerned, **A10** showed the highest activity (EC_50_ = 3.35 μM), superior to CBD (EC_50_ = 3.83 μM), while **A8** and **A15** showed the higher activity (EC_50_ = 5.79, 4.86 μM), close to TBZ (EC_50_ = 3.00 μM) and CBD. For *V. mali*, **A8** showed the smallest EC_50_ value of 11.1 μM, more active than TBZ (EC_50_ = 11.7 μM) but inferior to CBD (EC_50_ = 4.08 μM), and followed by **B2** and **A15** (EC_50_ = 12.2, 12.9 μM). For *C. gloeosporioides*, all the compounds except **A24** and **B7** showed the higher activity (EC_50_ = 12.9–35.7 μM) than both sanguinarine and chelerythrine (EC_50_ = 37.0, 114 μM) but much lower than TBZ (EC_50_ = 2–4 μM) and CBD (EC_50_ < 2 μM). The results above suggested the rationality of structure design of the target compounds to some extent.

### Structure-activity relationship

By comparison of the EC_50_ values of the compounds in Table 3 and their initial activity in Tables 1 and 2, we analyzed the SAR of compounds **A** and **B**. From Tables 4 and 5

**Table 4:** Effects of the substituents (R′) on C-ring on the activity of compounds A.


	
Fungus	F, Cl, Br or I	CF_3_	NO_2_	CN	OH	OMe		Me		
	*o*-, *m*- or *p*-	*o*- or *m*-	*m*-	***m*** **-**	*o*-, *m*- or *p*-	*o*- or *p*-	*m*-	*o*-	*m*-	*p*-
*F. solani*										
*F. graminearum*										
*V. mali*										
*C. lunata*										
*C. gloeosporioides*										

Significantly increasing the activity relative to compound **A1** (R′ = H);  Significantly decreasing the activity;  No significant effect on the activity.

**Table 5:** Effects of the substituents (R′) on C-ring and 5-Br on the A-ring on the activity.

Fungus																
	
	F			Cl		Br		*p*-I or *o*-OMe	F			Cl		Br	*p*-I	*o*-OMe
	*o*-	*m*-	*p*-	*m*-	*p*-	*m*-	*p*-		*o*-	*m*-	*p*-	*m*-	*p*-	*m*- or *p*-		
*F. solani*																
*F. graminearum*																
*V. mali*																
*C. lunata*																
*C. gloeosporioides*																

Significantly increasing the activity relative to compound **B1** or the corresponding compounds **A** with the same substituents on the C-ring.  Significantly decreasing the activity;  No significant effect on the activity.

, it is concluded that the substitution patterns on both the C-ring and the A-ring can significantly influence the antifungal activity of the target compounds. The general trends are as follows.

A. The type of substituents on the C-ring can significantly affect the activity. Compared with **A1** without substituents on the C-ring, the presence of all electron-withdrawing groups like halogen atoms (**A2**–**A15**), nitro (**A16**), trifluoromethyl (**A17**, **A18**) or cyano (**A19**) on the C-ring can significantly increase the activity against each fungus. However, the opposite was observed for electron-donating groups like hydroxyl (**A26**–**A28**), 2′-Me (**A20**), 2′-OMe (**A23**) or 4′-OMe (**A25**) (Table 4). On the other hand, comparison of **B2**–**B10** and **B1** (R′ = H) showed that except for 2′-F (**B2**), all the substituents on the C-ring caused significant decrease on the activity of **B** class of compounds in most cases, which is very different from or even opposite to that of **A** class of compounds (Table 5). Obviously, the effect of substiutents on the C-ring also depends on the substituent on the A-ring.

B. For the same substituent, the site of substituents on the C-ring can also significantly influence the activity (Table 3), but this impact varies with the type of substituents and species of fungi. For example, 2′-brominated compound (**A8**) showed the highest activities against *F. solani*, *V. mali* and *C. gloeosporioides* among three isomers (**A8**–**A10**), whereas 4′-brominated compound (**A10**) gave the highest activity against *F. graminearum* and *C. lunata*. Unlike brominated compounds, 3′-F compound (**A3**) showed the highest activity against *C. lunata* and *C. gloeosporioides* among three isomers (**A2**–**A4**). For methylate- or methoxylated compounds, only 3′-Me, 4′-Me or 3′-OMe isomer can give improvement of the activity in most cases (**A21**, **A22**, **A24**, Table 2).

C. The presence of double halogen atoms can obviously increase the activity compared with the corresponding mono-halogenated compounds (**A14** vs **A2**–**A4**; **A15** vs **A5**–**A7**) (Table 3).

D. Comparison of the activities of various compounds **B** (R = Br) and the corresponding compounds **A** (R = H) with the same substituents on the C-ring showed that the effect of 5-Br on the A-ring on the activity varies with the substitution patterns of the C-ring group. When 2′-, 3′- or 4′-F, 2′-OMe or no substituent is present on the C-ring, the introduction of 5-Br can significantly increase the activity against all or most of the fungi (**Bn** vs **An**, n = 1, 2, 3, 4; **B10** vs **A23**). However, the opposite is found when 4′-Cl, 3′-Br, 4′-Br or 4′-I is present (**B7** vs **A9**; **B8** vs **A10**; **B9** vs **A13**). When R′ is 3′-Cl, the introduction of 5-Br only makes a small influence on the activity against all the fungi (**B6** vs **A7**) (Table 5).

The results above show that there is an interaction effect between substituents on the A-ring and the C-ring, and the activity of compounds depends on the combined effect of substituents on both the A-ring and the C-ring. A similar case was found for the antifungal activity of 6-chloro-ADHIQs^9^ and 8-methoxy-ADHIQs^11^ reported previously by us. The facts above strongly suggest that the bioactivity of the compounds should be related with the electron density distribution of the conjugated system. Therefore, it is necessary to extend the modification of the A-ring to discover more potent ADHIQs agents.

## Discussions

The SAR of the title compounds is similar but not completely equal to that of 8-OMe-ADHIQs recently reported by us^11^, and obviously different from that of 6-chloro-ADHIQs^9^. The main difference between the present compounds (7,8-dimethoxy-ADHIQs) and 8-OMe-ADHIQs is the position effect of substituents on C-ring. For the 8-OMe-ADHIQs, the introduction of 3′-Cl to the C-ring gave the highest activity against *F. solani* and *F. graminearum*. For the 6-Cl-ADHIQs, the introduction of 2′-Me, an electron-donating group, led to the highest activity against *C. lunata* and *V. mali*, whereas the presence of halogen atoms on the C-ring did not give significant improvement of the activity in most cases. Compared with the 8-OMe- or 6-Cl-ADHIQs, the majority of the present compounds, especially compounds **A** showed the higher activity against each fungus. The results may be due to the present compounds possessing the higher structural similarity to CH than the 8-OMe or 6-Cl compounds (Fig. 1). Based on the results and analysis above, it is necessary to further explore the antifungal activity of 7,8-methylenedioxy-ADHIQs which are structurally similar to sanguinarine. At present, this work is under way in our lab.

**Figure 3:**
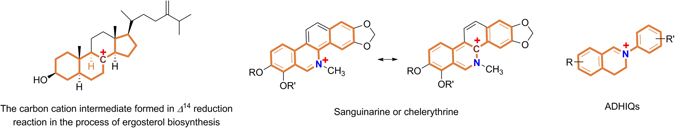
The structures of the carbon cation intermediate formed in the process of ergosterol biosynthesis, sanguinarine, chelerythrine and ADHIQs.

Up to now, no report was found on antifungal mechanism of ADHIQs or QBAs. However, as an analogue of chelerythrine or sanguinarine, berberine had been proved to inhibit the fungus *Aspergillus fumigatus* by targeting ergosterol biosynthesis pathway^20^. Based on the structural similarity, we conjecture that chelerythrine, sanguinarine and ADHIQs may have the same or similar action mechamism to berberine. Ergosterol biosynthesis process includes a *Δ* ^14^ reduction reaction involving a tertiary carbon cation intermediate (Fig. 3)^21^. Quaternary ADHIQ ions or QBAs have very high structural similarity to the intermediate. Therefore, we think that ADHIQs or QBAs may be inhibitors of *Δ* ^14^ reductase in ergosterol biosynthesis. This also is what we are going to do next.

As natural compounds, sanguinarine and chelerythrine possess very high safety to mammal such as pigs or mice^22, 23^. QBAs have been used as antimicrobial agents to treat oral disease^24^, dermatomycosis^25^ or expectorant^26^. Although we don’t know whether ADHIQs have high safety to mammal like QBAs at present, our previous study showed that ADHIQs have lower toxicity to normal cells than cancer cells^5, 16^. Additionally, our study also proved that ADHIQs have no effect on seed germination and seeding growth of plants (*Panicum miliaceum* L. and *Brassica campestris* L.)^17, 18^. Interestingly, some ADHIQs were found to have growth-promoting action for plants. Therefore, ADHIQs can be considered as promising and potent antifungal agents.

In conclusion, a series of new 2-aryl-7,8-dimethoxy-3,4-dihydroisoquinolin-2-ium bromides were designed, synthesized and evaluated for antifungal activity *in vitro* against the five plant pathogenic fungi in the present study. The majority of the compounds showed good to excellent inhibition activity with average EC_50_ values of 7.87–20.0 μM for the five fungi, superior to their natural model compounds SA and CH. Part of the compounds showed the higher activity against *F. solani*, *F. graminearum* and *C. gloeosporioides* than commercial fungicides TBZ or CBD. Compared with other series 2-aryl-3,4-dihydroisoquinolin-2-iums with or without substituents on the A-ring, the present compounds showed the highest activity in most cases. Compounds **A8**, **A10**, **A11**, **A13**, **A15** and **B2** possessed very great potential to be developed as new antifungal agents. In addition, SAR was derived also. It was found that substitution patterns of both the C-ring and A-ring significantly affect the activity. There exists an interaction effect between substituents on the A-ring and the C-ring, and the activity of compounds depends on the combined effect of substituents on the two aryl-rings. The presence of electron-withdrawing groups on the C-ring can significantly increase the activity. These findings will be of great importance for the design of more potent antifungal 2-aryl-3,4-dihydroisoquinolin-2-ium agents. It is necessary to conduct more extensive structural modification, especially for the A-ring, determination of an antifungal spectrum and the *in vivo* activity of these compounds.

## Methods

### Chemicals

Thiabendazole (TBZ, ≥99.1%), azoxystrobin (ASB, ≥98%), and carbendazim (CBD, ≥98%), three commercial fungicides, were purchased from Sigma-Aldrich Trading Co. Ltd. (Shanghai, China). Sanguinarine iodide (SA, >98%) and chelerythrine iodide (CH, >99%) were obtained by the isolation from the plant of *Macleaya microcarpa* (Maxim.) Fedde in our laboratory^27^. 2-(3,4-Dimethoxyphenyl)acetic acid and dimethyl sulfoxide (DMSO) were purchased from J&K Chemical Ltd. (Beijing, China). All anhydrous reagents and solvents were obtained by dehydration according to the standard methods before use. Bromine was dried with concentrated H_2_SO_4_ in a separating funnel. CH_2_Cl_2_ and toluene was distilled from CaH_2_. Methanol was distilled from sodium. THF was freshly distilled from CaH_2_ and sodium. Other reagents and solvents were obtained locally and of analytical grade.

### Fungi

The plant pathogenic fungi, *Fusarium solani, Fusarium graminearum, Valsa mali, Curvularia lunata* and *Colletotrichum gloeosporioides*, were provided by the Center of Pesticide Research, Northwest A&F University, China. The fungi were grown on potato dextrose agar (PDA) plates at 25 °C and maintained at 4 °C with periodic subculturing.

### Instruments

Melting points were determined on an mp420 automatic melting point meter (Hanon Instrument, Beijing, China) and uncorrected. NMR spectra were recorded on a BrukerAvance III 500 MHz instrument. Chemical shift values (*δ*) were given in parts per million (ppm). Coupling constant values (*J*) were given in Hz. High resolution mass spectra (HR-MS) and low resolution mass spectra (LR-MS) were carried out with Micromass Auto Spec-3000 instrument and Thermo FisherLCQ Fleet instrument, respectively.

### Synthesis

#### Synthesis of 2-(2-bromo-4,5-dimethoxyphenyl)acetic acid (**1**)

Bromine (17.60 g, 0.11 mol) in 55 mL anhydrous CH_2_Cl_2_ was dropwise added into a solution of 2-(3,4-dimethoxyphenyl)acetic acid (19.62 g, 0.10 mol) in 60 mL dry CH_2_Cl_2_ under ice bath. The resulting mixture were stirred at room temperature for 24 h, and extracted with saturated Na_2_SO_3_ aqueous solution (3 × 80 mL). To the aqueous phase was added 2 M HCl aqueous solution until the product was completely precipitated. The precipitates were filtered off and dried at 55 °C in vacuum to yield **1** as white solids (25.97 g) in 94% yield. ^1^H NMR (500 MHz, CDCl_3_) *δ*: 7.03 (1H, s), 6.79 (1H, s), 3.86 (3H, s), 3.85 (3H, s), 3.76 (2H, s). ^13^C NMR (125 MHz, CDCl_3_) *δ*: 176.5, 149.1, 148.6, 125.4, 115.7, 115.2, 114.1, 56.3, 56.2, 41.0.

#### Synthesis of 2-(2-bromo-4,5-dimethoxyphenyl)ethan-1-ol (**2**)

To a solution of **1** (13.76 g, 0.05 mol) in 150 mL dry THF was portion-wise added NaBH_4_ (5.67 g, 0.15 mol) under ice bath. After no gas was liberated, iodine (12.65 g, 0.05 mol) in 80 mL dry THF was dropwise added into the mixture, and then stirred at 40 °C until the reaction was completed. The reaction was quenched with methanol until no gas was generated. After evaporated under reduced pressure, to the residue was added 100 mL water, and then extracted with CH_2_Cl_2_ (3 × 80 mL). The organic phase was sequentially washed with saturated sodium sulfite solution (3 × 80 mL) and saturated brine (80 mL), and dried over anhydrous MgSO_4_. After filtration and concentration, crude **2** was obtained and directly used for the following reaction.

#### Synthesis of 5-bromo-7,8-dimethoxyisochromane (**3**)

Paraformaldehyde (1.65 g, 55 mmol) was fully dissolved in 50 mL trifluoroacetic acid with assistance of ultrasonic wave. The resulting solution was poured into **2** and stirred at room temperature until the reaction was completed. To the solution was added 100 mL water, extracted with CH_2_Cl_2_ (3 × 100 mL), sequentially washed with saturated sodium sulfite solution (150 mL), saturated sodium bicarbonate solution (2 × 100 mL) and saturated brine (100 mL). The organic phase was dried over anhydrous MgSO_4_. After filtration and concentration, the residue was purified by column chromatography on silica gel using petroleum ether-ethyl acetate (30:1) to yield **3** as white solids (10.29 g) in 75% yield for two steps. ^1^H NMR (500 MHz, CDCl_3_) *δ*: 7.03 (1H, s), 4.74 (2H, s), 3.91 (2H, t, *J* = 5.7 Hz), 3.84 (s-like, 3H), 3.80 (s-like, 3H), 2.70 (2H, t, *J* = 5.7 Hz). ^13^C NMR (125 MHz, CDCl_3_) *δ*: 150.8, 144.0, 131.0, 126.1, 118.6, 114.9, 65.2, 64.5, 60.2, 56.2, 28.7.

#### Synthesis of 7,8-dimethoxyisochromane (**4**)

To a solution of **3** (10.29 g, 37.7 mmol) in 150 mL dry THF was dropwise added 2.7 M n-butyllithium in hexane (20.9 mL, 56.5 mmol) under argon at −78 °C. The solution was stirred at −78 °C for 2 h, then quenched and extracted with water (200 mL). The organic phase was collected. The aqueous phase was extracted with diethyl ether (3 × 200 mL), washed with saturated brines and dried over anhydrous MgSO_4_. After filtration, the solution was evaporated under reduced pressure. The residue was purified by column chromatography on silica gel using petroleum ether-ethyl acetate (30:1) to yield **4** as pale yellow oils (5.79 g) in 79% yield. ^1^H NMR (500 MHz, CDCl_3_) *δ*: 6.82 (1H, d, *J* = 8.4 Hz), 6.78 (1H, d, *J* = 8.4 Hz), 4.80 (2H, s), 3.90 (2H, t, *J* = 5.7 Hz), 3.84 (3H, s), 3.82 (3H, s), 2.78 (2H, t, *J* = 5.7 Hz). ^13^C NMR (125 MHz, CDCl_3_) *δ*: 150.4, 144.7, 129.0, 126.6, 124.1, 111.1, 65.3, 64.7, 60.3, 56.0, 27.7.

#### Synthesis of 1,7,8-trimethoxyisochromane (**5**)

To a solution of **4** (5.79 g, 29.8 mmol) in 60 mL dry CH_2_Cl_2_ was added DDQ (7.94 g, 35 mmol) and anhydrous methanol (1.5 mL, 35.8 mmol), and then stirred under argon at room temperature for 24 h. After the reaction was completed, saturated sodium bicarbonate (80 mL) was added into the solution. The organic phase was collected, and the aqueous phase was extracted with CH_2_Cl_2_ (3 × 60 mL). The combined organic phase was washed with water (2 × 150 mL) and saturated brines (1 × 150 mL), and dried over anhydrous MgSO_4_. After filtration, the solution was evaporated under reduced pressure. The residue was chromatographed over silica gel using petroleum ether-ethyl acetate (20:1) to yield **5** as pale yellow oils (4.14 g) in 62% yield. The structure of **5** was unconfirmed and directly used for the following reaction.

#### Synthesis of 5-bromo-1,7,8-trimethoxyisochromane (**6**)

Compound **6** was prepared from **3** according to the same procedure as described for **5**. Briefly, **3** (5.46 g, 20 mmol) reacted with DDQ (5.45 g, 24 mmol) and anhydrous MeOH (1 mL, 24 mmol) to yield **6** (5.02 g) as white solids in 83% yield. ^1^H NMR (500 MHz, CDCl_3_) *δ*: 7.11 (1H, s), 5.59 (1H, s), 4.13 (1H, td, *J* = 12.0, 3.8 Hz, H-3a), 3.94–3.90 (1H, m, H-3b), 3.86 (3H, s), 3.84 (3H, s), 3.55 (3H, s), 2.77–2.70 (1H, m, H-4a), 2.62 (1H, dd, *J* = 16.0, 3.3 Hz, H-4b). ^13^C NMR (125 MHz, CDCl_3_) *δ*: 151.1, 146.1, 130.5, 126.3, 118.4, 116.8, 94.4 (C-1), 61.0, 56.9, 56.2, 55.4, 28.0.

#### Synthesis of 6-(2-bromoethyl)-2,3-dimethoxybenzaldehyde (**7**)

Bu_4_NBr (5.95 g, 18.5 mmol) and TMSBr (4.9 mL, 37 mmol) was added into a solution of **5** (4.14 g, 18.5 mmol) in 19 mL dry toluene at room temperature. The reaction flask was sealed with a glass stopper and stirred at 80 °C for 4 h. To the reaction solution was added 40 mL saturated NaHCO_3_ aqueous solution, and extracted with ethyl acetate (3 × 30 mL). The combined organic phases were washed with saturated brines (1 × 50 mL) and dried over anhydrous MgSO_4_. After filtration, the solution was evaporated under reduced pressure. The residue was chromatographed on silica gel eluting with petroleum ether-ethyl acetate (20:1) to yield **7** as white solids (2.80 g) in 56% yield. ^1^H NMR (500 MHz, CDCl_3_) *δ*: 10.54 (1H, s, CHO), 7.09 (1H, d, *J* = 8.4 Hz), 6.98 (1H, d, *J* = 8.4 Hz), 3.96 (3H, s), 3.90 (3H, s), 3.58 (2H, t, *J* = 7.0 Hz), 3.40 (2H, t, *J* = 7.0 Hz). ^13^C NMR (125 MHz, CDCl_3_) *δ*: 192.6 (CHO), 154.3, 152.1, 132.3, 127.9, 127.6, 117.4, 62.5, 56.1, 37.0, 33.7.

#### Synthesis of 3-bromo-2-(2-bromoethyl)-5,6-dimethoxybenzaldehyde (**8**)

Compound **8** was prepared from **6** according to the same procedure as described for **7**. Briefly, **6** (3.03 g, 10 mmol) reacted with Bu_4_NBr (3.22 g, 10 mmol) and TMSBr (2.7 mL, 20 mmol) to yield **8** (1.04 g) as white solids in 30% yield. ^1^H NMR (500 MHz, CDCl_3_) *δ*: 10.45 (1H, s, CHO), 7.32 (1H, s), 3.94 (3H, s), 3.90 (3H, s), 3.55–3.51 (2H, m), 3.50–3.46 (2H, m). ^13^C NMR (125 MHz, CDCl_3_) *δ*: 191.8 (CHO), 153.2, 152.3, 130.9, 129.8, 121.5, 121.4, 62.4, 56.4, 35.6, 30.5.

#### Synthesis of compounds **A** and **B**

According to the method reported by us, compounds **A** and **B** were synthesized by reaction of aniline or substituted aniline and the corresponding intermediate **7** or **8**. The general procedure was as follows. To the solution of **7** or **8** (1.0 mmol) in 10 mL 1,4-dioxane was added aniline or substituted aniline (1.0 mmol) under ice bath. The solution was stirred overnight at room temperature to produce yellow precipitate. The precipitate was filtered off and washed with a small volume of diethyl ether to yield compounds **A** or **B**.

#### 7,8-Dimethoxy-2-phenyl-3,4-dihydroisoquinolin-2-ium bromide (**A1**)

Yield, 58%; yellow solids; mp 145.4–146.4 °C; ^1^H NMR (500 MHz, CD_3_OD) *δ*: 9.35 (1H, s, H-1), 7.80 (2H, d, *J* = 8.3 Hz), 7.71–7.65 (3H, m), 7.56 (1H, d, *J* = 8.3 Hz), 7.19 (1H, d, *J* = 8.3 Hz), 4.56 (2H, t, *J* = 7.8 Hz), 4.14 (3H, s), 3.96 (3H, s), 3.38 (2H, t, *J* = 7.8 Hz); ^13^C NMR (125 MHz, CD_3_OD) *δ*: 164.1 (C-1), 154.2, 152.8, 145.0, 132.2, 131.4, 129.6, 125.0, 123.9, 123.8, 120.1, 63.0, 56.9, 53.1, 26.1; HR-ESI-MS [M–Br]^+^: Calcd for C_17_H_18_NO_2_^+^, 268.1332, found 268.1313; Negative ESI-MS m/z: 78.67 [^79^Br]^−^, 80.66 [^81^Br]^−^.

#### 2-(2-Fluorophenyl)-7,8-dimethoxy-3,4-dihydroisoquinolin-2-ium bromide (**A2**)

Yield, 60%; yellow solids; mp 105.3–108.2 °C; ^1^H NMR (500 MHz, CD_3_OD) *δ*: 9.45 (1H, s, H-1), 7.87 (1H, td, *J* = 7.9, 1.6 Hz), 7.73–7.68 (1H, m), 7.61 (1H, d, *J* = 8.3 Hz), 7.53–7.48 (2H, m),7.21 (1H, d, *J* = 8.3 Hz), 4.47 (2H, t, *J* = 7.8 Hz), 4.14 (3H, s), 3.97 (3H, s), 3.38 (2H, t, *J* = 7.8 Hz); ^13^C NMR (125 MHz, CD_3_OD) *δ*: 167.8 (C-1), 156.5 (d, *J* = 252.5 Hz, C-2′), 154.6, 152.8, 134.4 (d, *J* = 8.2 Hz), 132.4 (d, *J* = 11.4 Hz), 129.9, 127.4, 127.1 (d, *J* = 4.0 Hz), 125.7, 124.1, 119.9, 118.6 (d, *J* = 19.1 Hz), 63.1, 57.0, 53.9 (d, *J* = 2.7 Hz, C-3), 26.2; HR-ESI-MS [M–Br]^+^: Calcd for C_17_H_17_FNO_2_^+^, 286.1238, found 286.1226.

#### 2-(3-Fluorophenyl)-7,8-dimethoxy-3,4-dihydroisoquinolin-2-ium bromide (**A3**)

Yield, 59%; yellow solids; mp 112.6–114.0 °C; ^1^H NMR (500 MHz, CD_3_OD) *δ*: 9.39 (1H, s, H-1), 7.71–7.65 (3H, m), 7.58 (1H, d, *J* = 8.2 Hz), 7.43 (1H, t, *J* = 8.4 Hz), 7.19 (1H, d, *J* = 8.2 Hz), 4.54 (2H, t, *J* = 7.5 Hz), 4.15 (3H, s), 3.96 (3H, s), 3.37 (2H, t, *J* = 7.5 Hz). ^13^C NMR (125 MHz, CD_3_OD) *δ*: 165.0 (C-1), 164.3 (d, *J* = 249.0 Hz, C-3′), 154.5, 152.8, 146.0, 133.2 (d, *J* = 9.1 Hz), 129.8, 125.3, 123.9, 120.0, 119.9, 119.1 (d, *J* = 21.8 Hz), 111.8 (d, *J* = 26.8 Hz), 63.0, 57.0, 53.1, 26.1. ESI-MS m/z 286.15 [M–Br]^+^.

#### 2-(4-Fluorophenyl)-7,8-dimethoxy-3,4-dihydroisoquinolin-2-ium bromide (**A4**)

Yield, 73%; yellow solid; mp 167.9–168.8 °C; ^1^H NMR (500 MHz, CD_3_OD) *δ*: 9.35 (1H, s, H-1), 7.89–7.85 (2H, m), 7.57 (1H, d, J = 8.3 Hz), 7.42 (2H, t, *J* = 8.6 Hz), 7.19 (1H, d, *J* = 8.3 Hz), 4.53 (2H, t, *J* = 7.7 Hz), 4.14 (3H, s), 3.96 (3H, s), 3.37 (2H, t, *J* = 7.7 Hz). ^13^C NMR (125 MHz, CD_3_OD) *δ*: 165.0 (d, *J* = 251.0 Hz, C-4′), 164.4 (C-1), 154.2, 152.8, 141.2, 129.5, 126.5 (d, *J* = 9.4 Hz), 125.0, 123.9, 120.1, 118.2 (d, *J* = 24.5 Hz), 63.0, 57.0, 53.3, 26.1. ESI-MS m/z 286.14 [M–Br]^+^.

#### 2-(2-Chlorophenyl)-7,8-dimethoxy-3,4-dihydroisoquinolin-2-ium bromide (**A5**)

Yield, 47%; yellow solids; mp 149.3–150.0 °C; ^1^H NMR (500 MHz, CD_3_OD) *δ*: 9.51 (1H, s, H-1), 7.86 (1H, d, *J* = 7.7 Hz), 7.78 (1H, dd, *J* = 7.9, 1.3 Hz), 7.70–7.61 (3H, m), 7.22 (1H, d, *J* = 8.3 Hz), 4.40 (2H, t, *J* = 7.7 Hz), 4.12 (3H, s), 3.97 (3H, s), 3.42 (2H, t, *J* = 7.7 Hz). ^13^C NMR (125 MHz, CD_3_OD) *δ*: 168.7 (C-1), 154.7, 152.8, 142.0, 133.8, 132.3, 130.2, 129.8, 129.6, 128.3, 125.7, 124.2, 119.7, 63.1, 57.0, 53.9, 26.2. HR-ESI-MS [M–Br]^+^: Calcd for C_17_H_17_ClNO_2_^+^, 302.0942, found 302.0946.

#### 2-(3-Chlorophenyl)-7,8-dimethoxy-3,4-dihydroisoquinolin-2-ium bromide (**A6**)

Yield, 75%; yellow solids; mp 141.7–142.7 °C; ^1^H NMR (500 MHz, CD_3_OD) *δ*: 9.40 (1H, s, H-1), 7.93 (1H, s, H-2′), 7.75 (1H, d, *J* = 7.0 Hz), 7.69–7.65 (2H, m), 7.57 (1H, d, *J* = 8.3 Hz), 7.19 (1H, d, *J* = 8.3 Hz), 4.53 (2H, t, *J* = 7.7 Hz), 4.14 (3H, s), 3.96 (3H, s), 3.36 (2H, t, *J* = 7.7 Hz). ^13^C NMR (125 MHz, CD_3_OD) *δ*: 165.1 (C-1), 154.5, 152.8, 145.9, 136.8, 132.7, 132.2, 129.7, 125.3, 124.4, 123.9, 122.5, 120.0, 63.0, 57.0, 53.1, 26.1. ESI-MS *m*/*z* 302.13 [M–Br]^+^.

#### 2-(4-Chlorophenyl)-7,8-dimethoxy-3,4-dihydroisoquinolin-2-ium bromide (**A7**)

Yield, 73%; yellow solids; mp 155.1–156.2 °C; ^1^H NMR (500 MHz, CD_3_OD) *δ*: 9.37 (1H, s, H-1), 7.82 (2H, dt, *J* = 8.9, 2.8 Hz), 7.68 (2H, dt, *J* = 8.9, 2.8 Hz), 7.57 (1H, d, *J* = 8.3 Hz), 7.19 (1H, d, *J* = 8.3 Hz), 4.54 (2H, t, *J* = 7.7 Hz), 4.14 (3H, s), 3.96 (3H, s), 3.37 (2H, t, *J* = 7.7 Hz). ^13^C NMR (125 MHz, CD_3_OD) *δ*: 164.5 (C-1), 154.3, 152.8, 143.5, 138.0, 131.4, 129.6, 125.6, 125.2, 123.9, 120.1, 63.0, 57.0, 53.1, 26.1. ESI-MS *m*/*z* 302.12 [M–Br]^+^.

#### 2-(2-Bromophenyl)-7,8-dimethoxy-3,4-dihydroisoquinolin-2-ium bromide (**A8**)

Yield, 68%; yellow solids; mp 157.1–158.4 °C; ^1^H NMR (500 MHz, CD_3_OD) *δ*: 9.51 (1H, s, H-1), 7.94 (1H, dd, *J* = 8.0, 0.9 Hz), 7.84 (1H, d, *J* = 7.7 Hz), 7.68 (1H, t, *J* = 7.3 Hz), 7.63–7.58 (2H, m), 7.22 (1H, d, *J* = 8.3 Hz), 4.38 (2H, t, *J* = 7.7 Hz), 4.12 (3H, s), 3.97 (3H, s), 3.44 (2H, t, *J* = 7.7 Hz). ^13^C NMR (125 MHz, CD_3_OD) *δ*: 168.8 (C-1), 154.8, 152.9, 143.7, 135.5, 133.9, 130.9, 129.7, 128.3, 125.8, 124.3, 119.6, 118.9, 63.1, 57.0, 54.0, 26.3. HR-MS [M- Br]^+^: Calcd for C_17_H_17_BrNO_2_^+^, 346.0437, found 346.0430.

#### 2-(3-Bromophenyl)-7,8-dimethoxy-3,4-dihydroisoquinolin-2-ium bromide (**A9**)

Yield, 66%; yellow solids; mp 148.3–149.5 °C; ^1^H NMR (500 MHz, CD_3_OD) *δ*: 9.39 (1H, s, H-1), 8.07 (1H, s, H-2′), 7.84–7.79 (2H, m), 7.61–7.57 (2H, m), 7.19 (1H, d, *J* = 8.3 Hz), 4.52 (2H, t, *J* = 7.8 Hz), 4.14 (3H, s), 3.96 (3H, s), 3.36 (2H, t, *J* = 7.8 Hz). ^13^C NMR (125 MHz, CD_3_OD) *δ*: 165.1 (C-1), 154.5, 152.8, 145.9, 135.2, 132.9, 129.7, 127.2, 125.2, 124.3, 123.9, 122.9, 120.0, 63.0, 57.0, 53.1, 26.1. ESI-MS *m*/*z*: 346.09, 348.07 [M–Br]^+^.

#### 2-(4-Bromophenyl)-7,8-dimethoxy-3,4-dihydroisoquinolin-2-ium bromide (**A10**)

Yield, 64%; yellow solids; mp 140.7–141.1 °C; ^1^H NMR (500 MHz, CD_3_OD) *δ*: 9.38 (1H, s, H-1), 7.84 (2H, d, *J* = 8.8 Hz), 7.74 (2H, d, *J* = 8.8 Hz), 7.57 (1H, d, *J* = 8.3 Hz), 7.19 (1H, d, *J* = 8.3 Hz), 4.53 (2H, t, *J* = 7.8 Hz), 4.14 (3H, s), 3.96 (3 H, s), 3.36 (2 H, t, *J* = 7.8 Hz). ^13^C NMR (125 MHz, CD_3_OD) *δ*: 164.5 (C-1), 154.3, 152.8, 143.9, 134.5, 129.6, 126.0, 125.7, 125.1, 123.9, 120.1, 63.0, 57.0, 53.0, 26.1. HR-ESI-MS [M–Br]^+^: Calcd for C_17_H_17_BrNO_2_^+^, 346.0437, found 346.0433.

#### 2-(2-Iodophenyl)-7,8-dimethoxy-3,4-dihydroisoquinolin-2-ium bromide (**A11**)

Yield, 73%; yellow solids; mp 173.9–175.2 °C; ^1^H NMR (500 MHz, CD_3_OD) *δ*: 9.49 (1H, s, H-1), 8.15 (1H, dd, *J* = 8.0, 1.2 Hz), 7.80 (1H, dd, *J* = 8.0, 1.4 Hz), 7.68 (1H, dt, *J* = 7.9, 1.3 Hz), 7.62 (1H, d, *J* = 8.3 Hz), 7.41 (1H, dt, *J* = 7.8, 1.5 Hz), 7.23 (1H, d, *J* = 8.3 Hz), 4.36 (2 H, t, *J* = 7.8 Hz), 4.13 (3 H, s), 3.97 (3 H, s), 3.49 (2 H, t, *J* = 7.8 Hz). ^13^C NMR (125 MHz, CD_3_OD) *δ*: 168.7 (C-1), 154.8, 152.9, 147.4, 141.9, 133.7, 131.6, 129.6, 127.5, 125.9, 124.3, 119.5, 94.1, 63.2, 57.0, 54.2, 26.4. HR-ESI-MS [M–Br]^+^: Calcd for C_17_H_17_INO_2_^+^, 394.0298, found 394.0303.

#### 2-(3-Iodophenyl)-7,8-dimethoxy-3,4-dihydroisoquinolin-2-ium bromide (**A12**)

Yield, 43%; yellow solids; mp 136.6–137.4 °C; ^1^H NMR (500 MHz, CD_3_OD) *δ*: 9.37 (1H, s, H-1), 8.21 (1H, s, H-2′), 8.02 (1H, d, *J* = 7.9 Hz), 7.80 (1H, dd, *J* = 8.1, 1.6 Hz), 7.57 (1H, d, *J* = 8.3 Hz), 7.43 (1H, t, *J* = 8.0 Hz), 7.18 (1H, d, *J* = 8.3 Hz), 4.50 (2 H, t, *J* = 7.8 Hz), 4.14 (3 H, s), 3.96 (3 H, s), 3.35 (2 H, t, *J* = 7.8 Hz). ^13^C NMR (125 MHz, CD_3_OD) *δ*: 165.0 (C-1), 154.4, 152.8, 145.7, 141.3, 132.8, 132.7, 129.7, 125.2, 123.9, 123.4, 120.0, 95.3, 63.0, 57.0, 53.1, 26.1. ESI-MS *m*/*z*: 394.03 [M–Br]^+^.

#### 2-(4-Iodophenyl)-7,8-dimethoxy-3,4-dihydroisoquinolin-2-ium bromide (**A13**)

Yield, 73%; yellow solids; mp 155.9–157.0 °C; ^1^H NMR (500 MHz, CD_3_OD) *δ*: 9.38 (1H, s, H-1), 8.03 (2 H, d, *J* = 8.8 Hz), 7.58–7.56 (3 H, m), 7.18 (1H, d, *J* = 8.3 Hz), 4.52 (2 H, t, *J* = 7.8 Hz), 4.14 (3 H, s), 3.96 (3 H, s), 3.36 (2 H, t, *J* = 7.8 Hz). ^13^C NMR (125 MHz, CD_3_OD) *δ*: 164.3 (C-1), 154.3, 152.8, 144.5, 140.6, 129.6, 125.5, 125.2, 123.9, 120.1, 97.7, 63.0, 57.0, 52.9, 26.1. ESI-MS *m*/*z*: 394.06 [M–Br]^+^.

#### 2-(2,6-Ddifluorophenyl)-7,8-dimethoxy-3,4-dihydroisoquinolin-2-ium (**A14**)

Yield, 77%; yellow solid; mp 138.2–139.4 °C; ^1^H NMR (500 MHz, CD_3_OD) *δ*: 9.63 (1H, s, H-1), 7.77–7.71 (1H, m), 7.66 (1H, d, *J* = 8.3 Hz), 7.40 (2 H, t, *J* = 8.5 Hz), 7.23 (1H, d, *J* = 8.3 Hz), 4.44 (2 H, t, *J* = 7.5 Hz), 4.15 (3 H, s), 3.98 (3 H, s), 3.39 (2 H, t, *J* = 7.5 Hz). ^13^C NMR (125 MHz, CD_3_OD) *δ*: 170.7 (C-1), 157.4 (dd, *J* = 254.4, 2.7 Hz, C-2′, 6′), 155.2, 152.8, 134.6 (t, *J* = 10.0 Hz, C-4′), 130.3, 126.6, 124.3, 121.4 (t, *J* = 15.4 Hz, C-1′), 119.7, 114.3 (dd, *J* = 20.0, 3.6 Hz, C-3′, 5′), 63.3, 57.1, 54.0, 26.2. ESI-MS *m*/*z*: 304.13 [M–Br]^+^.

#### 2-(2,4-Dichlorophenyl)-7,8-dimethoxy-3,4-dihydroisoquinolin-2-ium (**A15**)

Yield, 67%; yellow solids; mp 157.5–158.7 °C; ^1^H NMR (500 MHz, CD_3_OD) *δ*: 9.52 (1H, s, H-1), 7.91–7.88 (2 H, m), 7.67 (1H, dd, *J* = 8.6, 2.2 Hz), 7.62 (1H, d, J = 8.3 Hz), 7.22 (1H, d, *J* = 8.3 Hz), 4.39 (2 H, t, *J* = 7.7 Hz), 4.14 (3 H, s), 3.97 (3 H, s), 3.42 (2 H, t, *J* = 7.7 Hz). ^13^C NMR (125 MHz, CD_3_OD) *δ*: 169.1 (C-1), 154.9, 152.8, 140.7, 138.9, 132.0, 130.9, 130.4, 129.9, 129.5, 126.0, 124.2, 119.7, 63.2, 57.1, 53.9, 26.2. HR-ESI-MS [M–Br]^+^: Calcd for C_17_H_16_Cl_2_NO_2_^+^, 336.0553, found 336.0570.

#### 7,8-Dimethoxy-2-(3-nitrophenyl)-3,4-dihydroisoquinolin-2-ium (**A16**)

Yield, 70%; yellow solids; mp 155.7–156.6 °C; ^1^H NMR (500 MHz, CD_3_OD) *δ*: 9.52 (1H, s, H-1), 8.74 (1H, s, H-2′), 8.49 (1H, dd, *J* = 8.2, 1.0 Hz), 8.25 (1H, dd, *J* = 8.0, 1.5 Hz), 7.94 (1H, t, *J* = 8.3 Hz), 7.60 (1H, d, *J* = 8.3 Hz), 7.21 (1H, d, *J* = 8.3 Hz), 4.46 (2 H, t, *J* = 7.7 Hz), 4.16 (3 H, s), 3.97 (3 H, s), 3.41 (2 H, t, *J* = 7.7 Hz). ^13^C NMR (125 MHz, CD_3_OD) *δ*: 166.1 (C-1), 154.7, 152.8, 150.3, 145.4, 132.7, 130.2, 129.8, 126.4, 125.6, 124.0, 120.1, 120.0, 63.1, 57.0, 53.2, 26.1. ESI-MS *m*/*z*: 313.13 [M–Br]^+^.

#### 7,8-Dimethoxy-2-(2-(trifluoromethyl)phenyl)-3,4-dihydroisoquinolin-2-ium bromide (**A17**)

Yield, 45%; yellow solids; mp 153.7–155.0 °C; ^1^H NMR (500 MHz, CD_3_OD) *δ*: 9.60 (1H, s, H-1), 8.04 (1H, d, *J* = 8.0 Hz), 8.00–7.96 (2 H, m), 7.91–7.87 (1H, m), 7.64 (1H, d, *J* = 8.4 Hz), 7.23 (1H, d, *J* = 8.4 Hz), 4.51 (1H, br s, H-3a), 4.35 (1H, br s, H-3b), 4.11 (3 H, s), 3.98 (3 H, s), 3.40 (2 H, br s). ^13^C NMR (125 MHz, CD_3_OD) *δ*: 169.2 (C-1), 155.0, 152.9, 141.6, 136.0, 133.1, 129.6, 129.2 (q, *J* = 5.0 Hz), 128.8, 126.2, 125.9 (q, *J* = 28.9 Hz, C-2′), 124.3, 122.2 (q, *J* = 273.2 Hz, CF_3_), 119.4, 63.1, 57.0, 55.0, 26.0. ESI-MS *m*/*z*: 336.15 [M–Br]^+^.

#### 7,8-Dimethoxy-2-(3-(trifluoromethyl)phenyl)-3,4-dihydroisoquinolin-2-ium (**A18**)

Yield, 45%; yellow solids; mp 162.6–168.3 °C; ^1^H NMR (500 MHz, CD_3_OD) *δ*: 9.46 (1H, s, H-1), 8.21 (1H, s, H-2′), 8.10 (1H, d, *J* = 8.0 Hz), 7.98 (1H, d, *J* = 7.9 Hz), 7.89 (1H, t, *J* = 8.0 Hz), 7.59 (1H, d, *J* = 8.3 Hz), 7.20 (1H, d, *J* = 8.3 Hz), 4.58 (2 H, t, *J* = 7.8 Hz), 4.15 (3 H, s), 3.97 (3 H, s), 3.39 (2 H, t, *J* = 7.8 Hz). ^13^C NMR (125 MHz, CD_3_OD) *δ*: 165.7 (C-1), 154.6, 152.8, 145.4, 133.5 (q, *J* = 33.1 Hz, C-3′), 132.5, 129.7, 128.9, 128.0, 125.4, 124.7 (q, *J* = 272.3 Hz, CF_3_), 123.9, 121.4, 120.1, 63.0, 57.0, 53.2, 26.1. 336.16. ESI-MS *m*/*z*: 336.16 [M–Br]^+^.

#### 2-(3-Cyanophenyl)-7,8-dimethoxy-3,4-dihydroisoquinolin-2-ium (**A19**)

Yield, 86%; yellow solid; mp 154.1–155.5 °C; ^1^H NMR (500 MHz, CD_3_OD) *δ*: 9.48 (1H, s, H-1), 8.28 (1H, s, H-2′), 8.14 (1H, dd, *J* = 8.3, 1.7 Hz), 8.01 (1H, d, *J* = 7.8 Hz), 7.86 (1H, t, *J* = 8.1 Hz), 7.59 (1H, d, *J* = 8.3 Hz), 7.20 (1H, d, *J* = 8.3 Hz), 4.55 (2 H, t, *J* = 7.8 Hz), 4.16 (3 H, s), 3.96 (3 H, s), 3.39 (2 H, t, *J* = 7.8 Hz). ^13^C NMR (125 MHz, CD_3_OD) *δ*: 165.8 (C-1), 154.7, 152.8, 145.3, 135.5, 132.6, 129.8, 128.7, 128.0, 125.6, 123.9, 120.1, 118.2, 115.3, 63.1, 57.0, 53.0, 26.1. ESI-MS *m*/*z*: 293.15 [M–Br]^+^.

#### 7,8-Dimethoxy-2-(2-tolyl)-3,4-dihydroisoquinolin-2-ium bromide (**A20**)

Yield, 59%; yellow solids; mp 147.8–148.7 °C; ^1^H NMR (500 MHz, CD_3_OD) *δ*: 9.36 (1H, s, H-1), 7.62–7.57 (2 H, m), 7.56–7.52 (2 H, m), 7.48 (1H, dt, *J* = 7.2, 2.0 Hz), 7.22 (1H, d, *J* = 8.6 Hz), 4.55 (2 H, t-like, *J* = 7.8 Hz), 4.11 (3 H, s), 3.97 (3 H, s), 3.40 (2 H, t, *J* = 7.8 Hz), 2.46 (3 H, s). ^13^C NMR (125 MHz, CD_3_OD) *δ*: 167.1 (C-1), 154.2, 152.8, 144.3, 133.5, 133.4, 132.3, 129.6, 129.0, 126.1, 125.1, 124.1, 119.8, 63.0, 57.0, 53.8, 26.2, 17.6. ESI-MS *m*/*z*: 282.15 [M–Br]^+^.

#### 7,8-Dimethoxy-2-(3-tolyl)-3,4-dihydroisoquinolin-2-ium bromide (**A21**)

Yield, 45%; yellow solids; mp 111.7–112.7 °C; ^1^H NMR (500 MHz, CD_3_OD) *δ*: 9.32 (1H, s, H-1), 7.63 (1H, s, H-2′), 7.58–7.53 (3 H, m), 7.48 (1H, d, *J* = 7.3 Hz), 7.19 (1H, d, *J* = 8.2 Hz), 4.53 (2 H, t-like, *J* = 7.7 Hz), 4.14 (3 H, s), 3.96 (3 H, s), 3.36 (2 H, t, *J* = 7.8 Hz), 2.50 (3 H, s). ^13^C NMR (125 MHz, CD_3_OD) *δ*: 163.8 (C-1), 154.1, 152.8, 145.0, 142.2, 132.9, 131.2, 129.6, 124.9, 124.2, 123.9, 120.8, 120.1, 63.0, 57.0, 53.0, 26.2, 21.3. ESI-MS *m*/*z*: 282.17 [M–Br]^+^.

#### 7,8-Dimethoxy-2-(4-tolyl)-3,4-dihydroisoquinolin-2-ium bromide (**A22**)

Yield, 69%; yellow solids; mp 148.1–149.1 °C; ^1^H NMR (500 MHz, CD_3_OD) *δ*: 9.30 (1H, s, H-1), 7.68 (2 H, d, *J* = 8.2 Hz), 7.55 (1H, d, *J* = 8.3 Hz), 7.48 (2 H, d, *J* = 8.2 Hz), 7.18 (1H, d, *J* = 8.3 Hz), 4.53 (2 H, t-like, *J* = 7.7 Hz), 4.13 (3 H, s), 3.96 (3 H, s), 3.36 (2 H, t, *J* = 7.7 Hz), 2.47 (3 H, s, Ph-CH_3_). ^13^C NMR (125 MHz, CD_3_OD) *δ*: 163.3 (C-1), 154.0, 152.8, 143.2, 142.6, 131.8, 129.5, 124.7, 123.9, 123.5, 120.2, 63.0, 57.0, 53.0, 26.2, 21.2. ESI-MS *m*/*z*: 282.14 [M–Br]^+^.

#### 7,8-Dimethoxy-2-(2-methoxyphenyl)-3,4-dihydroisoquinolin-2-ium bromide (**A23**)

Yield, 70%; yellow solids; mp 151.2–152.9 °C; ^1^H NMR (500 MHz, CD_3_OD) *δ*: 9.31 (1H, s, H-1), 7.67 (1H, dd, *J* = 7.9, 1.6 Hz), 7.67 (1H, dt-like, *J* = 7.9, 1.6 Hz), 7.57 (1H, d, *J* = 8.3 Hz), 7.34 (1H, d, *J* = 7.8 Hz), 7.19–7.22 (2 H, m), 4.36 (2 H, t-like, *J* = 7.7 Hz), 4.11 (3 H, s), 4.00 (3 H, s), 3.96 (3 H, s), 3.35 (2 H, t, *J* = 7.7 Hz). ^13^C NMR (125 MHz, CD_3_OD) *δ*: 166.8 (C-1), 154.1, 153.8, 152.8, 133.9, 133.4, 129.9, 126.8, 125.0, 124.1, 122.6, 120.0, 114.3, 63.0, 57.1, 57.0, 53.7, 26.3. ESI-MS *m*/*z*: 298.16 [M–Br]^+^.

#### 7,8-Dimethoxy-2-(3-methoxyphenyl)-3,4-dihydroisoquinolin-2-ium bromide (**A24**)

Yield, 70%; yellow solids; mp 132.4–133.9 °C; ^1^H NMR (500 MHz, CD_3_OD) *δ*: 9.33 (1H, s, H-1), 7.58–7.55 (2 H, m), 7.38 (1H, t, *J* = 2.3 Hz), 7.33 (1H, dd, *J* = 7.9, 1.8 Hz), 7.22–7.18 (2 H, m), 4.54 (2 H, t, *J* = 7.8 Hz), 4.14 (3 H, s), 3.96 (3 H, s), 3.92 (3 H, s), 3.35 (2 H, t, *J = *7.8 Hz). ^13^C NMR (125 MHz, CD_3_OD) *δ*: 164.2 (C-1), 162.4, 154.2, 152.8, 146.0, 132.3, 129.7, 125.0, 123.9, 120.1, 117.9, 115.6, 109.7, 63.0, 57.0, 56.5, 53.1, 26.1. ESI-MS *m*/*z*: 298.16 [M–Br]^+^.

#### 7,8-Dimethoxy-2-(4-methoxyphenyl)-3,4-dihydroisoquinolin-2-ium bromide (**A25**)

Yield, 63%; yellow solids; mp 99.5–100.3 °C; ^1^H NMR (500 MHz, *d*_6_-DMSO) *δ*: 9.22 (1H, s, H-1), 7.83 (2 H, d-like, *J* = 9.1 Hz), 7.59 (1H, d, *J* = 8.3 Hz), 7.23 (1H, d, *J* = 8.3 Hz), 7.19 (2 H, d-like, *J* = 9.1 Hz), 4.48 (2 H, t, *J* = 7.7 Hz), 4.01 (3 H, s), 3.91 (3 H, s), 3.87 (3 H, s), 3.29 (2 H, t, *J* = 7.7 Hz). ^13^C NMR (125 MHz, *d*_6_-DMSO) *δ*: 160.8, 160.7, 151.2, 150.9, 136.3, 128.0, 124.4, 122.9, 119.2, 114.8, 62.1, 56.5, 55.8, 51.3, 24.5. ESI-MS *m*/*z*: 298.15 [M–Br]^+^.

#### 2-(2-Hydroxyphenyl)-7,8-dimethoxy-3,4-dihydroisoquinolin-2-ium bromide (**A26**)

Yield, 77%; yellow solid; mp 151.0–151.9 °C; ^1^H NMR (500 MHz, *d*_6_-DMSO) *δ*: 11.01(1H, s, OH), 9.33 (1H, s, H-1), 7.70 (1H, dd, *J* = 8.2, 1.5 Hz), 7.62 (1H, d, *J* = 8.3 Hz), 7.45 (1H, dt, *J* = 7.8, 1.5 Hz), 7.25 (1H, d, *J* = 8.3 Hz), 7.18 (1H, dd, *J* = 8.2, 0.9 Hz), 7.05 (1H, dt, *J* = 7.7, 1.2 Hz), 4.34 (2 H, t, *J* = 7.7 Hz), 3.99 (3 H, s), 3.92 (3 H, s), 3.28 (2 H, t, *J* = 7.7 Hz). ^13^C NMR (125 MHz, *d*_6_-DMSO) *δ*: 164.5 (C-1), 151.4, 151.0, 150.5, 131.9, 130.7, 128.3, 125.9, 123.3, 123.1, 119.7, 118.8, 117.2, 62.2, 56.5, 51.7, 24.6. ESI-MS *m*/*z*: 284.15 [M–Br]^+^.

#### 2-(3-Hydroxyphenyl)-7,8-dimethoxy-3,4-dihydroisoquinolin-2-ium bromide (**A27**)

Yield, 64%; yellow solids; mp 137.6–139.1 °C; ^1^H NMR (500 MHz, *d*_6_-DMSO) *δ*: 10.27(1H, br s, OH), 9.22 (1H, s, H-1), 7.60 (1H, d, *J* = 8.4 Hz), 7.44 (1H, t, *J* = 8.1 Hz), 7.28 (1H, dd, *J* = 8.0, 1.7 Hz), 7.23–7.21 (2 H, m), 7.04 (1H, dd, *J* = 8.2, 1.9 Hz), 4.46 (2 H, t, *J* = 7.8 Hz), 4.01 (3 H, s), 3.90 (3 H, s), 3.27 (2 H, t, *J* = 7.8 Hz). ^13^C NMR (125 MHz, *d*_6_-DMSO) *δ*: 162.0 (C-1), 158.4, 151.5, 151.0, 144.4, 130.8, 128.3, 123.3, 122.9, 119.1, 117.7, 113.2, 109.8, 62.2, 56.5, 51.2, 24.5. ESI-MS *m*/*z*: 284.13 [M–Br]^+^.

#### 2-(4-Hydroxyphenyl)-7,8-dimethoxy-3,4-dihydroisoquinolin-2-ium bromide (**A28**)

Yield, 74%; yellow solid; mp 175.8–176.3 °C; ^1^H NMR (500 MHz, *d*_6_-DMSO) *δ*: 11.33(1H, s, OH), 9.17 (1H, s, H-1), 7.70 (2 H, d-like, *J* = 9.0 Hz), 7.58 (1H, d, *J* = 8.3 Hz), 7.22 (1H, d, *J* = 8.3 Hz), 7.00 (2 H, d-like, *J* = 9.0 Hz), 4.56 (2 H, t, *J* = 7.7 Hz), 4.00 (3 H, s), 3.91 (3 H, s), 3.27 (2 H, t, *J* = 7.7 Hz). ^13^C NMR (125 MHz, *d*_6_-DMSO) *δ*: 159.9, 159.5, 151.1, 150.9, 134.9, 127.9, 124.3, 122.9, 122.7, 119.2, 116.0, 62.1, 56.5, 51.2, 24.5. ESI-MS *m*/*z*: 284.14 [M−Br]^+^.

#### 5-Bromo-7,8-dimethoxy-2-phenyl-3,4-dihydroisoquinolin-2-ium (**B1**)

Yield, 58%; yellow solids; mp 151.8–152.9 °C; ^1^H NMR (500 MHz, CD_3_OD) *δ*: 9.35 (1H, s, H-1), 7.82–7.79 (2 H, m), 7.76 (1H, s, H-6), 7.71–7.68 (3 H, m), 4.60 (2 H, t, *J* = 7.8 Hz), 4.15 (3 H, s), 3.98 (3 H, s), 3.40 (2 H, t, *J* = 7.8 Hz). ^13^C NMR (125 MHz, CD_3_OD) *δ*: 163.6 (C-1), 154.1, 153.4, 144.7, 132.5, 131.4, 128.4, 127.5, 123.8, 121.1, 117.4, 63.2, 57.4, 52.7, 27.0. HR-ESI-MS [M–Br]^+^: Calcd for C_17_H_17_BrNO_2_^+^, 346.0437, found 346.0447.

#### 5-Bromo-2-(2-fluorophenyl)-7,8-dimethoxy-3,4-dihydroisoquinolin-2-ium (**B2**)

Yield, 34%; yellow solids; mp 140.1–141.6 °C; ^1^H NMR (500 MHz, CD_3_OD) *δ*: 9.46 (1H, s, H-1), 7.85 (1H, t-like, *J* = 7.8 Hz), 7.80 (1H, s, H-6), 7.73–7.69 (1H, m), 7.54–7.48 (2 H, m), 4.51 (2 H, t, *J* = 7.7 Hz), 4.14 (3 H, s), 3.99 (3 H, s), 3.40 (2 H, t, *J* = 7.7 Hz). ^13^C NMR (125 MHz, CD_3_OD) *δ*: 167.4 (C-1), 156.5 (d, *J* = 253.1 Hz, C-2′), 154.6, 153.4, 134.6, 132.2, 128.7, 128.1, 127.3, 127.1 (d, *J* = 3.7 Hz), 120.8, 118.6 (d, *J* = 19.4 Hz), 117.5, 63.3, 57.4, 53.5, 27.1. HR-ESI-MS [M–Br]^+^: Calcd for C_17_H_16_BrFNO_2_^+^, 364.0343, found 364.0354.

#### 5-Bromo-2-(3-fluorophenyl)-7,8-dimethoxy-3,4-dihydroisoquinolin-2-ium (**B3**)

Yield, 51%; yellow solid; mp 144.1–145.2 °C; ^1^H NMR (500 MHz, CD_3_OD) *δ*: 9.39 (1H, s, H-1), 7.77 (1H, s, H-6), 7.73–7.65 (3 H, m), 7.44 (1H, t, *J* = 8.2 Hz), 4.57 (2 H, t, *J* = 7.7 Hz), 4.16 (3 H, s), 3.98 (3 H, s), 3.39 (2 H, t, *J* = 7.7 Hz). ^13^C NMR (125 MHz, CD_3_OD) *δ*: 164.5 (C-1), 164.3 (d, *J* = 248.9 Hz, C-3′), 154.3, 153.4, 145.7, 133.2 (d, *J* = 8.9 Hz), 128.5, 127.7, 121.0, 120.0, 119.3 (d, *J* = 21.1Hz), 117.4, 111.9 (d, *J* = 27.4 Hz), 63.2, 57.4, 52.7, 27.0. ESI-MS *m*/*z*: 364.09, 366.08 [M–Br]^+^.

#### 5-Bromo-2-(4-fluorophenyl)-7,8-dimethoxy-3,4-dihydroisoquinolin-2-ium (**B4**)

Yield, 38%; yellow solids; mp 125.1–126.2 °C; ^1^H NMR (500 MHz, CD_3_OD) *δ*: 9.35 (1H, s, H-1), 7.88–7.86 (2 H, m), 7.76 (1H, s, H-6), 7.43 (2 H, t, *J* = 8.6 Hz), 4.56 (2 H, t, *J* = 7.8 Hz), 4.15 (3 H, s), 3.98 (3 H, s), 3.40 (2 H, t, *J* = 7.8 Hz). ^13^C NMR (125 MHz, CD_3_OD) *δ*: 165.2 (d, *J* = 251.5 Hz, C-4′), 163.9 (C-1), 154.1, 153.4, 140.9, 128.3, 127.5, 126.5 (d, *J* = 9.4 Hz, C-2′, 6′), 121.1, 118.3 (d, *J* = 23.9 Hz, C-3′, 5′), 117.4, 63.2, 57.4, 52.9, 27.0. ESI-MS *m*/*z*: 364.07, 366.08 [M–Br]^+^.

#### 5-Bromo-2-(3-chlorophenyl)-7,8-dimethoxy-3,4-dihydroisoquinolin-2-ium (**B5**)

Yield, 64%; yellow solids; mp 149.2–150.0 °C; ^1^H NMR (500 MHz, CD_3_OD) *δ*: 9.40 (1H, s, H-1), 7.94 (1H, s), 7.77–7.76 (2 H, m), 7.68–7.65 (2 H, m), 4.56 (2 H, t, *J* = 7.7 Hz), 4.16 (3 H, s), 3.98 (3 H, s), 3.39 (2 H, t, *J* = 7.7 Hz). ^13^C NMR (125 MHz, CD_3_OD) *δ*: 164.6 (C-1), 154.5, 153.4, 145.6, 136.9, 132.7, 132.5, 128.5, 127.8, 124.4, 122.5, 121.1, 117.4, 63.2, 57.4, 52.7, 27.0. ESI-MS *m*/*z*: 380.04, 382.03 [M–Br]^+^.

#### 5-Bromo-2-(4-chlorophenyl)-7,8-dimethoxy-3,4-dihydroisoquinolin-2-ium (**B6**)

Yield, 60%; yellow solids; mp 145.7–146.8 °C; ^1^H NMR (500 MHz, CD_3_OD) *δ*: 9.37 (1H, s, H-1), 7.82 (2 H, d, *J* = 8.7 Hz), 7.77 (1H, s, H-6), 7.69 (2 H, d, *J* = 8.7 Hz), 4.56 (2 H, t, *J* = 7.8 Hz), 4.15 (3 H, s), 3.98 (3 H, s), 3.39 (2 H, t, *J* = 7.8 Hz). ^13^C NMR (125 MHz, CD_3_OD) *δ*: 164.0 (C-1), 154.2, 153.4, 143.1, 138.3, 131.4, 128.4, 127.7, 125.6, 121.1, 117.4, 63.2, 57.4, 52.7, 26.9. ESI-MS *m*/*z*: 380.05, 382.04 [M–Br]^+^.

#### 5-Bromo-2-(3-bromophenyl)-7,8-dimethoxy-3,4-dihydroisoquinolin-2-ium (**B7**)

Yield, 74%; yellow solids; mp 151.8–153.0 °C; ^1^H NMR (500 MHz, CD_3_OD) *δ*: 9.39 (1H, s, H-1), 8.08 (1H, s, H-2′), 7.85–7.80 (2 H, m), 7.77 (1H, s, H-6), 7.60 (1H, t, *J* = 8.1 Hz), 4.56 (2 H, t, *J* = 7.6 Hz), 4.15 (3 H, s), 3.98 (3 H, s), 3.39 (2 H, t, *J* = 7.6 Hz). ^13^C NMR (125 MHz, CD_3_OD) *δ*: 164.7 (C-1), 154.4, 153.4, 145.6, 135.5, 132.9, 128.5, 127.8, 127.3, 124.4, 123.0, 121.0, 117.4, 63.2, 57.4, 52.7, 27.0. HR-ESI-MS [M–Br]^+^: Calcd for C_17_H_16_Br_2_NO_2_^+^, 423.9542, found 423.9545.

#### 5-Bromo-2-(4-bromophenyl)-7,8-dimethoxy-3,4-dihydroisoquinolin-2-ium (**B8**)

Yield, 59%; yellow solids; mp 154.5–155.7 °C; ^1^H NMR (500 MHz, CD_3_OD) *δ*: 9.38 (1H, s, H-1), 7.85 (2 H, d, *J* = 8.7 Hz), 7.77 (1H, s, H-6), 7.74 (2 H, d, *J* = 8.7 Hz), 4.56 (2 H, t, *J* = 7.8 Hz), 4.15 (3 H, s), 3.98 (3 H, s), 3.39 (2 H, t, *J* = 7.8 Hz). ^13^C NMR (125 MHz, CD_3_OD) *δ*: 164.0 (C-1), 154.2, 153.4, 143.6, 134.5, 128.4, 127.7, 126.3, 125.8, 121.1, 117.4, 63.2, 57.4, 52.6, 26.9. ESI-MS *m*/*z*: 422.03, 424.02, 426.00, 428.01 [M–Br]^+^.

#### 5-Bromo-2-(4-iodophenyl)-7,8-dimethoxy-3,4-dihydroisoquinolin-2-ium (**B9**)

Yield, 64%; yellow solids; mp 186.7–187.8 °C; ^1^H NMR (500 MHz, CD_3_OD) *δ*: 9.37 (1H, s, H-1), 8.04 (2 H, d, *J* = 8.3 Hz), 7.76 (1H, s, H-6), 7.58 (2 H, d, *J* = 8.3 Hz), 4.56 (2 H, t, *J* = 7.7 Hz), 4.15 (3 H, s), 3.98 (3 H, s), 3.38 (2 H, t, *J* = 7.7 Hz). ^13^C NMR (125 MHz, CD_3_OD) *δ*: 163.8 (C-1), 154.3, 153.4, 144.2, 140.6, 128.4, 127.7, 125.5, 121.1, 117.4, 98.1, 63.2, 57.4, 52.5, 26.9. ESI-MS *m*/*z*: 472.00, 474.01 [M–Br]^+^.

#### 5-Bromo-7,8-dimethoxy-2-(2-methoxyphenyl)-3,4-dihydroisoquinolin-2-ium (**B10**)

Yield, 52%; yellow solids; mp 156.2–157.3 °C; ^1^H NMR (500 MHz, CD_3_OD) *δ*: 9.32 (1H, s, H-1), 7.77 (1H, s, H-6), 7.68–7.63 (2 H, m), 7.35 (1H, d, *J* = 8.4 Hz), 7.21 (1H, t, *J* = 7.7 Hz), 4.41 (2 H, t, *J* = 7.7 Hz), 4.12 (3 H, s), 4.01 (3 H, s), 3.98 (3 H, s), 3.38 (2 H, t, *J* = 7.7 Hz). ^13^C NMR (125 MHz, CD_3_OD) *δ*: 166.3 (C-1), 154.0, 153.8, 153.4, 134.1, 133.1, 128.8, 127.6, 126.8, 122.6, 121.0, 117.5, 114.3, 63.2, 57.4, 57.3, 57.1, 53.4, 27.1. ESI-MS *m*/*z*: 376.10, 378.08 [M–Br]^+^.

### Antifungal Assay

According to the mycelium linear growth rate method reported by us^9^, compounds **A** and **B** were screened for antifungal activity *in vitro* against five plant pathogenic fungi.

Briefly, a solution of the tested compound (36 or 18 mmol) in 10 mL sterile water containing 0.6 mL DMSO was fully mixed with 230 mL of 50 °C melted PDA agar to provide a culture medium containing 150 μM or 75 μM tested compound and 0.25% (v/v) DMSO, and then poured into a sterilized Petri dish (ca. 16 mL each plate). No observable effect on the growth of fungi was proved for 0.25% (v/v) DMSO in the culture medium. TBZ, CBD and ASB were used as positive controls and 0.25% DMSO was used as a blank control. SA iodine and CH iodine were used as reference controls. A fungus disc (d = 5 mm) cut from subcultured petri dishes was placed at the center of the just solidified medium in petri dish. The dishes were kept in an incubator at 25 °C for 72 h. Each experiment was carried out in triplicate. The diameters (in mm) of a fungal colony were measured in three different directions, and the growth inhibition rates were calculated according to the method reported previously^9^. Duncan multiple comparison test was performed on the data to evaluate significant difference between the activities of various compounds at the same concentration.

The compounds with higher initial activities were further assayed for EC_50_ values according to the method described above. Based on the screening results, a series of test concentrations of the compound was set and tested for inhibition rate against the fungi. Antifungal toxicity regression equations were established according to the method previously reported^12^. EC_50_ values were calculated from the equations by using PRISM software ver. 5.0 (GraphPad Software Inc., San Diego, CA, USA).

## Electronic supplementary material

Supplementary information

## References

[1] Savary S, Teng PS, Willocquet L, Nutter FW (2006). Quantification and modeling of crop losses: a review of purposes. Annu. Rev. Phytopathol..

[2] Bräse S, Encinas A, Keck J, Nising CF (2009). Chemistry and Biology of Mycotoxins and Related Fungal Metabolites. Chem. Rev..

[3] Delp CJ (1980). Coping with resistance to plant disease control agents. Plant Dis..

[4] Wedge, D. E. & Camper, N. D. *Biologically active natural products in agrochemicals and pharmaceuticals*; Cutler, H. G., Cutler, S. J., Eds.; CRC Press: Boca Raton, FL, USA; pp 1–15 (2000).

[5] Cao FJ (2015). Pseudocyanides of sanguinarine and chelerythrine and their series of structurally simple analogues as new anticancer lead compounds: Cytotoxic activity, structure–activity relationship and apoptosis induction. Eur. J. Pharm. Sci..

[6] Yang XJ (2012). *In vitro* antifungal activity of sanguinarine and chelerythrine derivatives against phytopathogenic fungi. Molecules..

[7] Miao F (2012). Structural modification of sanguinarine and chelerythrine and their *in Vitro* acaricidal activity against *Psoroptes cuniculi*. Chem. Pharm. Bull..

[8] Miao F (2011). Structural modification of sanguinarine and chelerythrine and their antibacterial activity. Nat. Prod. Res..

[9] Yang R (2015). New Class of 2‑Aryl-6-chloro-3,4-dihydroisoquinolinium Salts as Potential Antifungal Agents for Plant Protection: Synthesis, Bioactivity and Structure–Activity Relationships. J. Agric. Food Chem..

[10] Hou Z (2016). Design, Synthesis, and Structure-Activity Relationship of New 2-Aryl-3,4-dihydro-β-carbolin-2-ium Salts as Antifungal Agents. J. Agric. Food Chem..

[11] Zhu LF (2016). Synthesis, bioactivity and structure–activity relationships of new 2-aryl-8-OR-3,4-dihydroisoquinolin-2-iums salts as potential antifungal agents. Bioorg. Med. Chem. Lett..

[12] Hou Z (2013). 2-(Substituted phenyl)-3,4-dihydroisoquinolin-2-iums as Novel Antifungal Lead Compounds: Biological Evaluation and Structure-Activity Relationships. Molecules..

[13] Yang XJ (2013). Synthesis and *in vitro* antifungal activities of new 2-aryl-6,7-methylenedioxy-3,4-dihydroisoquinolin-2-ium bromides. Chem. Pharm. Bull..

[14] Ma YN (2013). Synthesis of 2-aryl-3,4-dihydroisoquinolin-2-ium bromides and their *in vitro* acaricidal activity against *Psoroptes cuniculi*. Chem. Pharm. Bull..

[15] Yang R (2014). A class of promising acaricidal tetrahydroisoquinoline derivatives: synthesis, biological evaluation and structure-activity relationships. Molecules.

[16] Cao FJ (2017). Cytotoxic activity, apoptosis induction and structure–activity relationship of 8-OR-2-aryl-3,4-dihydroisoquinolin-2-ium salts as promising anticancer agents. Bioorg. Med. Chem. Lett..

[17] Zhang, B. Y. *et al*. Effects of 2-aryl-3,4-dihydroisoquinolin-2-iums on seed germination and seedling growth. *J. Northwest A&F University (Nat. Sci. Ed.)*. **42**, 169–174 (2014).

[18] Zheng, Z. L., Miao, F., Yang, X. J. & Zhou, L. Effects of 2-aryl-3,4-dihydroisoquinolin-2-iums as antifungal agents on plant seed germination and seedling growth. *J. Northwest A&F University (Nat. Sci. Ed.)*. **43**, 116–122 (2015).

[19] Yamato M, Hashigaki K, Qais N, Ishikawa S (1990). Asymmetric synthesis of 1-alkyltetrahydroisoquinolines using chiral oxazolo[2,3-a]tetrahydroisoquinolines. Tetrahedron.

[20] Gao L (2011). Berberine and itraconazole are not synergistic *in vitro* against Aspergillus fumigatus isolated from clinical patients. Molecules.

[21] Jachak GR (2015). Silicon incorporated morpholine antifungals: design, synthesis, and biological evaluation. ACS Med. Chem. Lett..

[22] Psotova J (2006). Safety assessment of sanguiritrin, alkaloid fraction of Macleaya cordata, in rats. Vet. Med..

[23] Kosina P (2004). Sanguinarine and chelerythrine: assessment of safety on pigs in ninety days feeding experiment. Food Chem. Toxicol..

[24] Tenenbaum H, Dahan. N, Soell M (1991). Effectiveness of a sanguinarine regimen after scaling and root planing. J. Periodontol..

[25] Martindale. The Extra Pharmacopeia (Edition 31) 1410 (The Pharmaceutical Press, London, 1996).

[26] Kazushige, Y. Topical preparations for treatment of dermatomycosis. Jpn. Kokai Tokkyo Koho, JP 10175879A, 3 (1998).

[27] Liu L, Pan M, Zhou L (2013). Extraction and isolation of five kinds of alkaloids from *Macleaya microcarpa*. Acta Agriculture Boreali-occidentalis Sinica..

