# Supplementary material for: New 2-aryl-7,8-dimethoxy-3,4-dihydroisoquinolin-2-ium salts as potential antifungal agents: synthesis, bioactivity and structure-activity relationships
Source: Sci Rep. 2017 Aug 8;7:7537. doi: 10.1038/s41598-017-07303-8 (PMC5548800; doi:10.1038/s41598-017-07303-8)
Supplement: Supplementary file 1 — Supplementary information [file 41598_2017_7303_MOESM1_ESM.pdf]

Supplementary information

New 2-aryl-7,8-dimethoxy-3,4-dihydroisoquinolin-2-ium salts as potential antifungal agents: synthesis, bioactivity and structure-activity relationships

Lifei Zhu[†,§], Bohang Zhou[†,§], Bingyu Zhang[†], Mingxuan Xu[†], Huiling Geng[*,†] and Le Zhou[*,†]

[†]College of Chemistry & Pharmacy, Northwest A&F University, Yangling 712100, Shaanxi Province, People's Republic of China

*Corresponding author (Tel: +86-29-87092226; Fax: +86-29-87092226; E-mail: zhoulechem@nwsuaf.edu.cn (L. Zhou); genghuiling5@163.com (H.-L. Geng))

[§]These authors contributed equally to this work.

Contents

^{1}H NMR and ^{13}C NMR spectra of compounds **1–8** 3

^{1}H NMR and ^{13}C NMR spectra of compounds **A** 9

^{1}H NMR and ^{13}C NMR spectra of compounds **B** 37

ESI-MS of compounds **A** and **B** 47

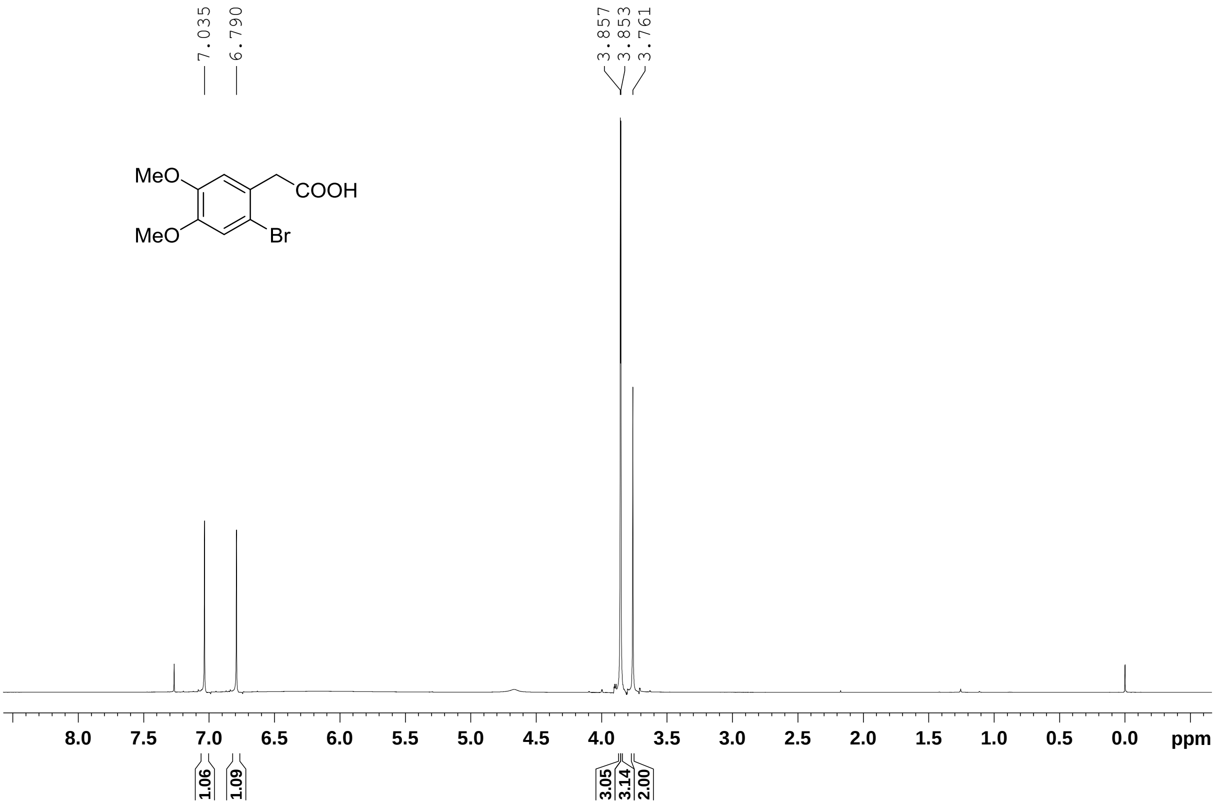

^{1}H NMR of compound **1**

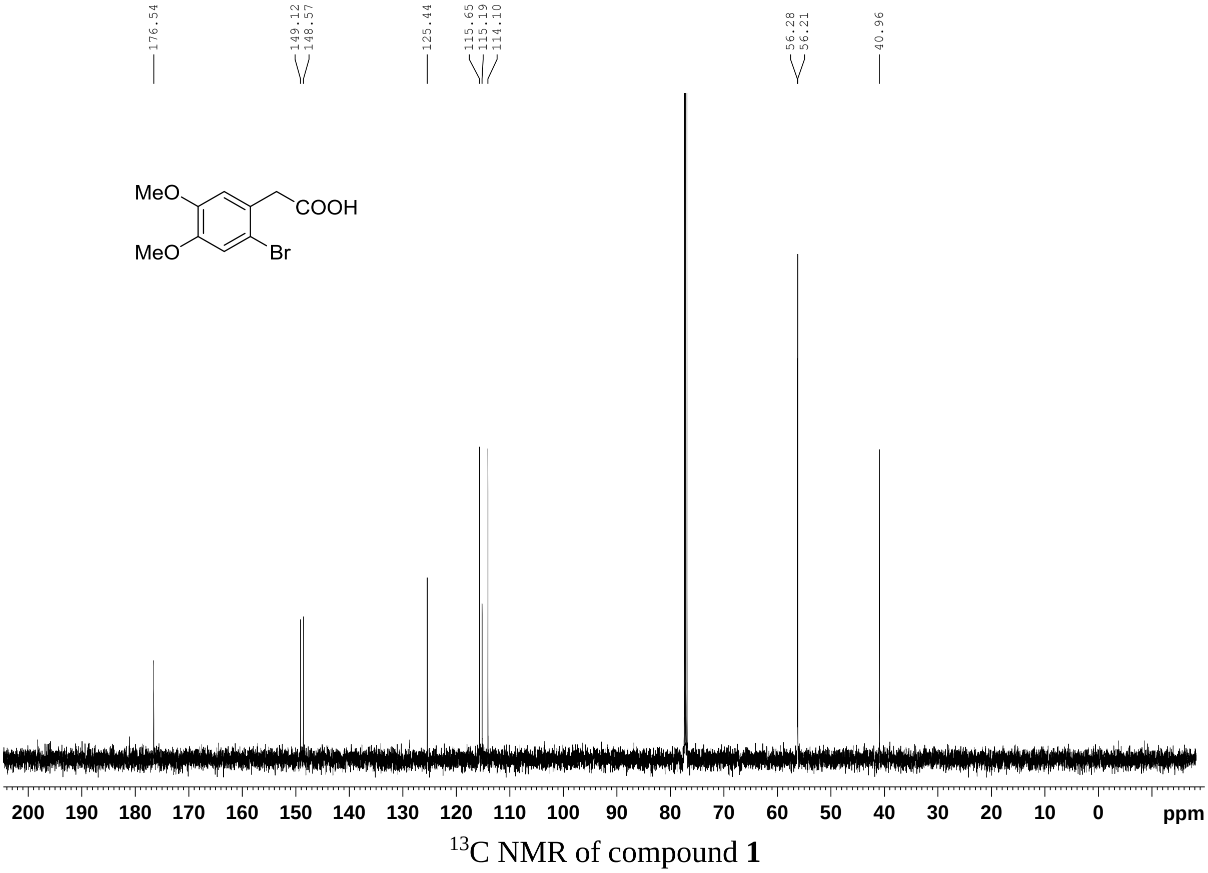

^{13}C NMR of compound **1**

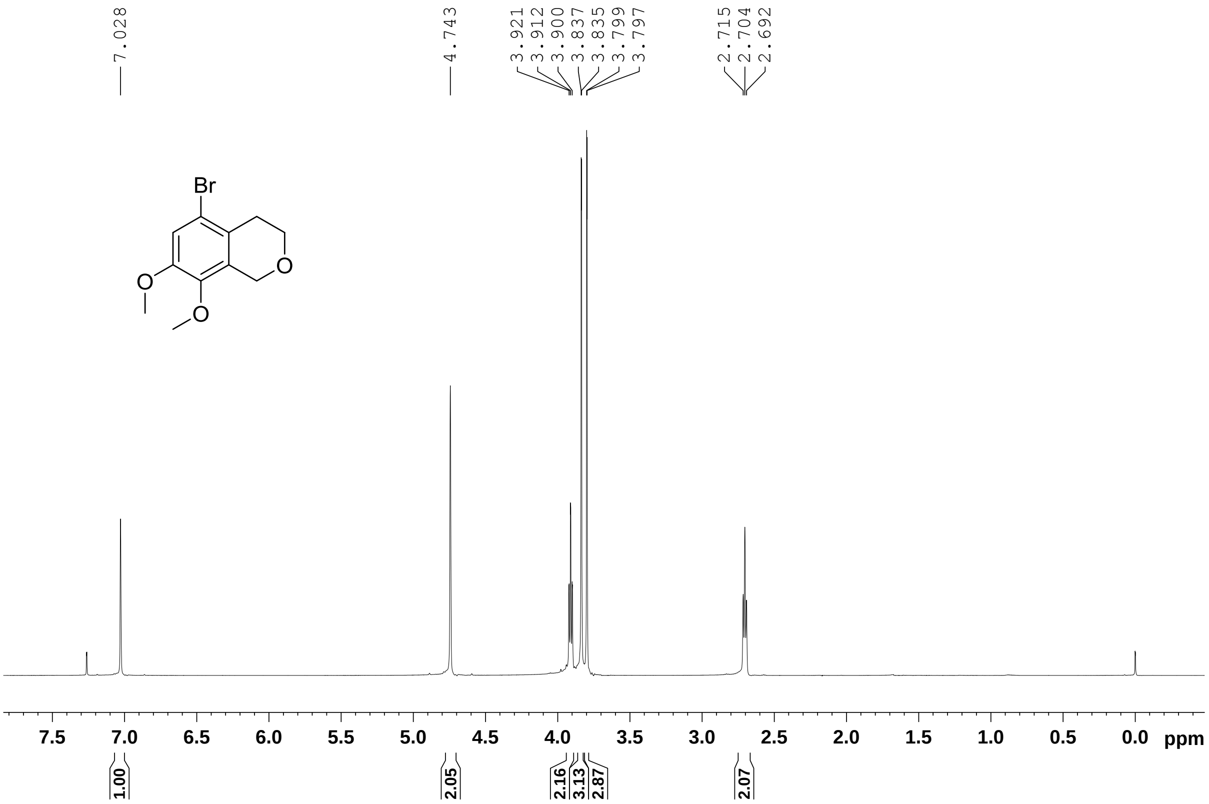

^{1}H NMR of compound **3**

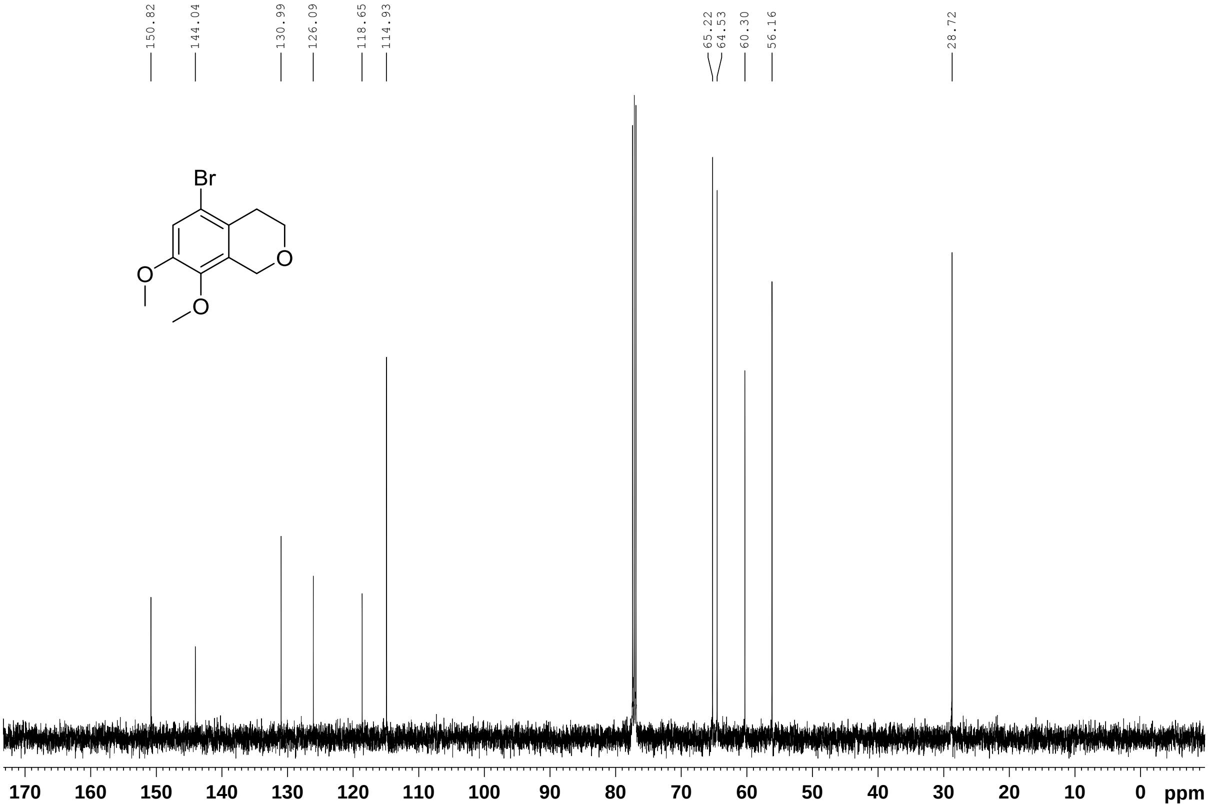

^{13}C NMR of compound **3**

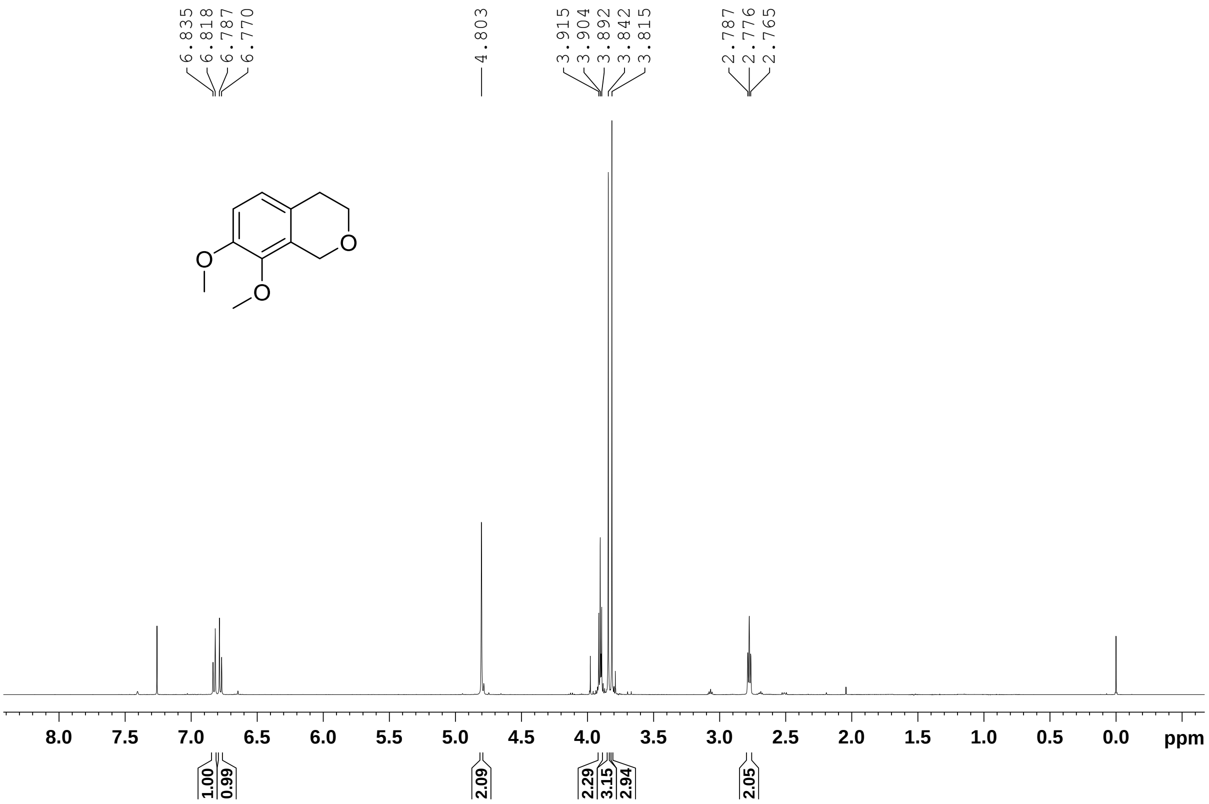

^{1}H NMR of compound **4**

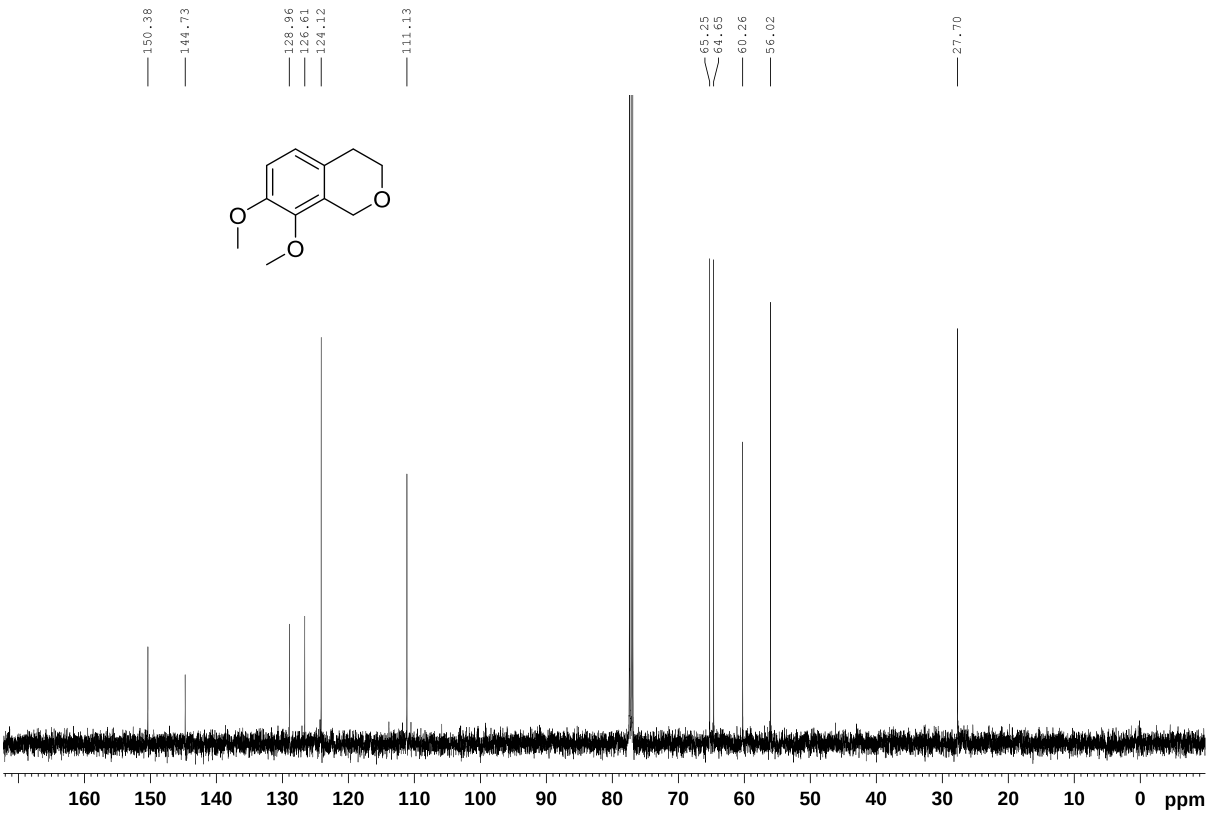

^{13}C NMR of compound **4**

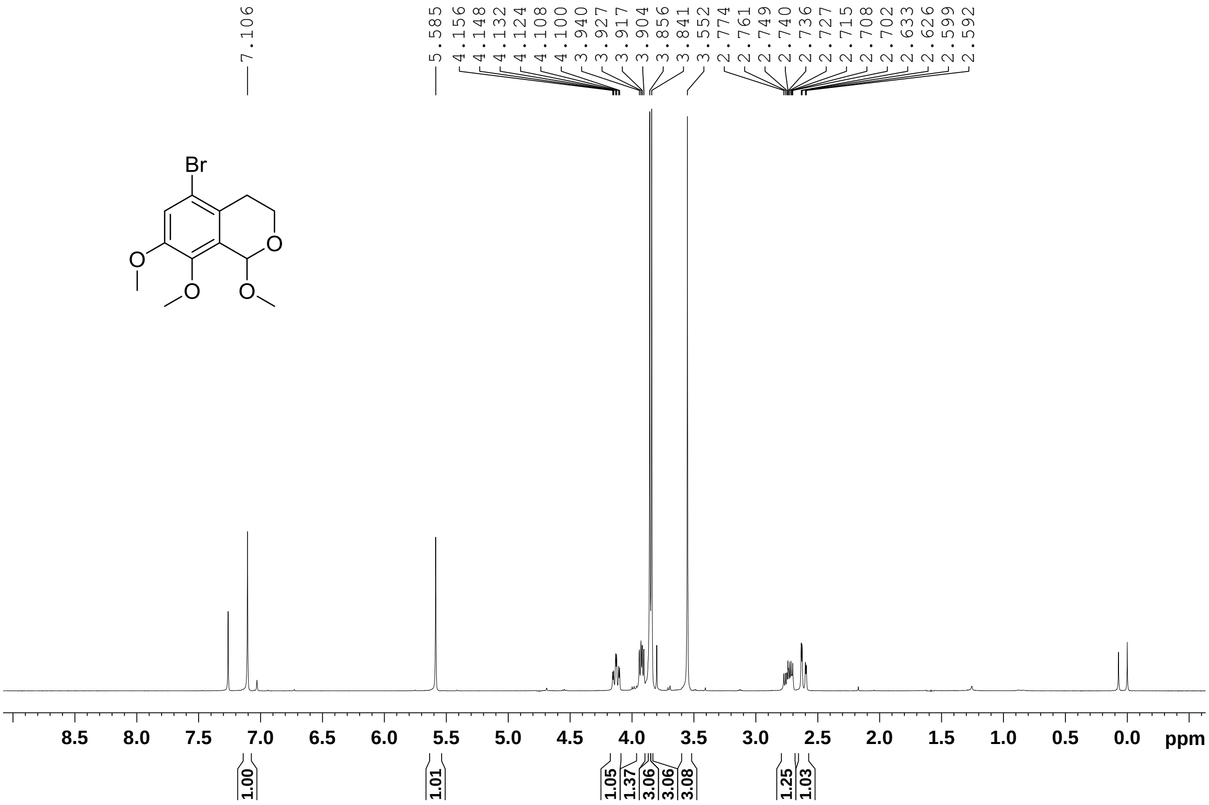

^{1}H NMR of compound **6**

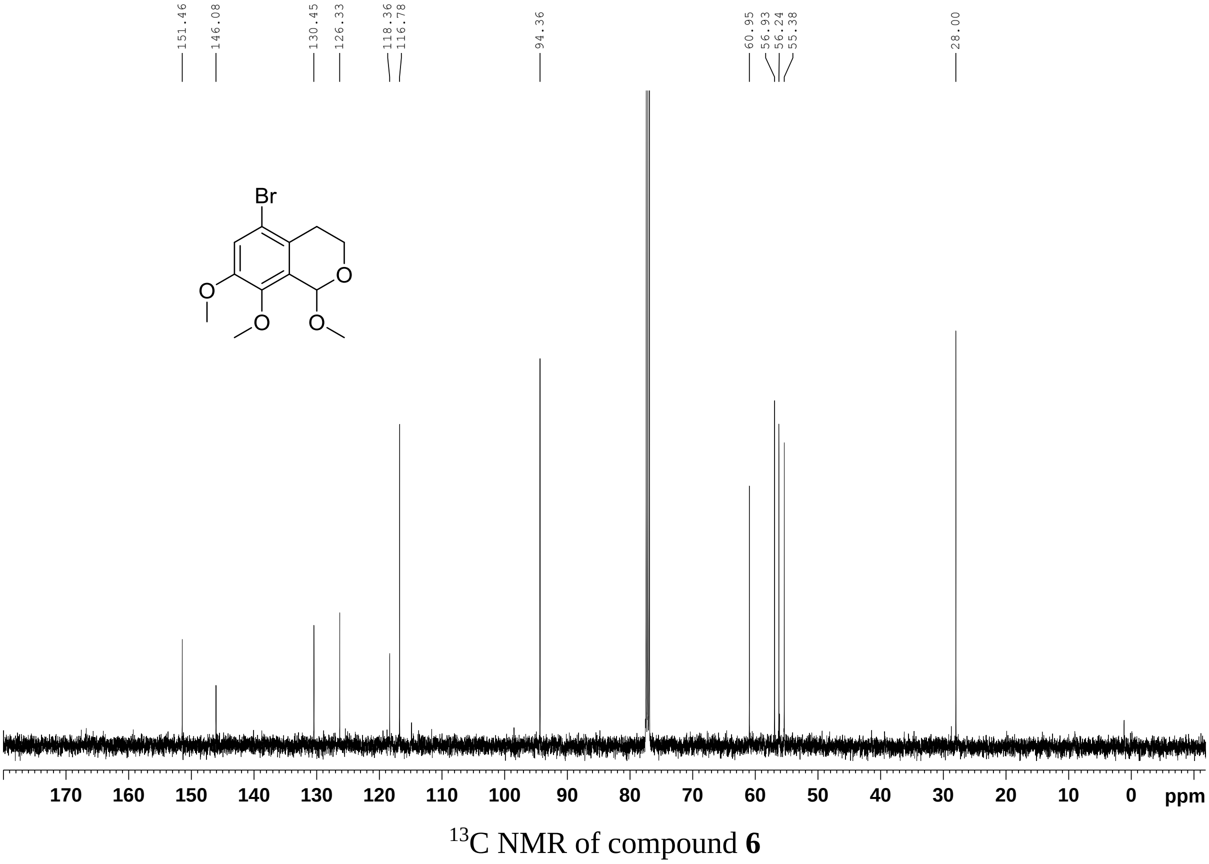

^{13}C NMR of compound **6**

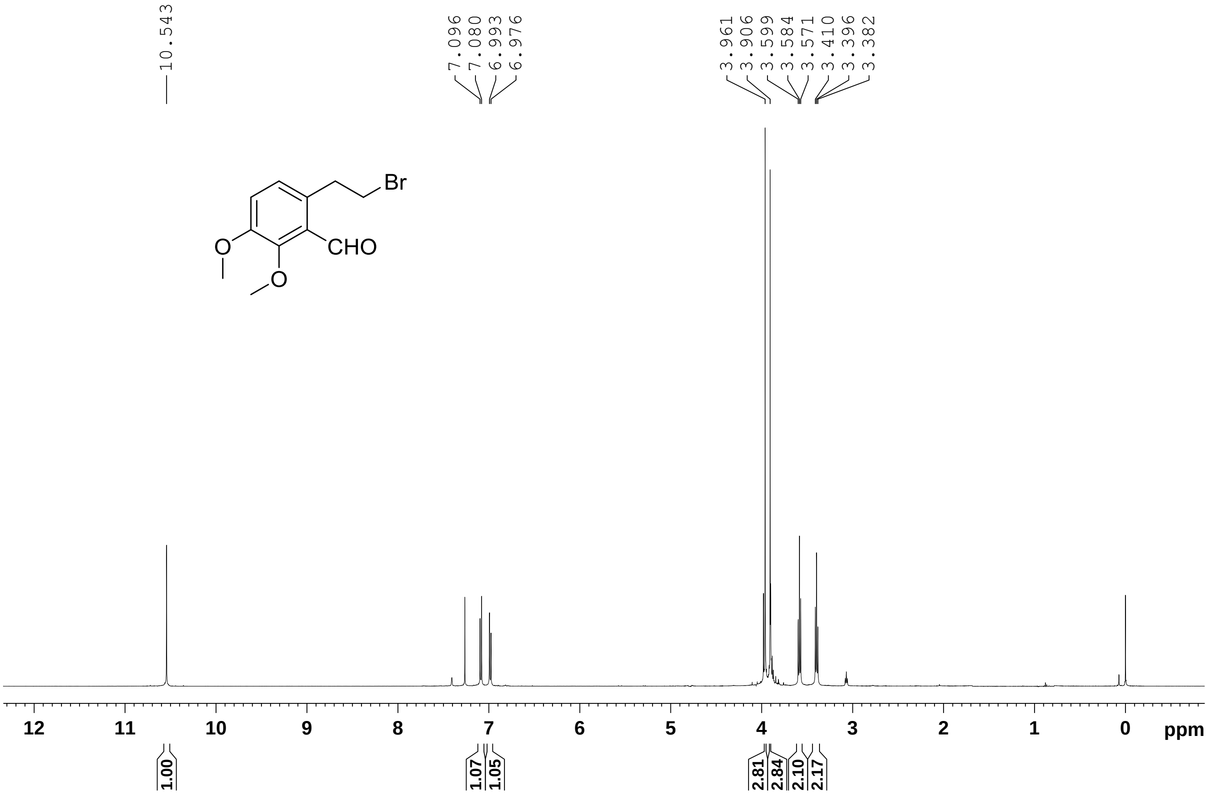

^{1}H NMR of compound **7**

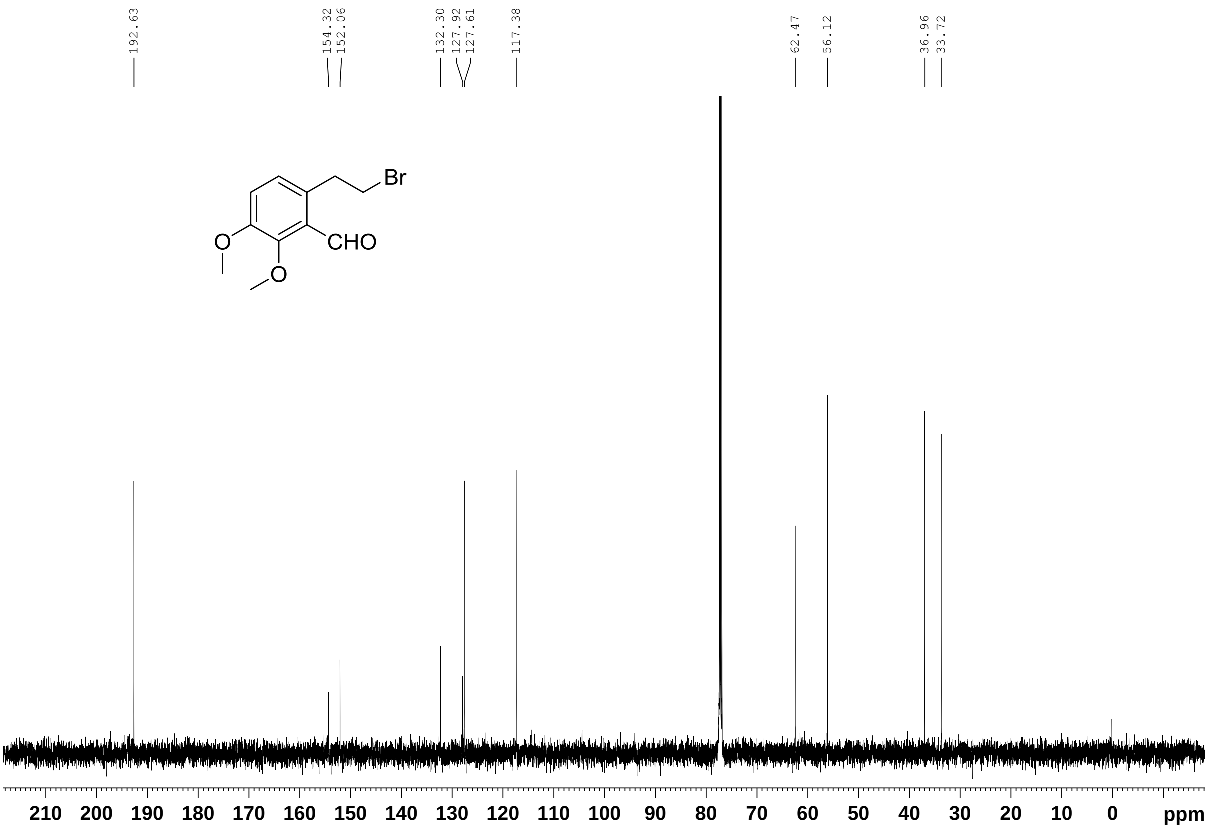

^{13}C NMR of compound **7**

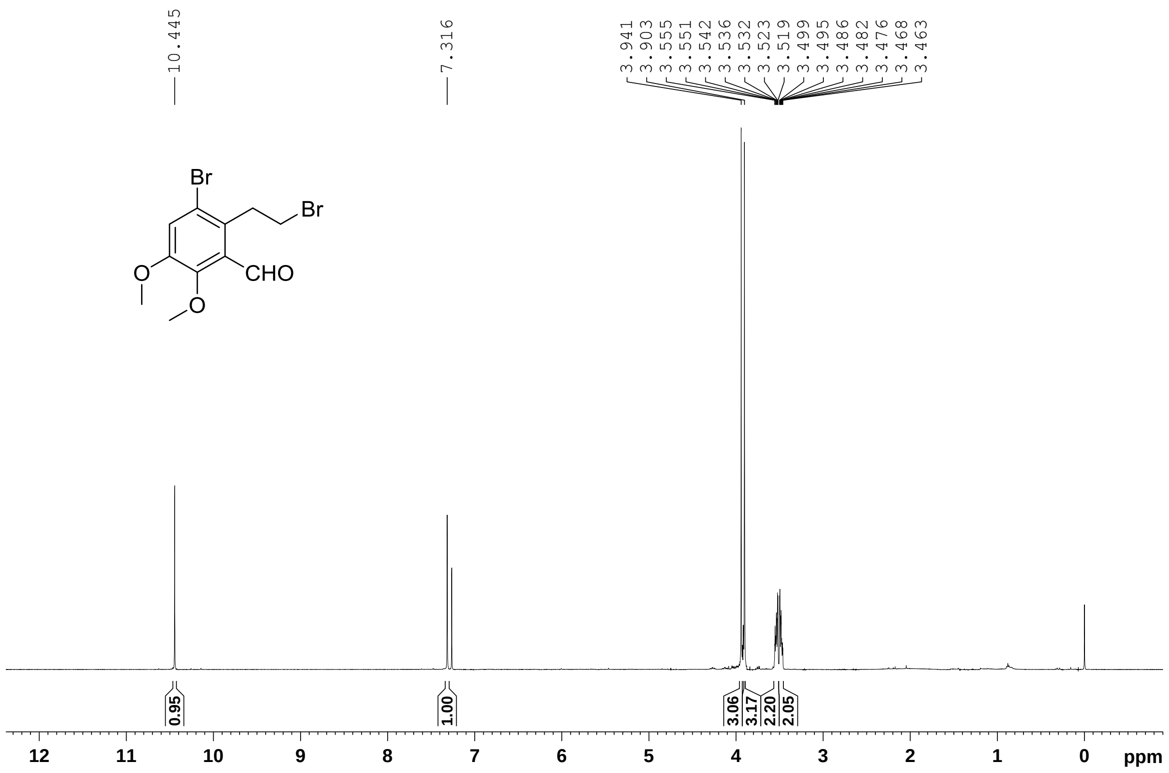

^{1}H NMR of compound **8**

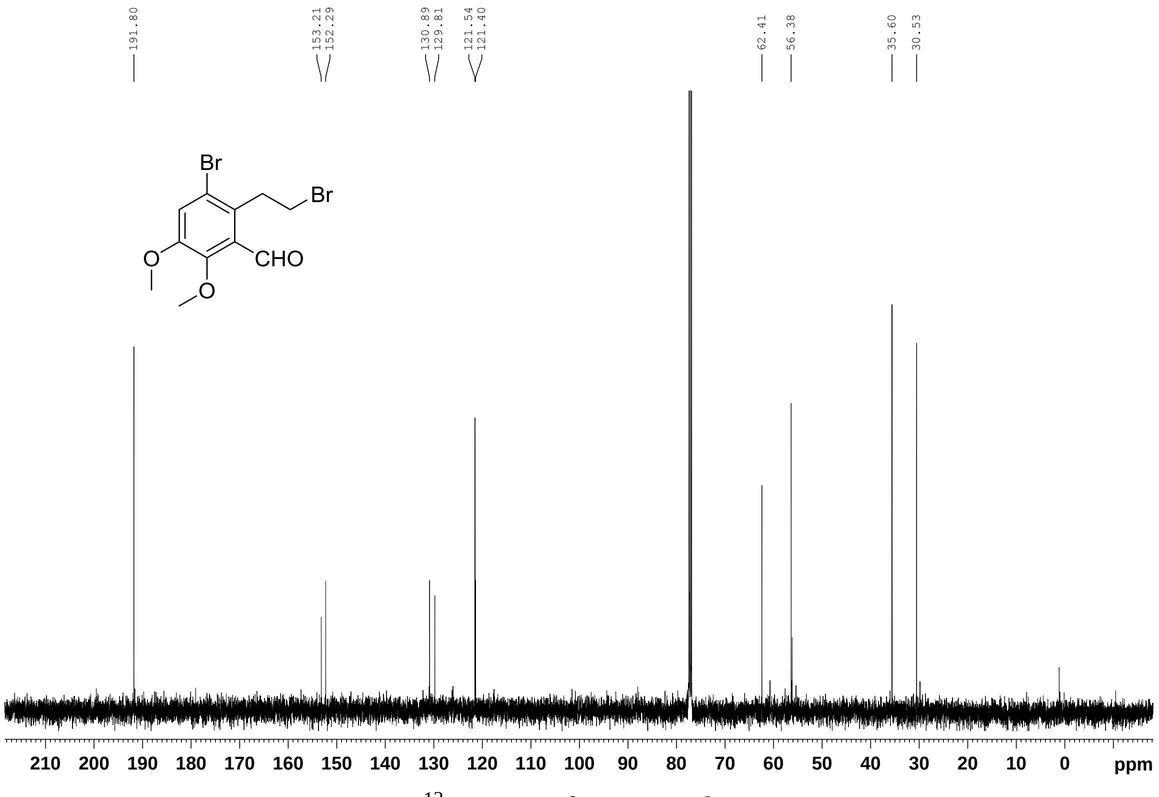

^{13}C NMR of compound **8**

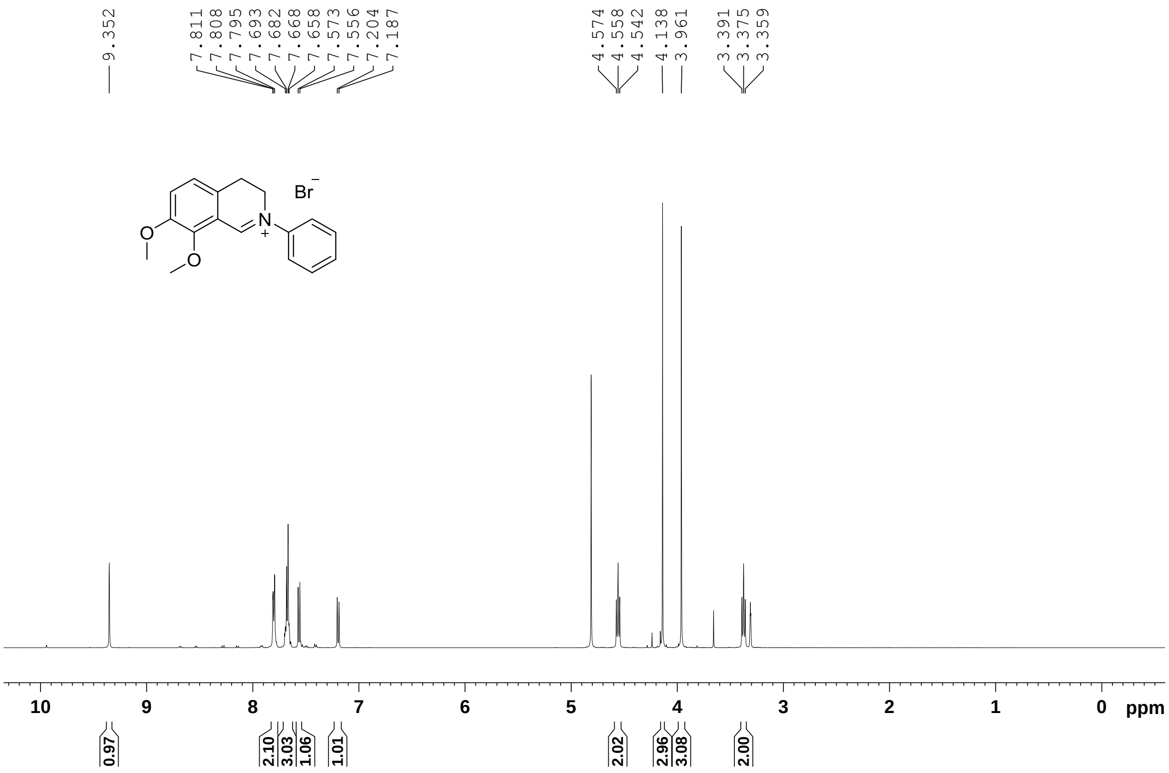

^{1}H NMR of compound **A1**

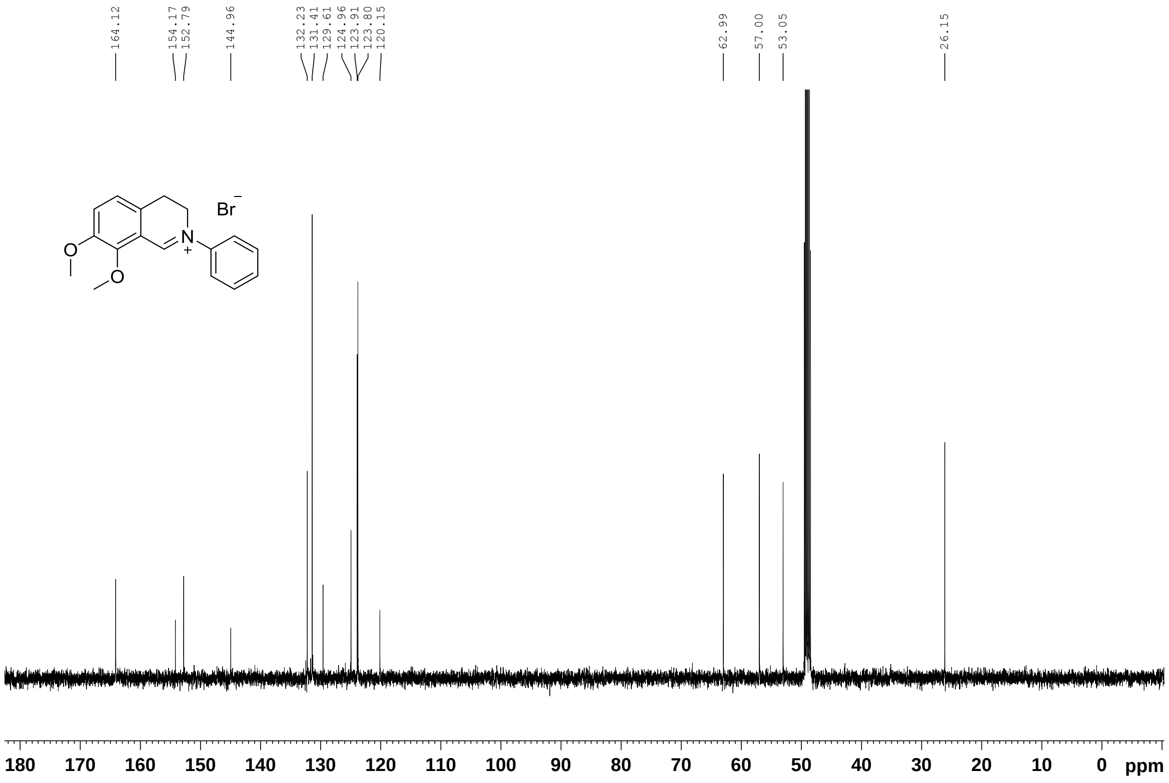

^{13}C NMR of compound **A1**

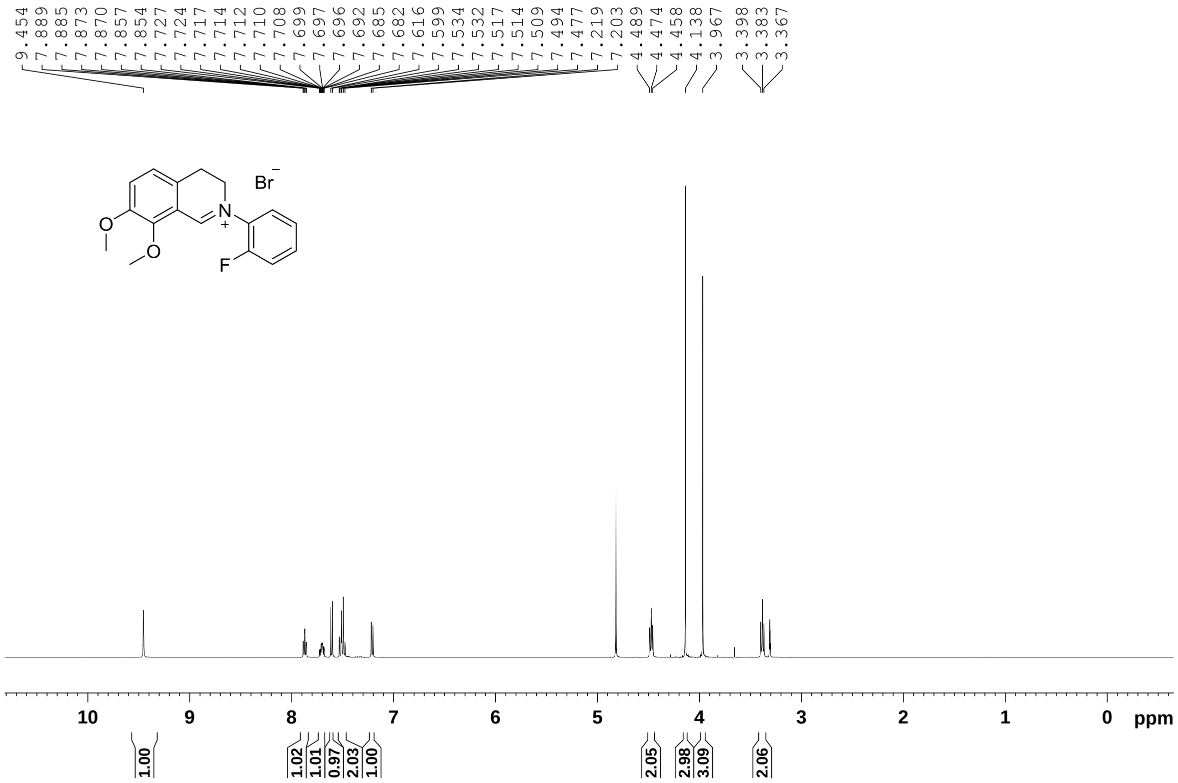

^{1}H NMR of compound **A2**

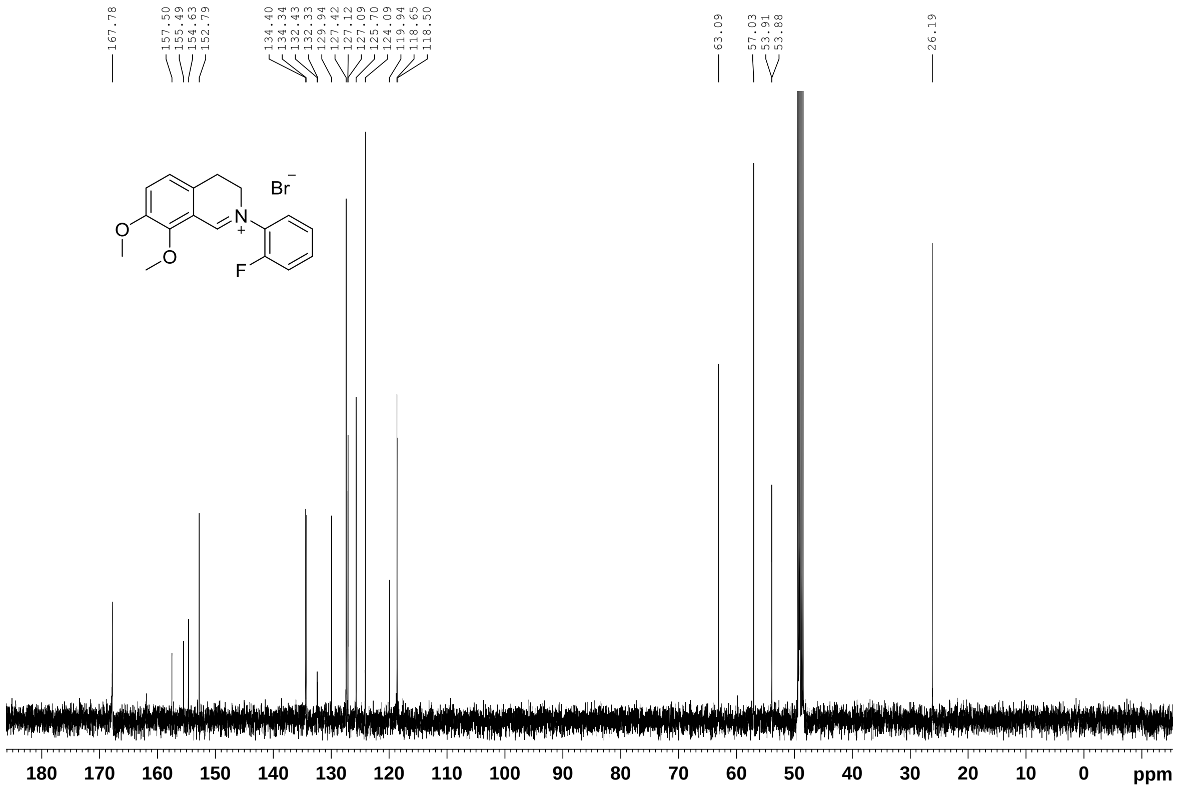

^{13}C NMR of compound **A2**

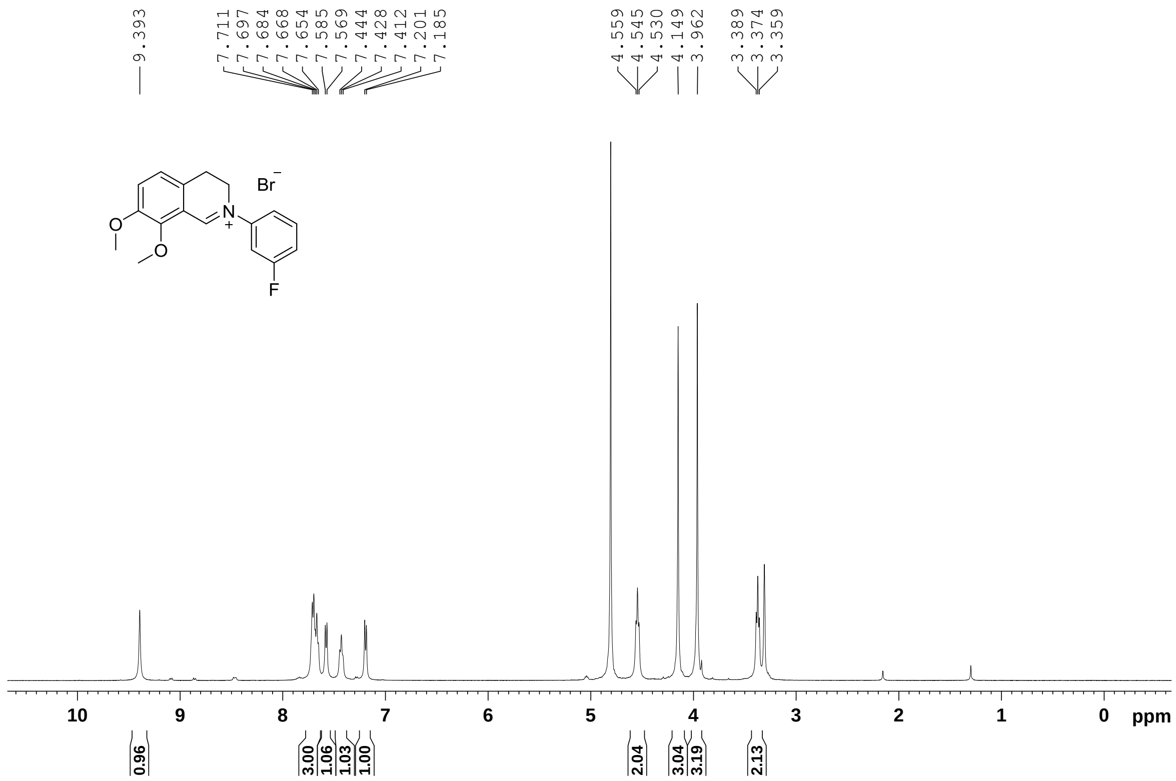

^{1}H NMR of compound **A3**

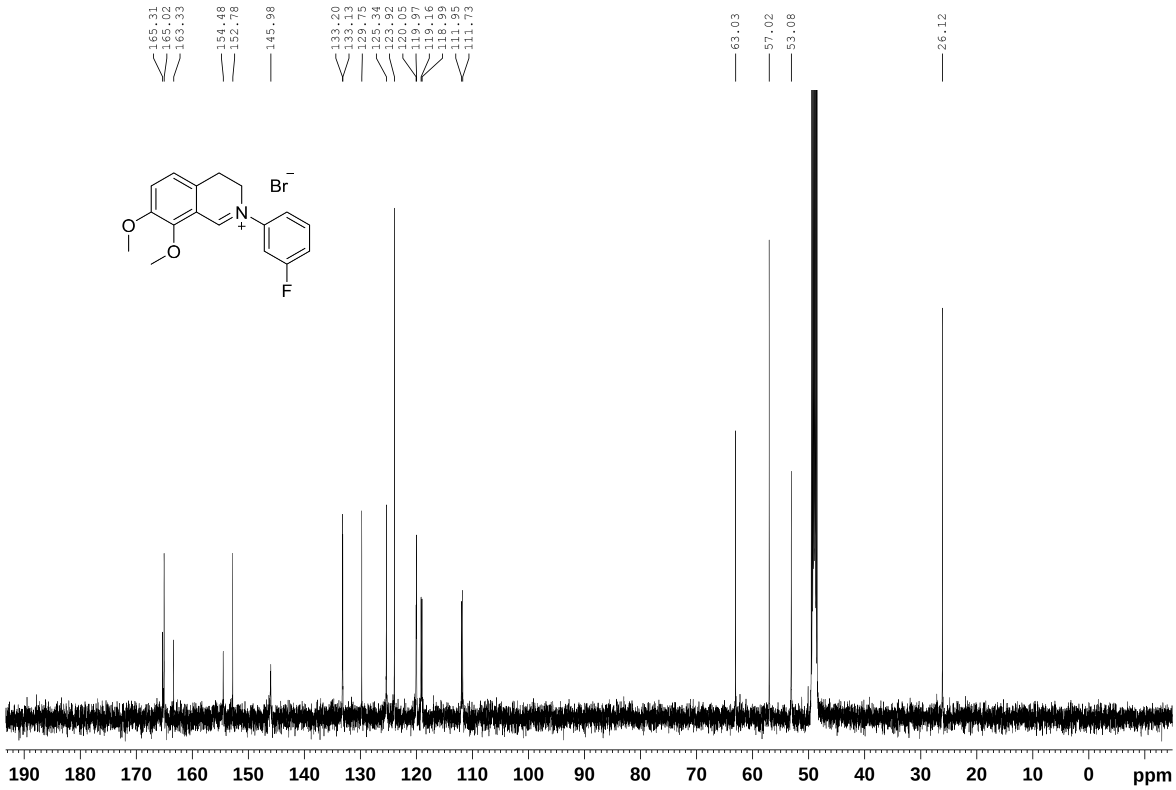

^{13}C NMR of compound **A3**

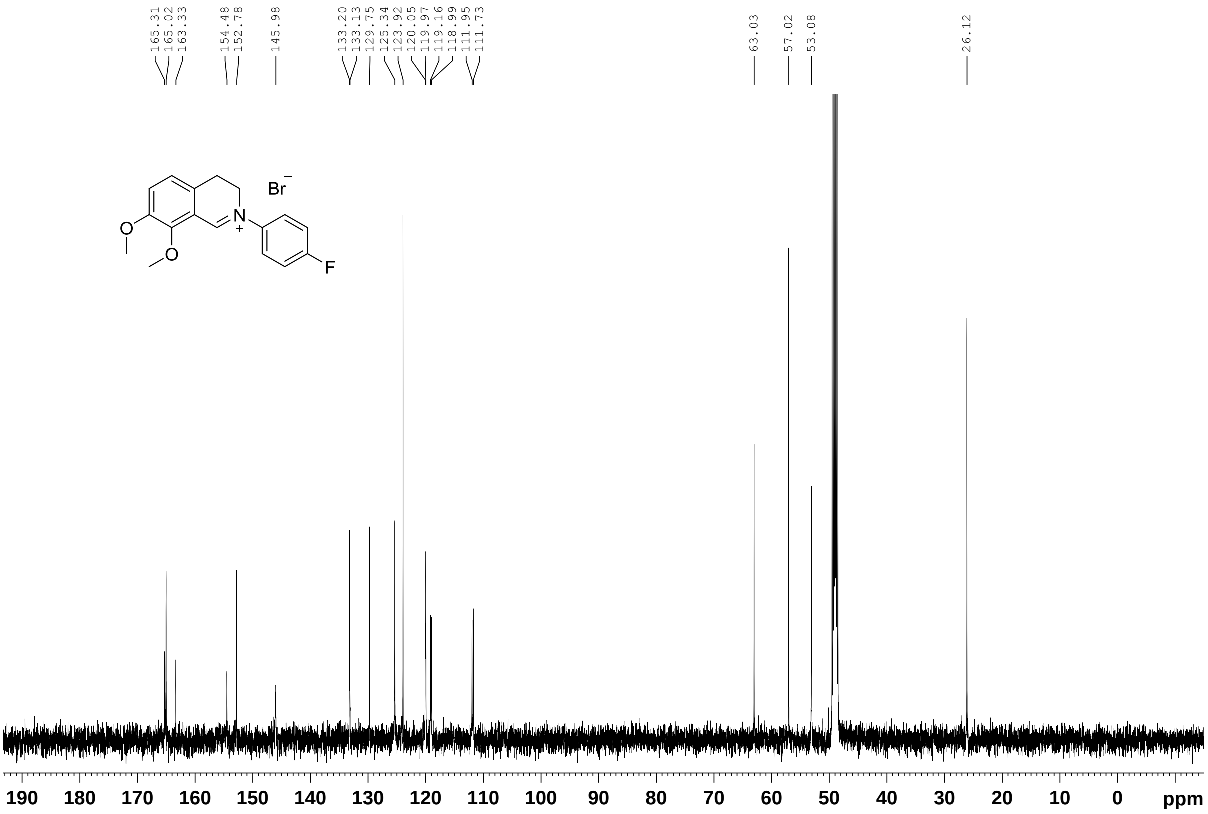

^{1}H NMR of compound **A4**

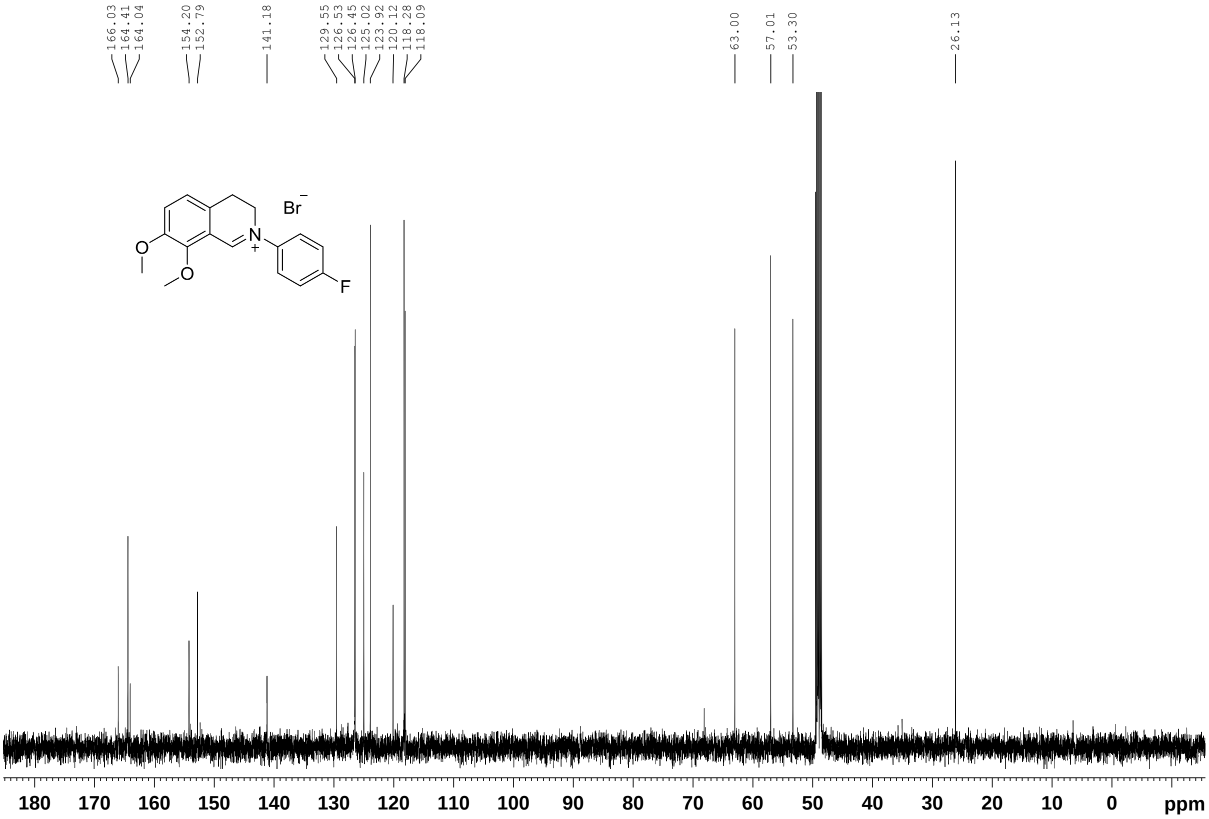

^{13}C NMR of compound **A4**

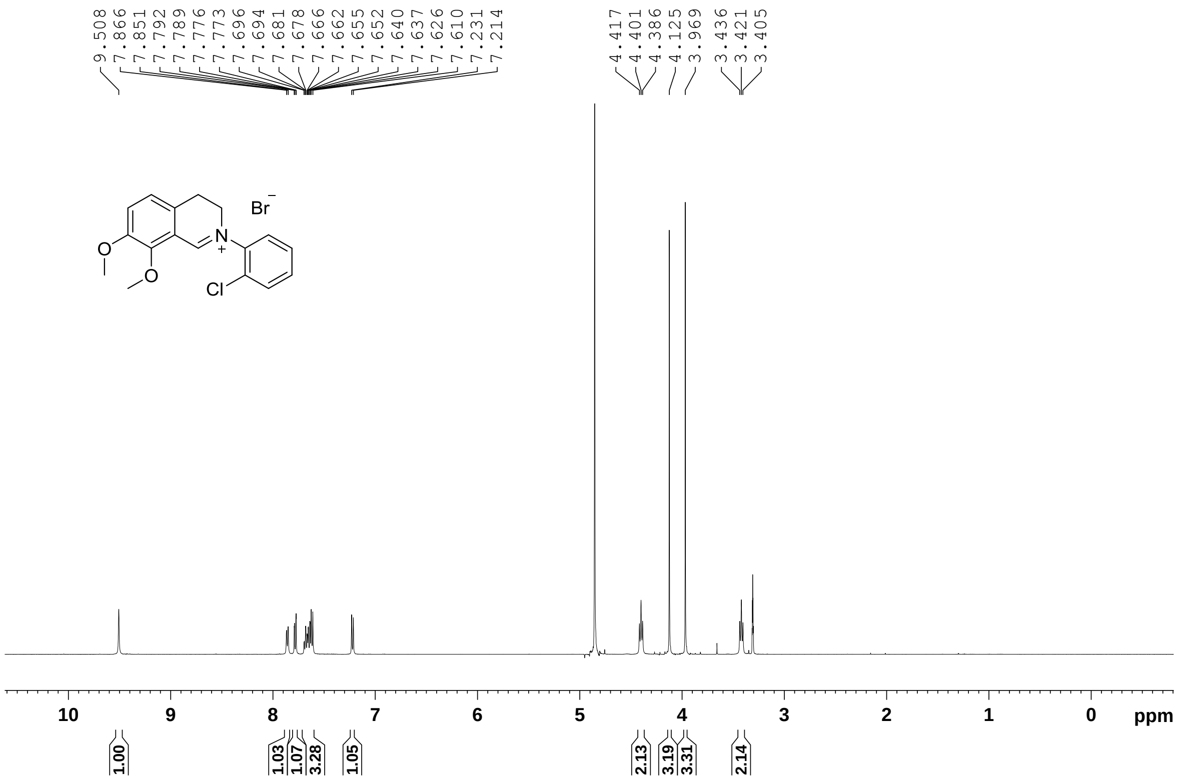

^{1}H NMR of compound **A5**

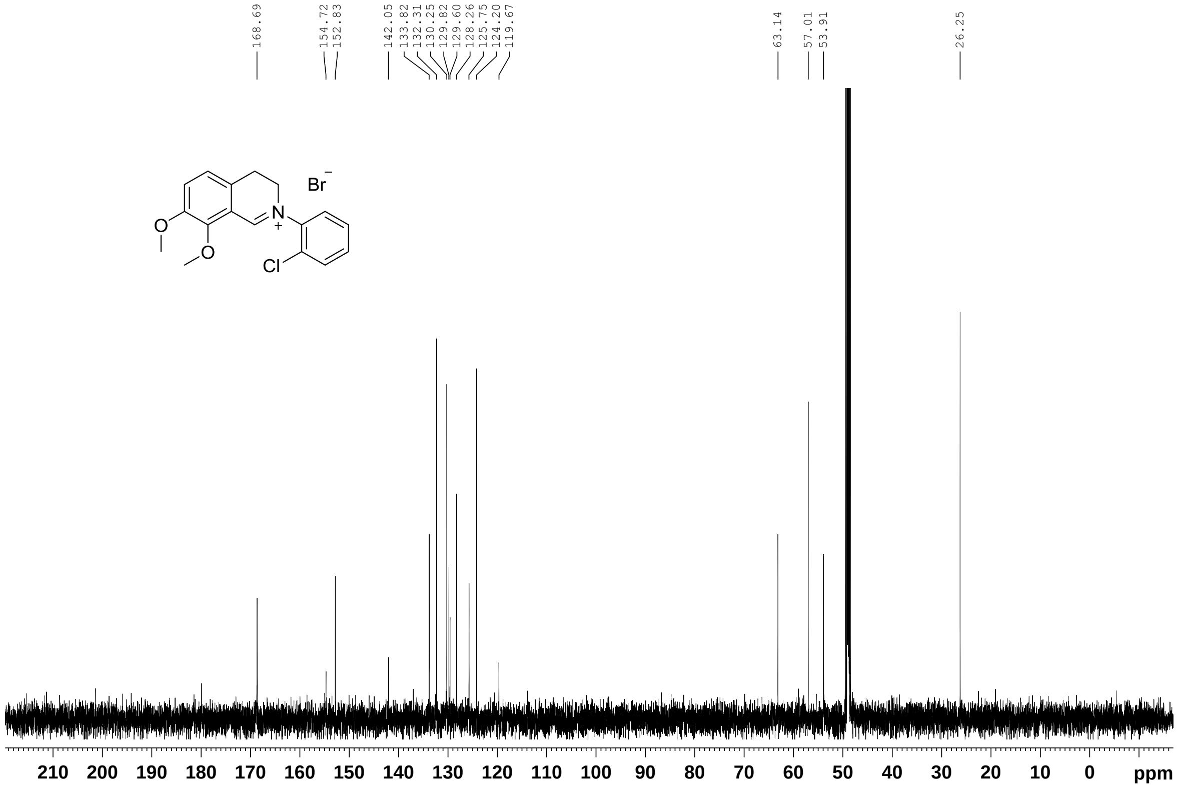

^{13}C NMR of compound **A5**

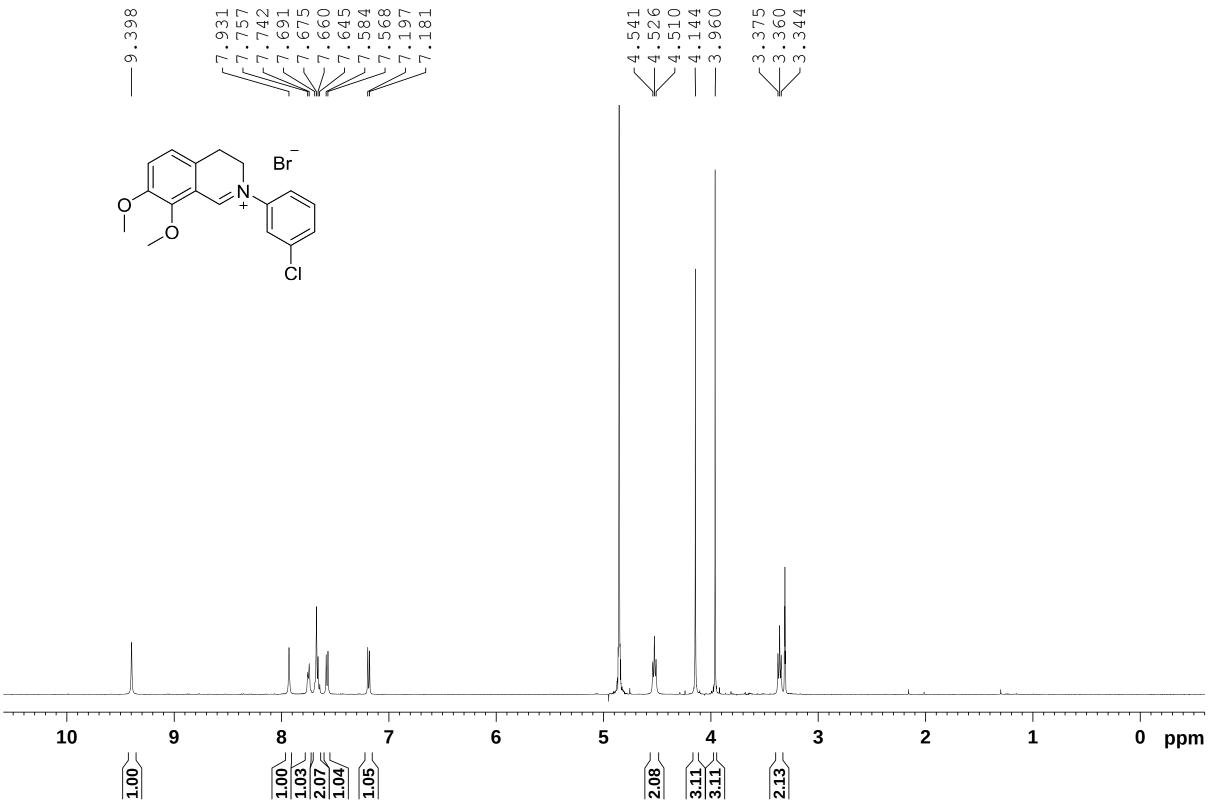

^{1}H NMR of compound **A6**

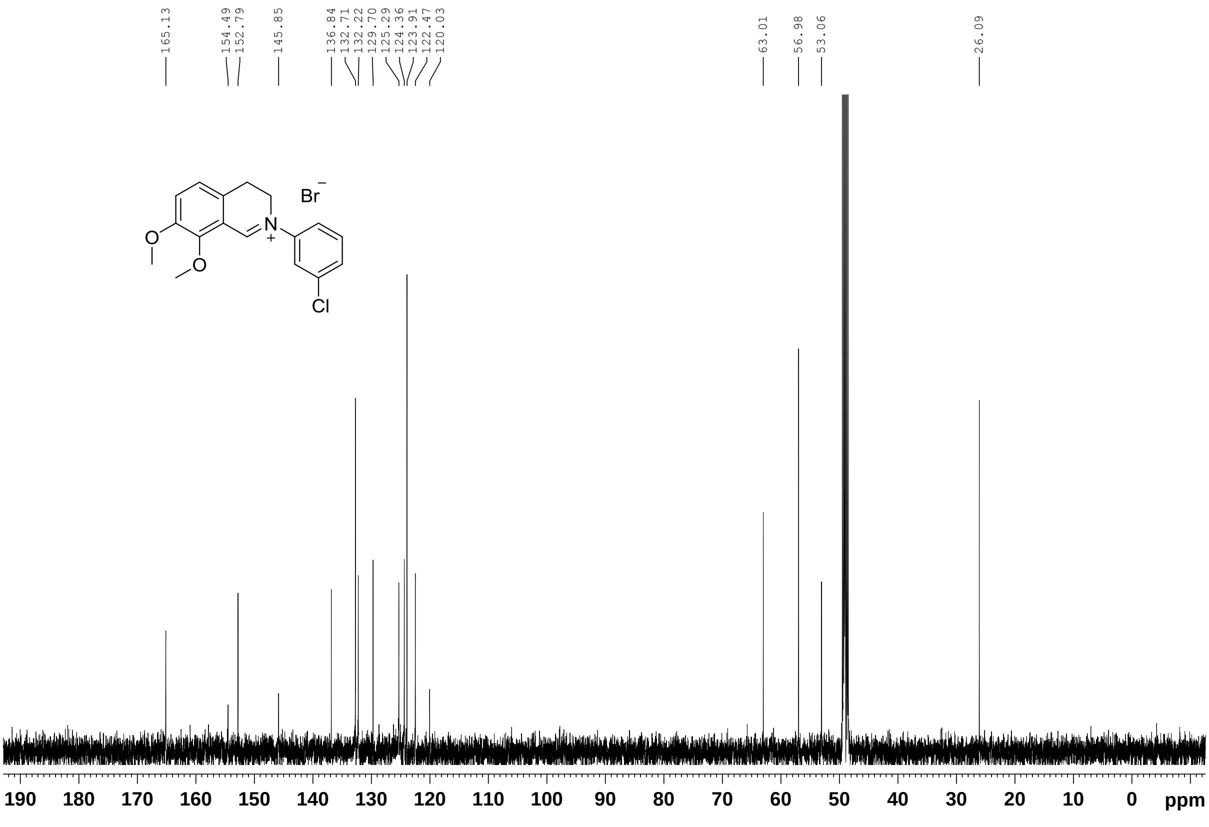

^{13}C NMR of compound **A6**

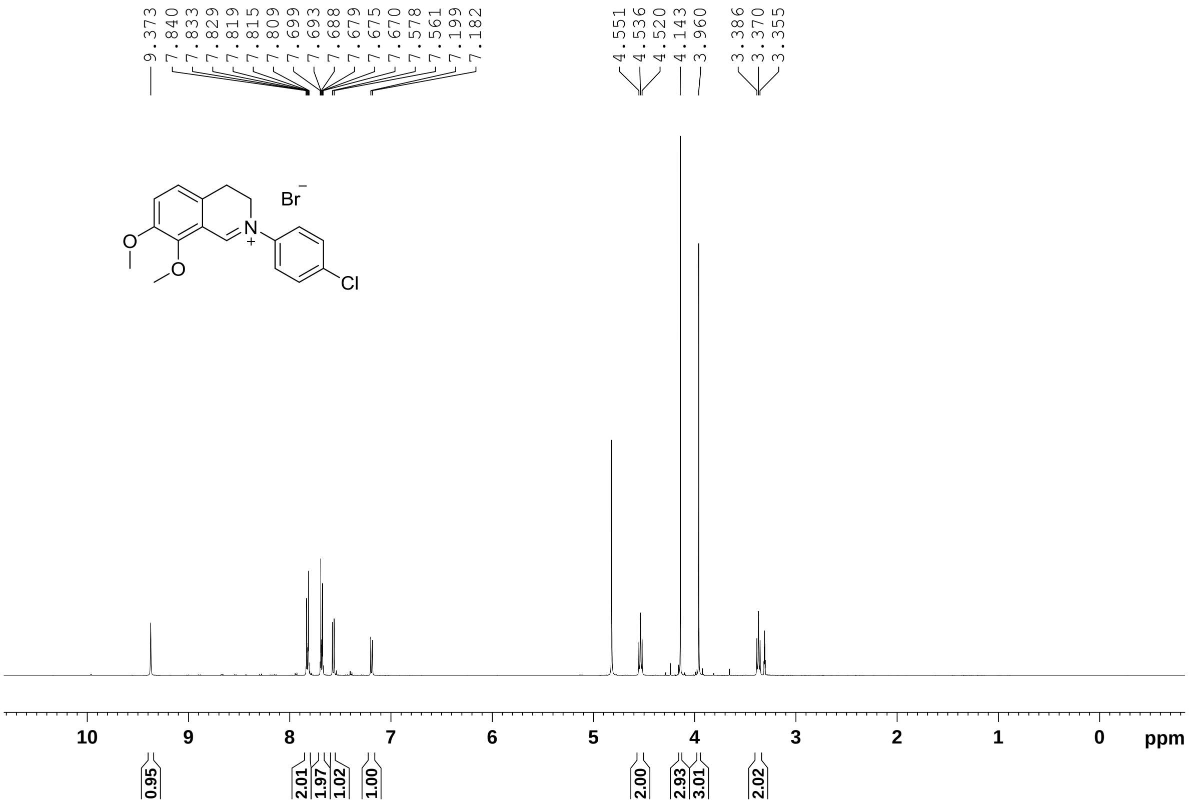

^{1}H NMR of compound **A7**

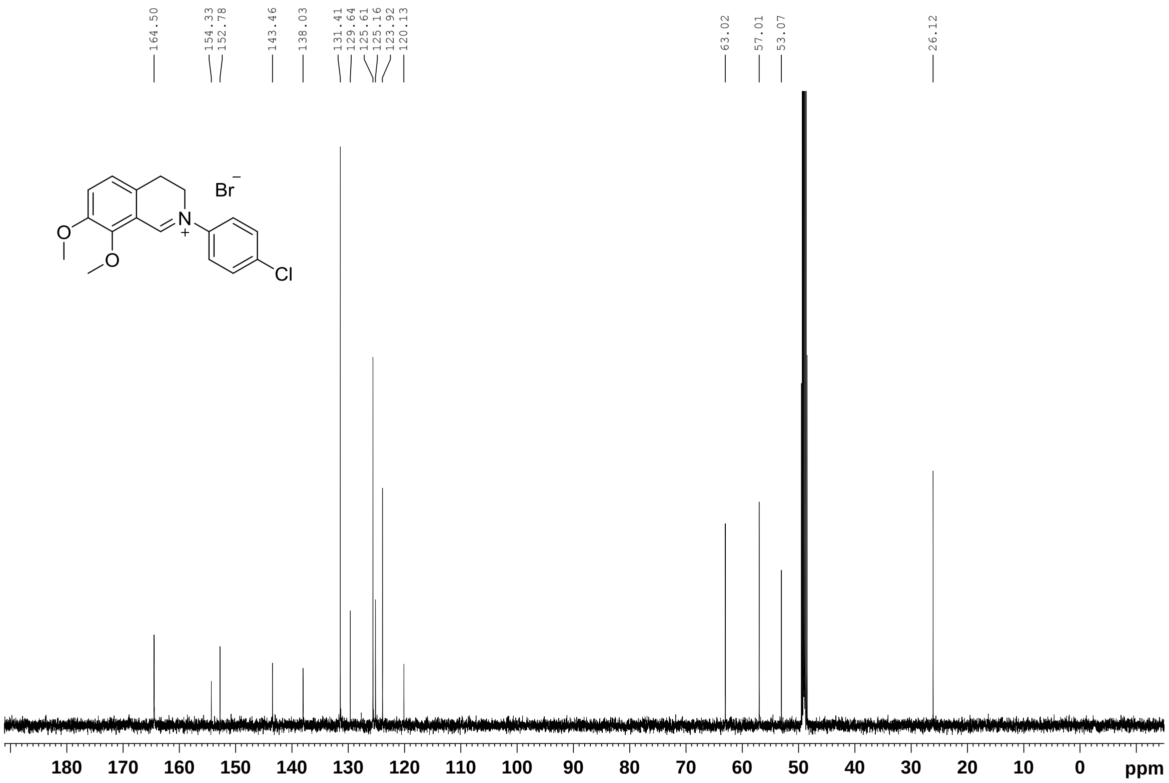

^{13}C NMR of compound **A7**

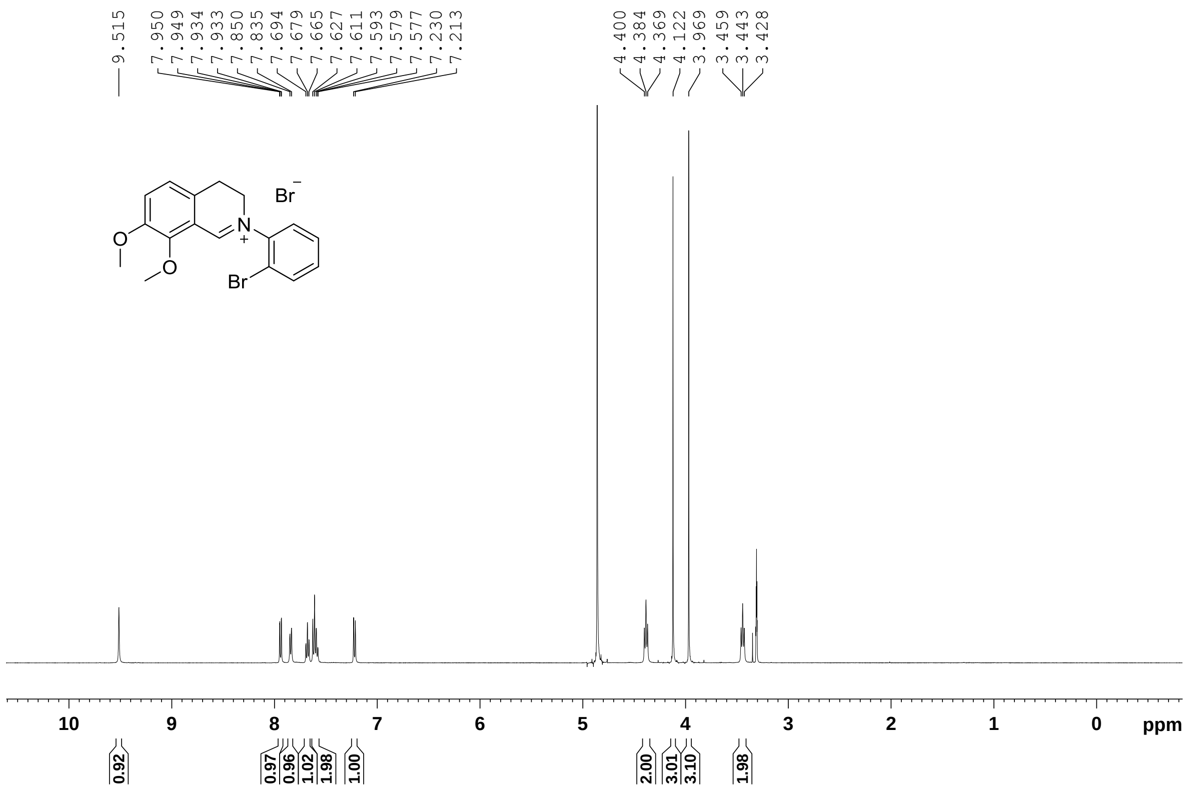

^{1}H NMR of compound **A8**

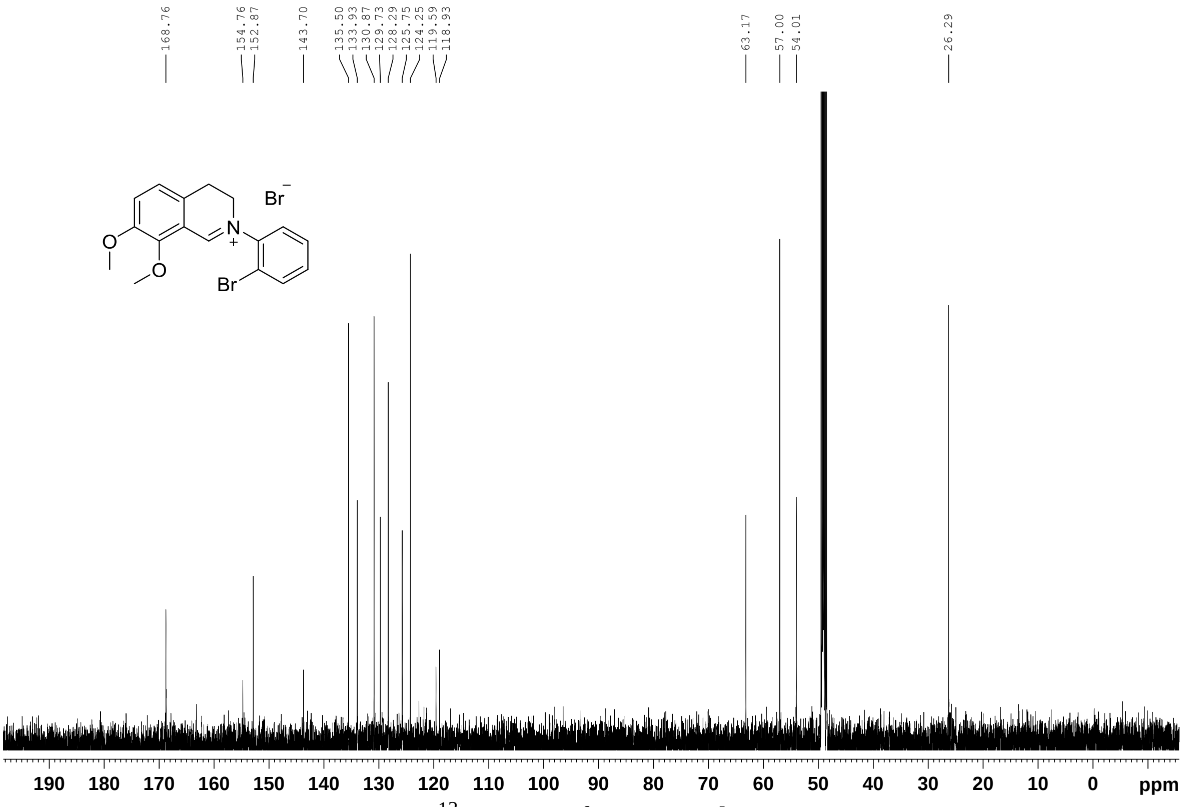

^{13}C NMR of compound **A8**

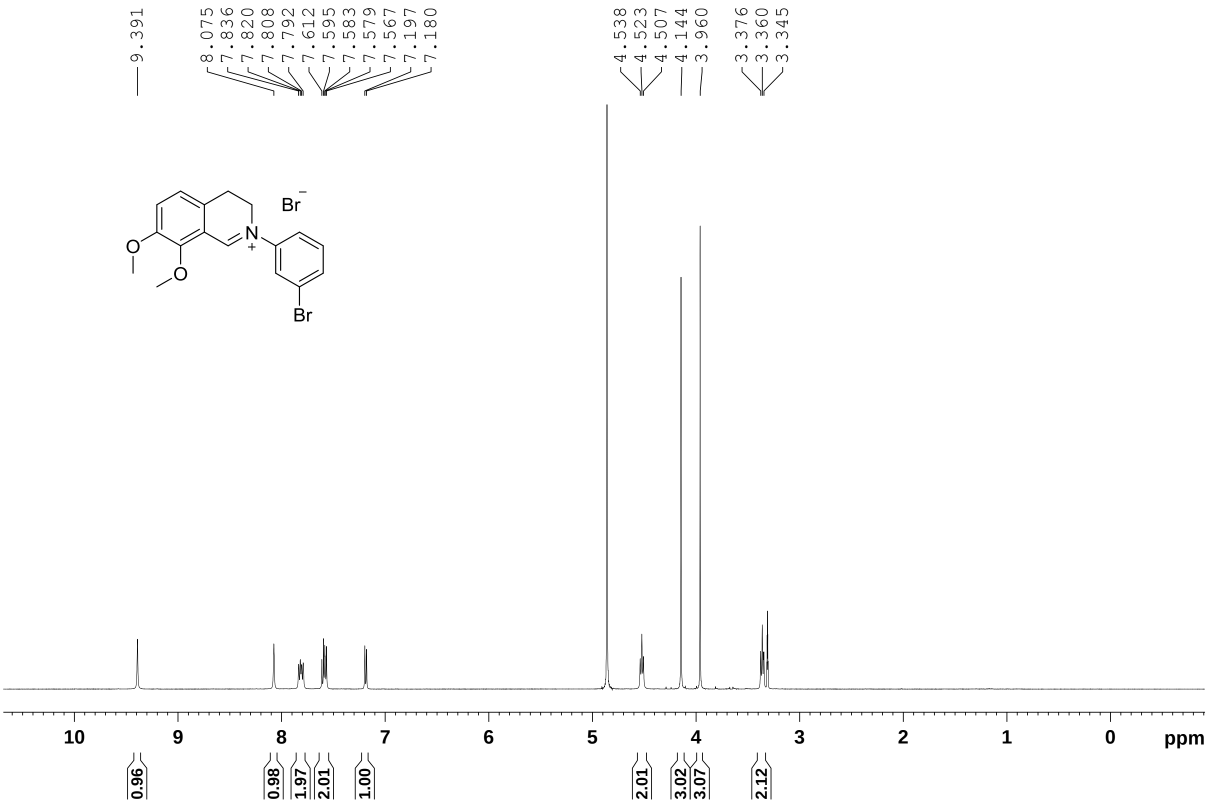

^{1}H NMR of compound **A9**

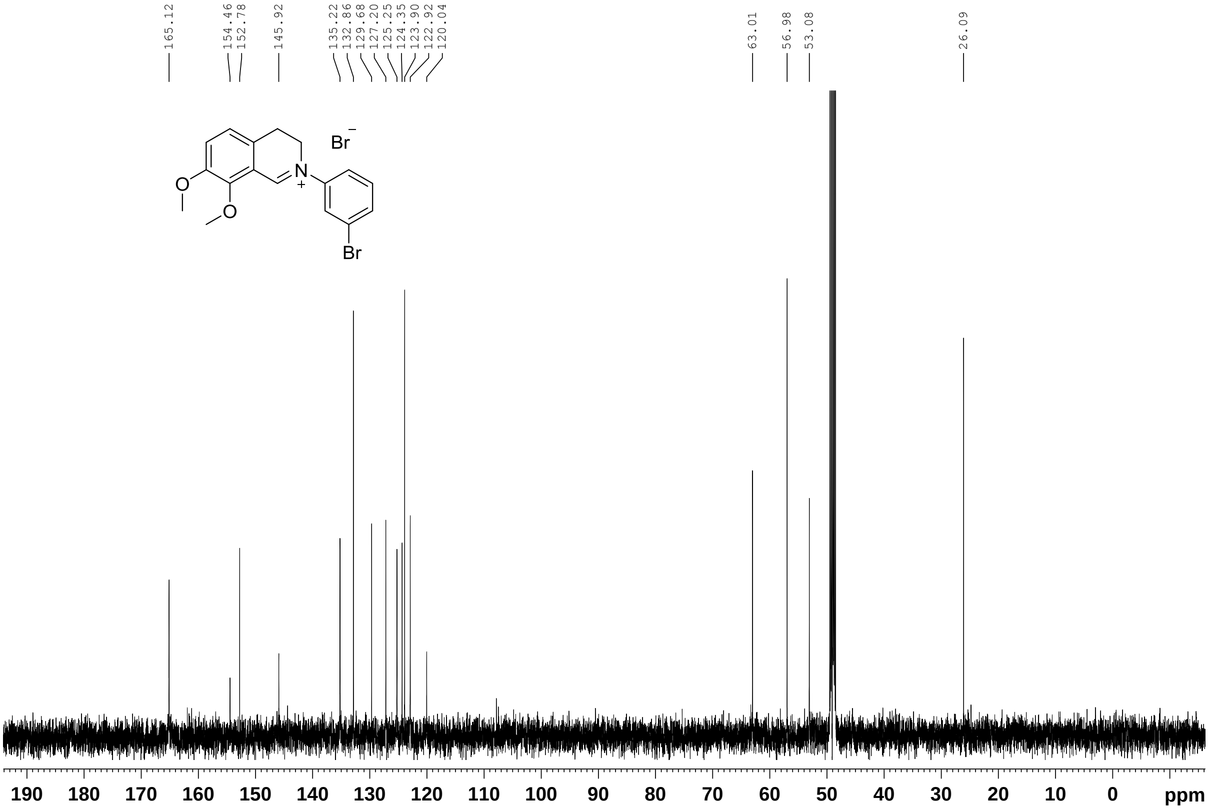

^{13}C NMR of compound **A9**

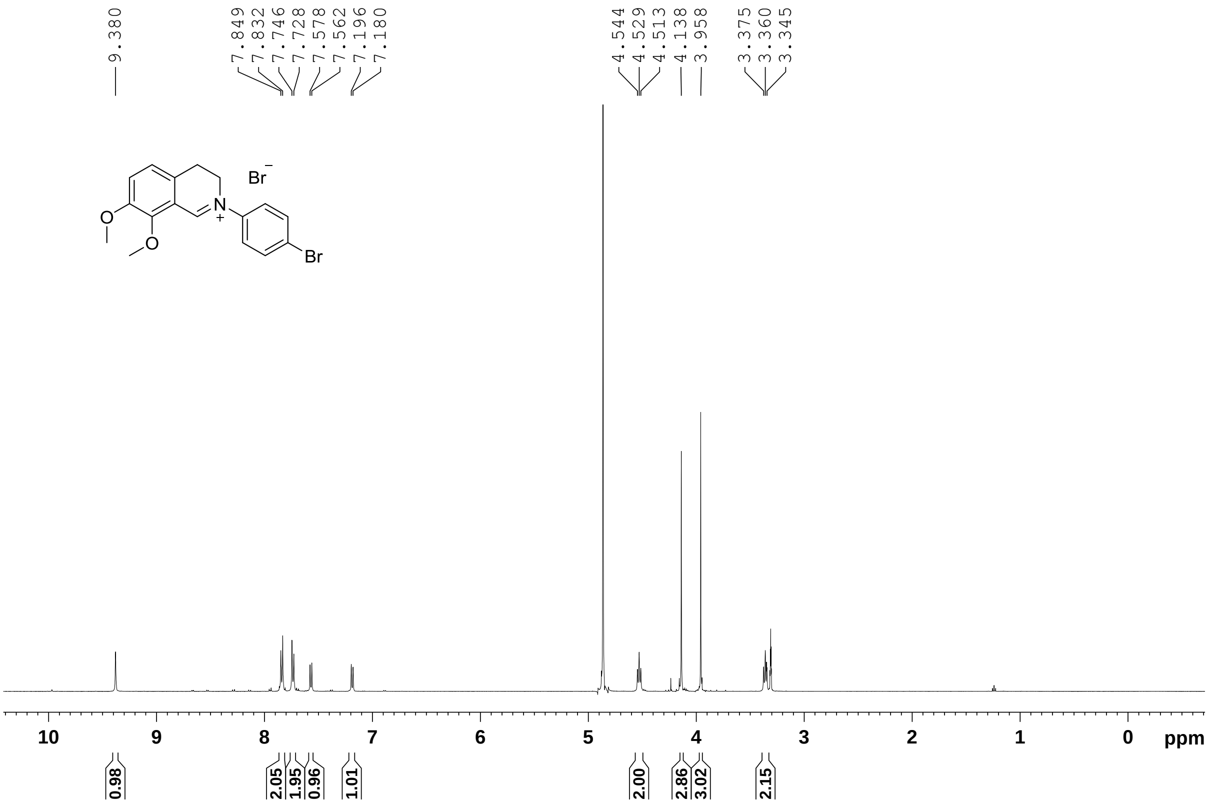

^{1}H NMR of compound **A10**

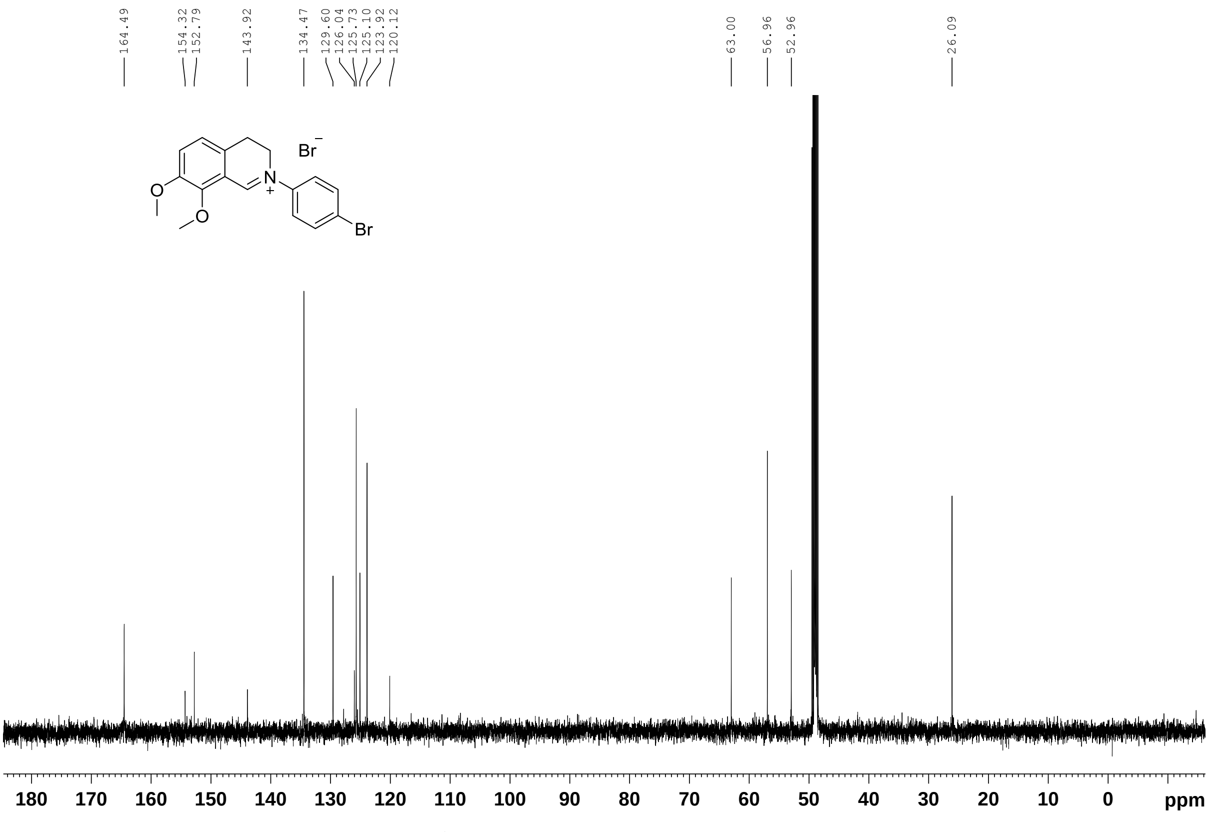

^{13}C NMR of compound **A10**

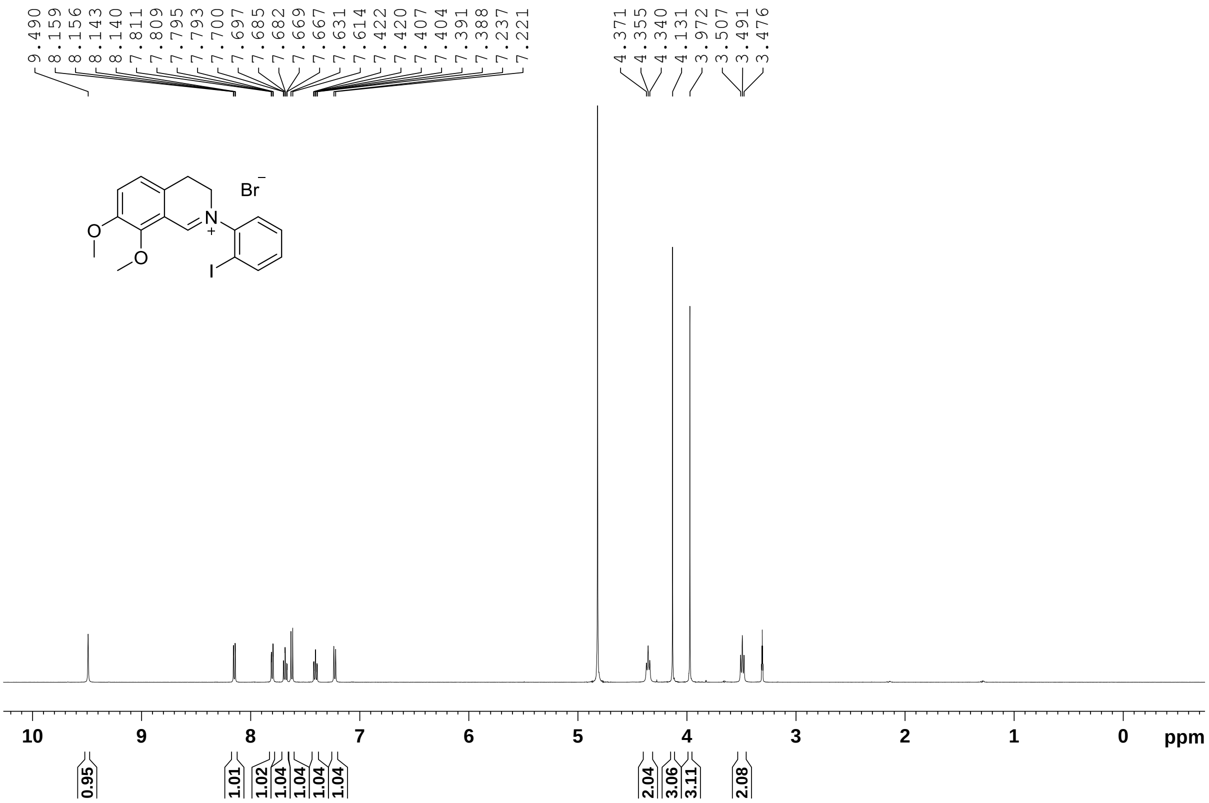

^{1}H NMR of compound **A11**

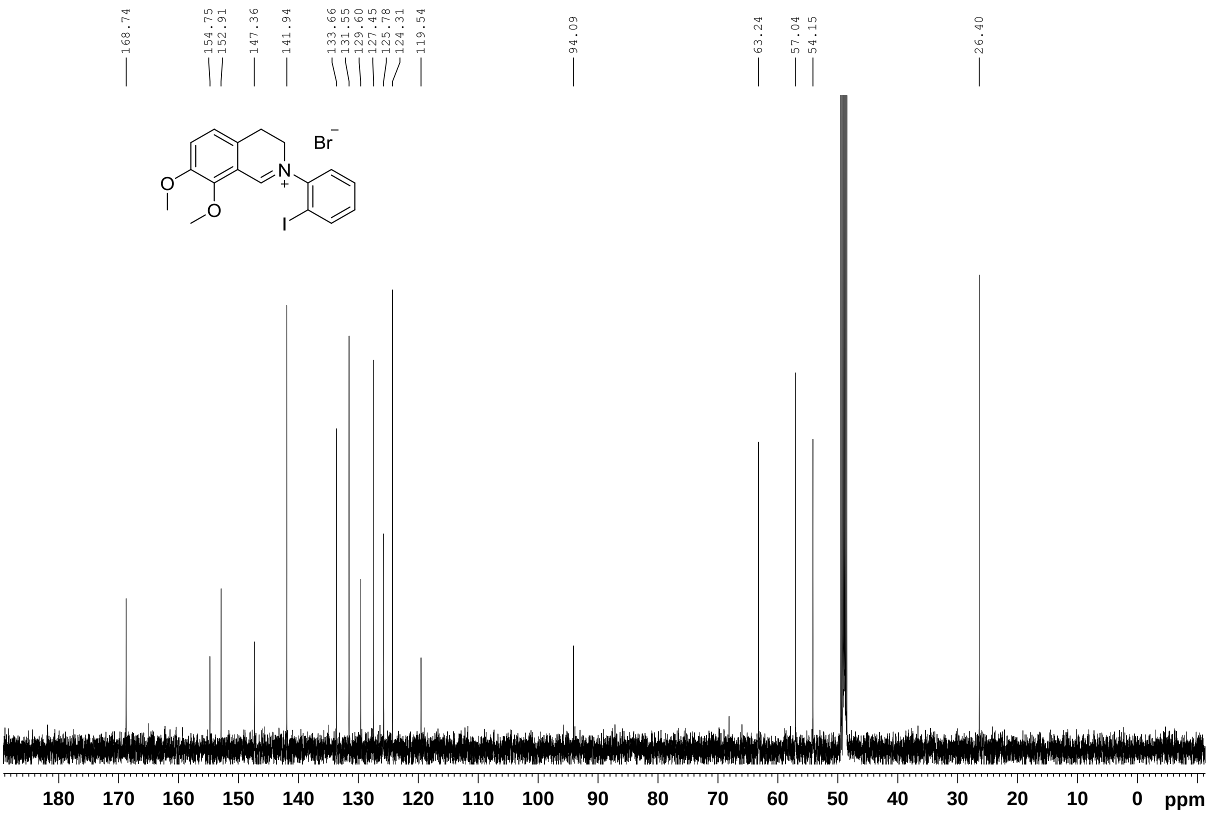

^{13}C NMR of compound **A11**

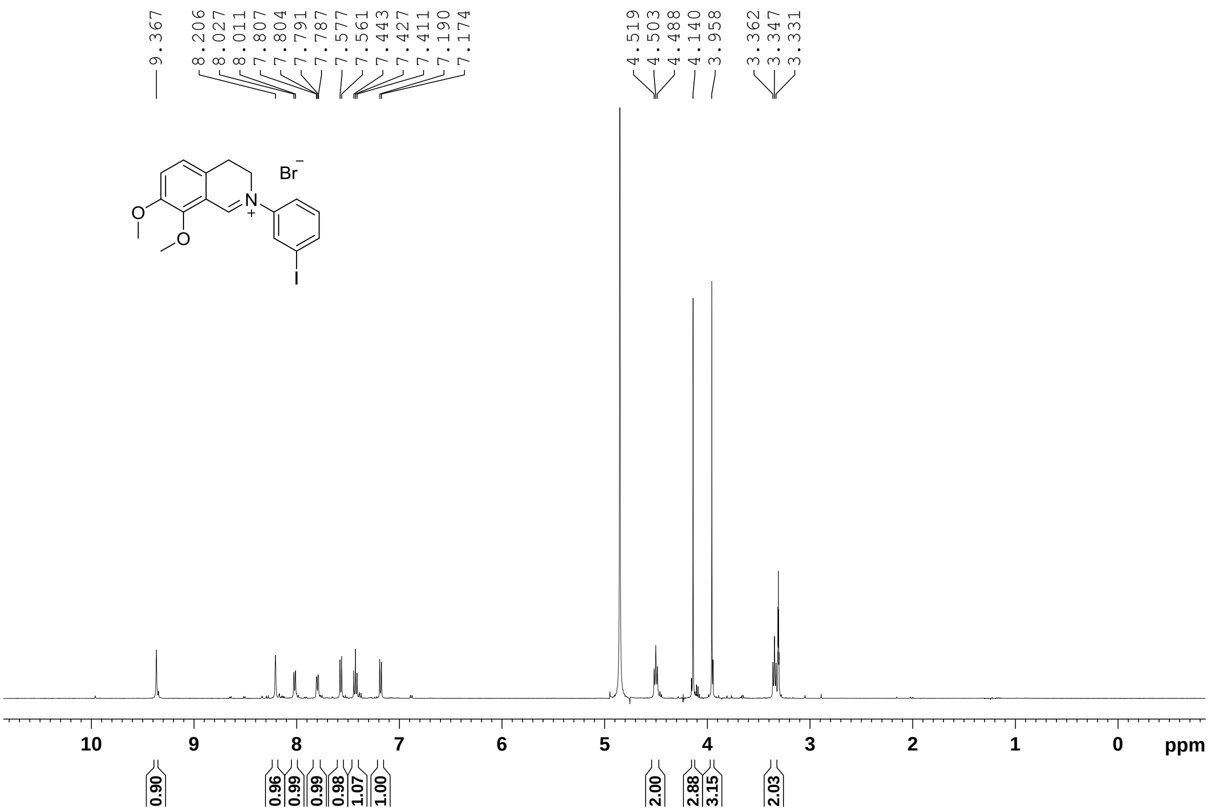

^{1}H NMR of compound **A12**

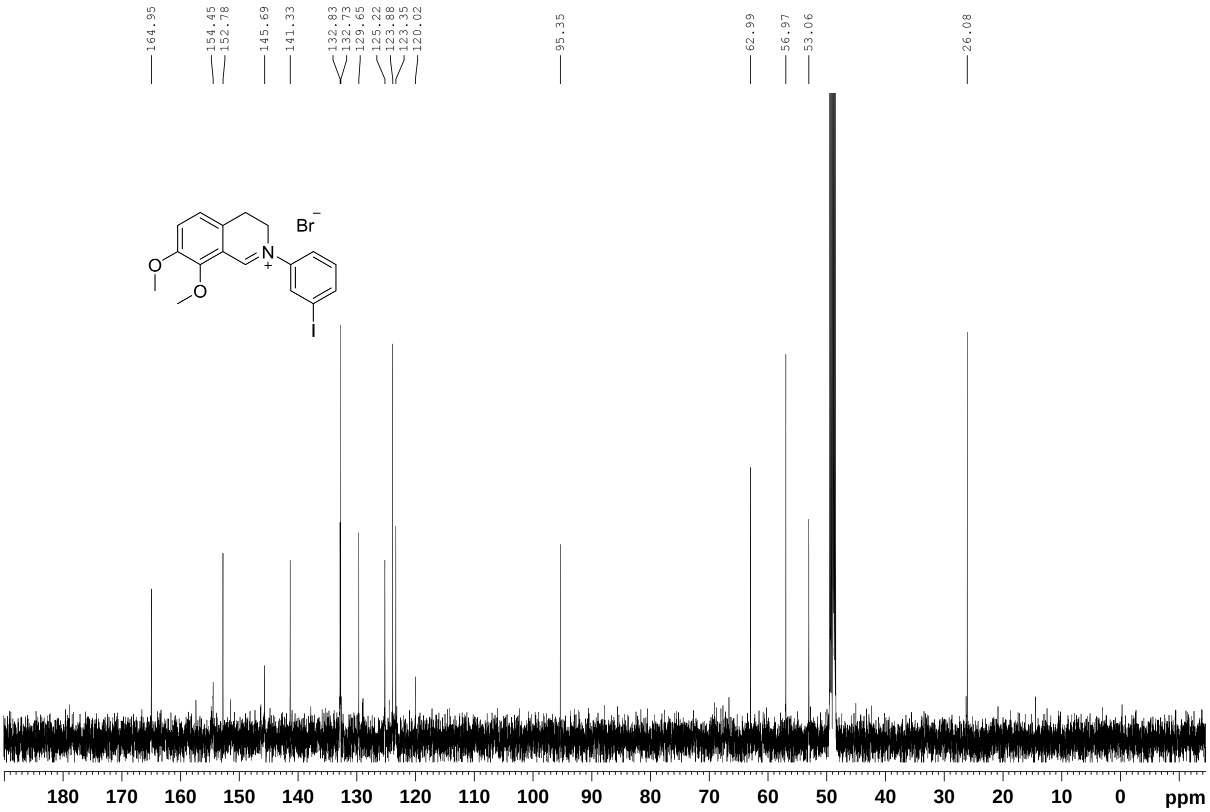

^{13}C NMR of compound **A12**

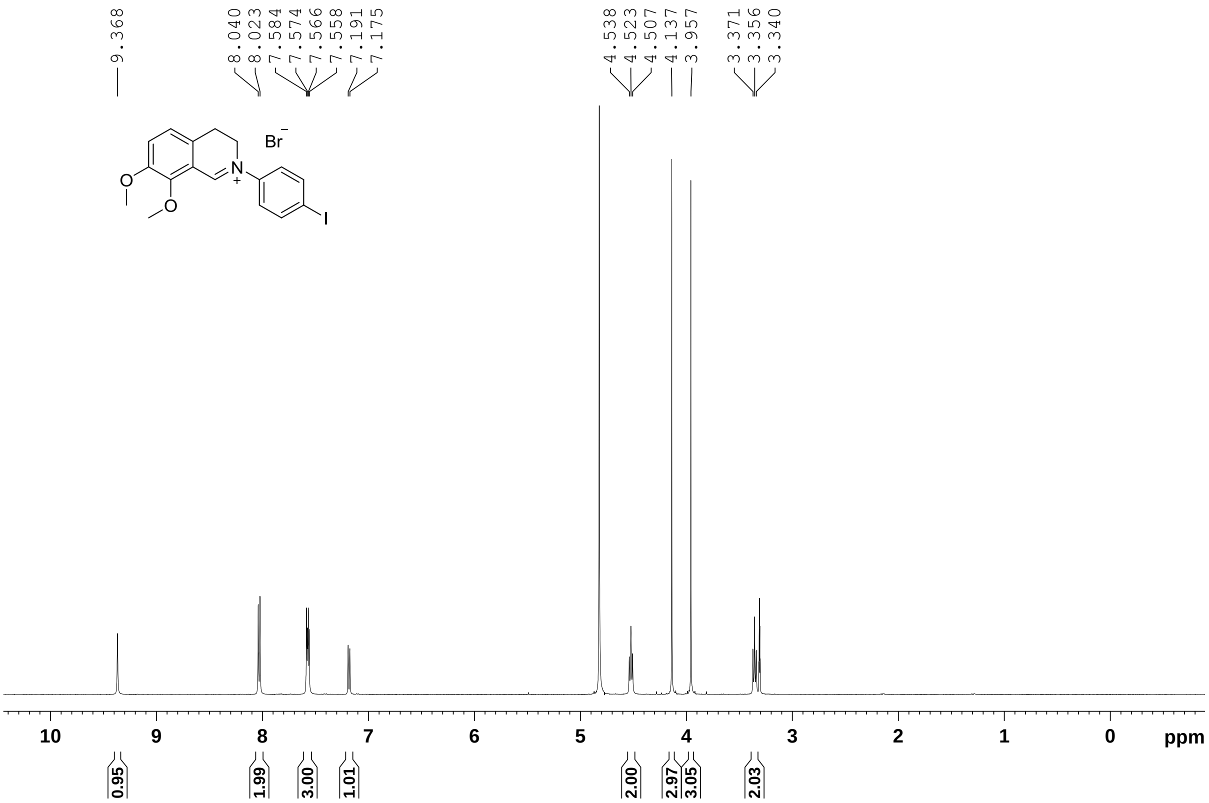

^{1}H NMR of compound **A13**

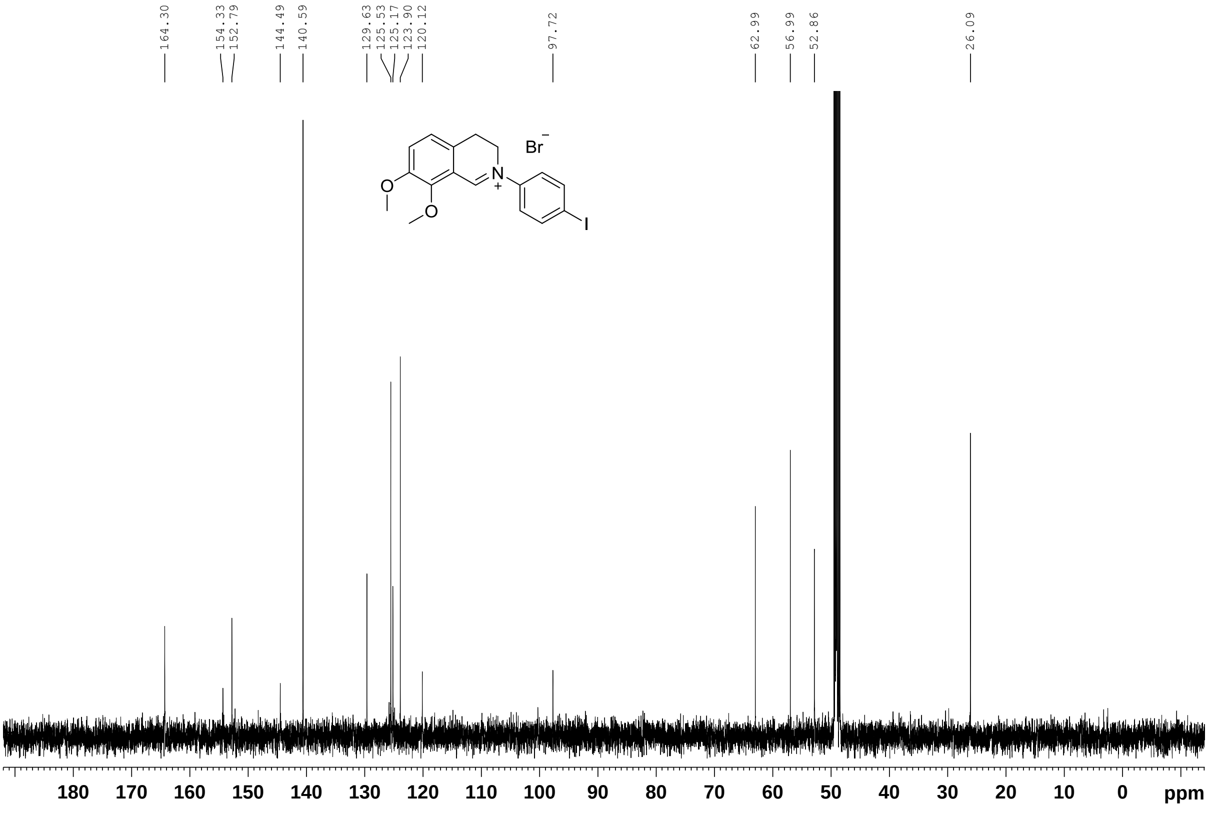

^{13}C NMR of compound **A13**

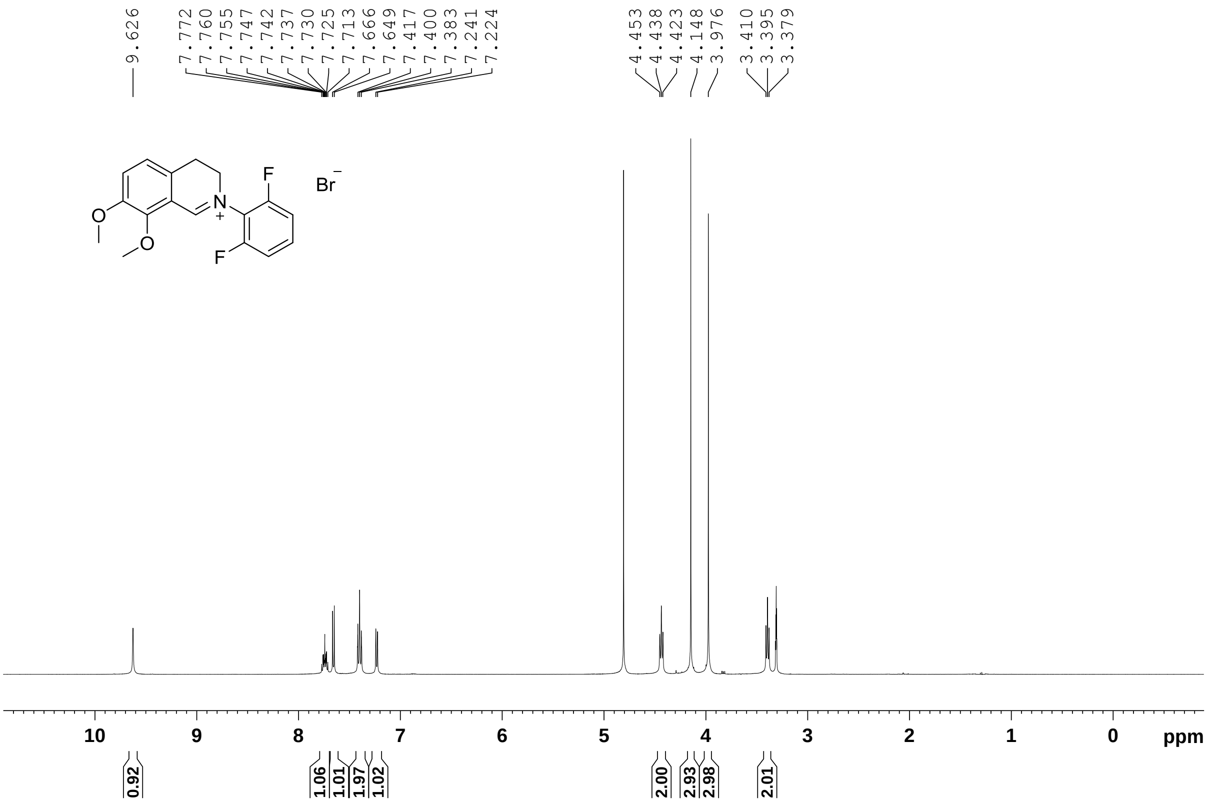

^{1}H NMR of compound **A14**

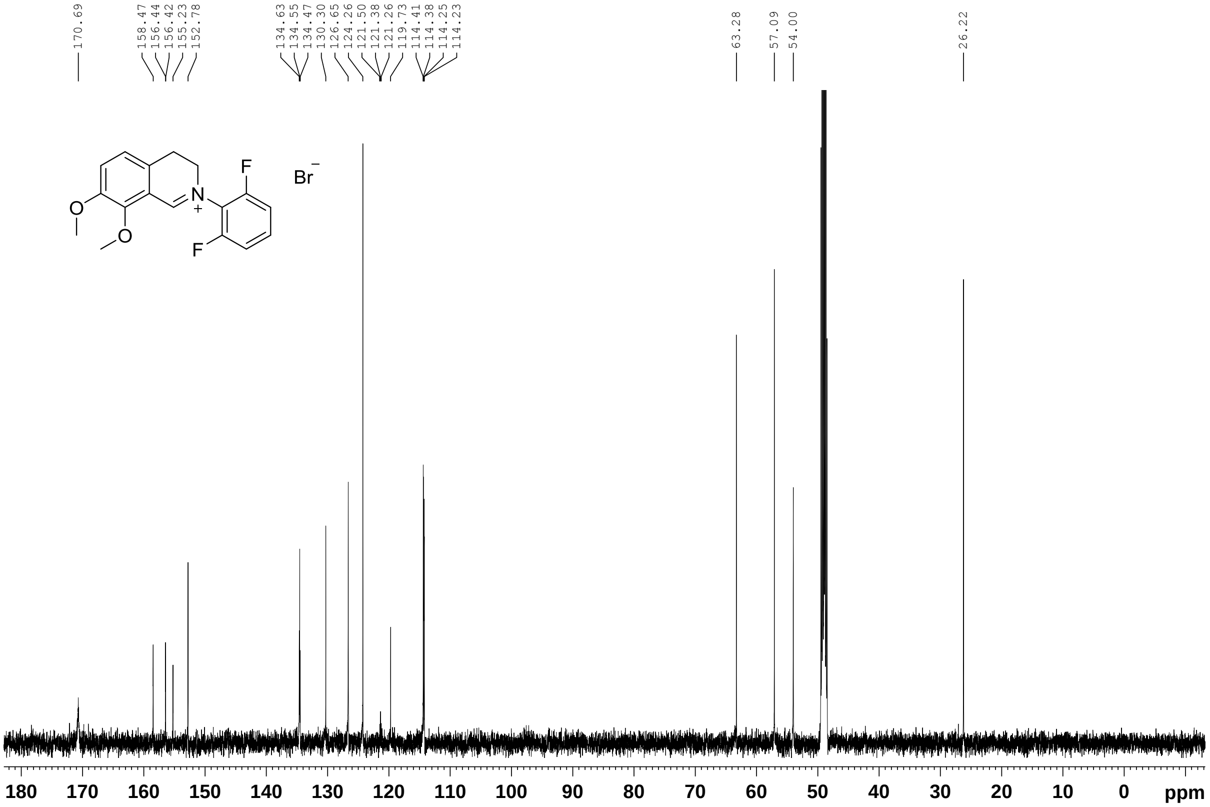

^{13}C NMR of compound **A14**

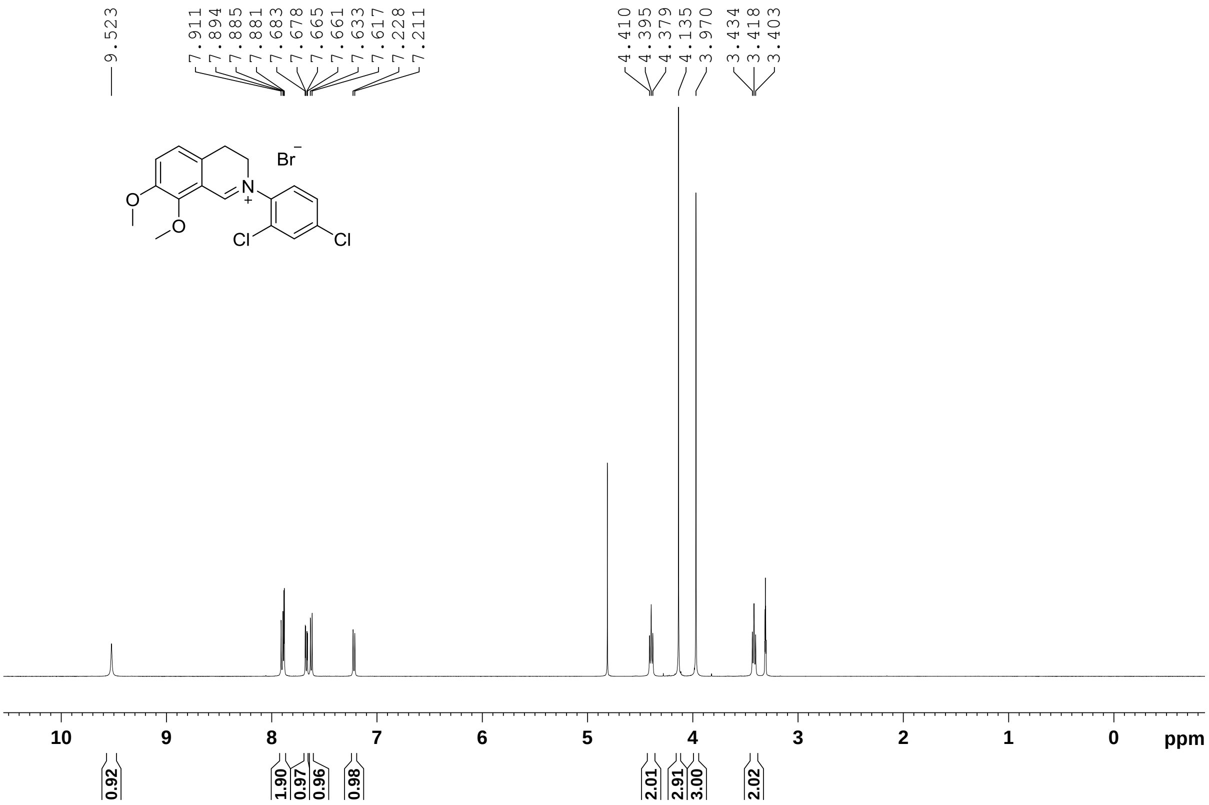

^{1}H NMR of compound **A15**

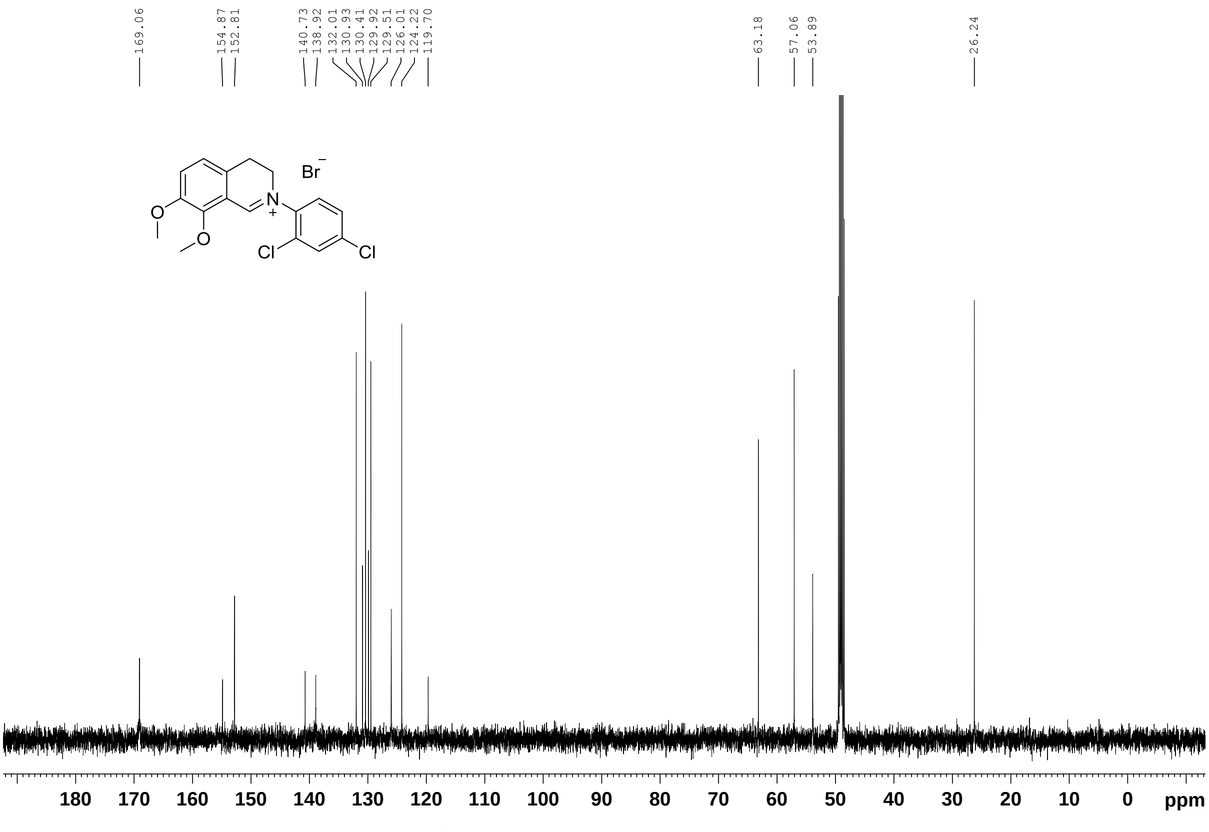

^{13}C NMR of compound **A15**

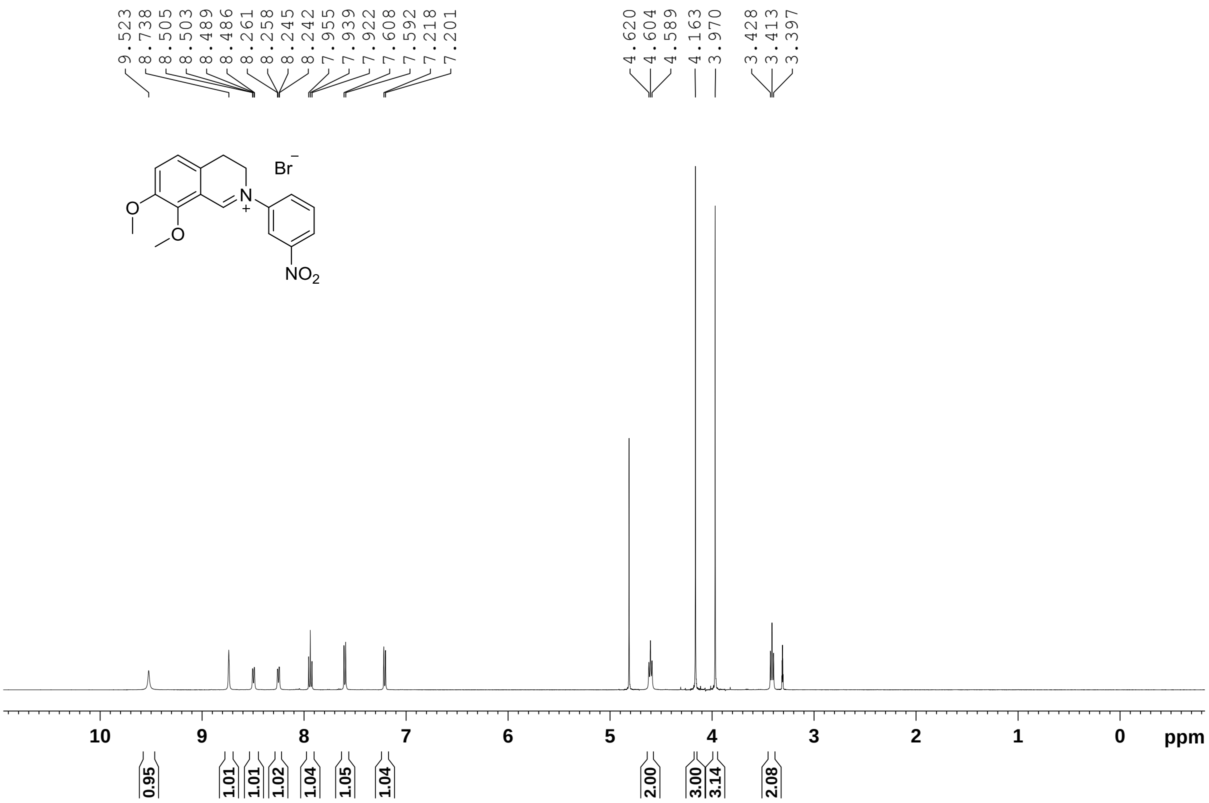

^{1}H NMR of compound **A16**

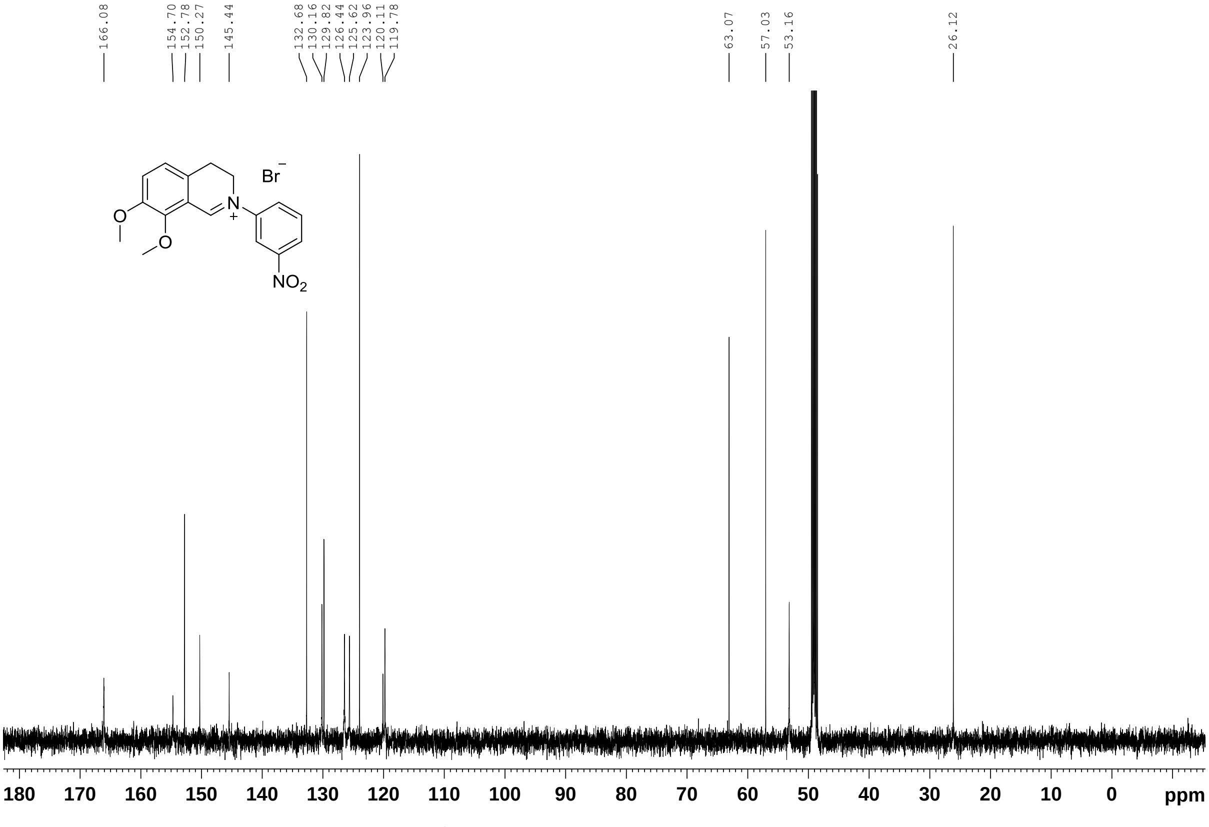

^{13}C NMR of compound **A16**

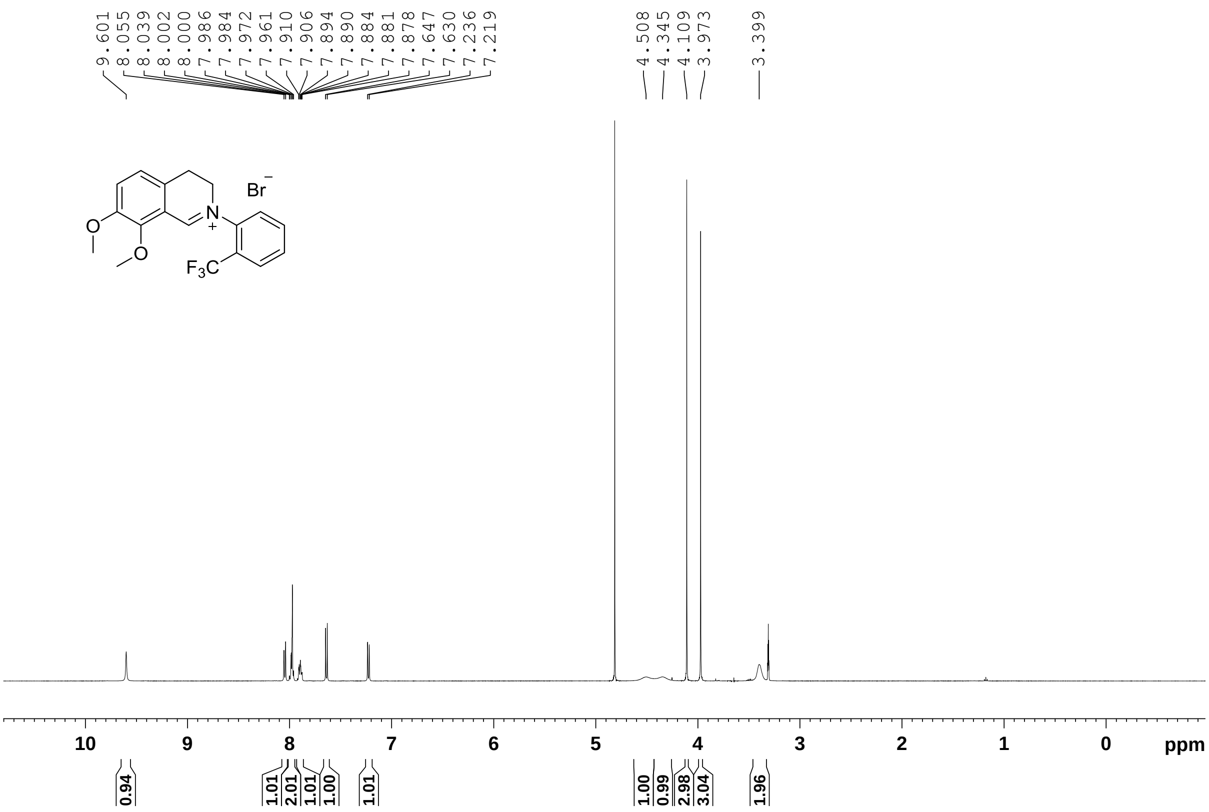

^{1}H NMR of compound **A17**

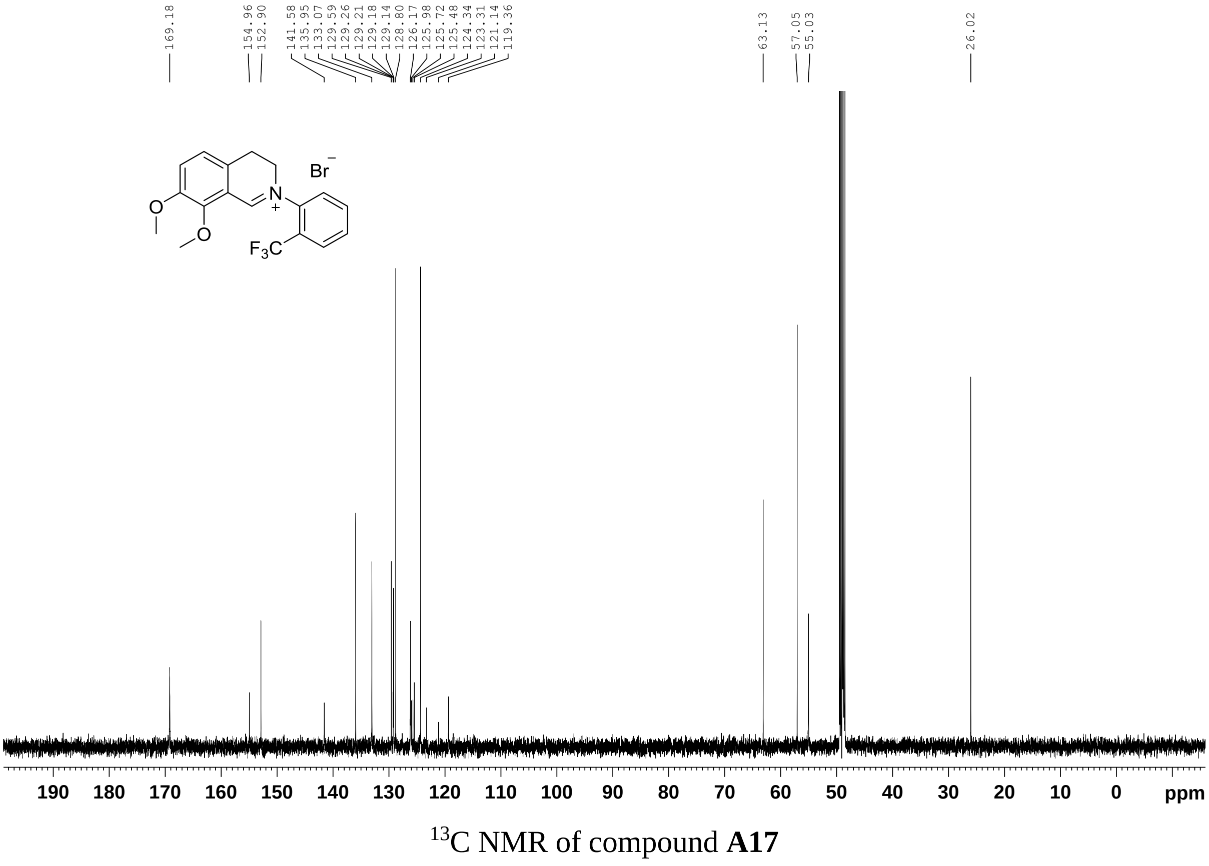

^{13}C NMR of compound **A17**

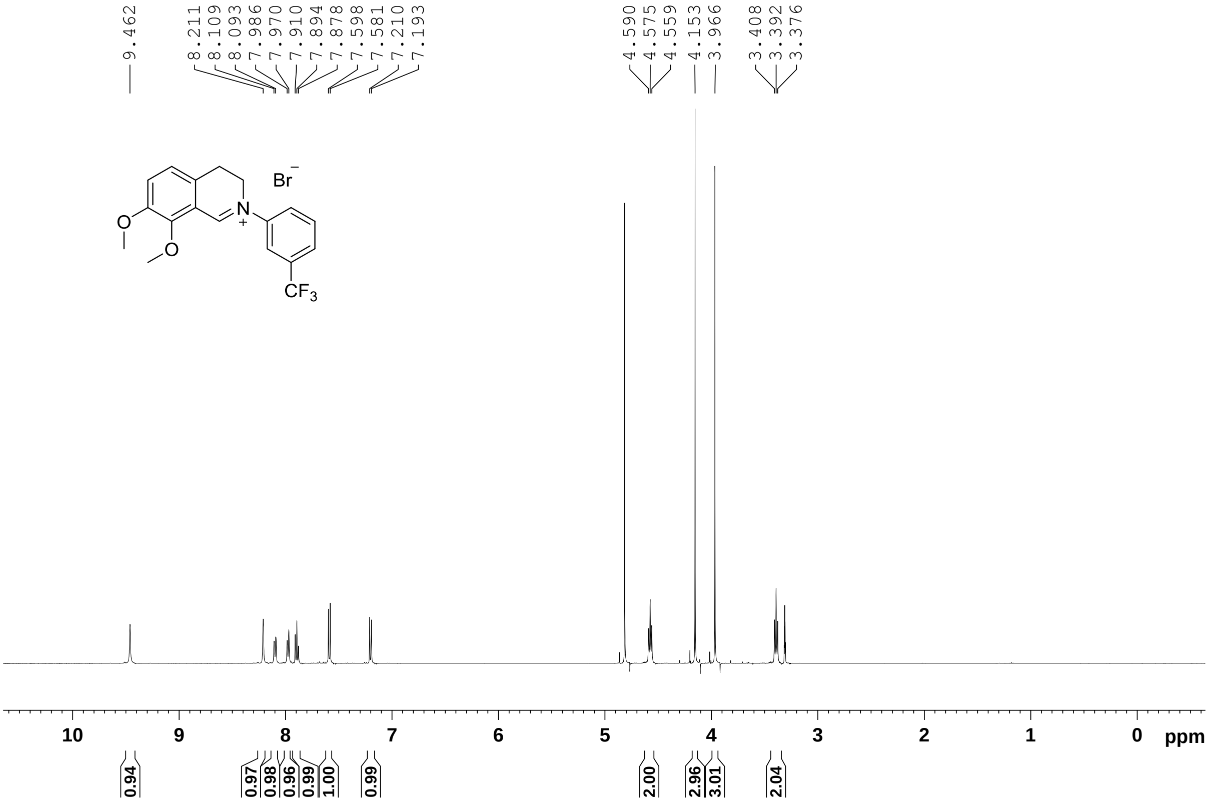

^{1}H NMR of compound **A18**

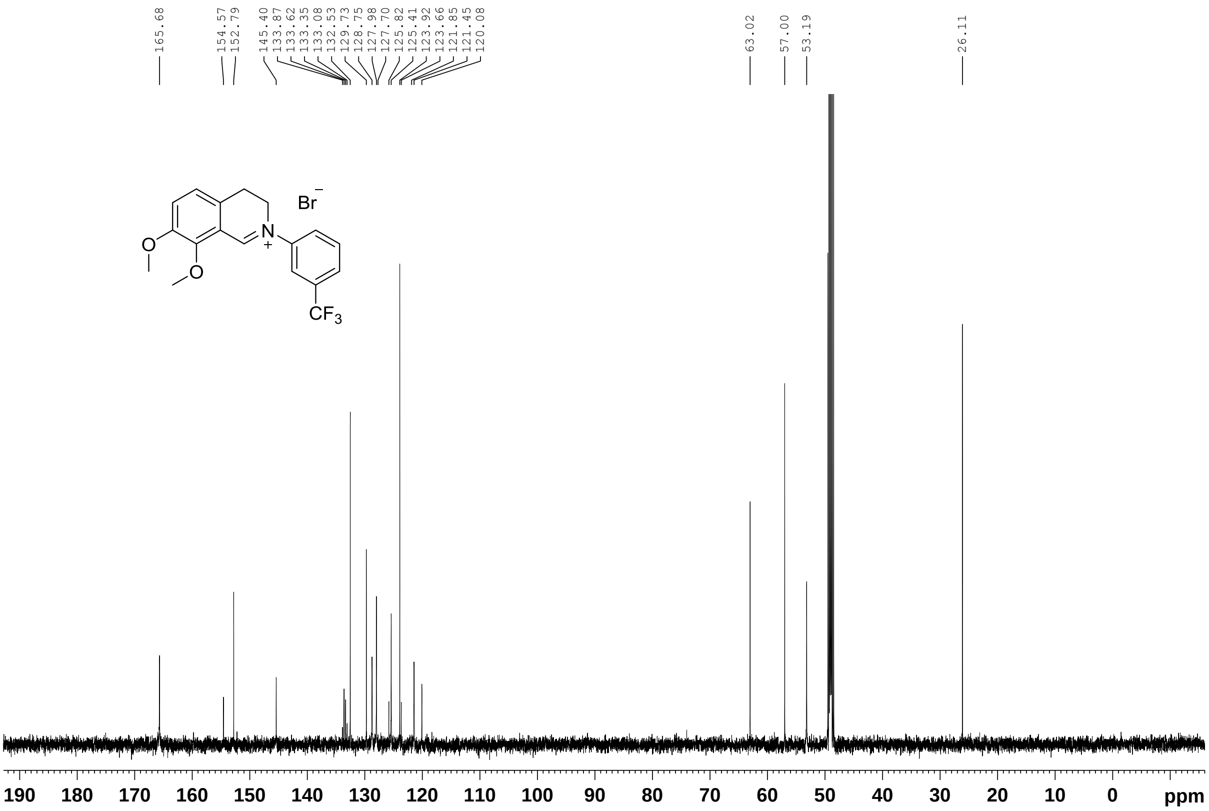

^{13}C NMR of compound **A18**

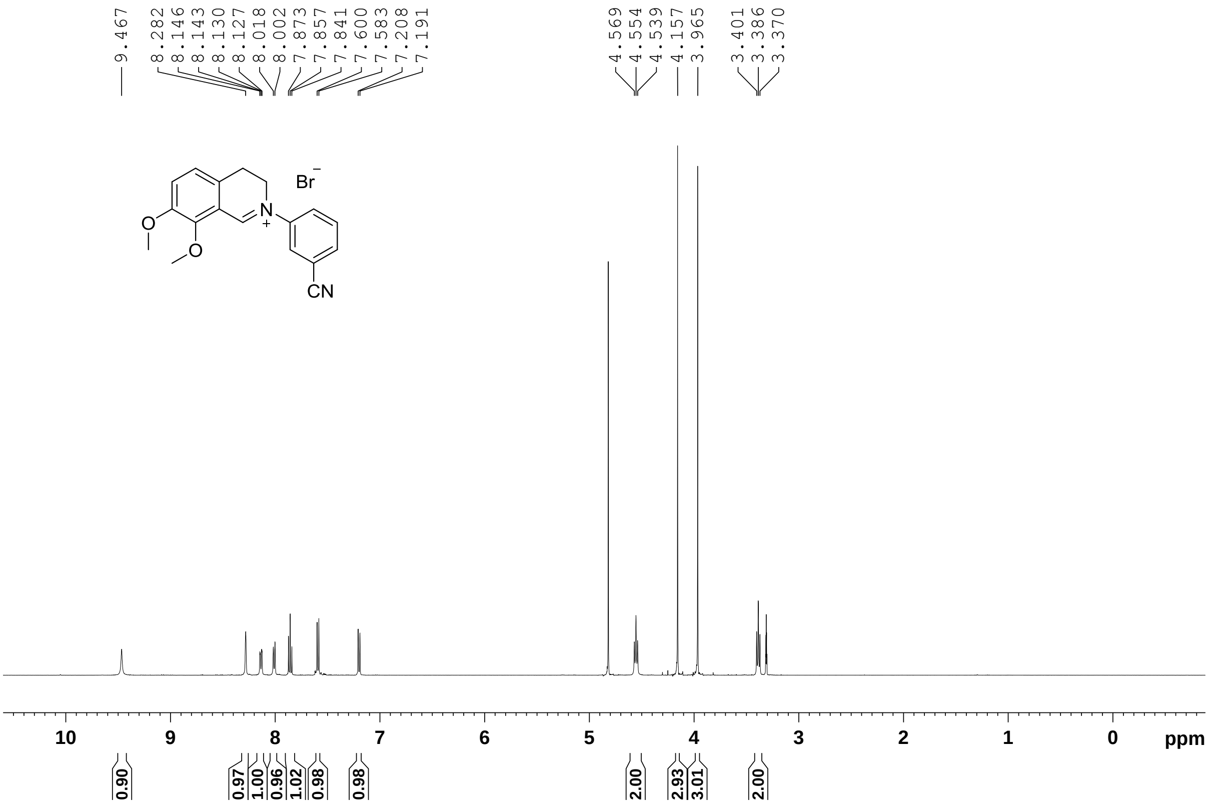

^{1}H NMR of compound **A19**

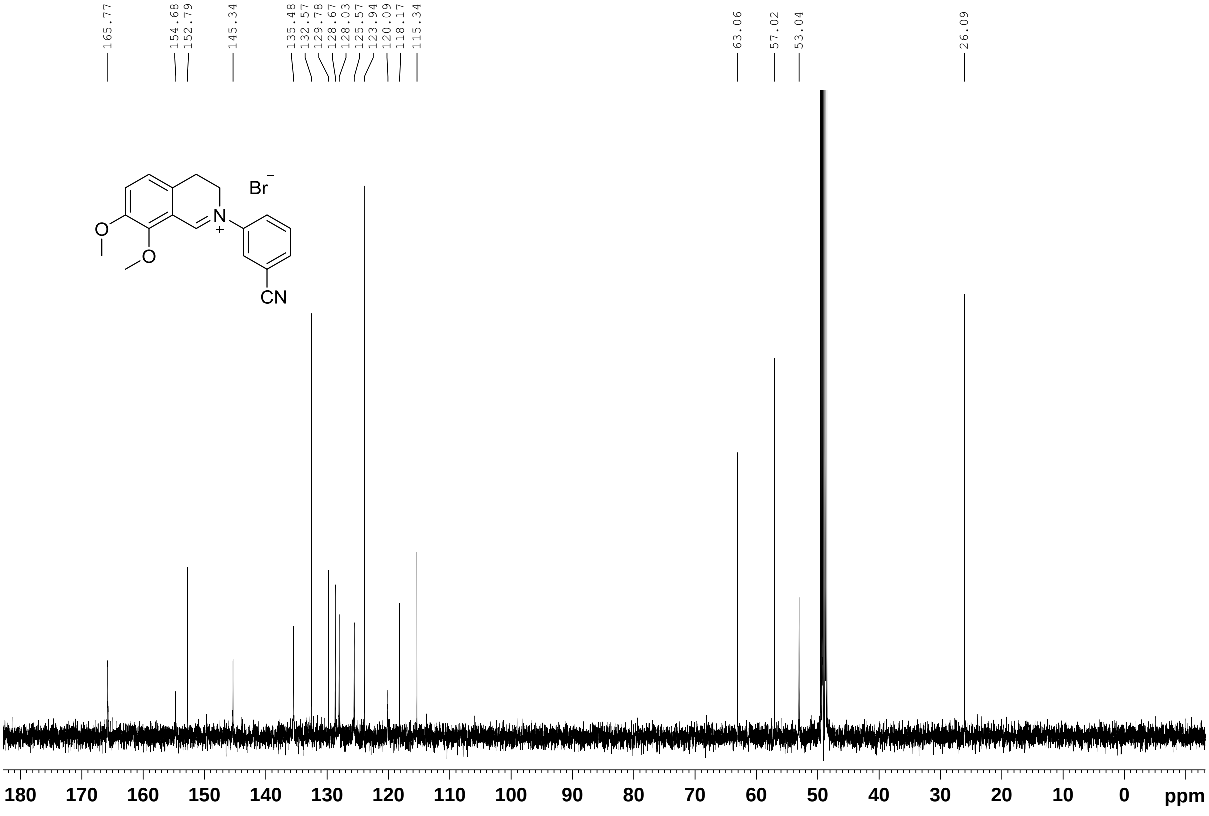

^{13}C NMR of compound **A19**

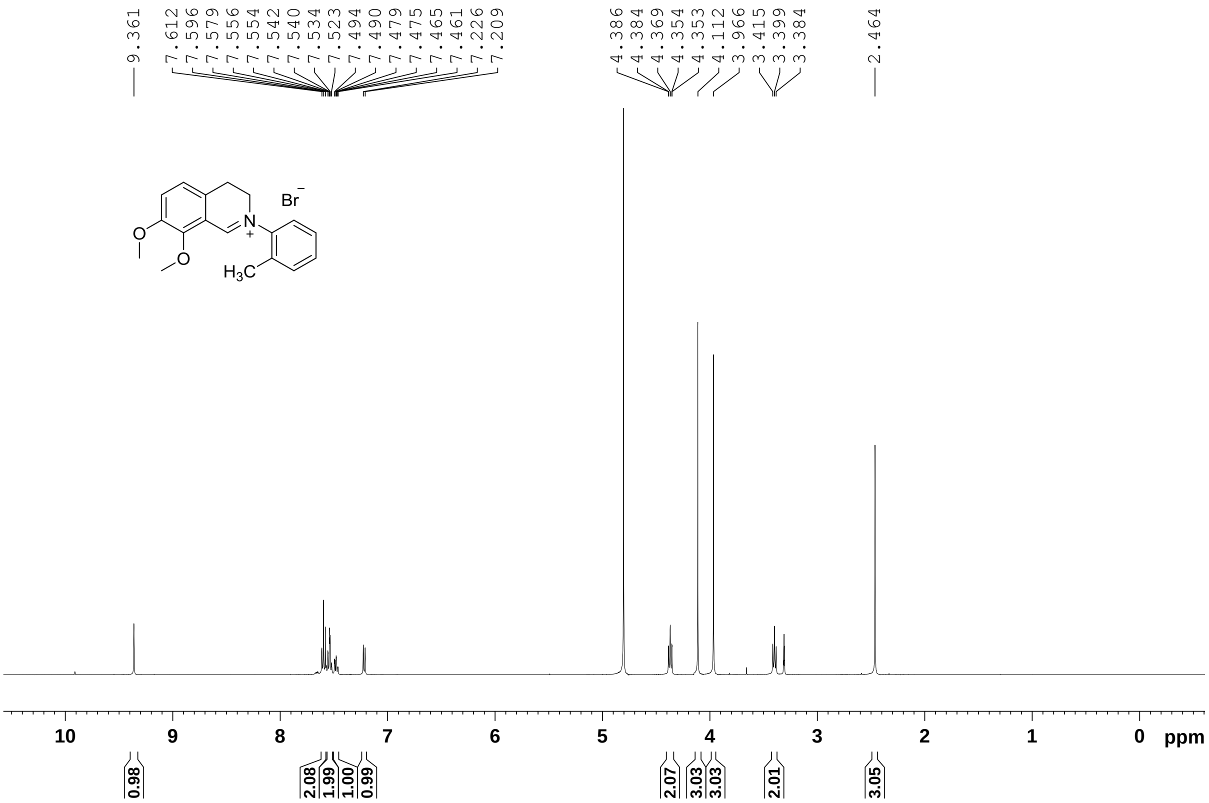

^{1}H NMR of compound **A20**

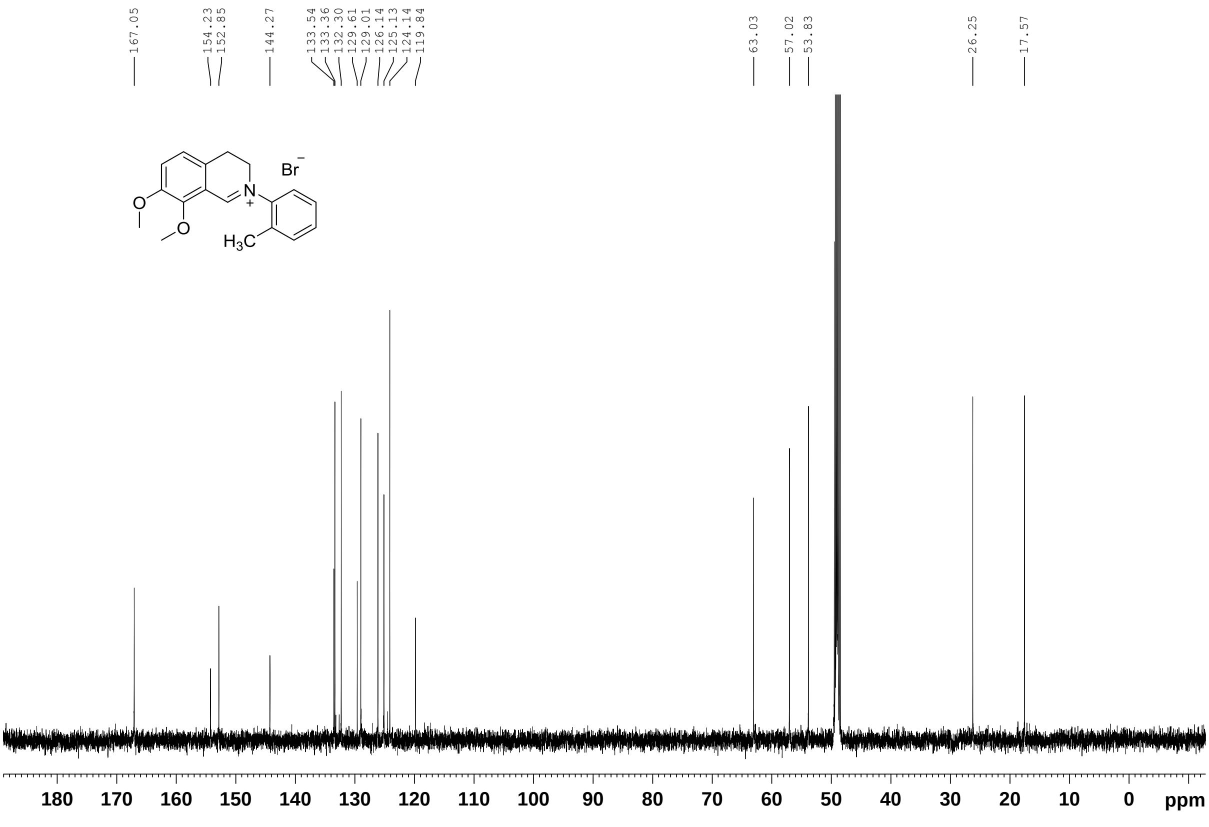

^{13}C NMR of compound **A20**

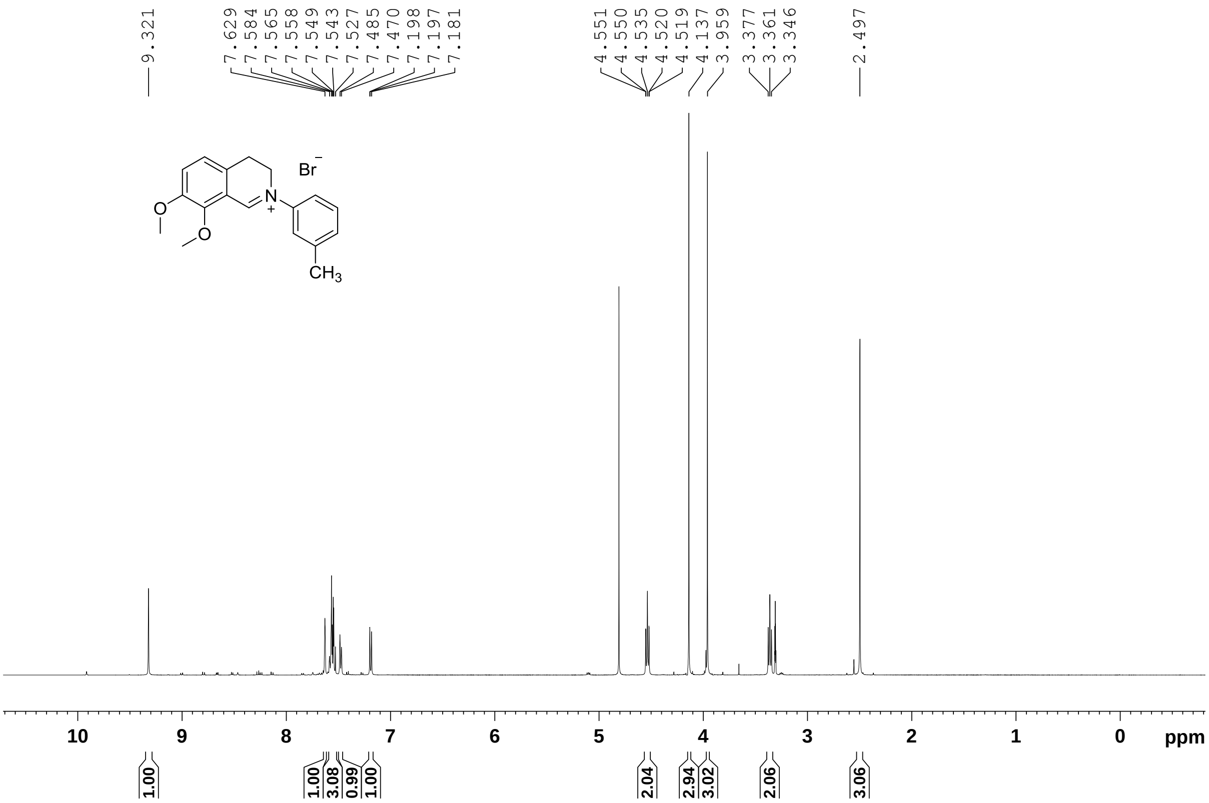

^{1}H NMR of compound **A21**

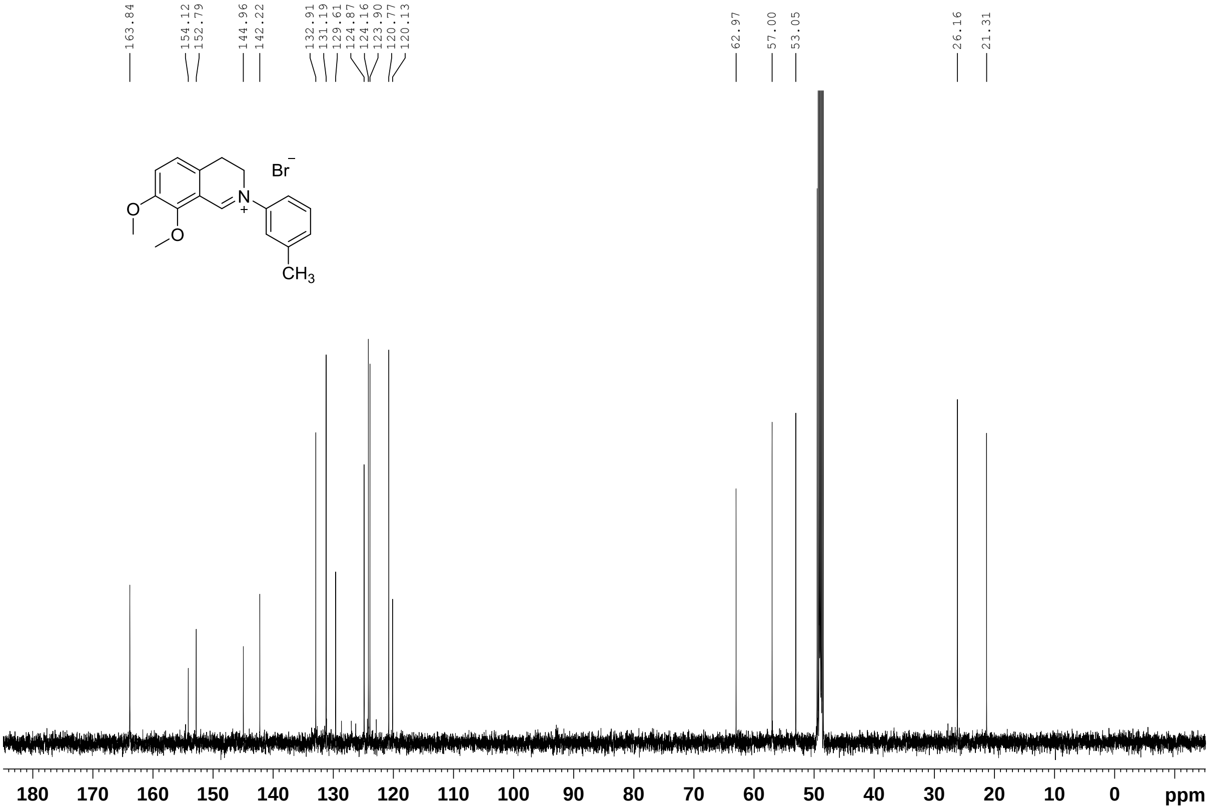

^{13}C NMR of compound **A21**

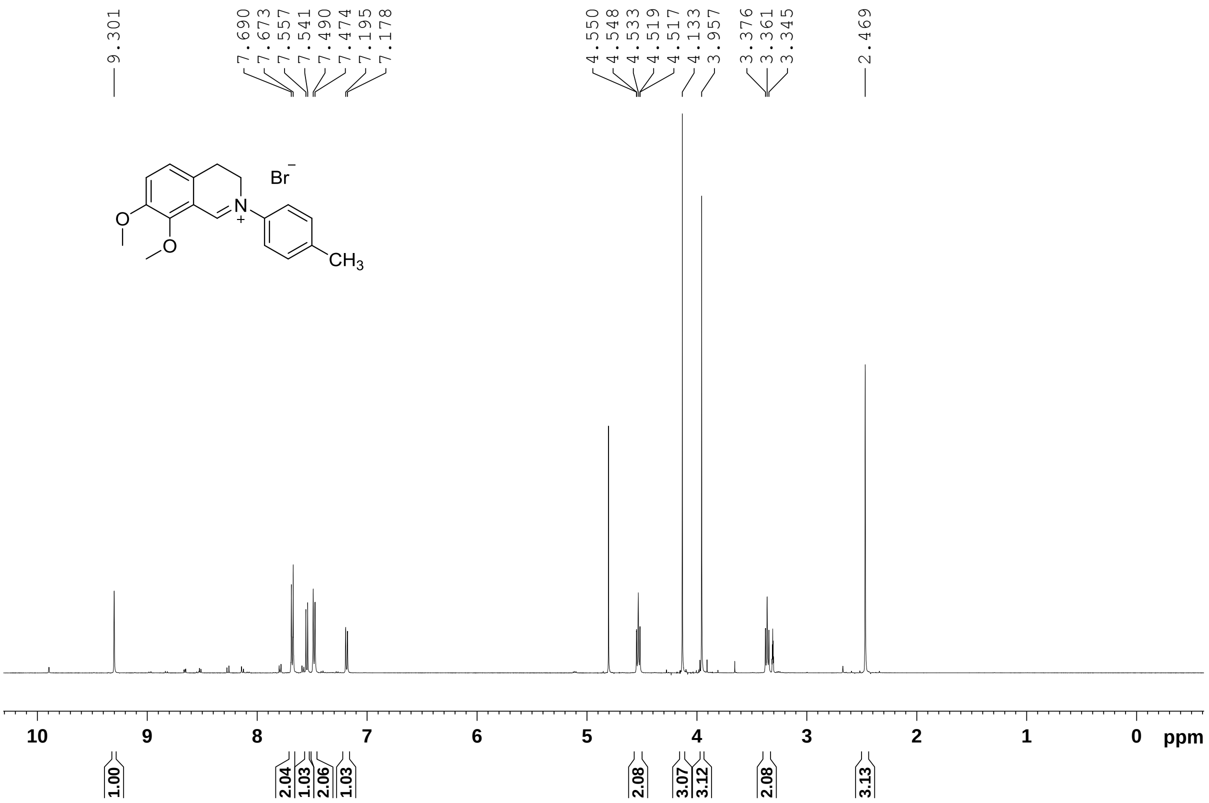

^{1}H NMR of compound **A22**

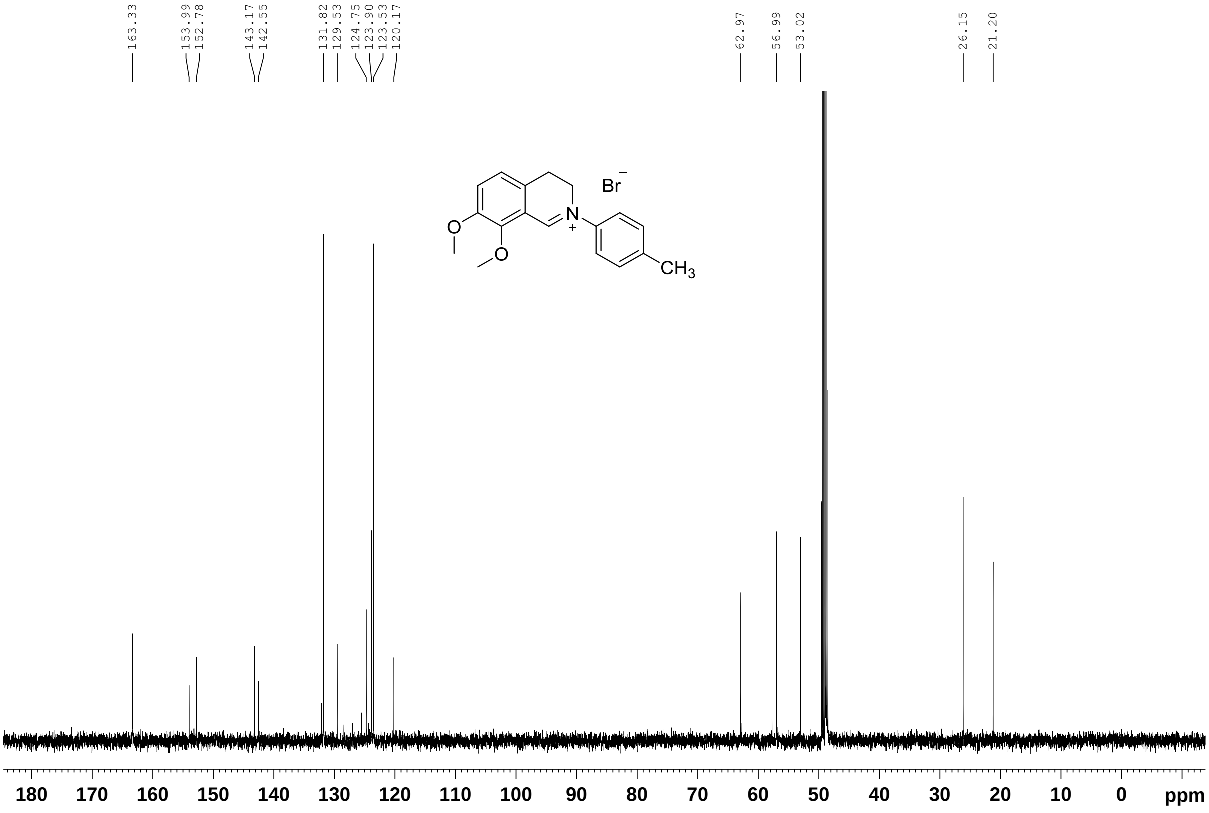

^{13}C NMR of compound **A22**

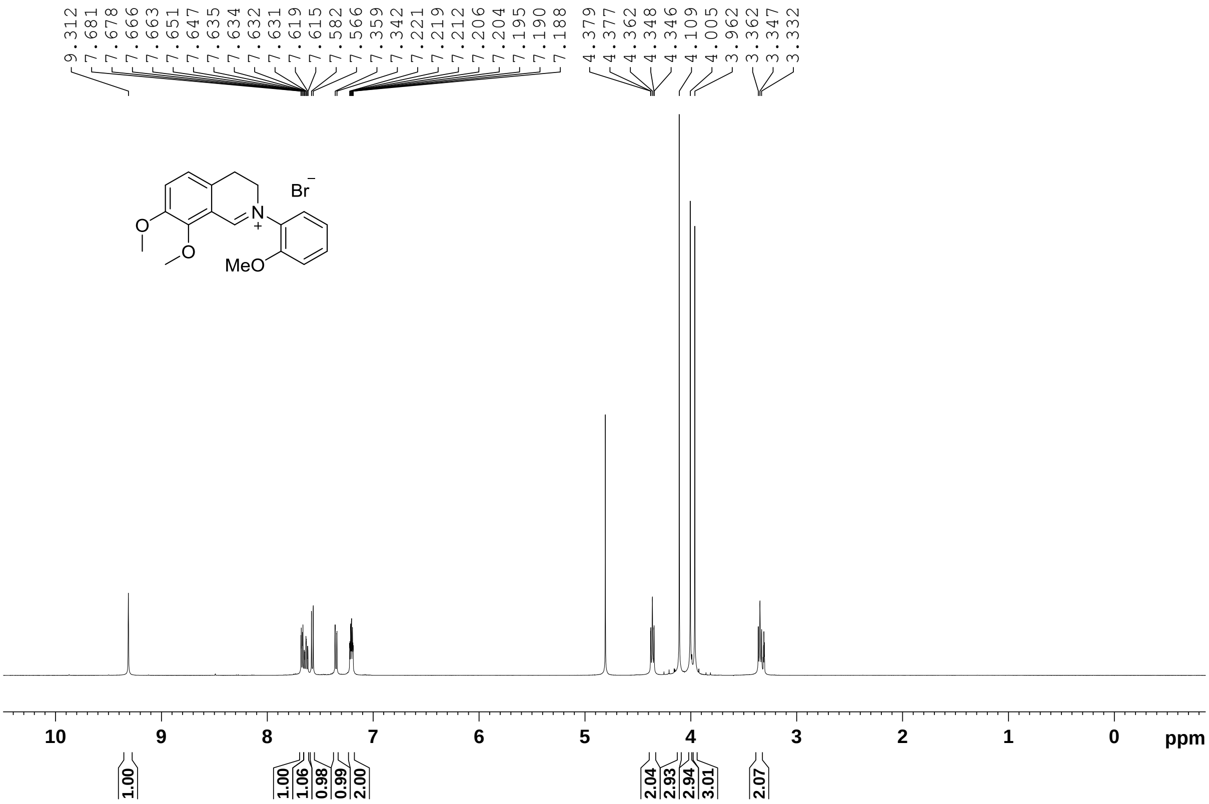

^{1}H NMR of compound **A23**

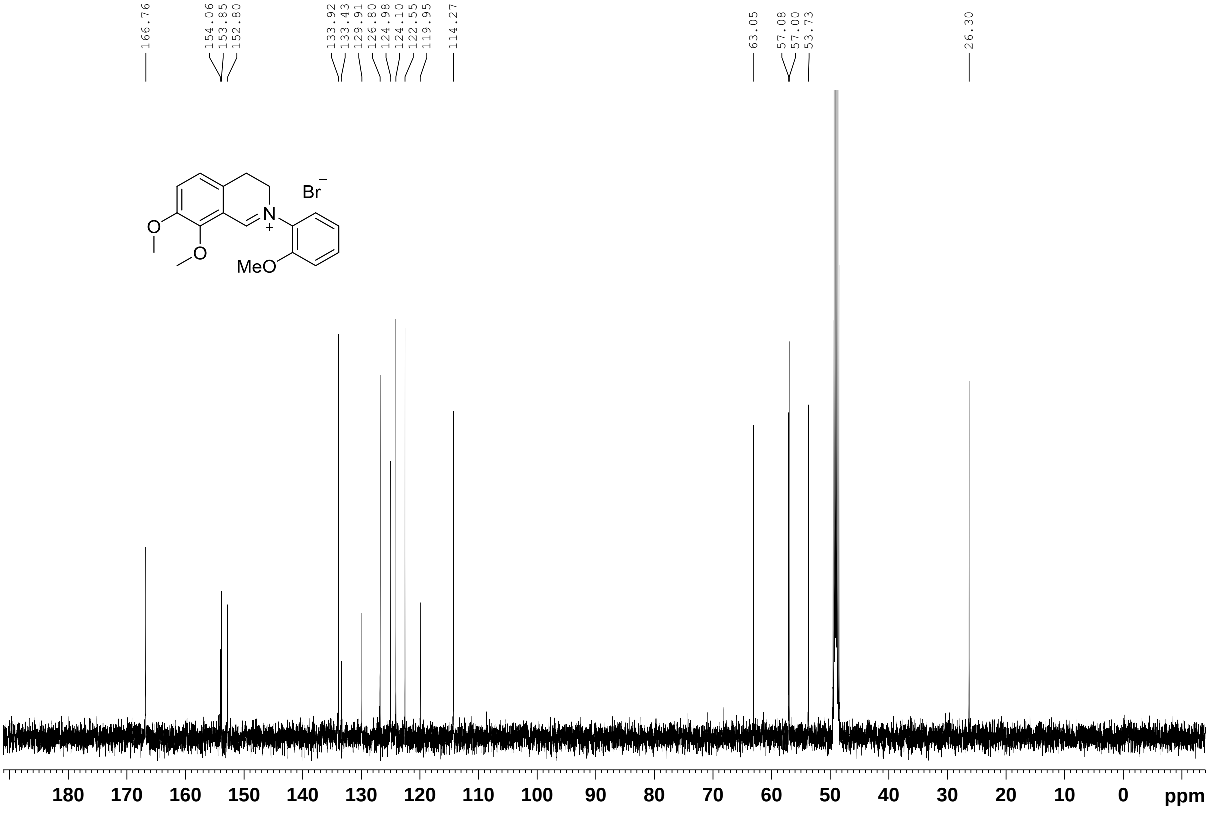

^{13}C NMR of compound **A23**

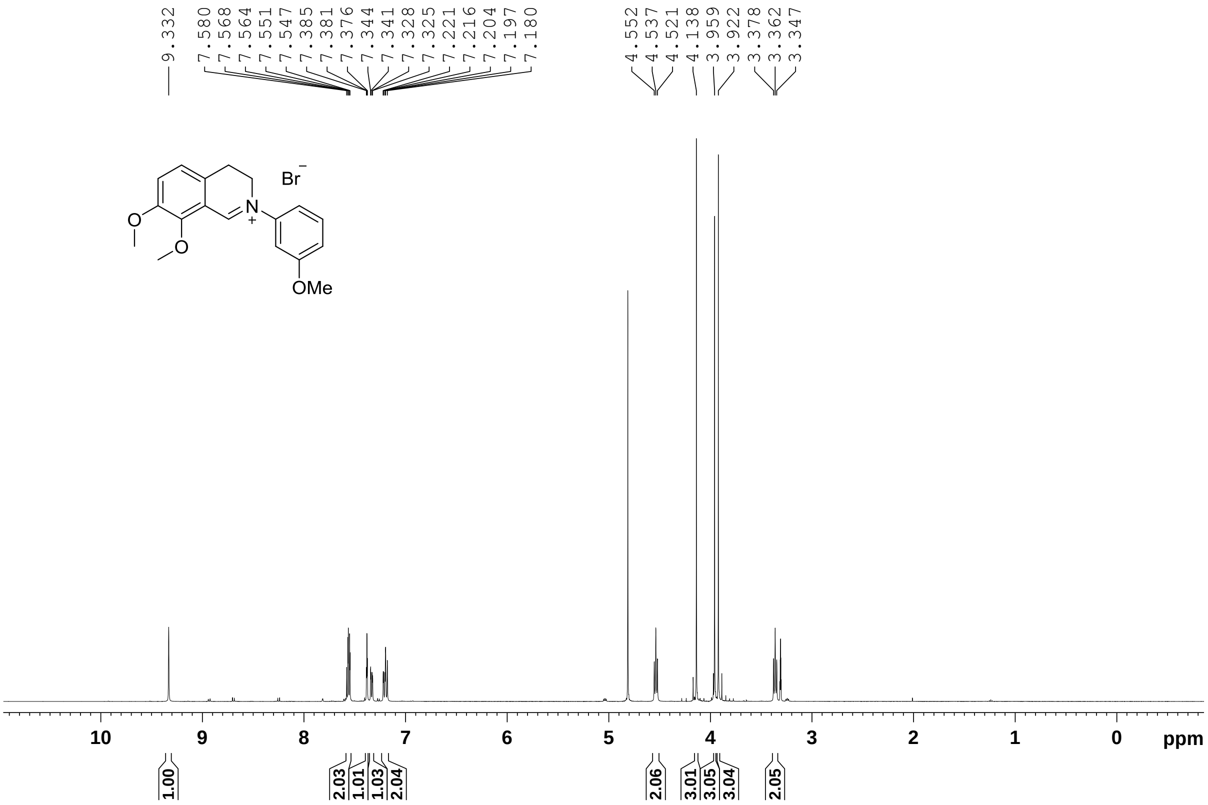

^{1}H NMR of compound **A24**

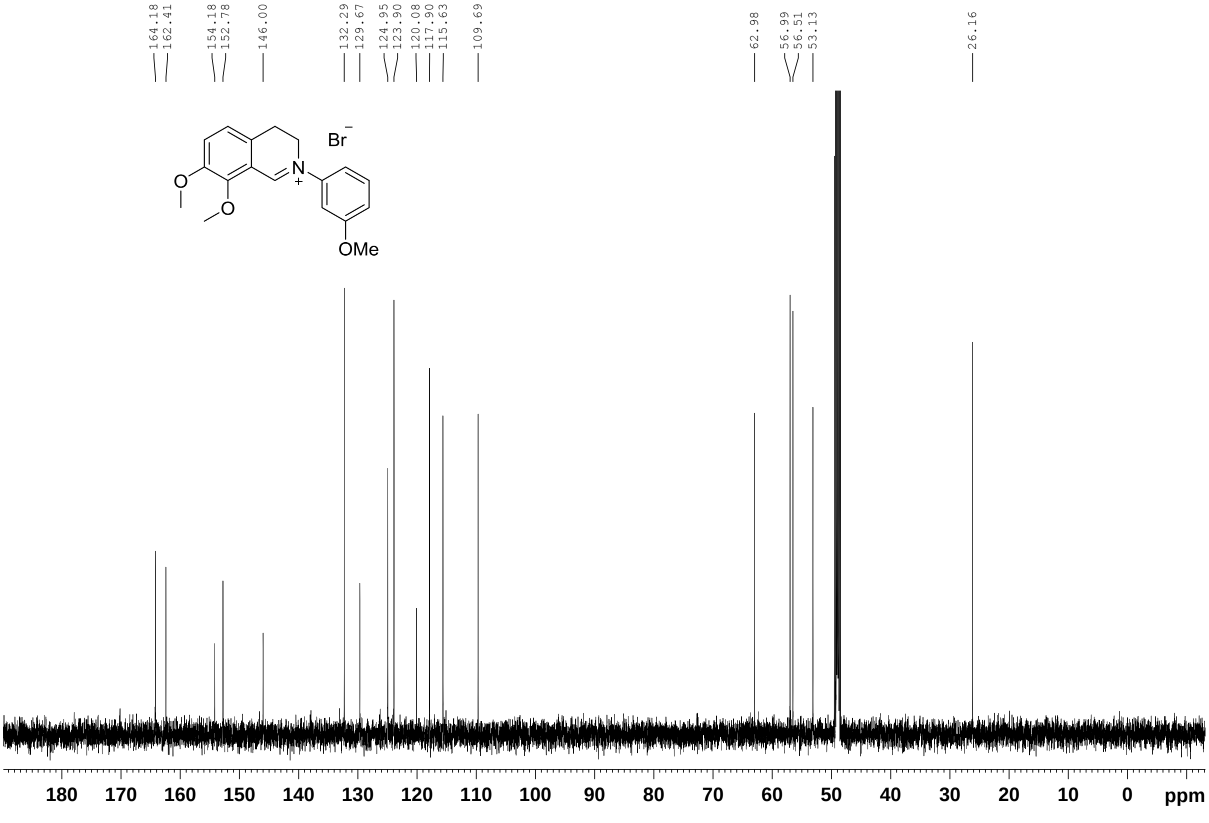

^{13}C NMR of compound **A24**

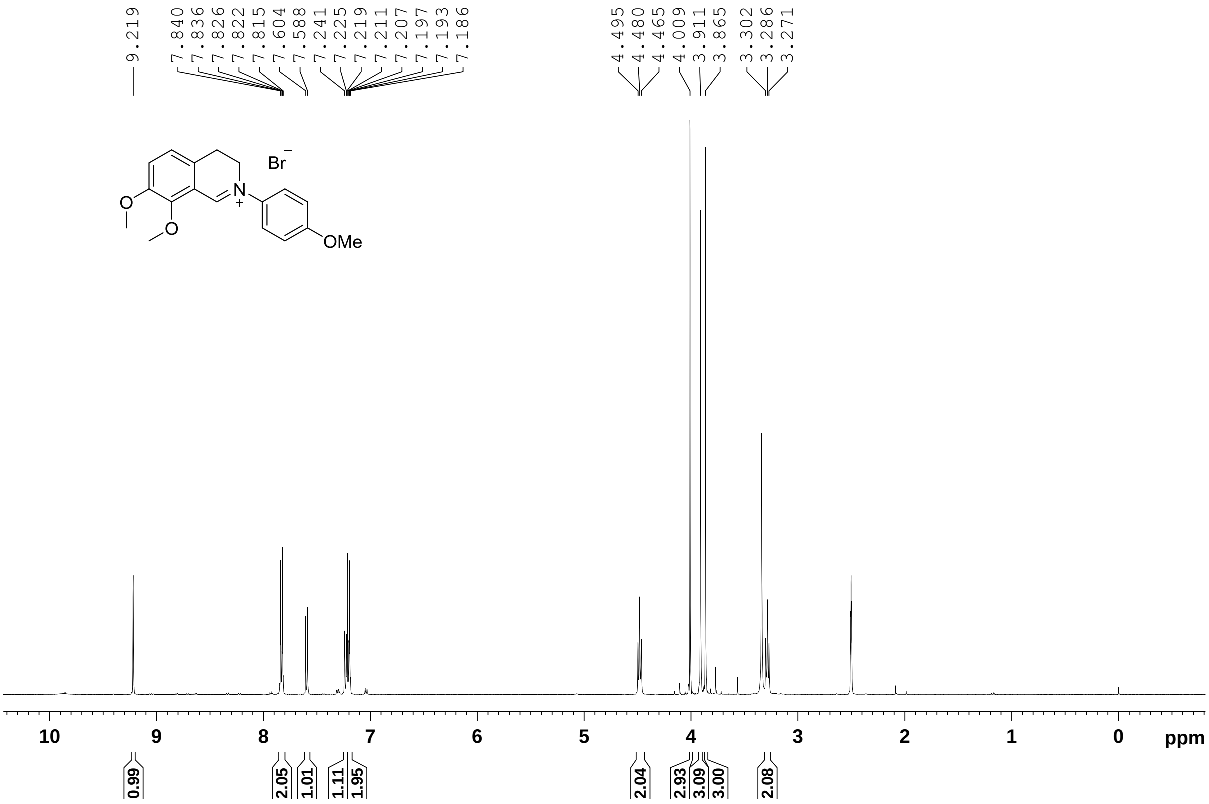

^{1}H NMR of compound **A25**

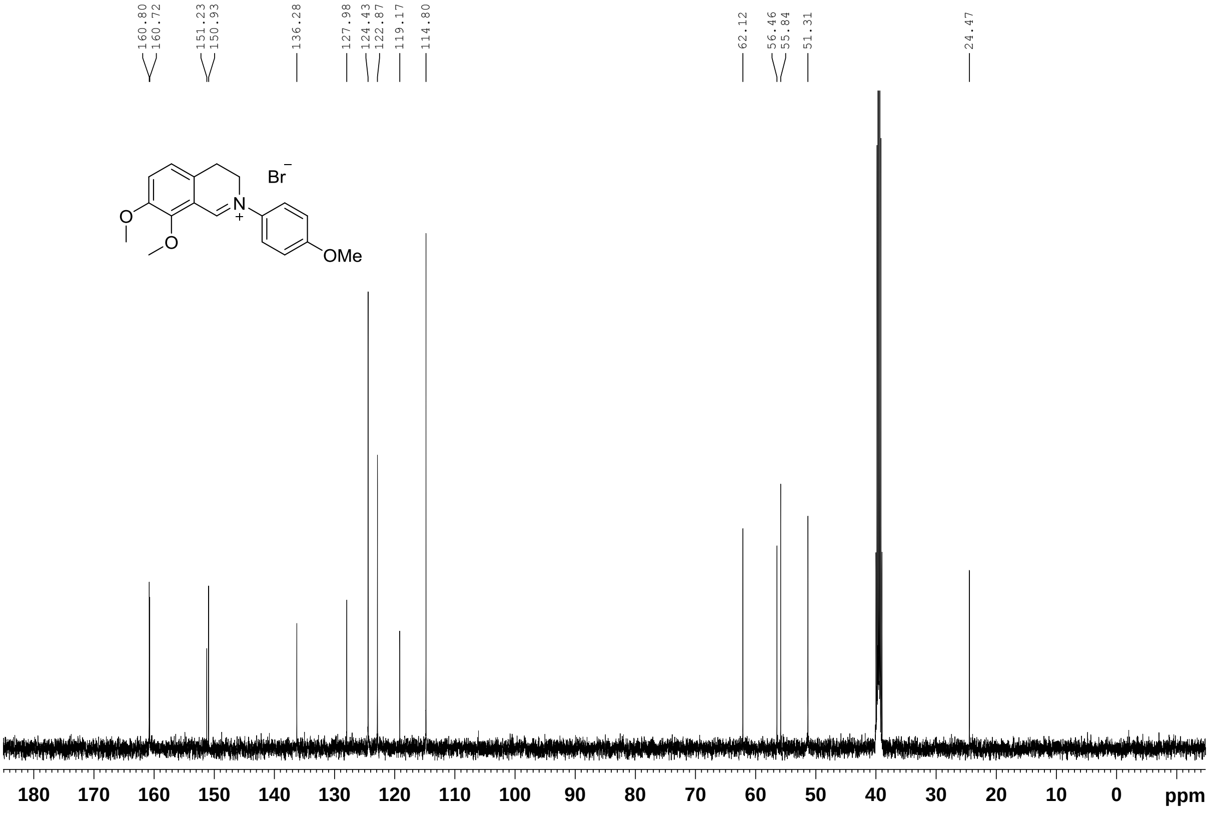

^{13}C NMR of compound **A25**

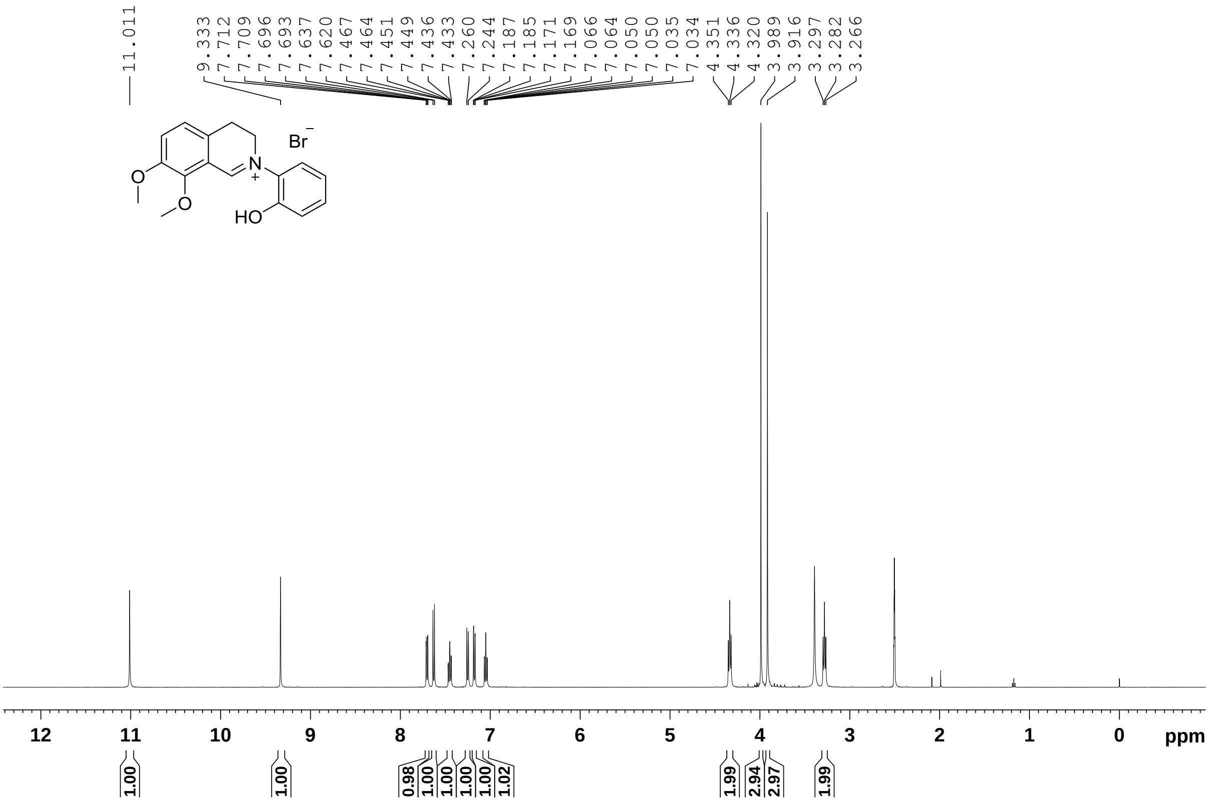

^{1}H NMR of compound **A26**

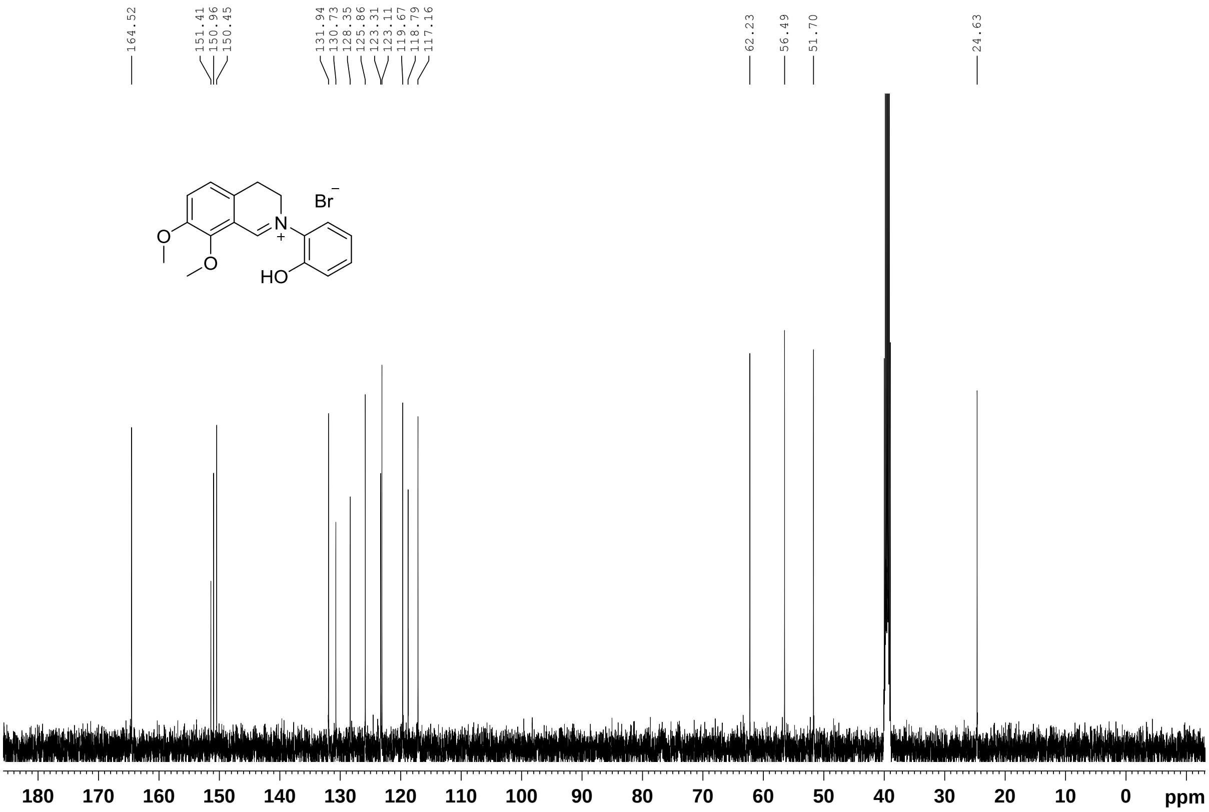

^{13}C NMR of compound **A26**

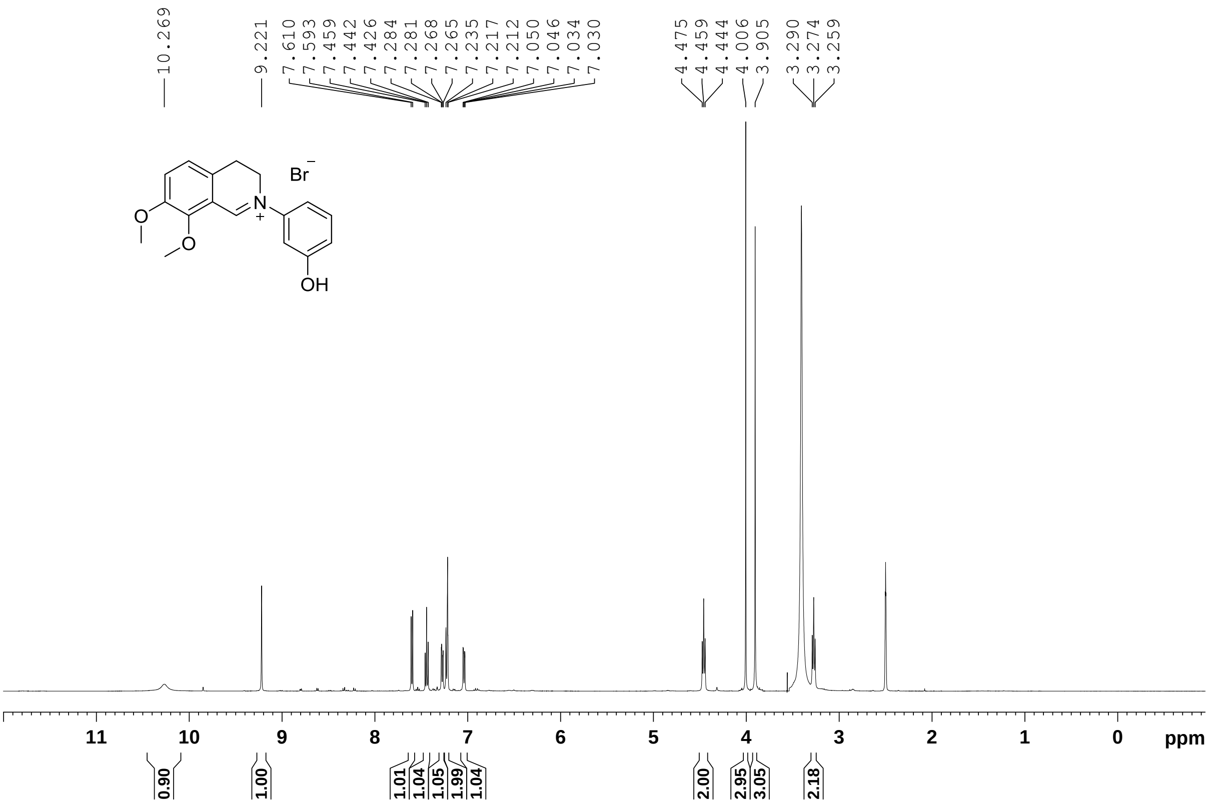

^{1}H NMR of compound **A27**

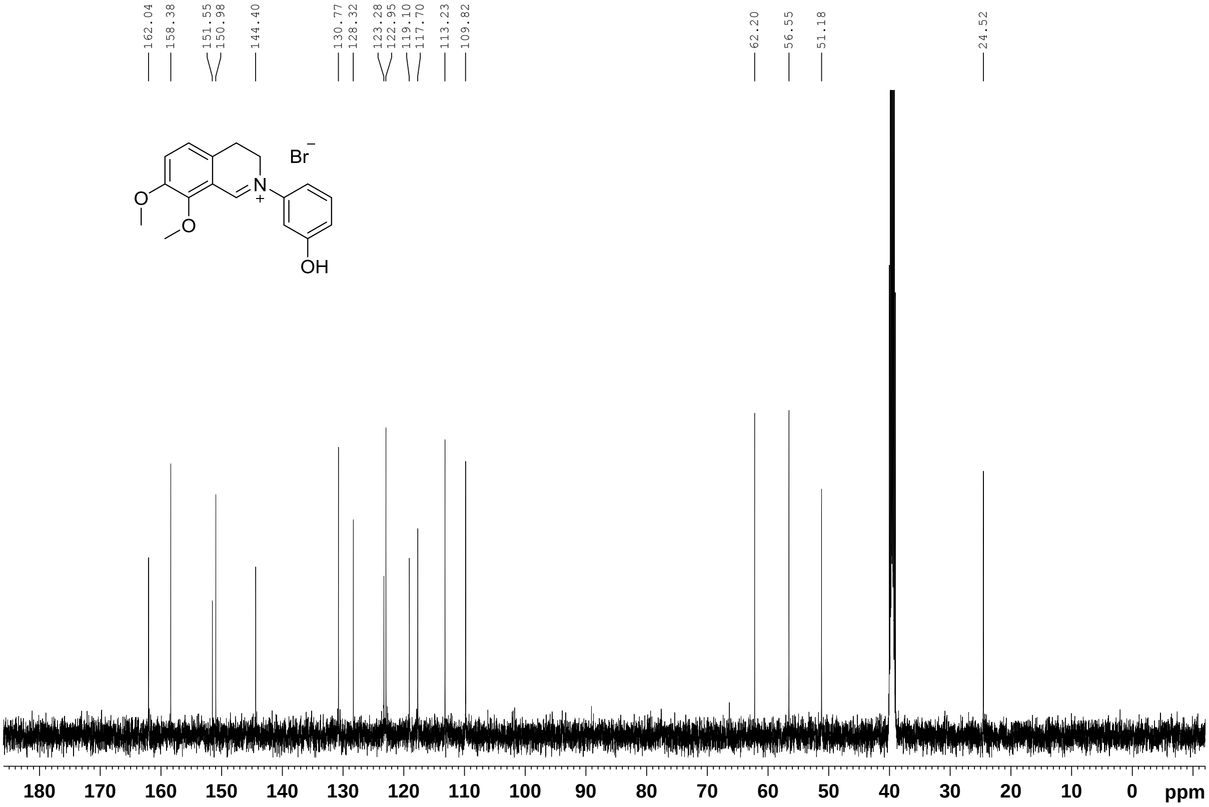

^{13}C NMR of compound **A27**

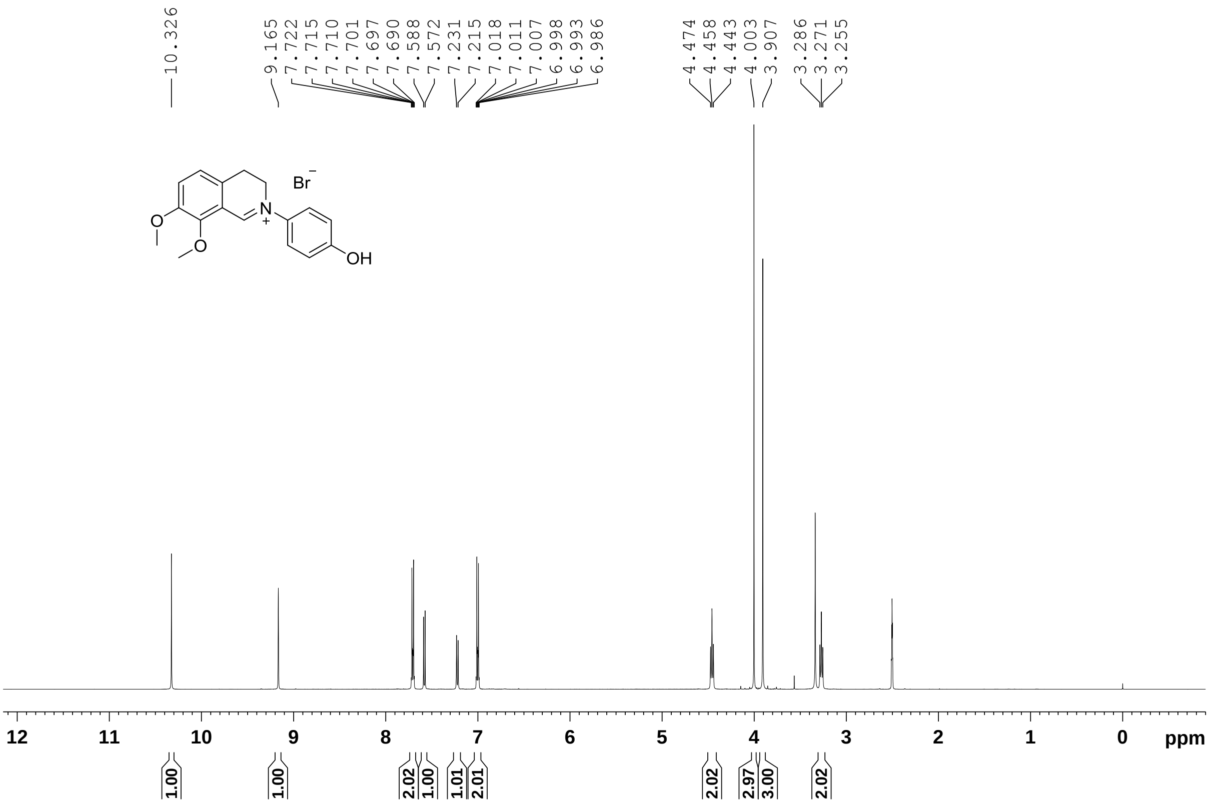

^{1}H NMR of compound **A28**

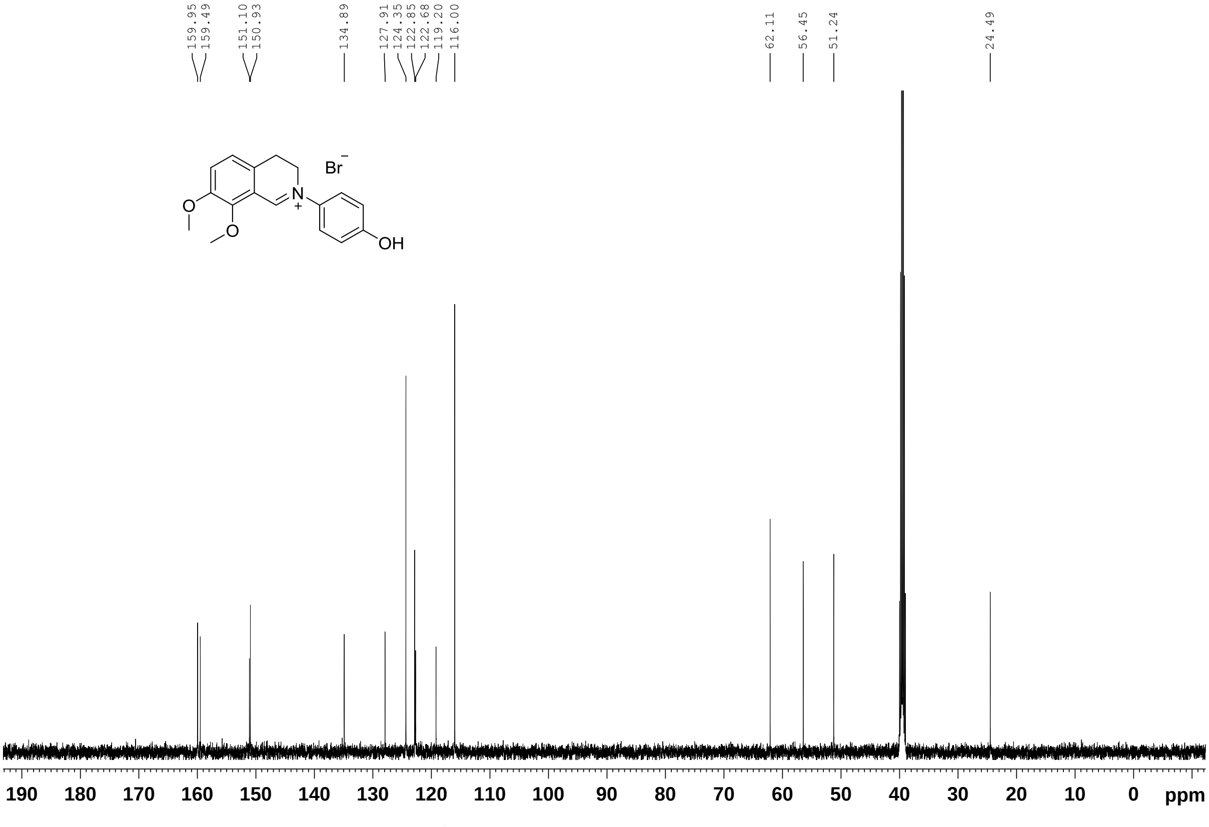

^{13}C NMR of compound **A28**

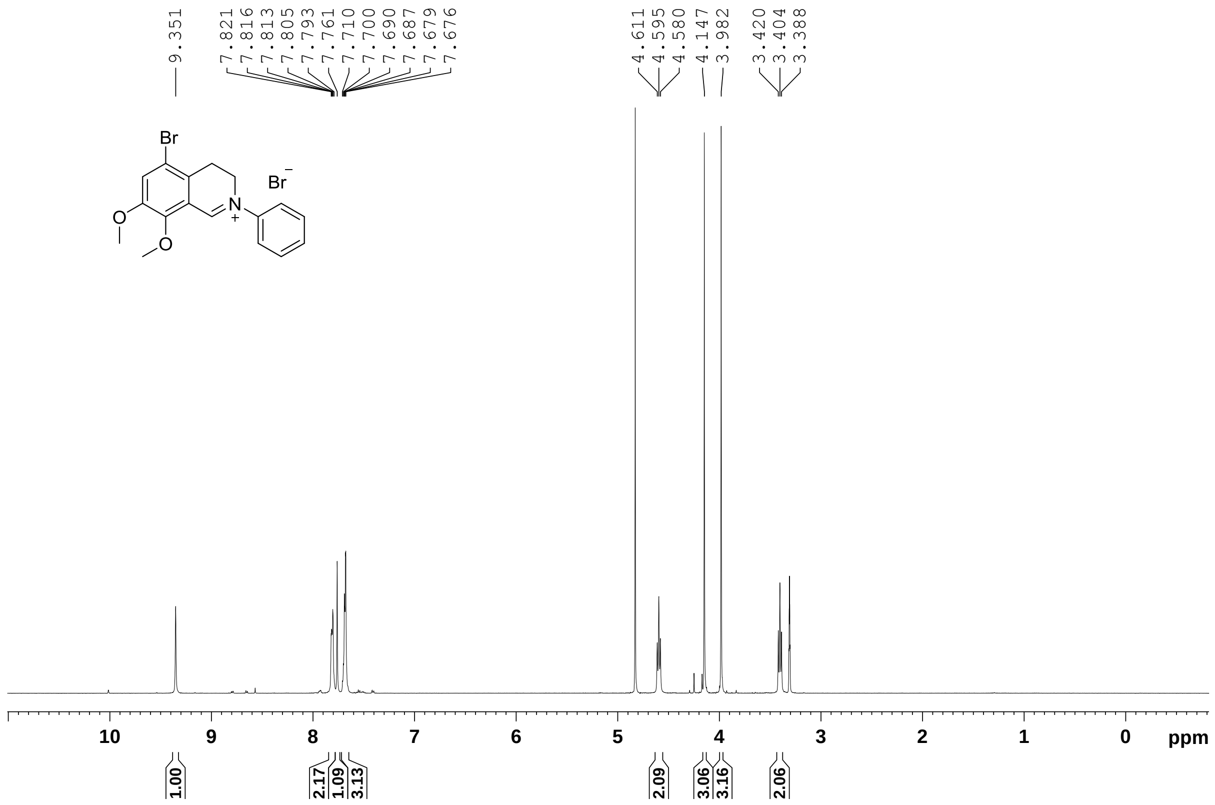

^{1}H NMR of compound **B1**

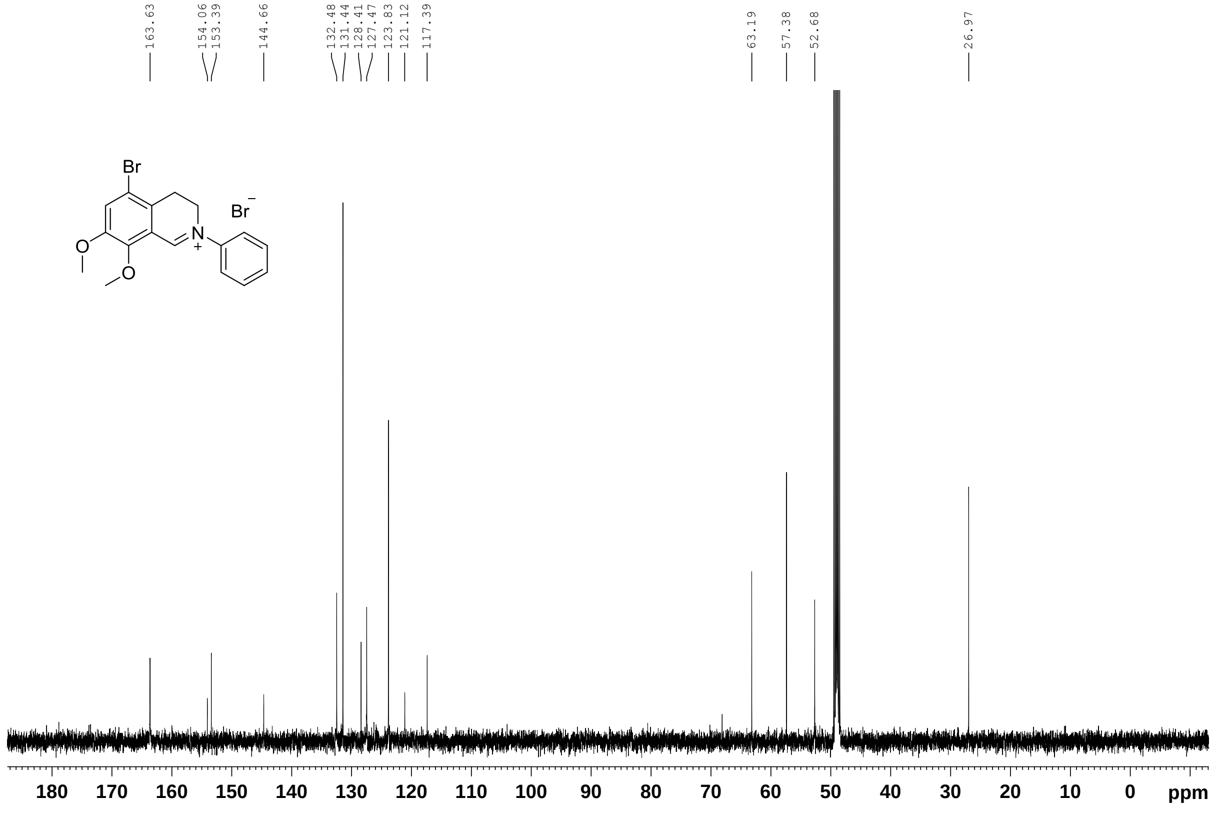

^{13}C NMR of compound **B1**

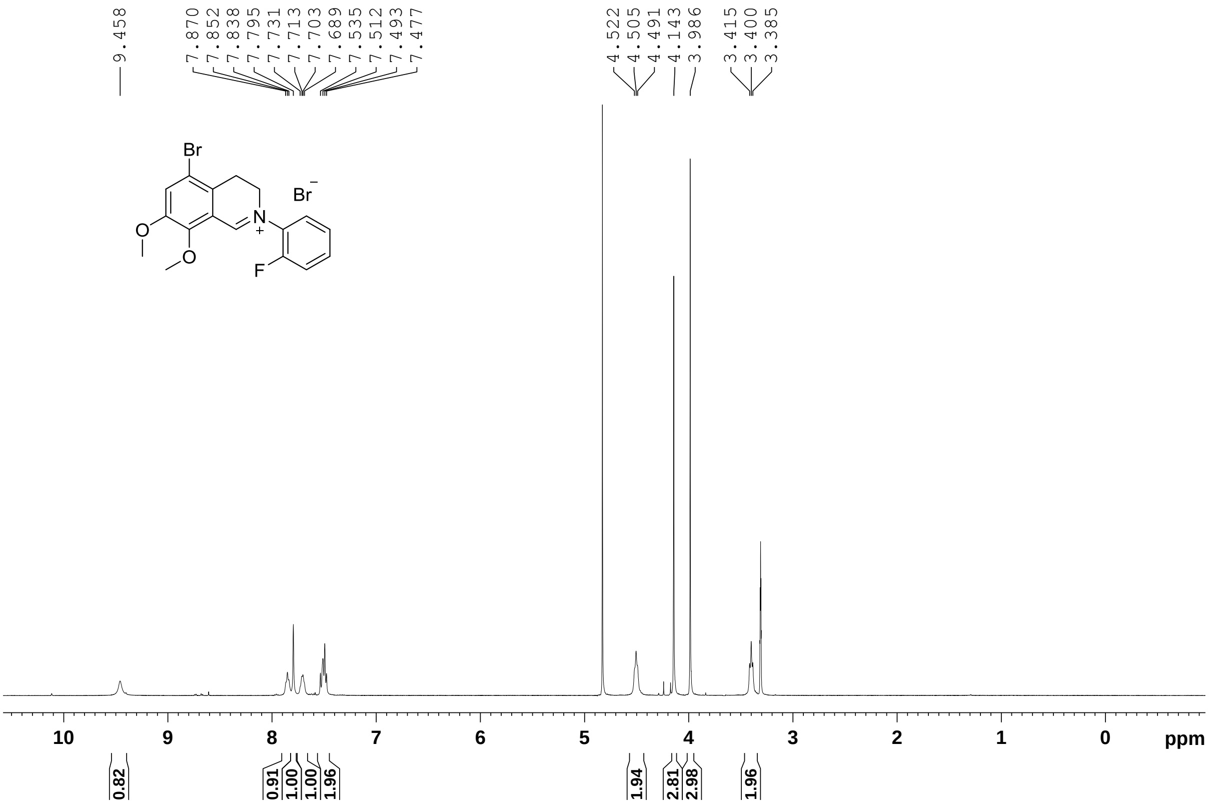

^{1}H NMR of compound **B2**

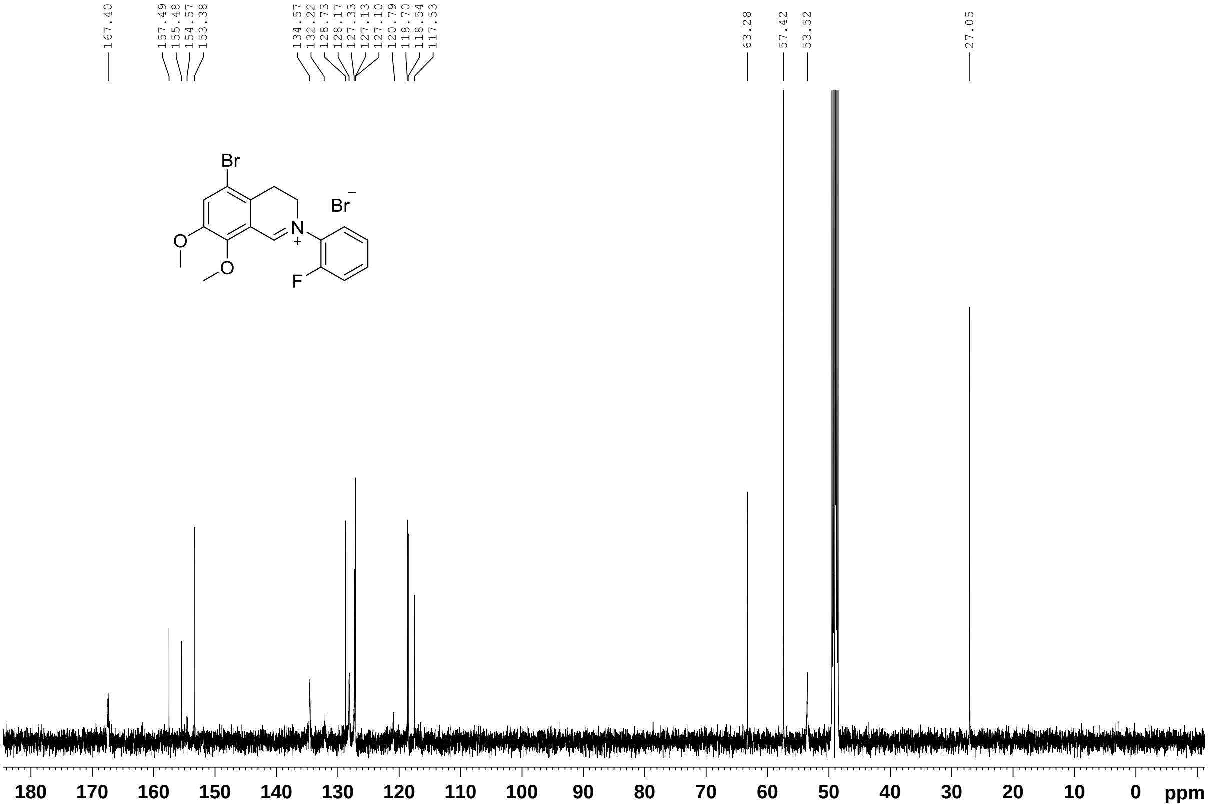

^{13}C NMR of compound **B2**

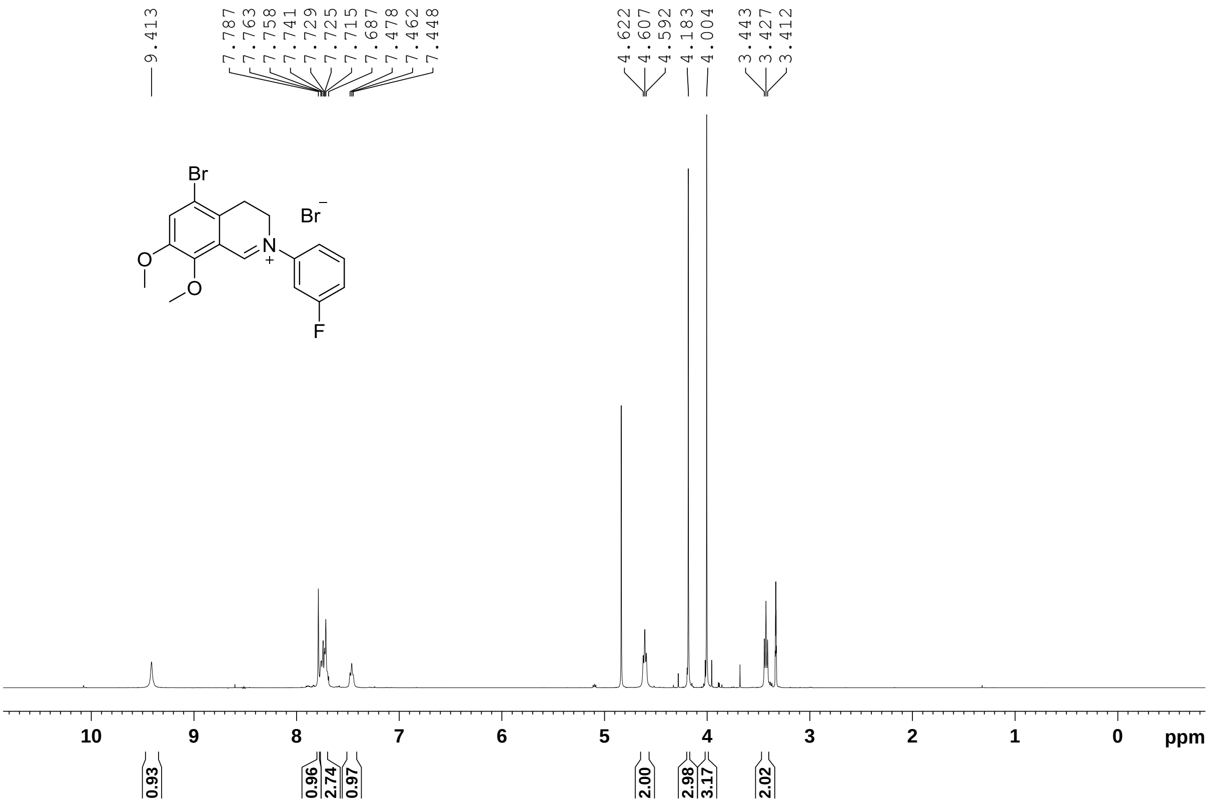

^{1}H NMR of compound **B3**

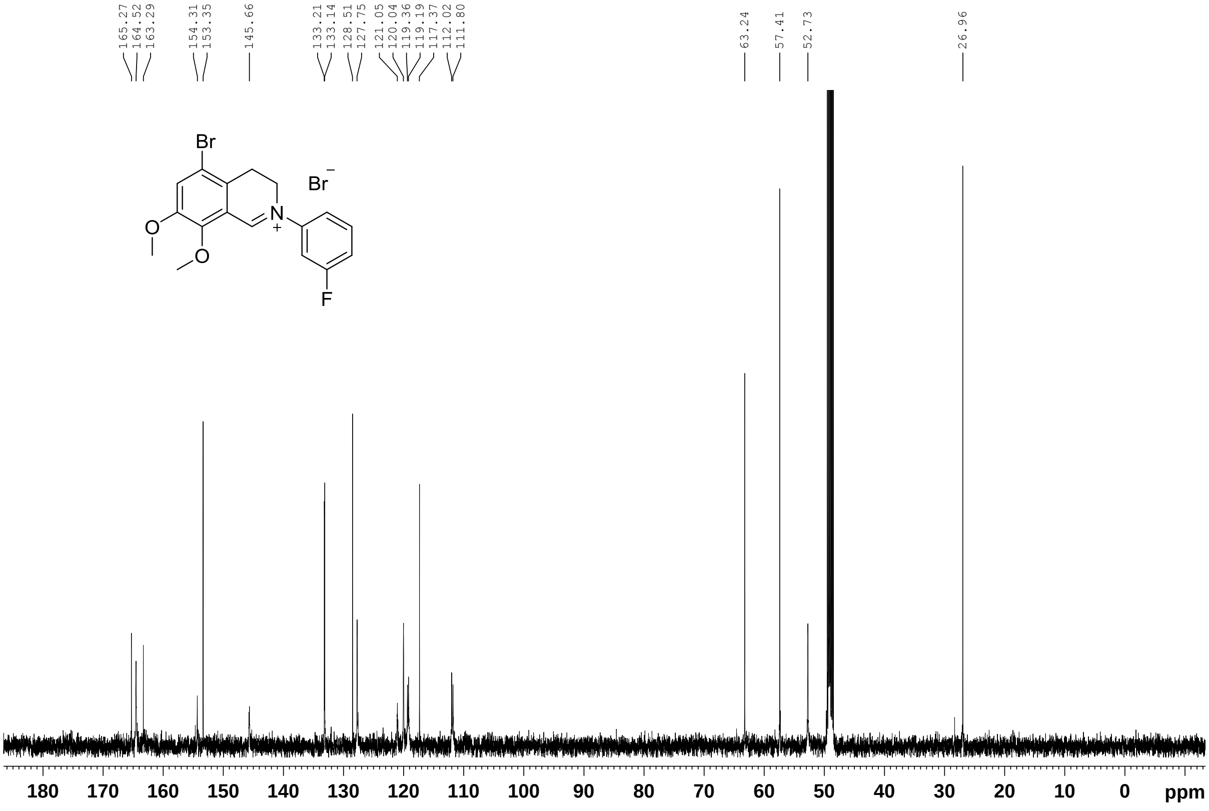

^{13}C NMR of compound **B3**

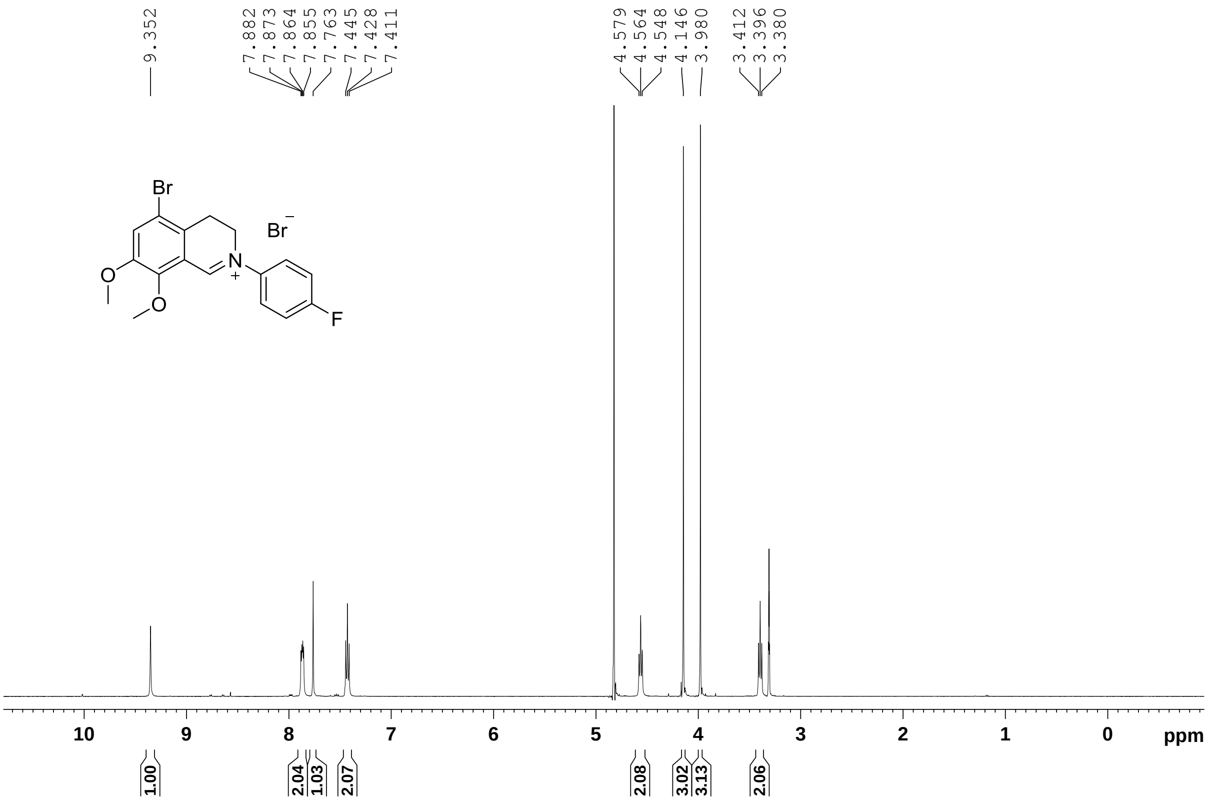

^{1}H NMR of compound **B4**

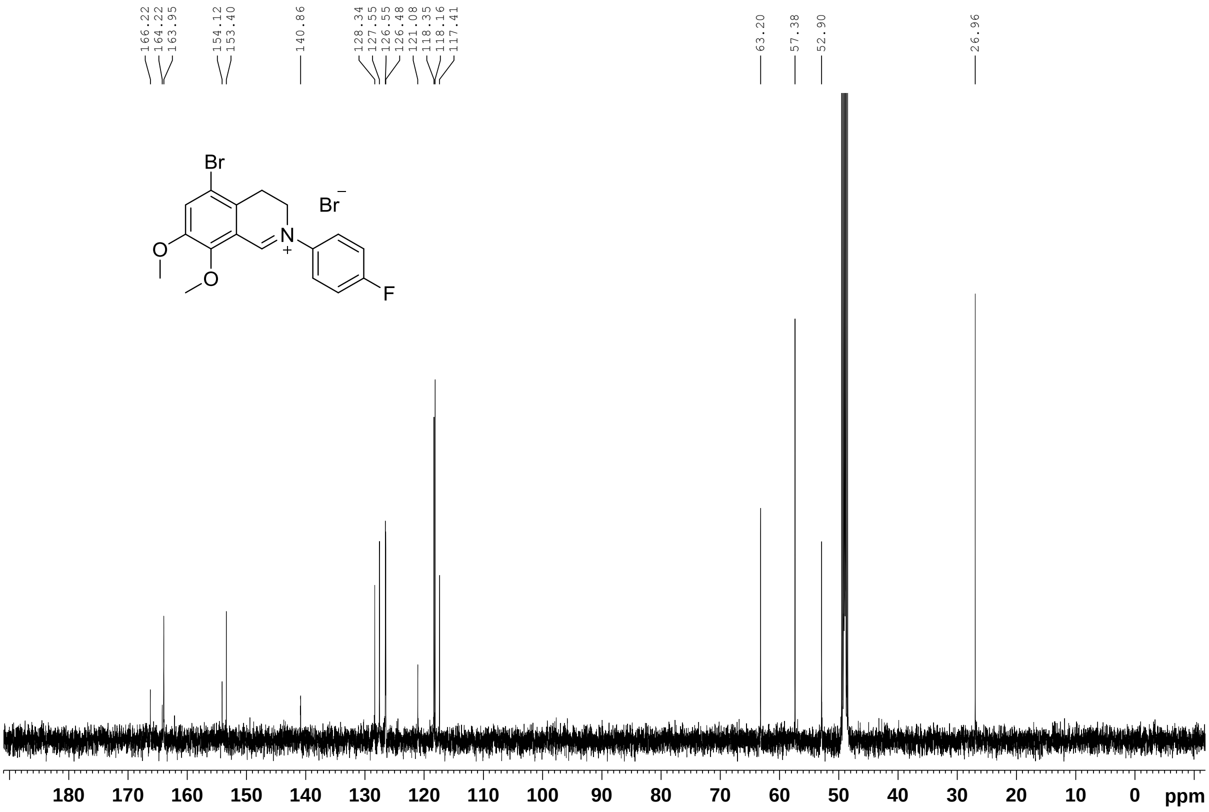

^{13}C NMR of compound **B4**

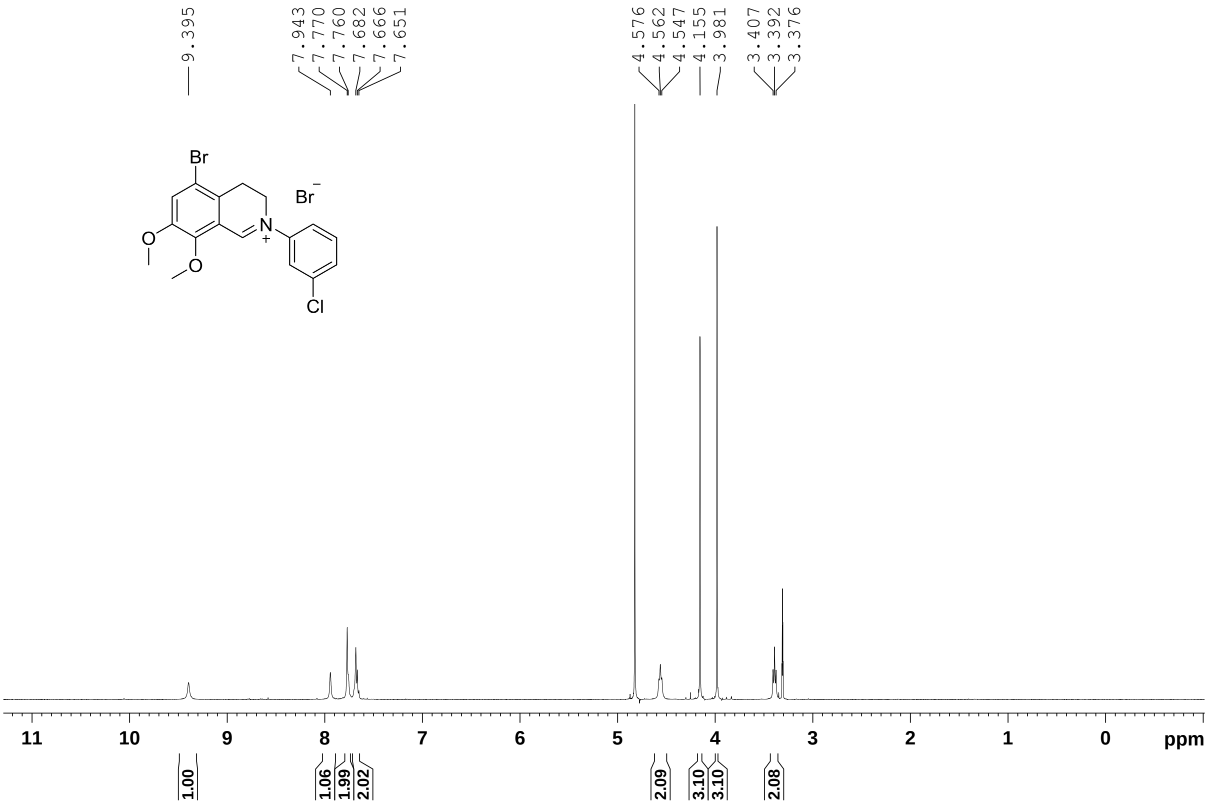

^{1}H NMR of compound **B5**

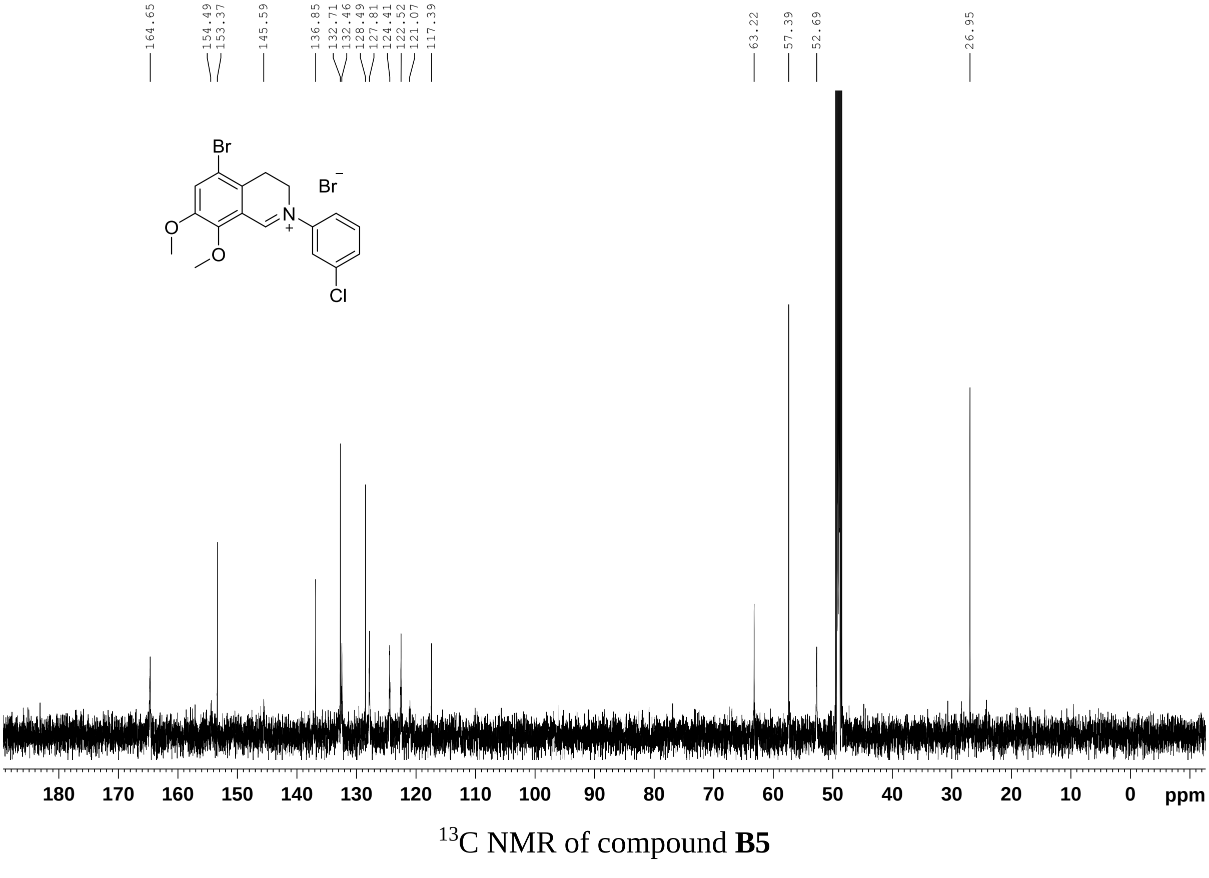

^{13}C NMR of compound **B5**

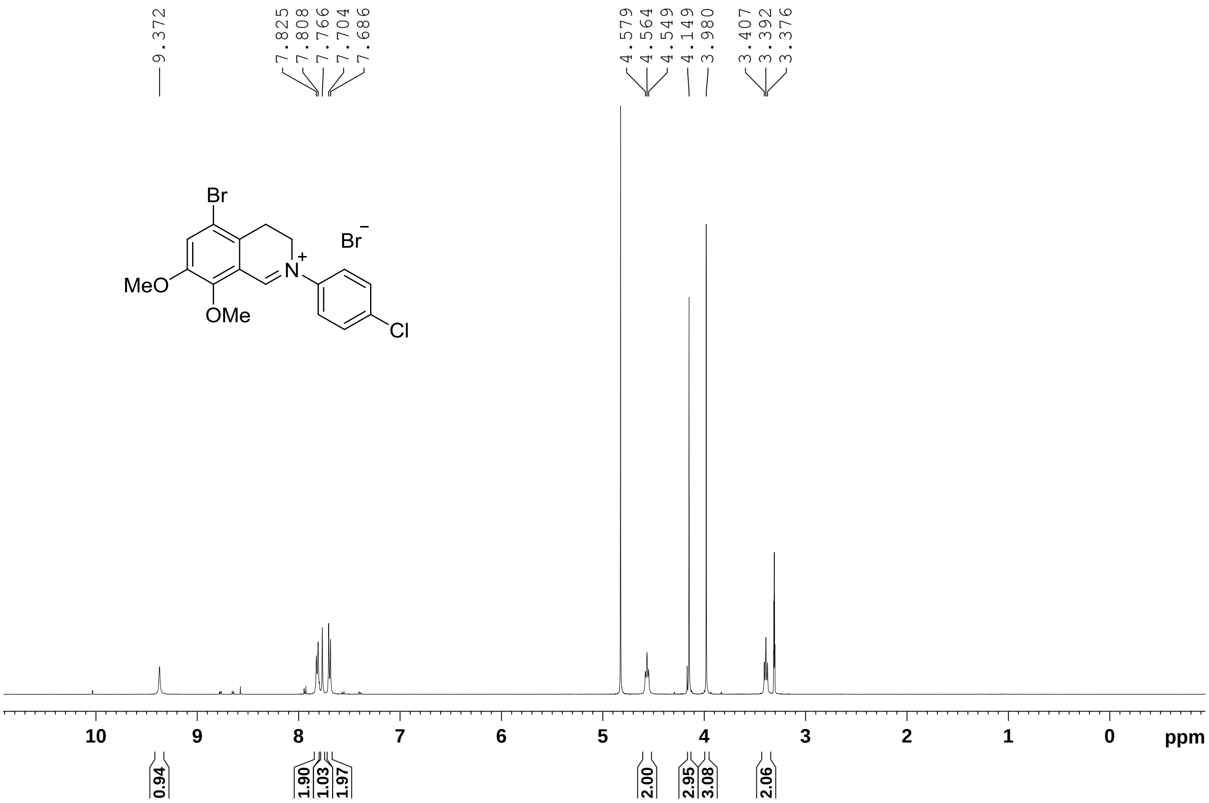

^{1}H NMR of compound **B6**

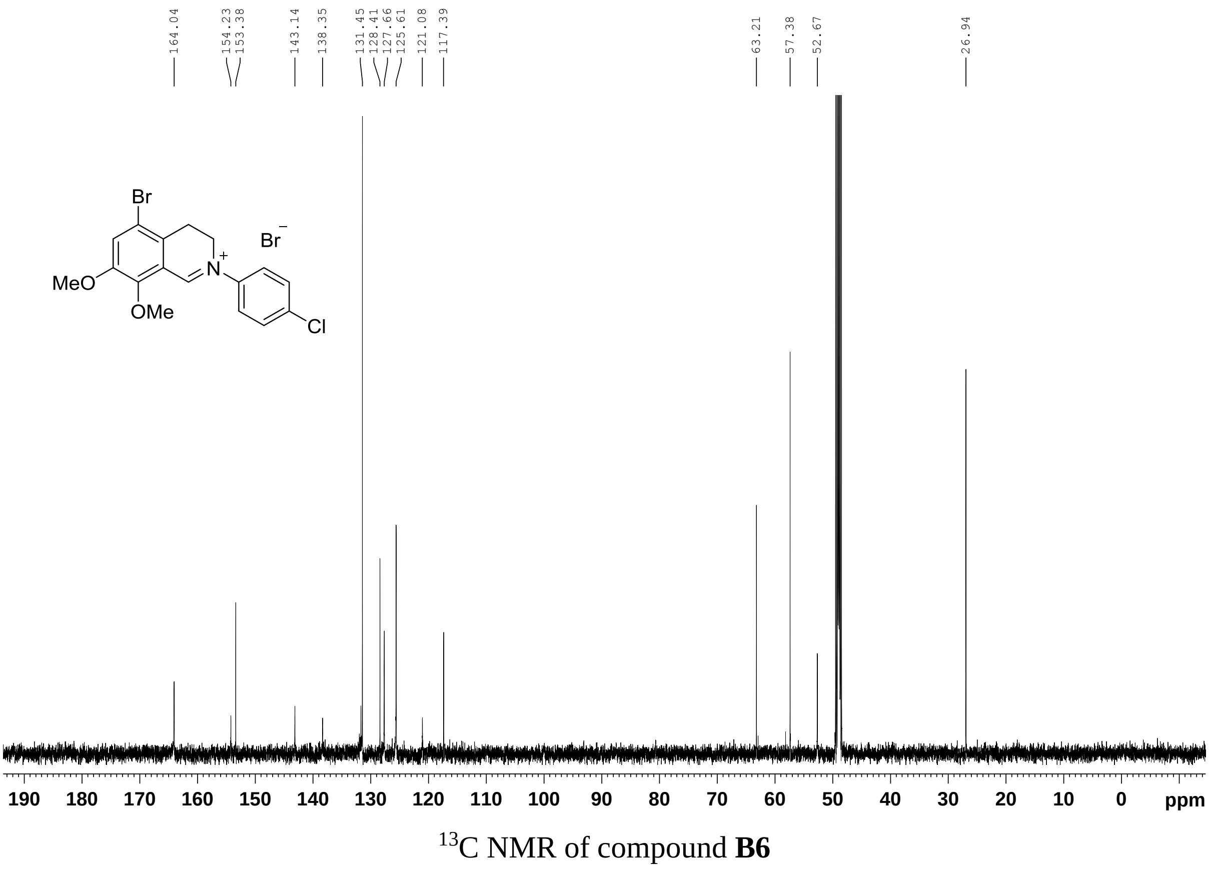

^{13}C NMR of compound **B6**

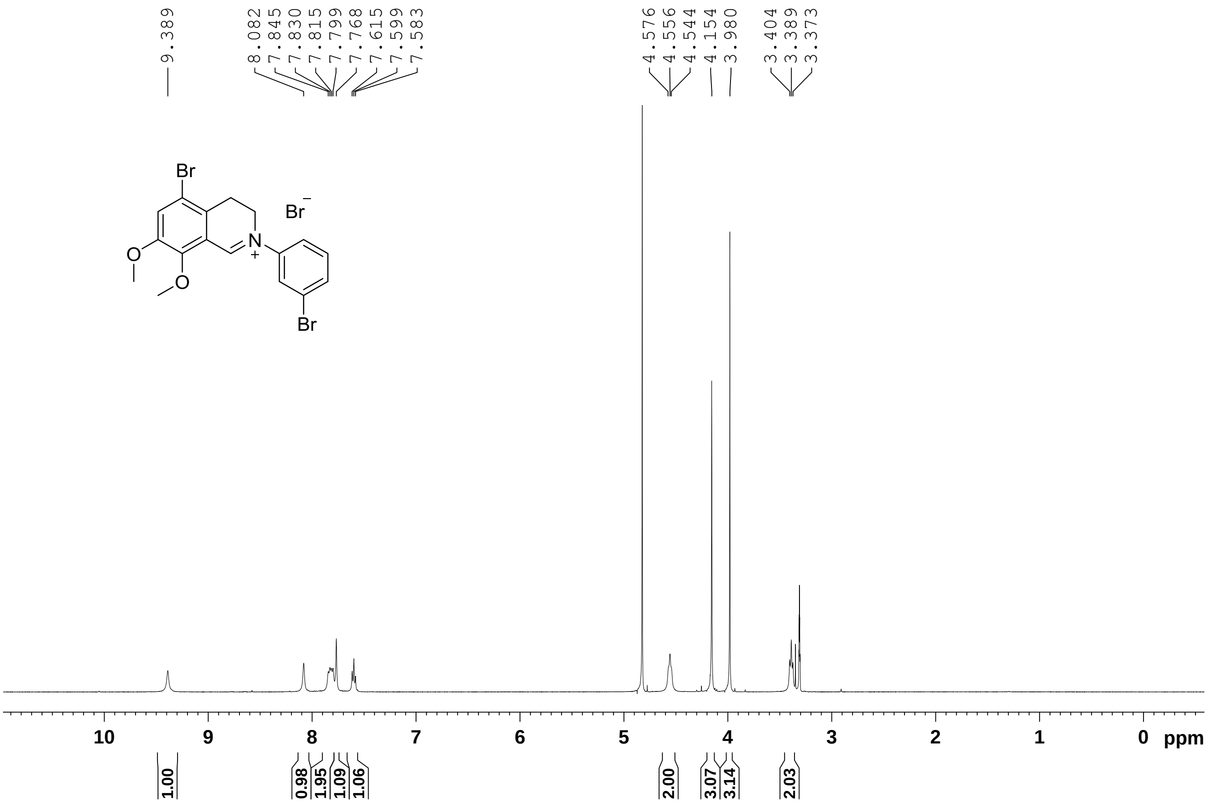

^{1}H NMR of compound **B7**

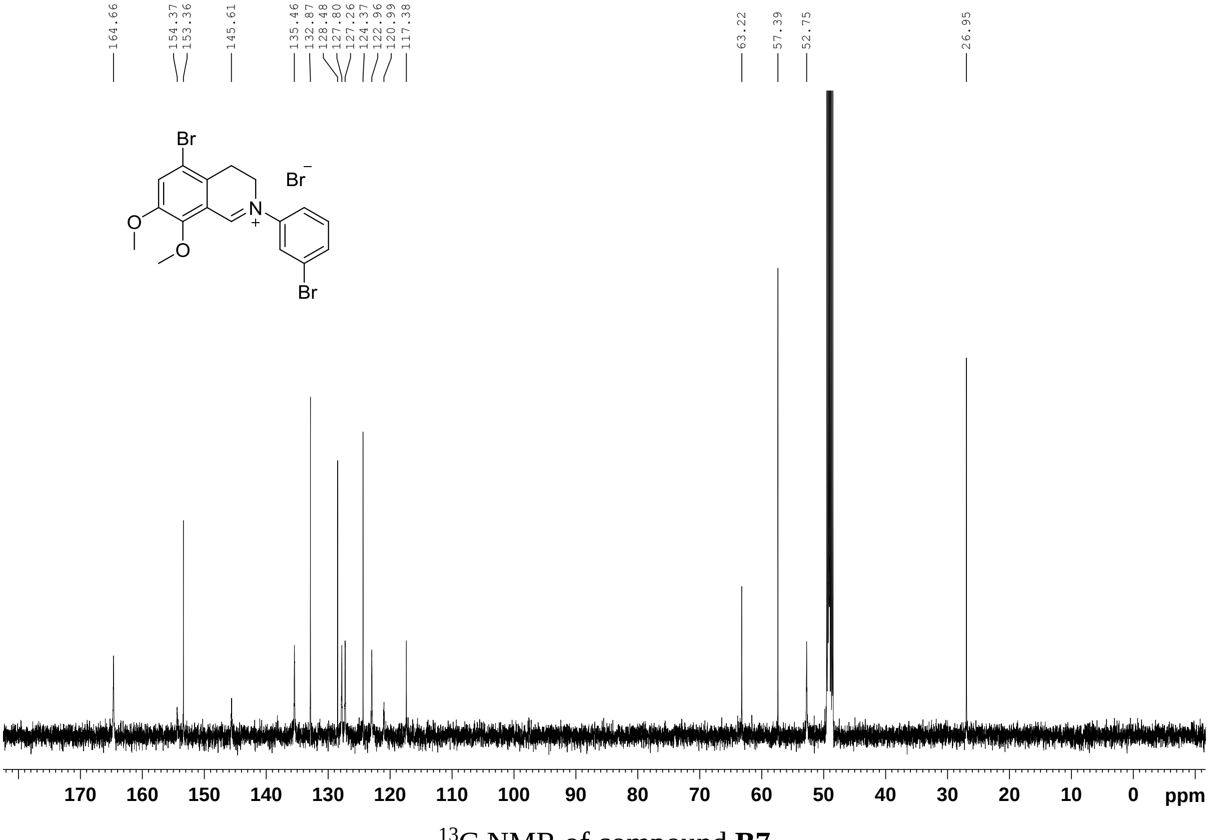

^{13}C NMR of compound **B7**

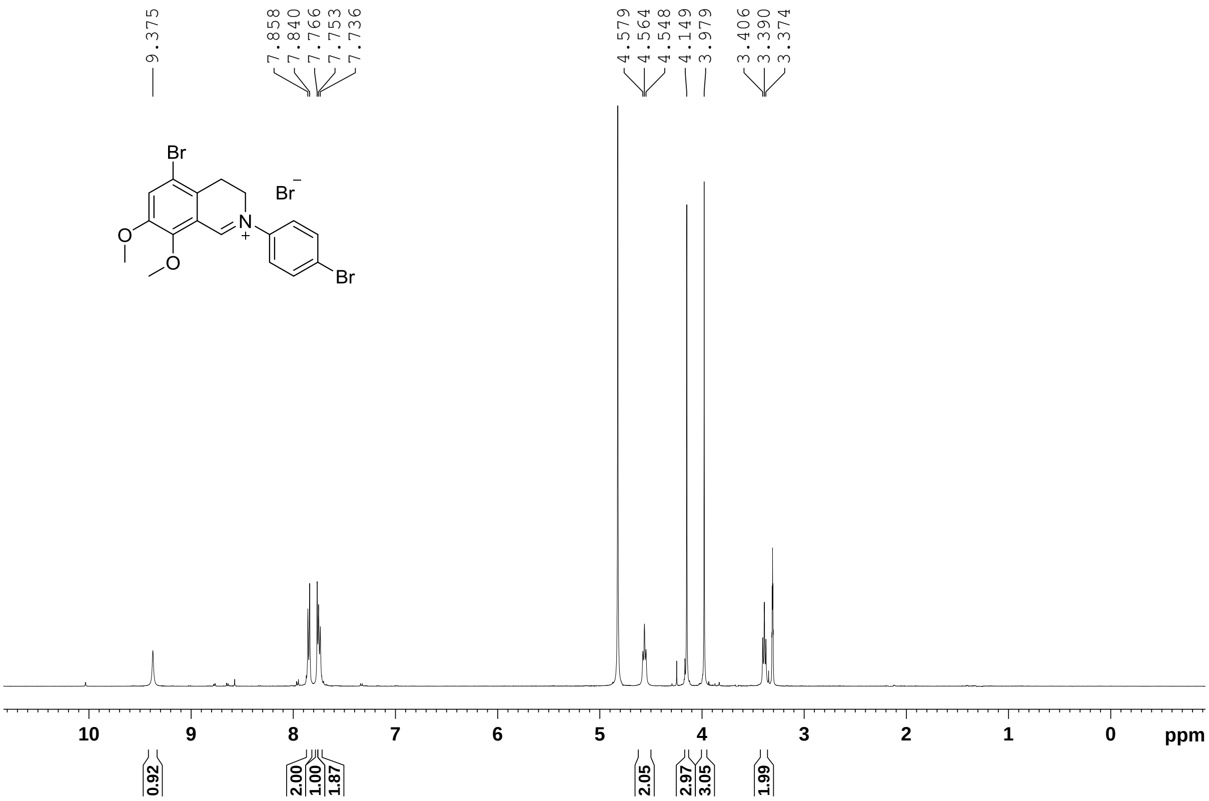

^{1}H NMR of compound **B8**

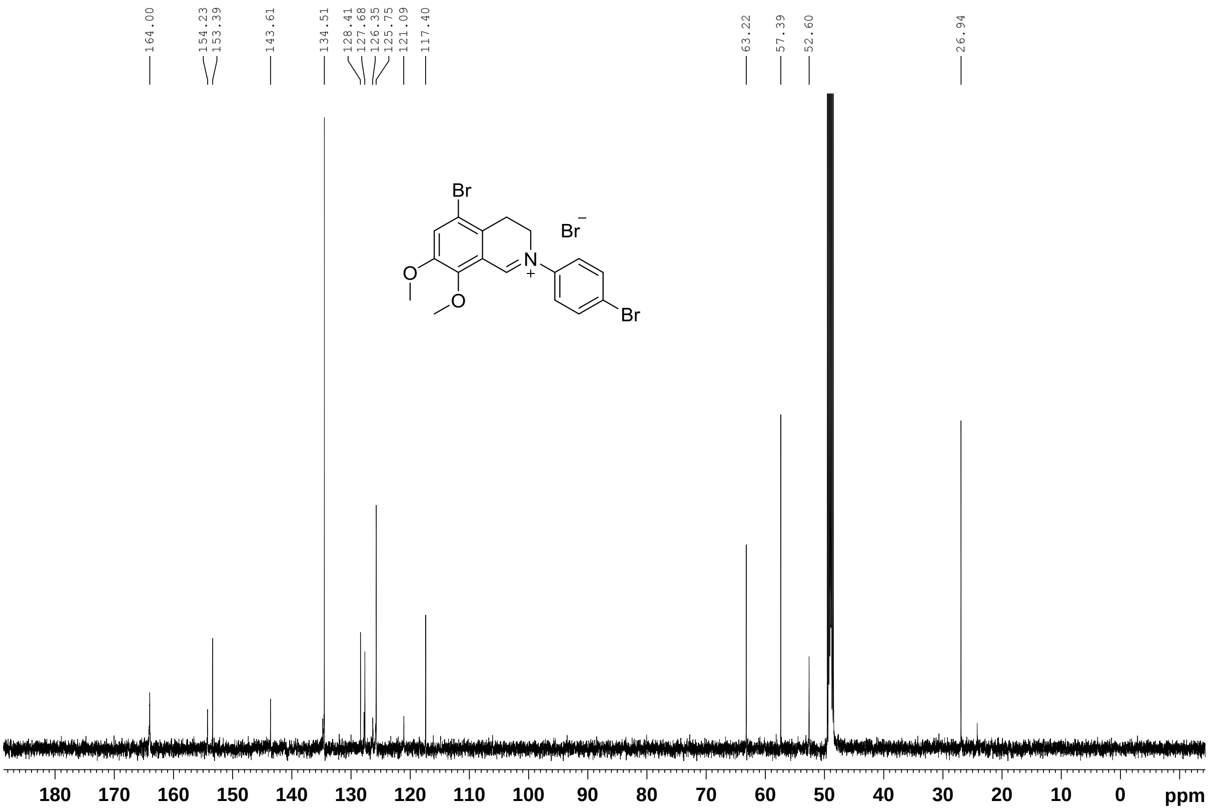

^{1}H NMR of compound **B8**

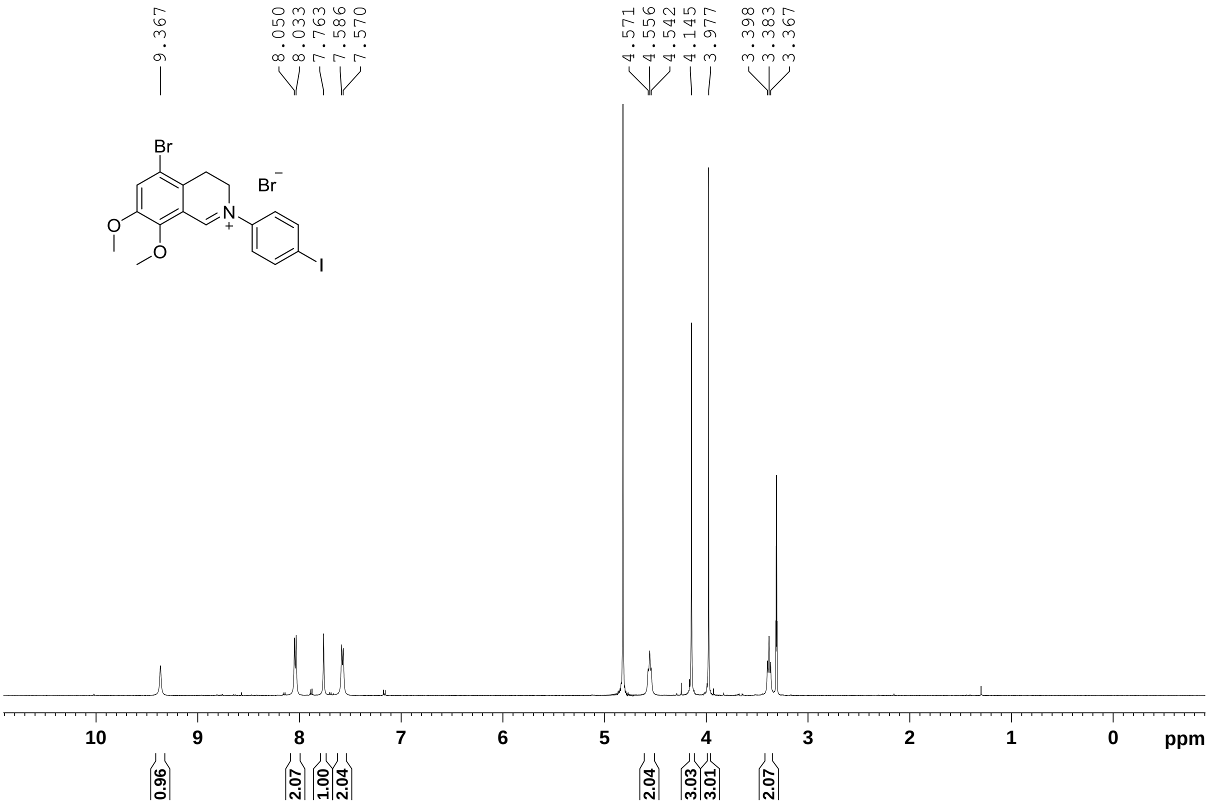

^{1}H NMR of compound **B9**

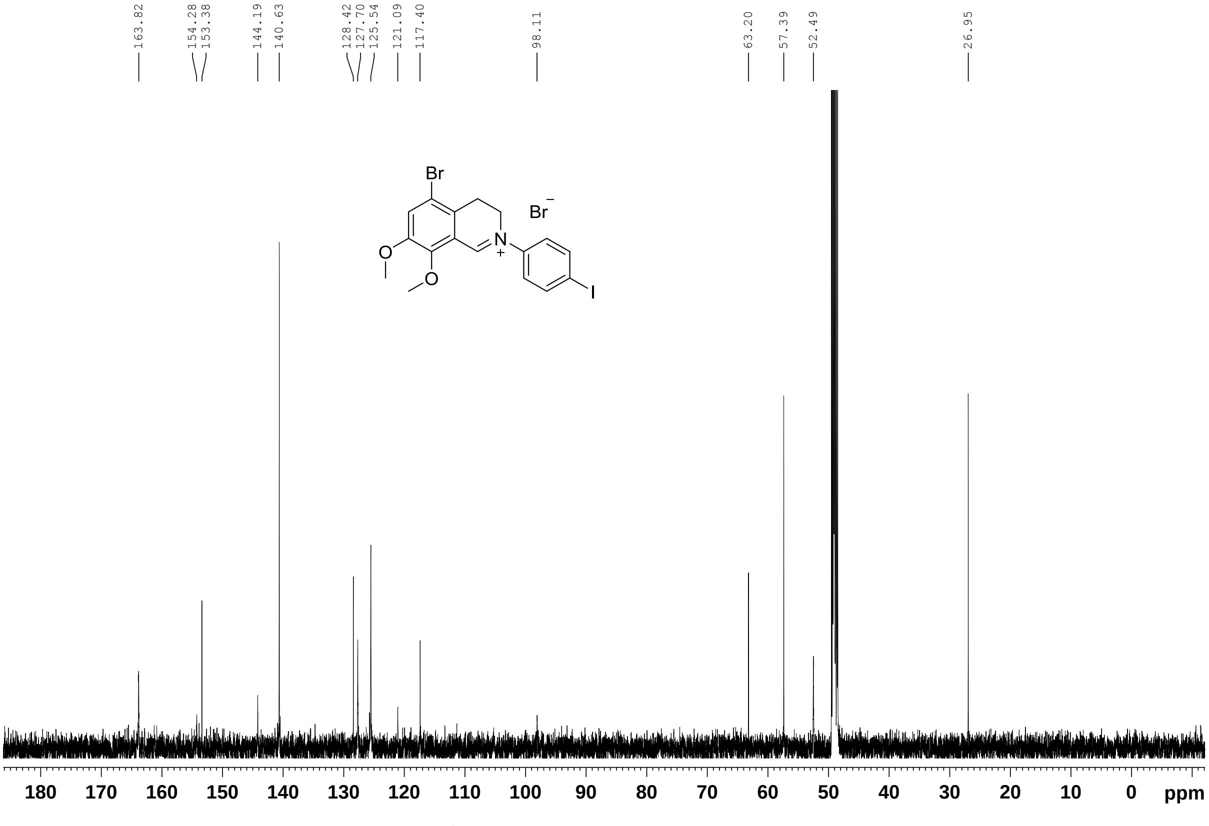

^{13}C NMR of compound **B9**

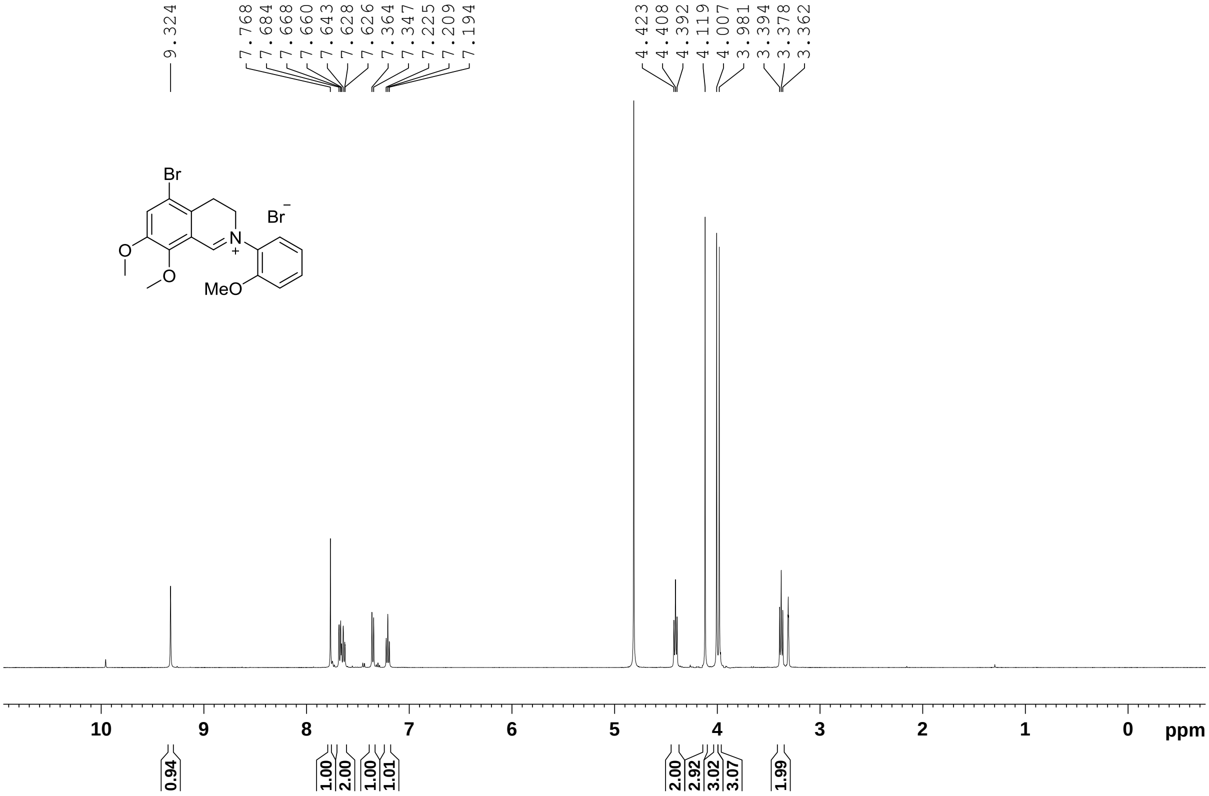

^{1}H NMR of compound **B10**

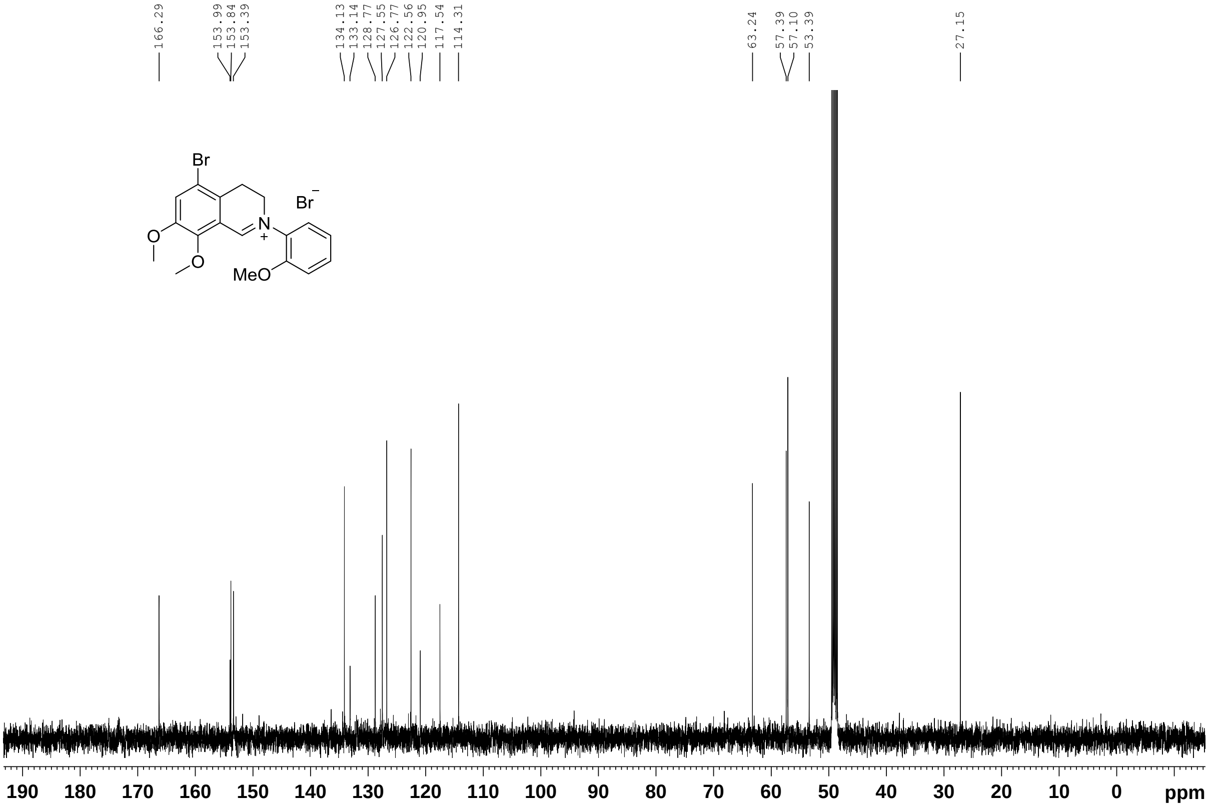

^{13}C NMR of compound **B10**

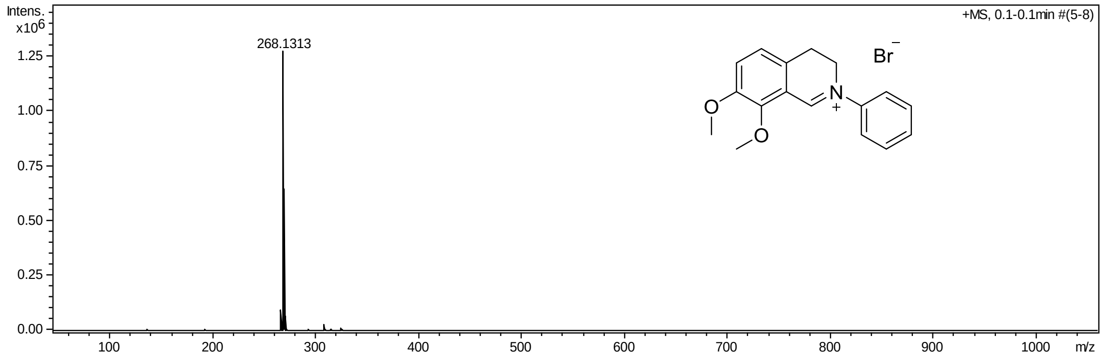

HR-ESI-MS of compound **A1**

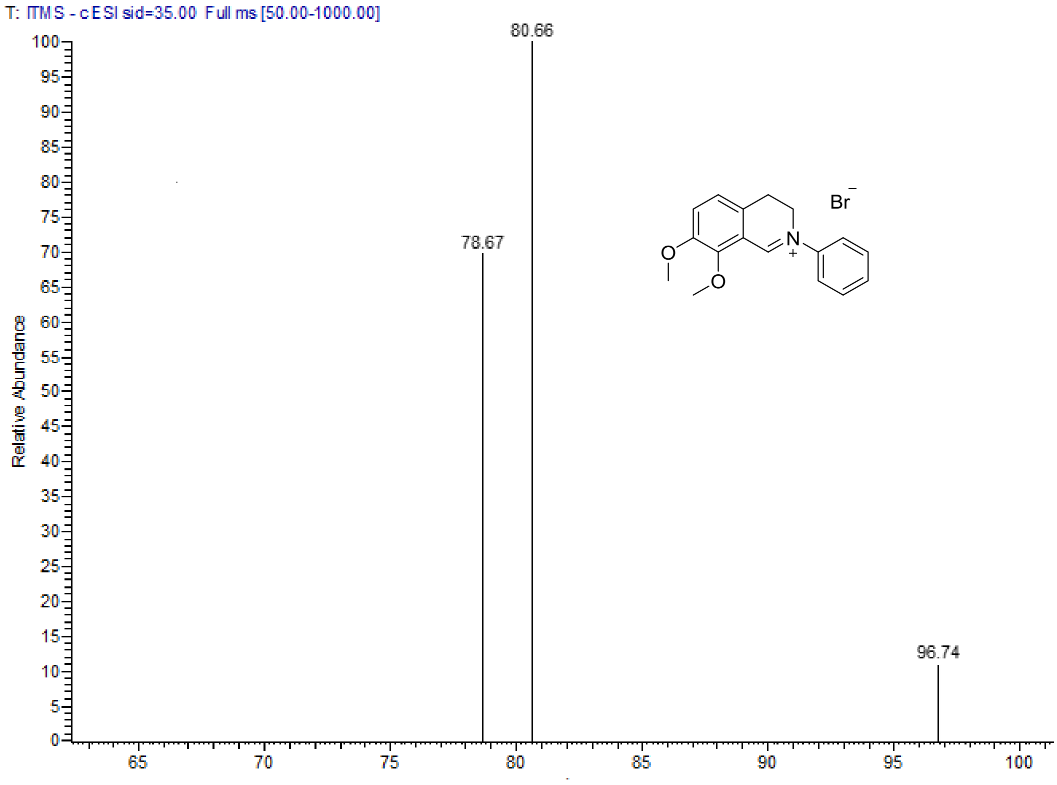

ESI-MS of compound **A1**

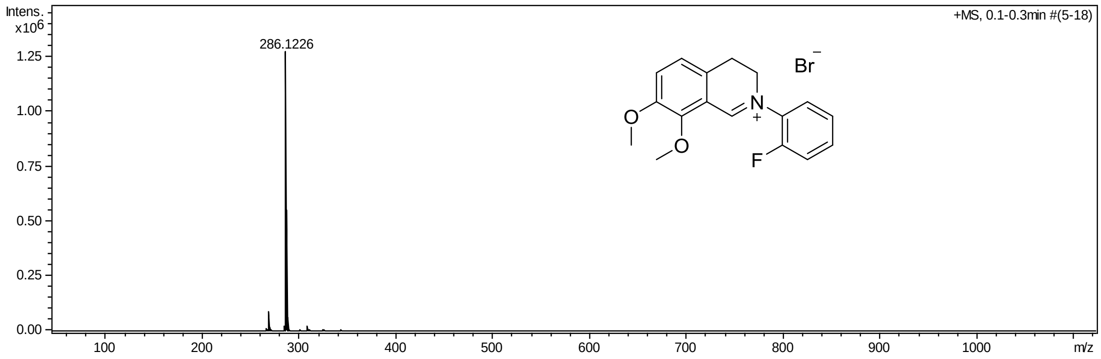

HR-ESI-MS of compound **A2**

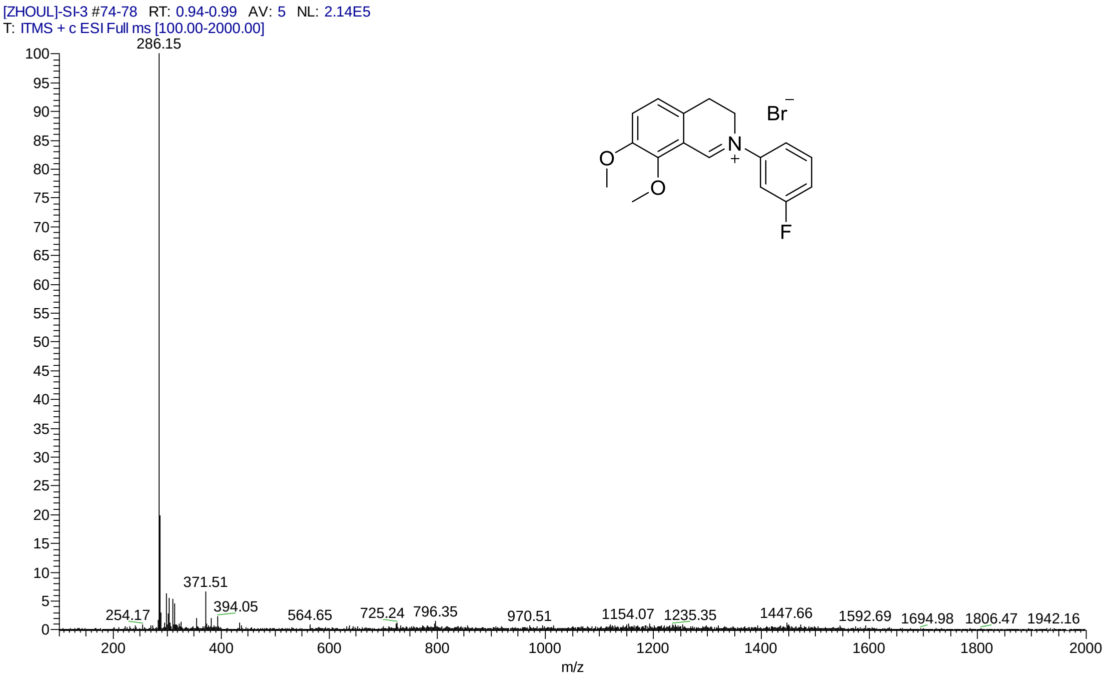

ESI-MS of compound **A3**

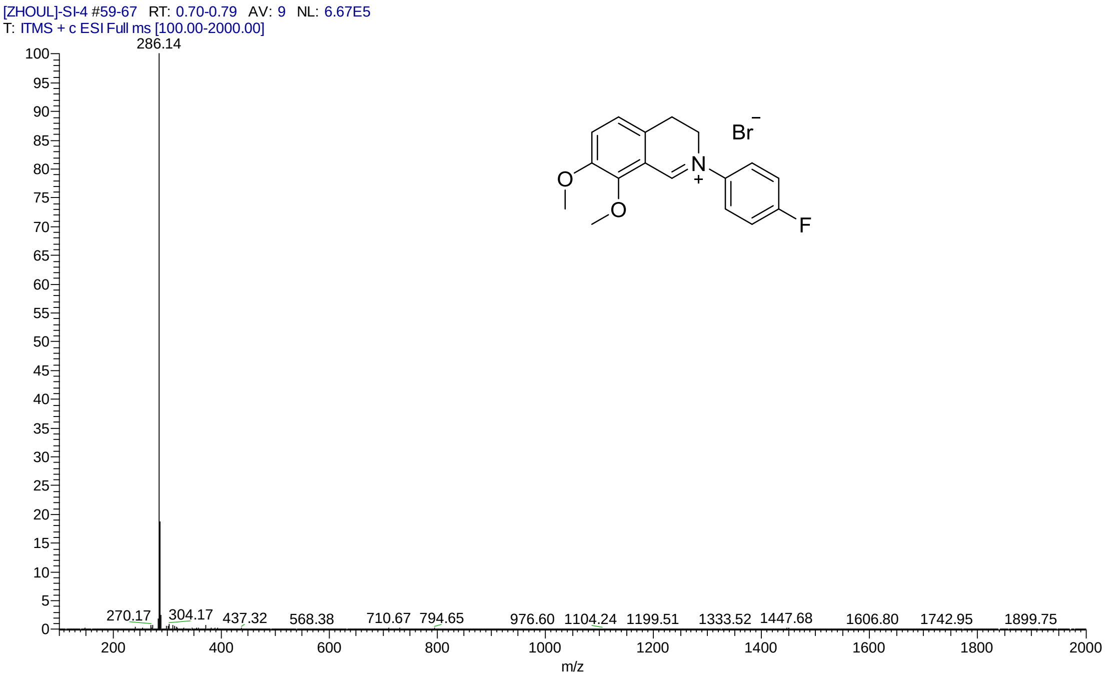

ESI-MS of compound **A4**

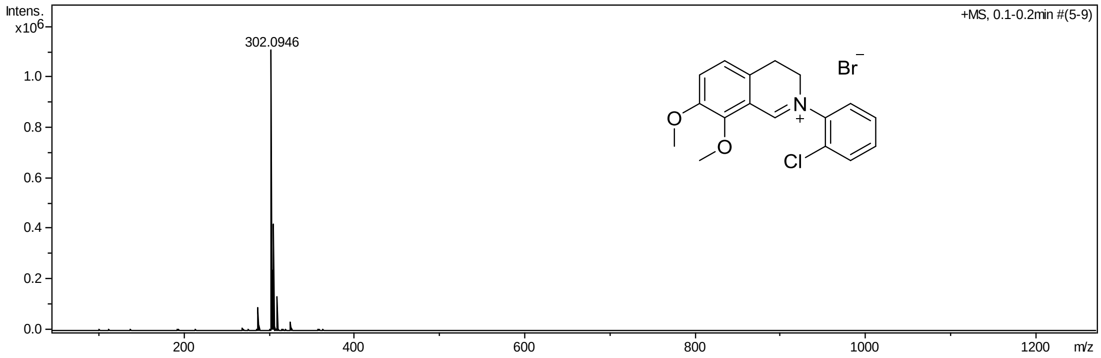

ESI-MS of compound **A5**

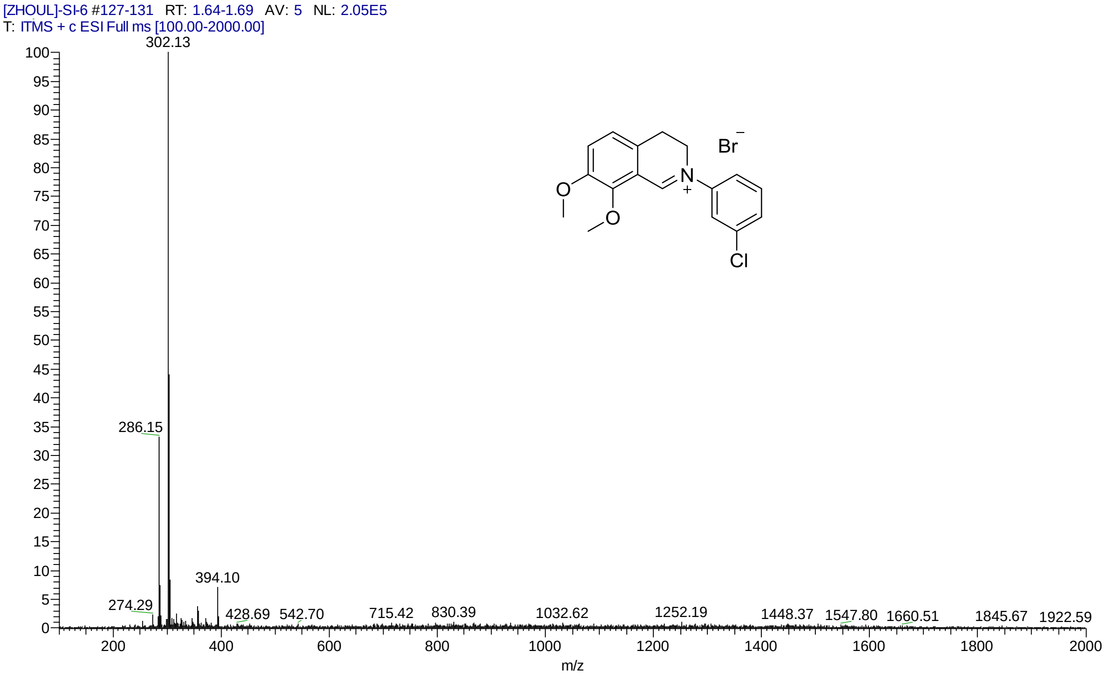

ESI-MS of compound **A6**

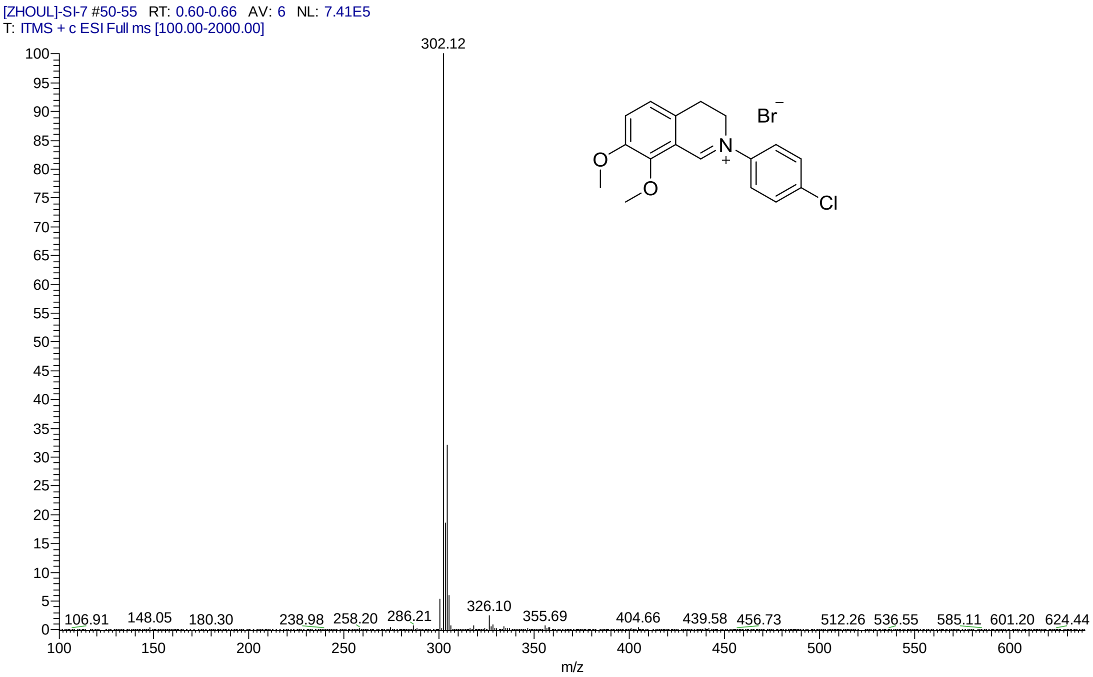

ESI-MS of compound **A7**

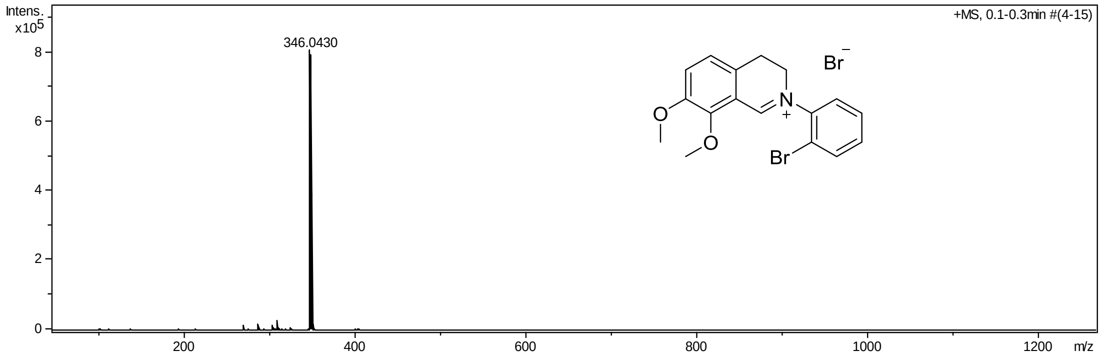

HR-ESI-MS of compound **A8**

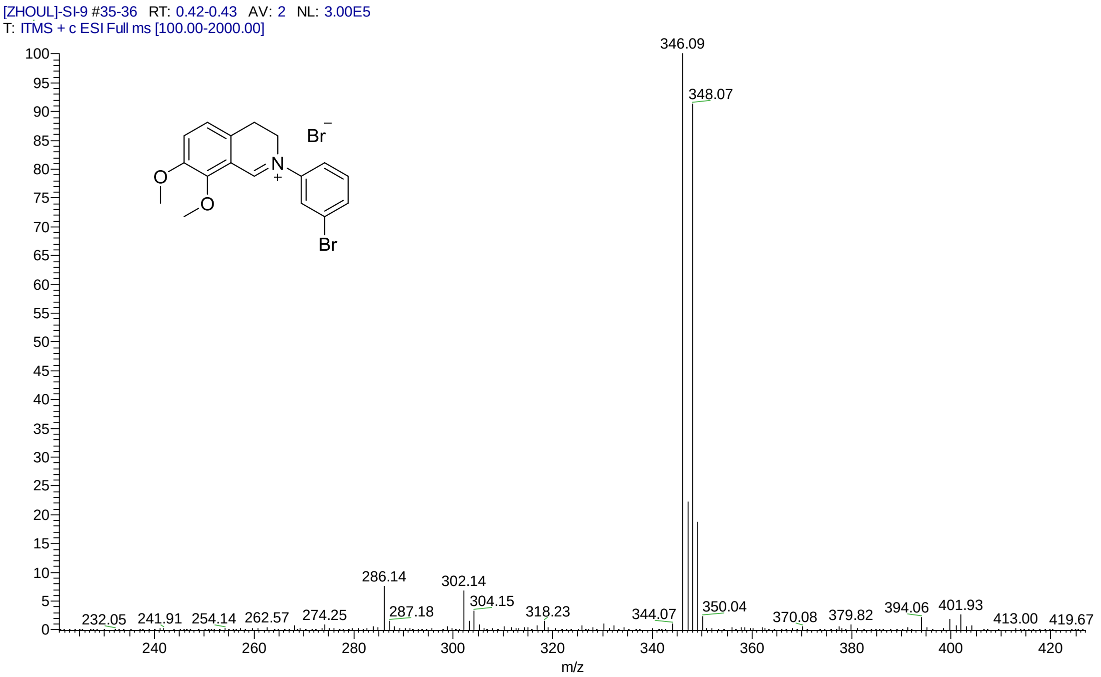

ESI-MS of compound **A9**

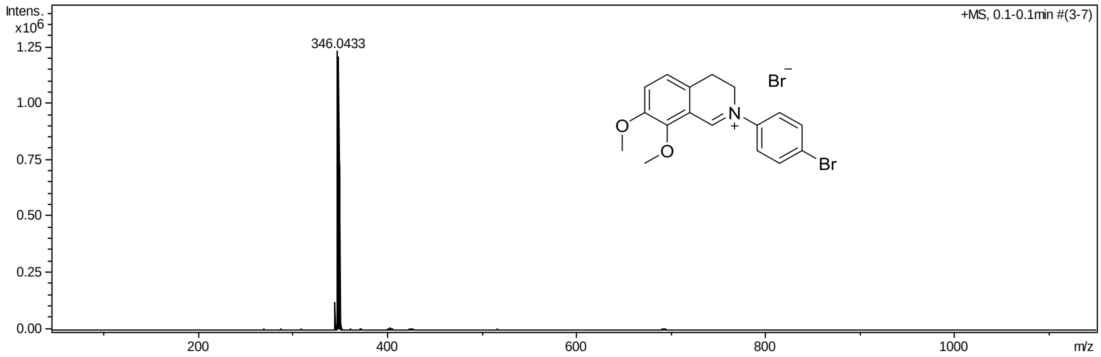

HR-ESI-MS of compound **A10**

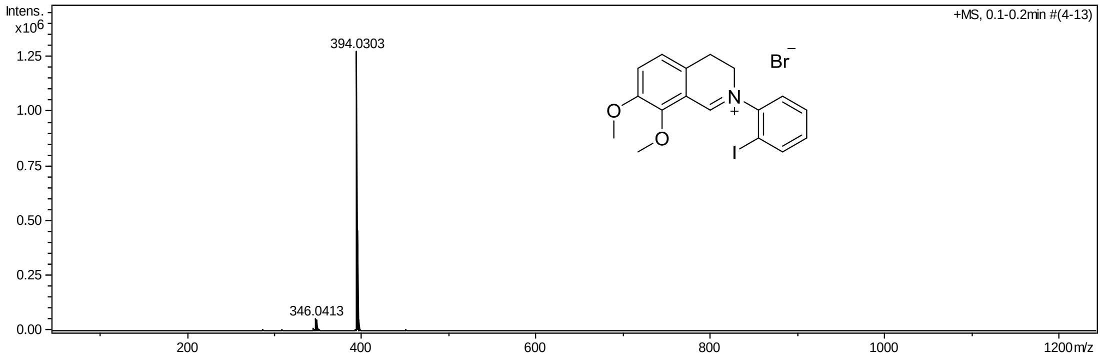

HR-ESI-MS of compound **A11**

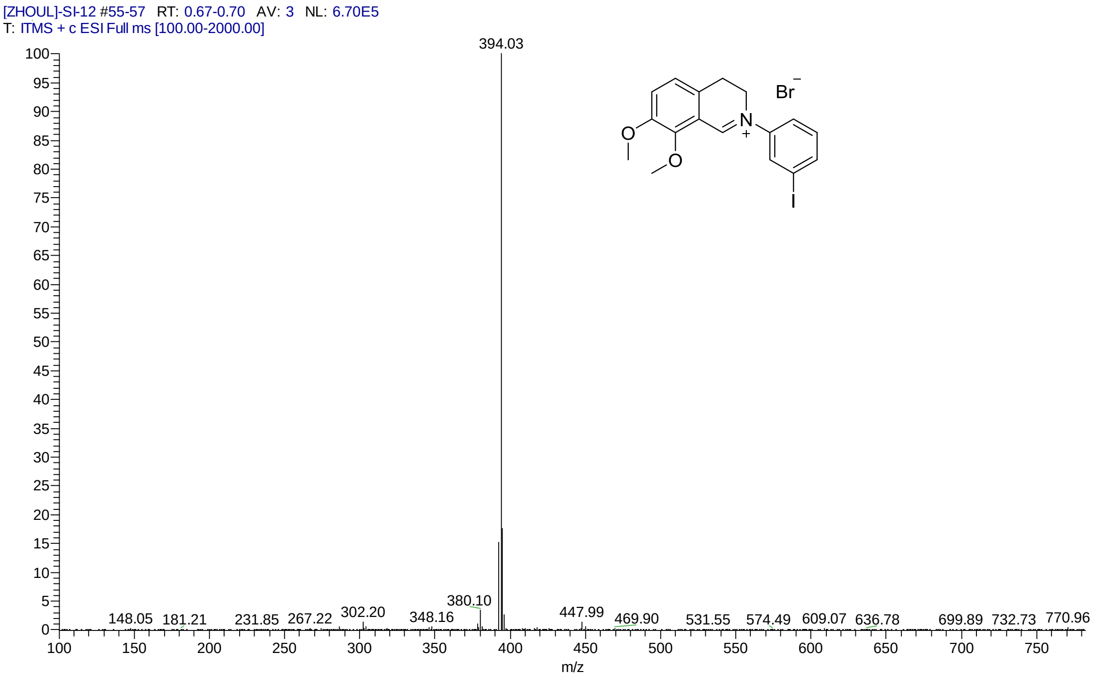

ESI-MS of compound **A12**

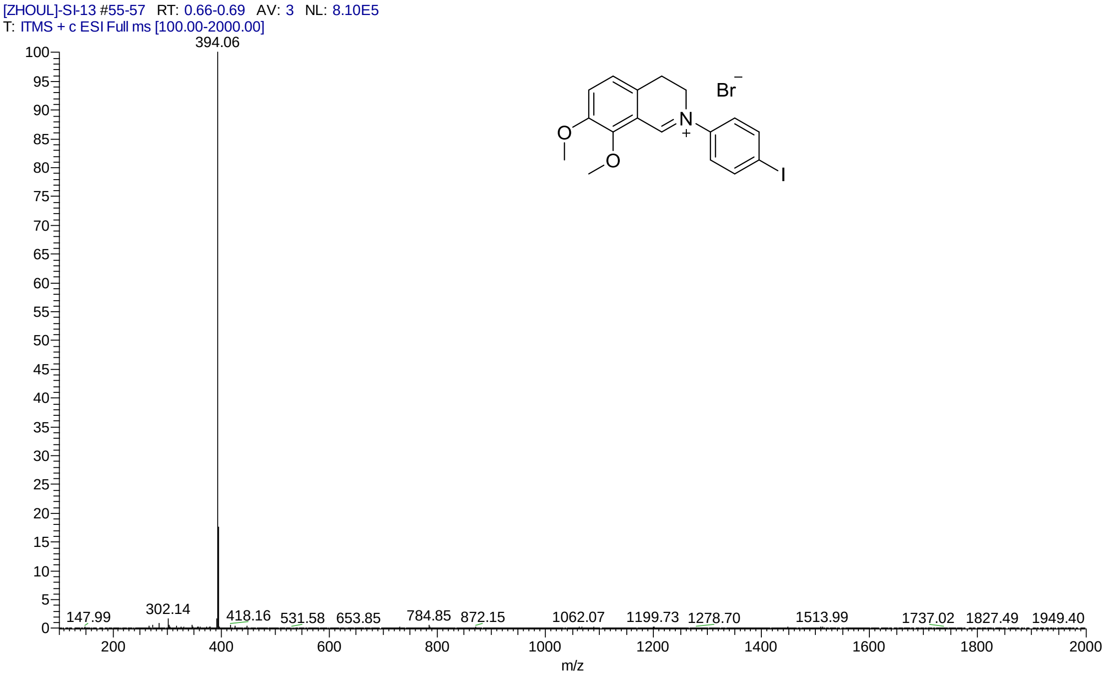

ESI-MS of compound **A13**

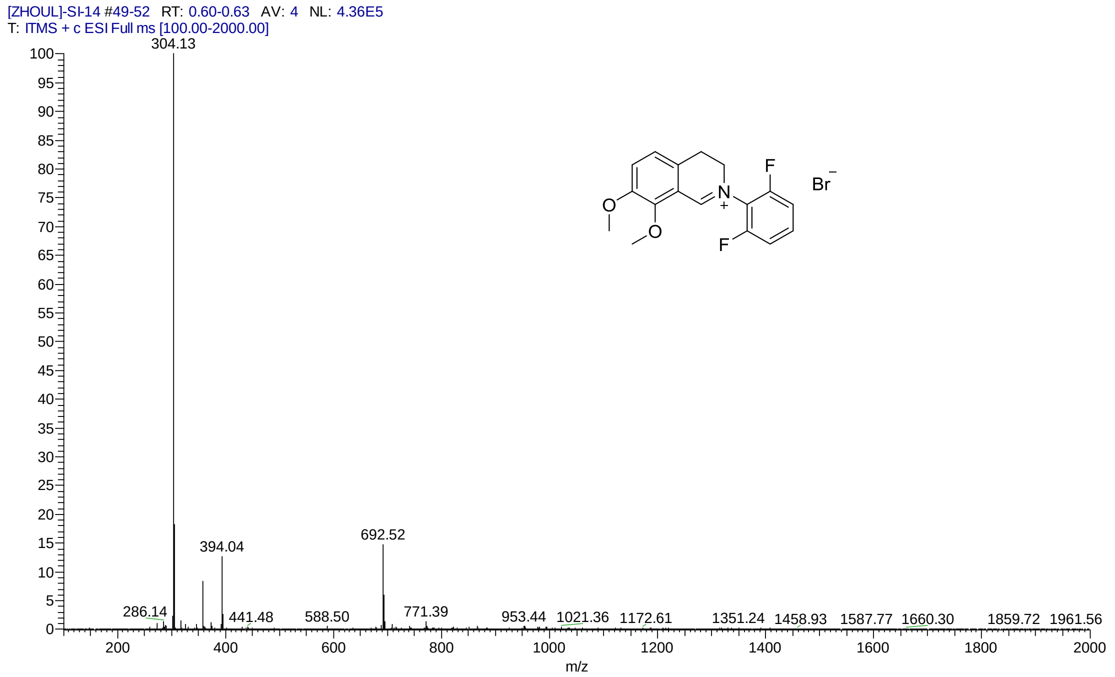

ESI-MS of compound **A14**

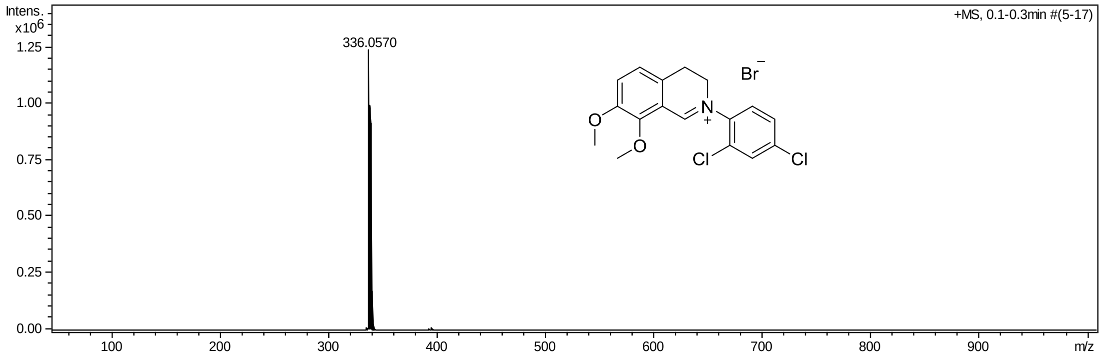

HR-ESI-MS of compound **A15**

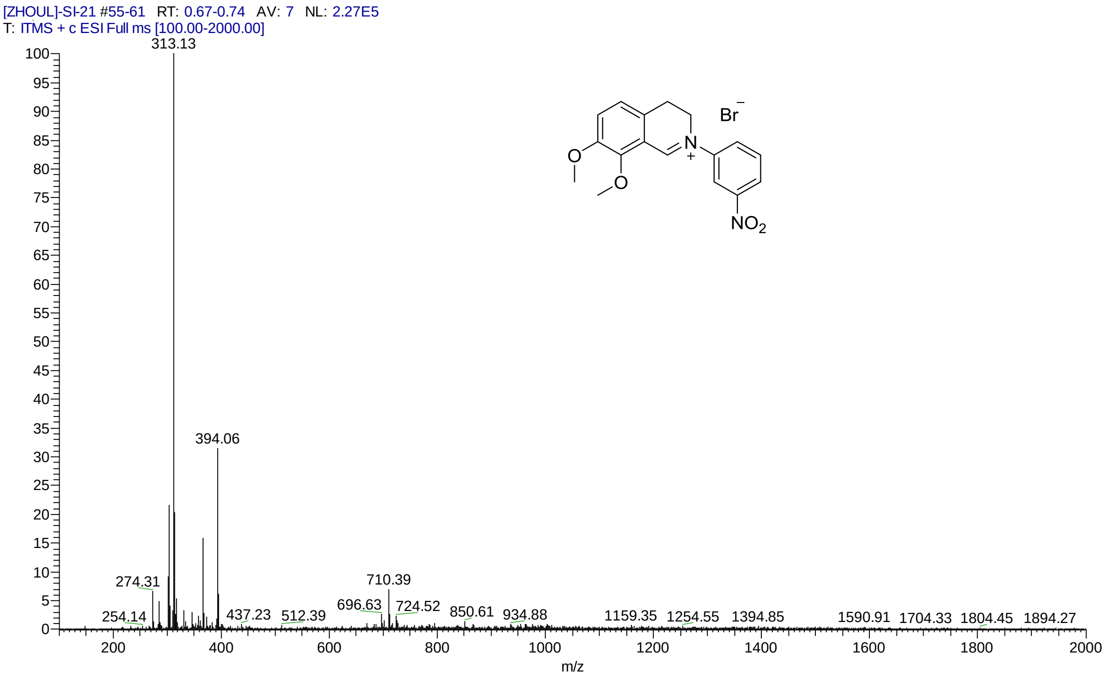

ESI-MS of compound **A16**

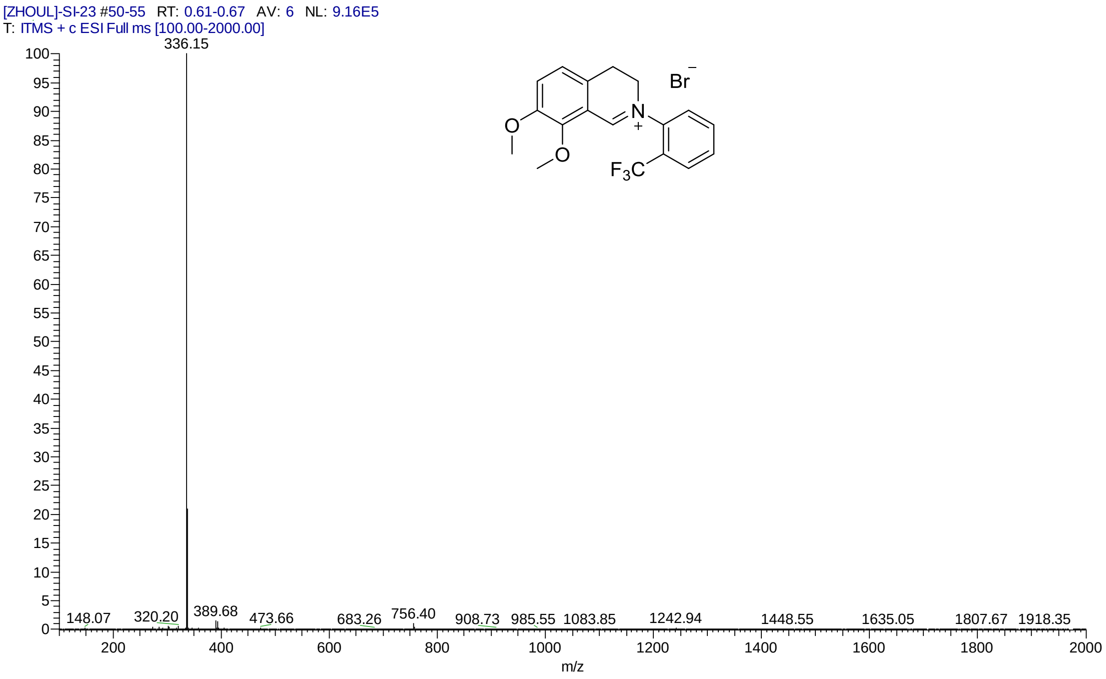

ESI-MS of compound **A17**

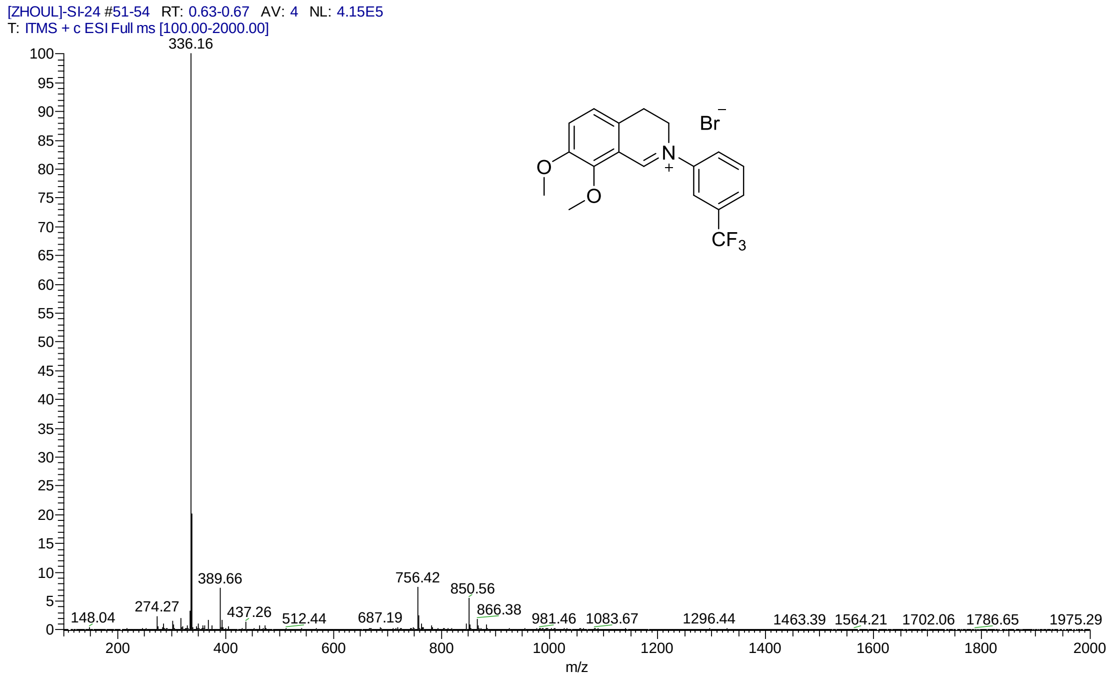

ESI-MS of compound **A18**

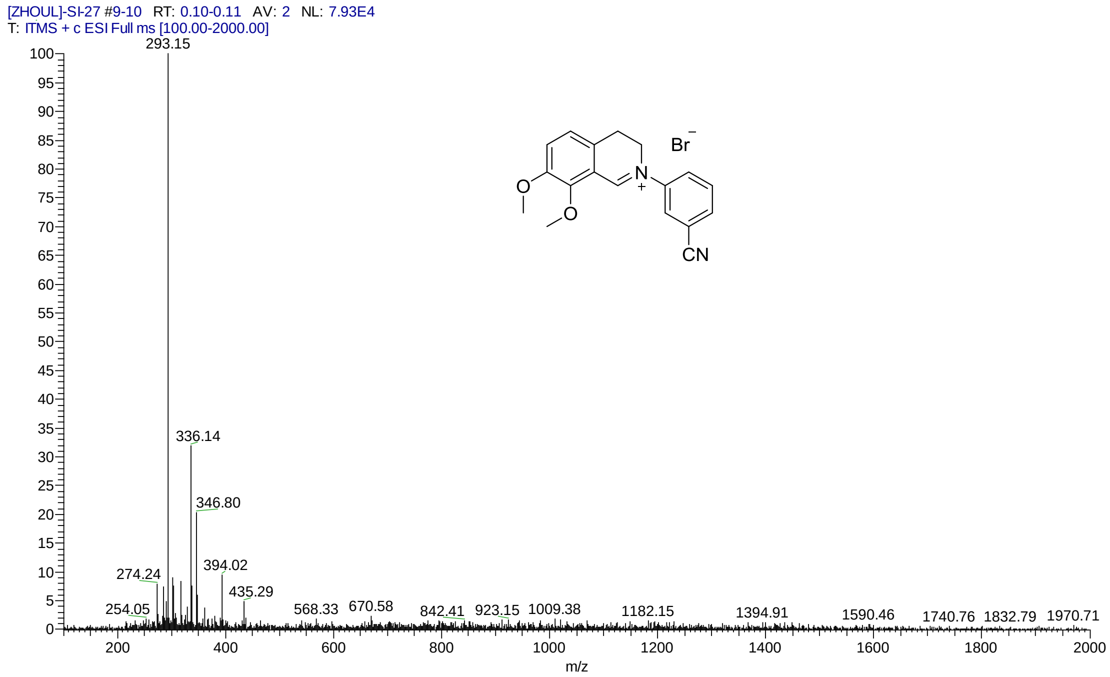

ESI-MS of compound **A19**

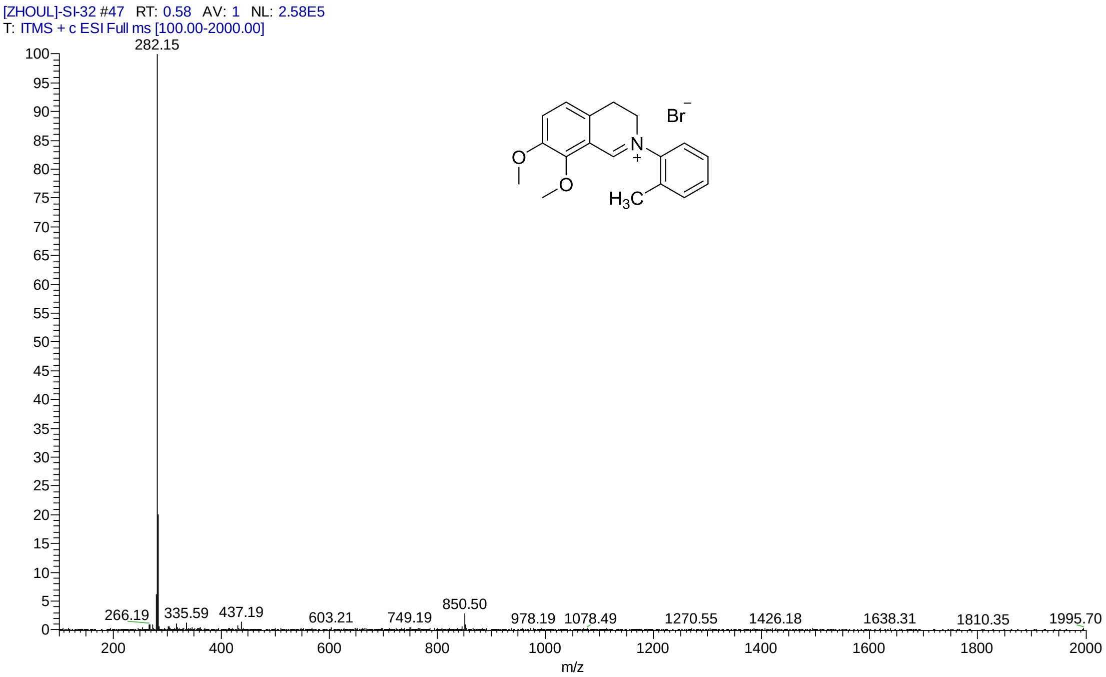

ESI-MS of compound **A20**

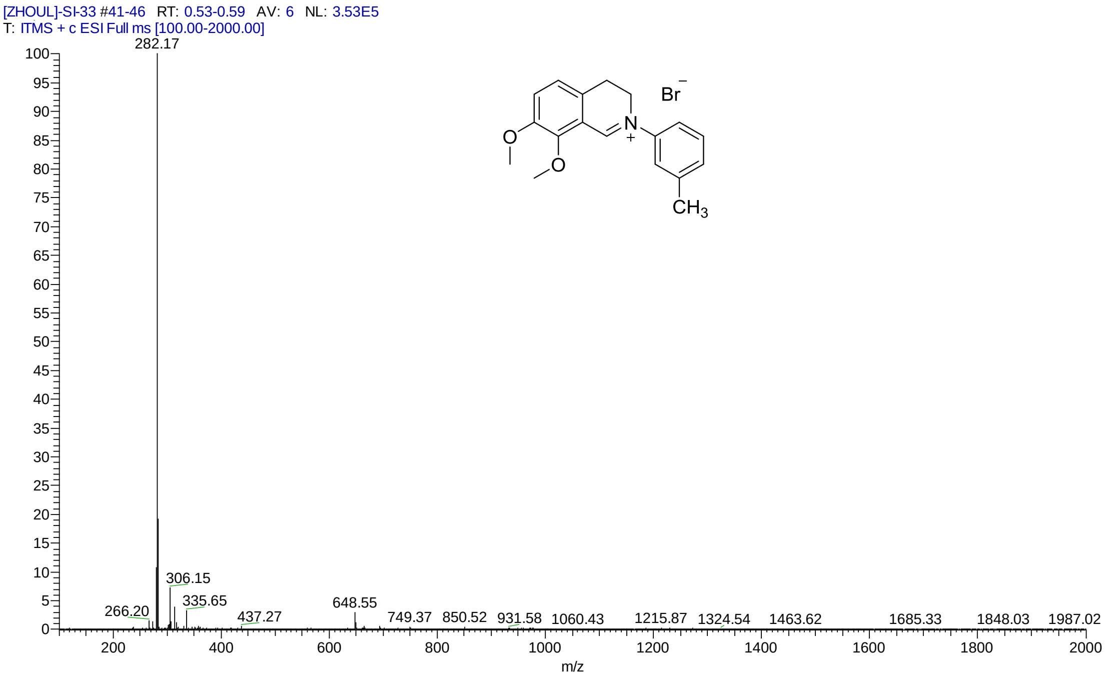

ESI-MS of compound **A21**

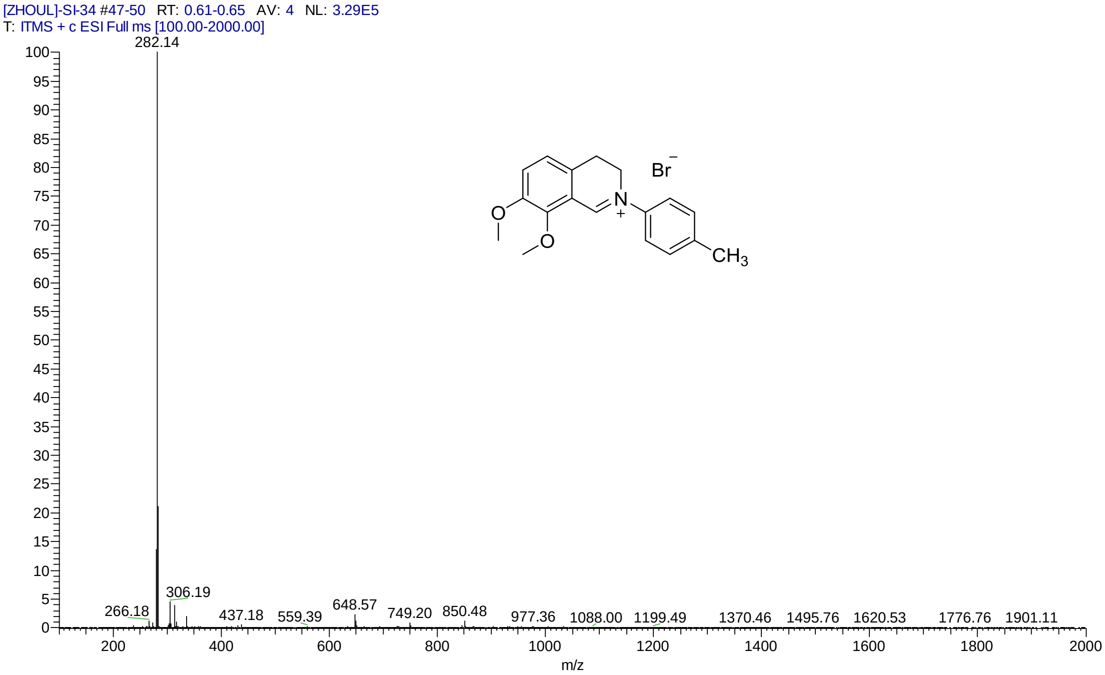

ESI-MS of compound **A22**

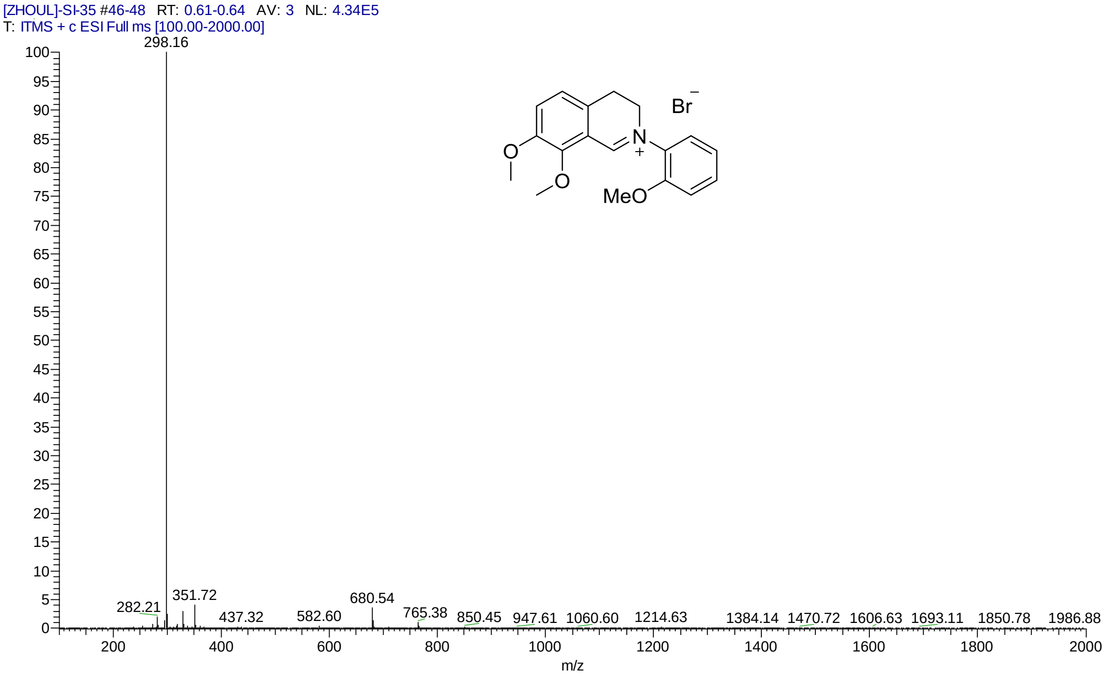

ESI-MS of compound **A23**

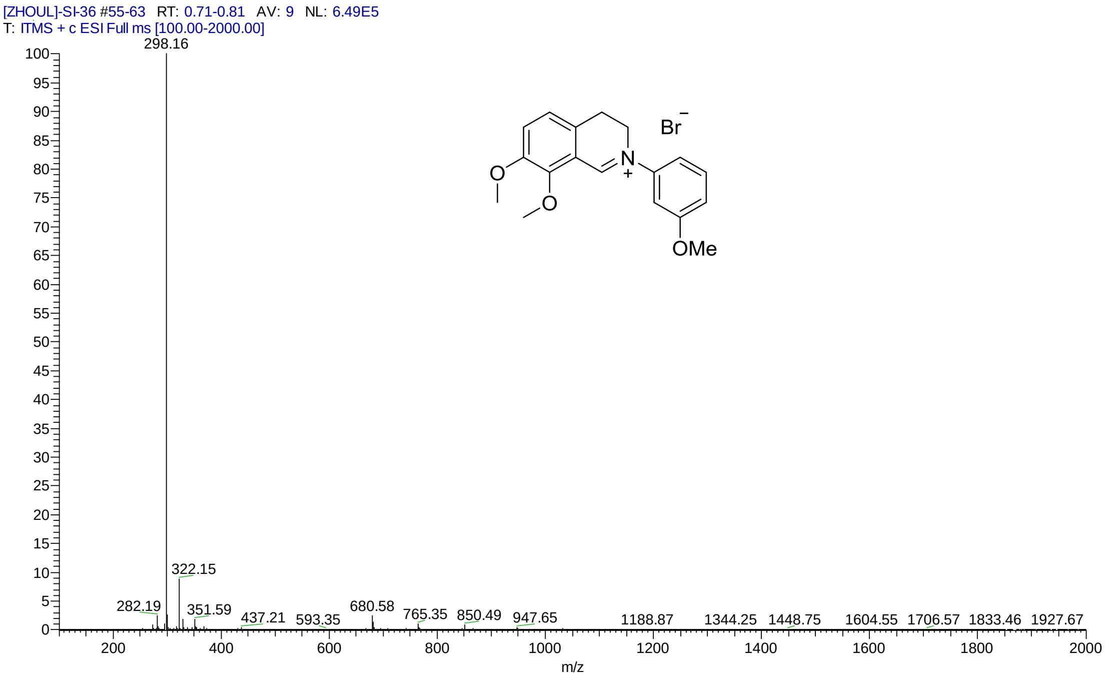

ESI-MS of compound **A24**

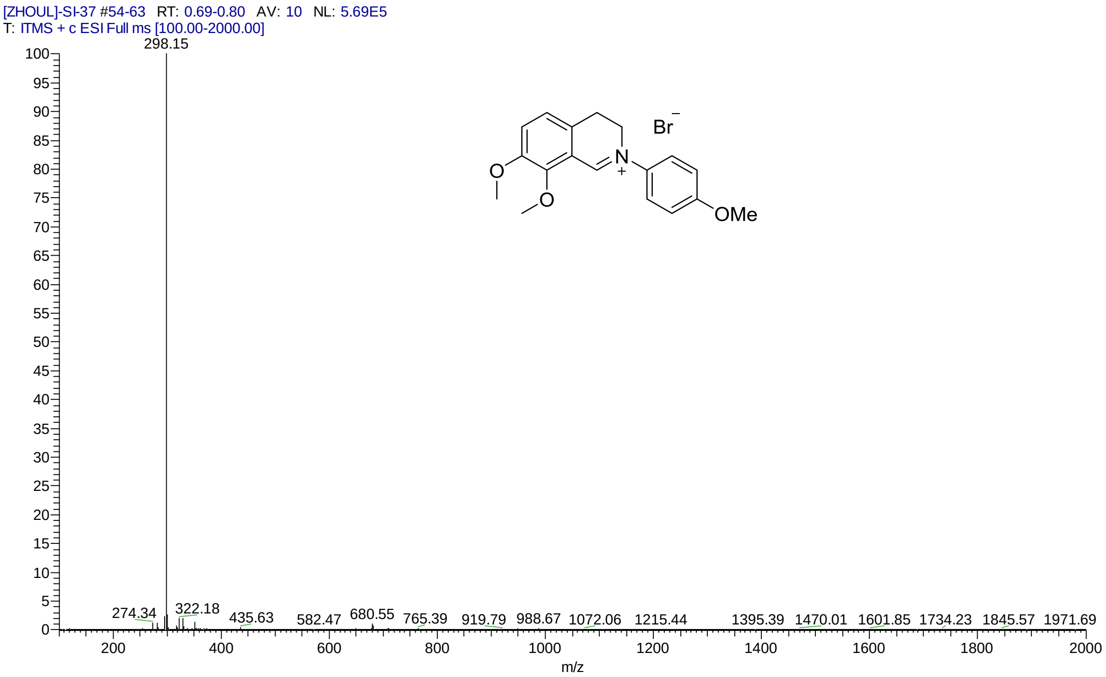

ESI-MS of compound **A25**

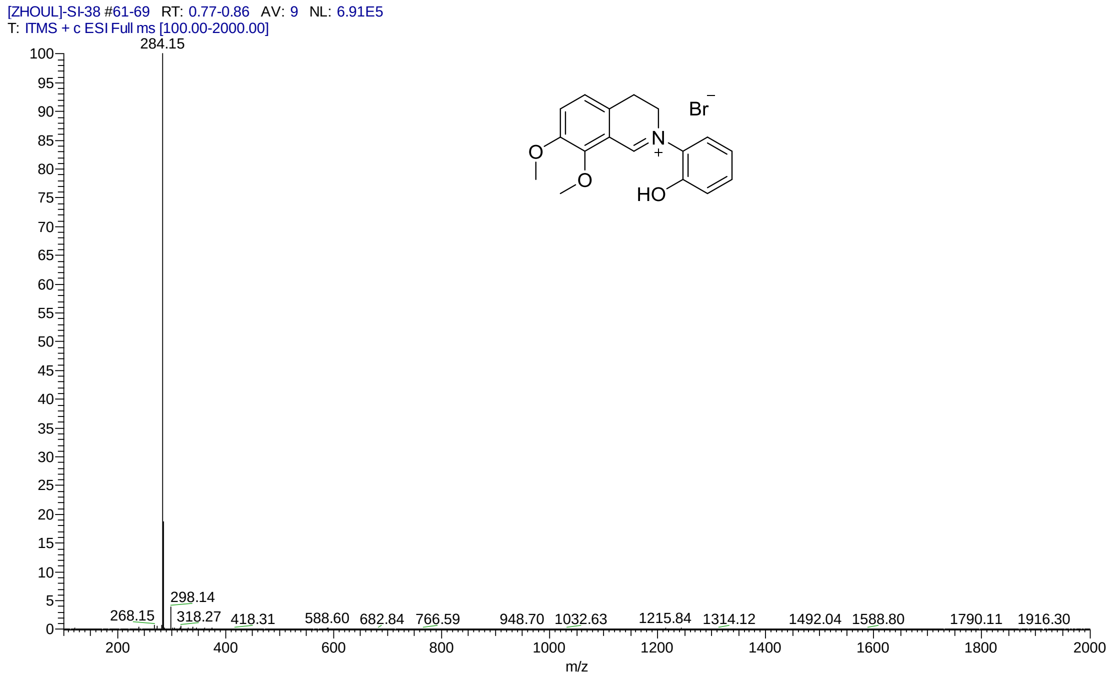

ESI-MS of compound **A26**

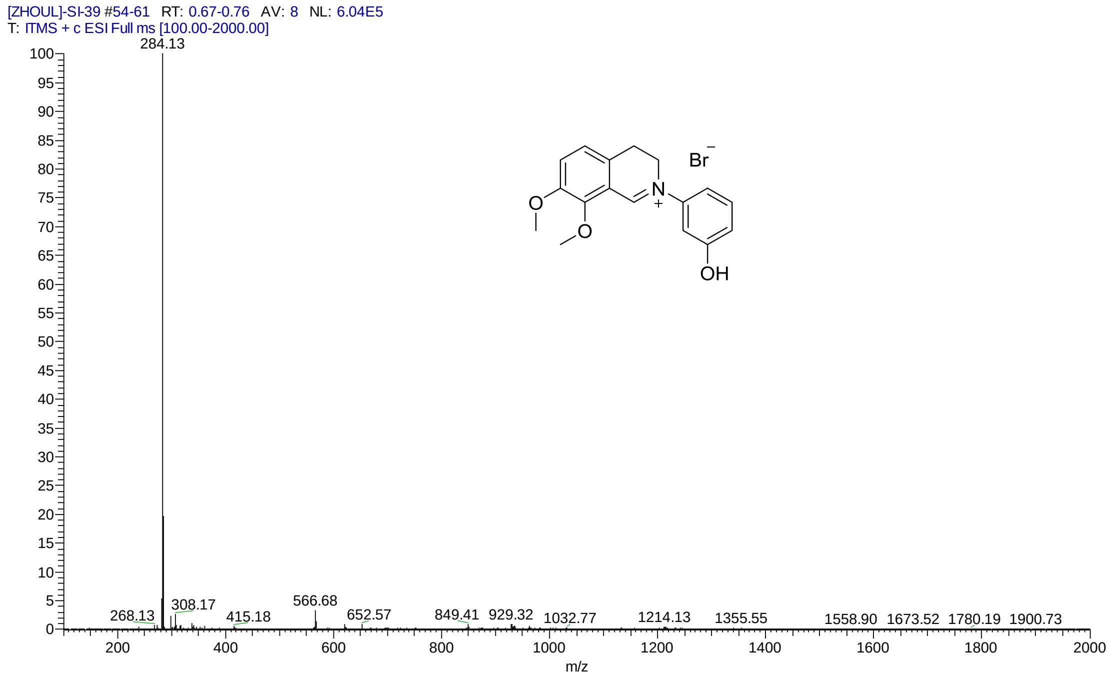

ESI-MS of compound **A27**

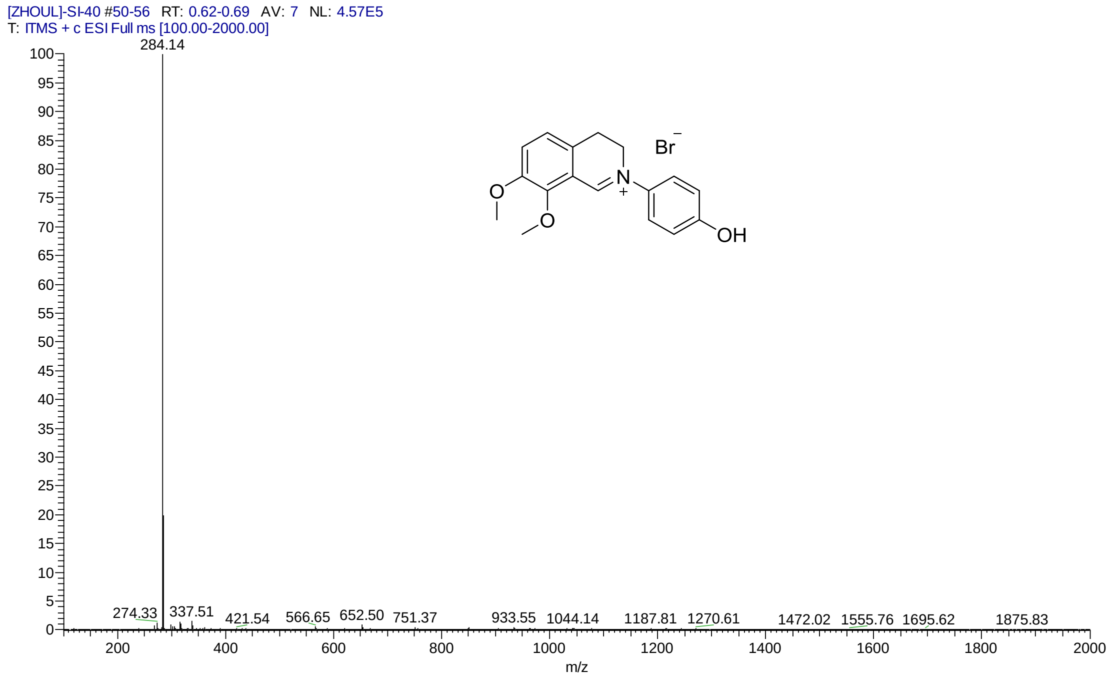

ESI-MS of compound **A28**

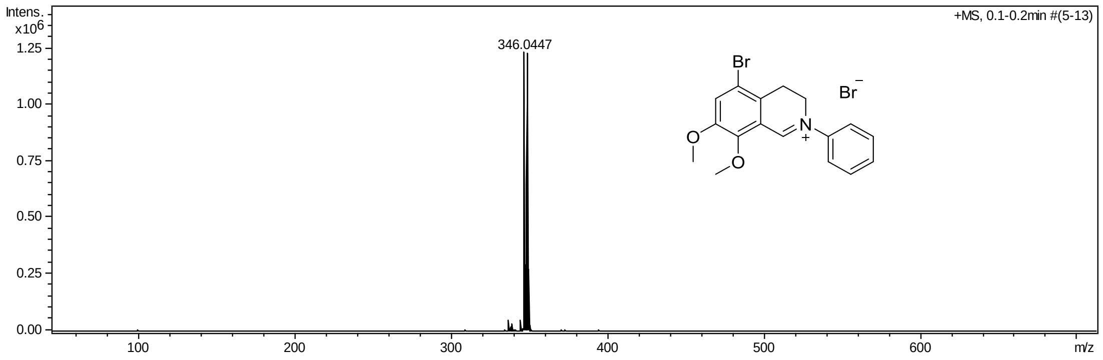

HR-ESI-MS of compound **B1**

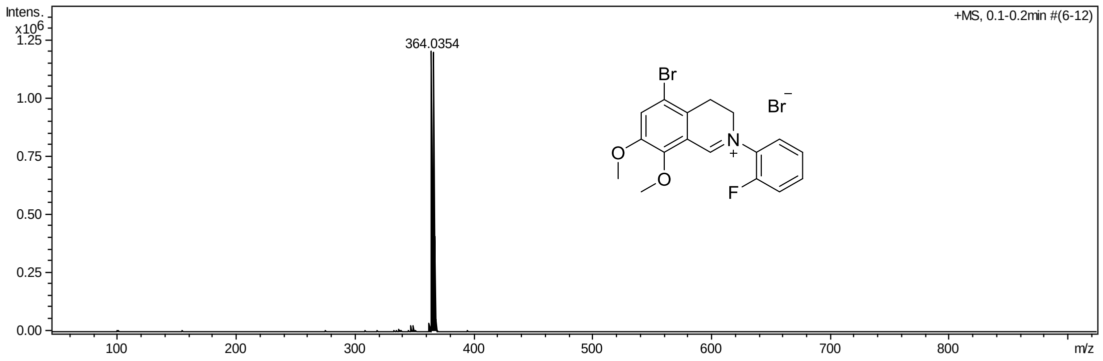

HR-ESI-MS of compound **B2**

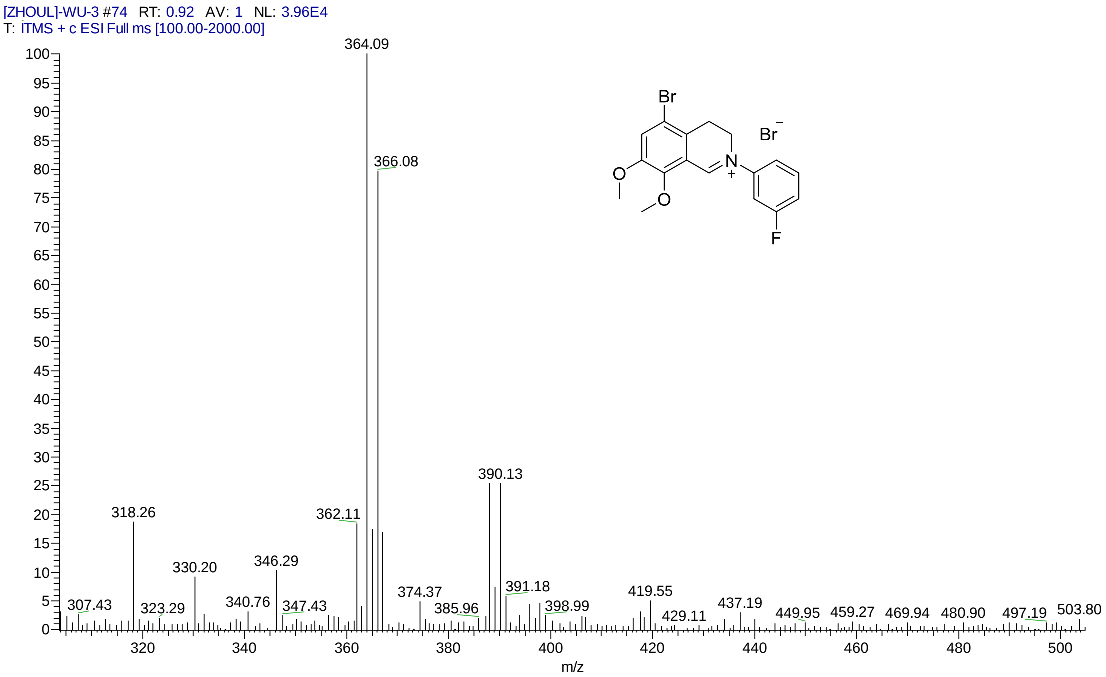

ESI-MS of compound **B3**

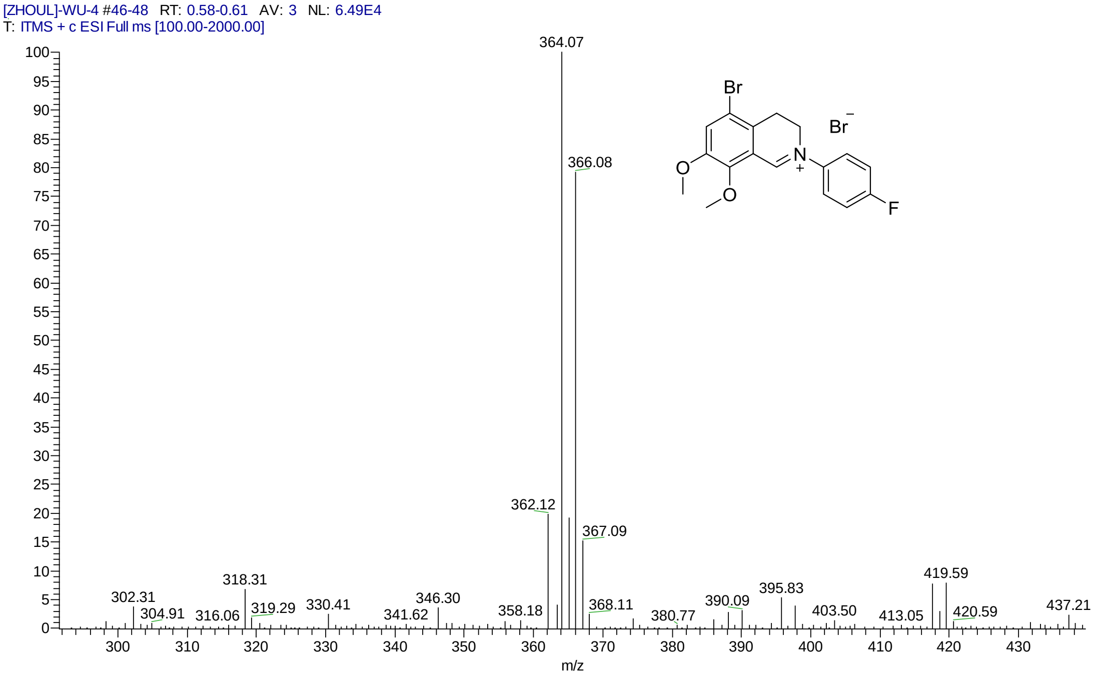

ESI-MS of compound **B4**

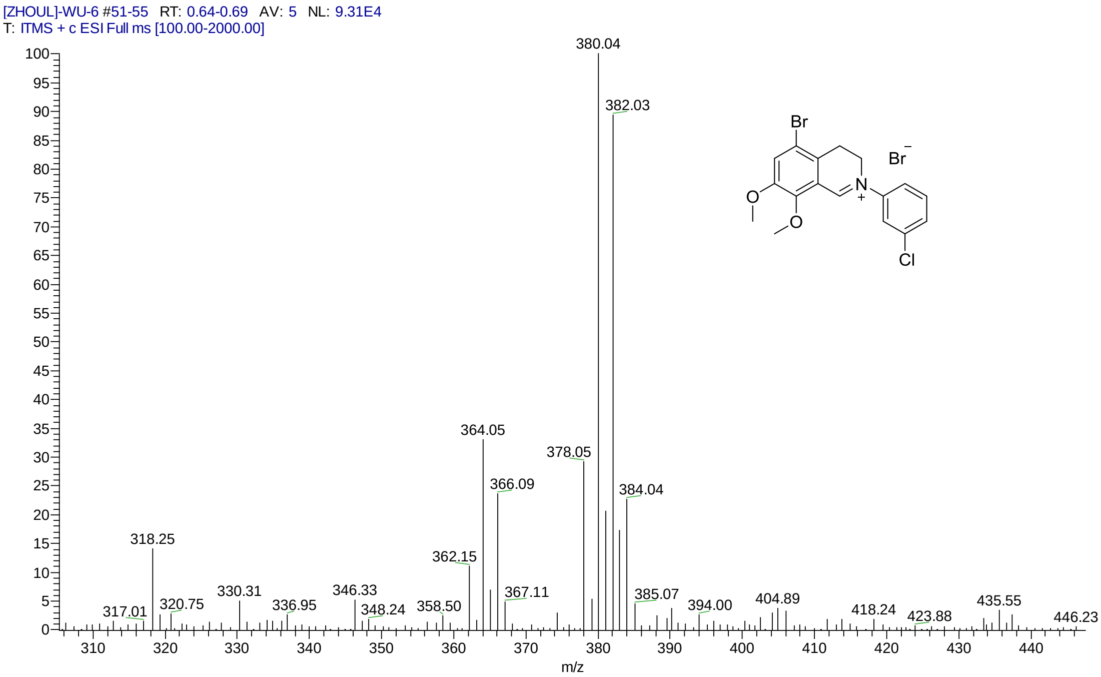

ESI-MS of compound **B5**

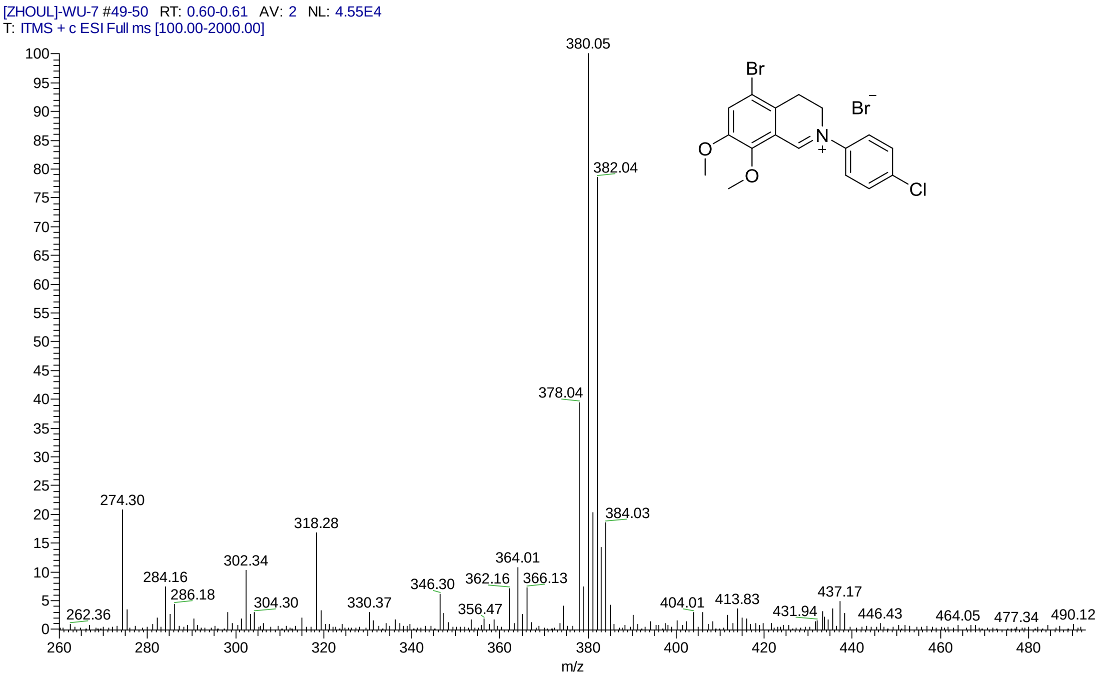

ESI-MS of compound **B6**

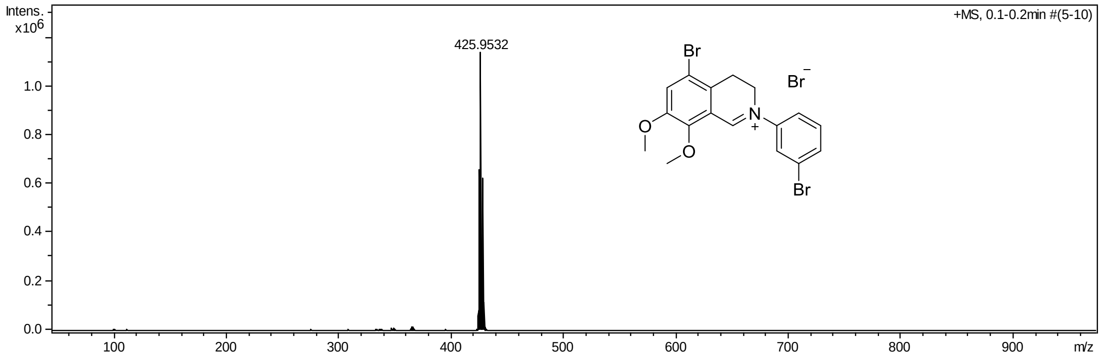

HR-ESI-MS of compound **B7**

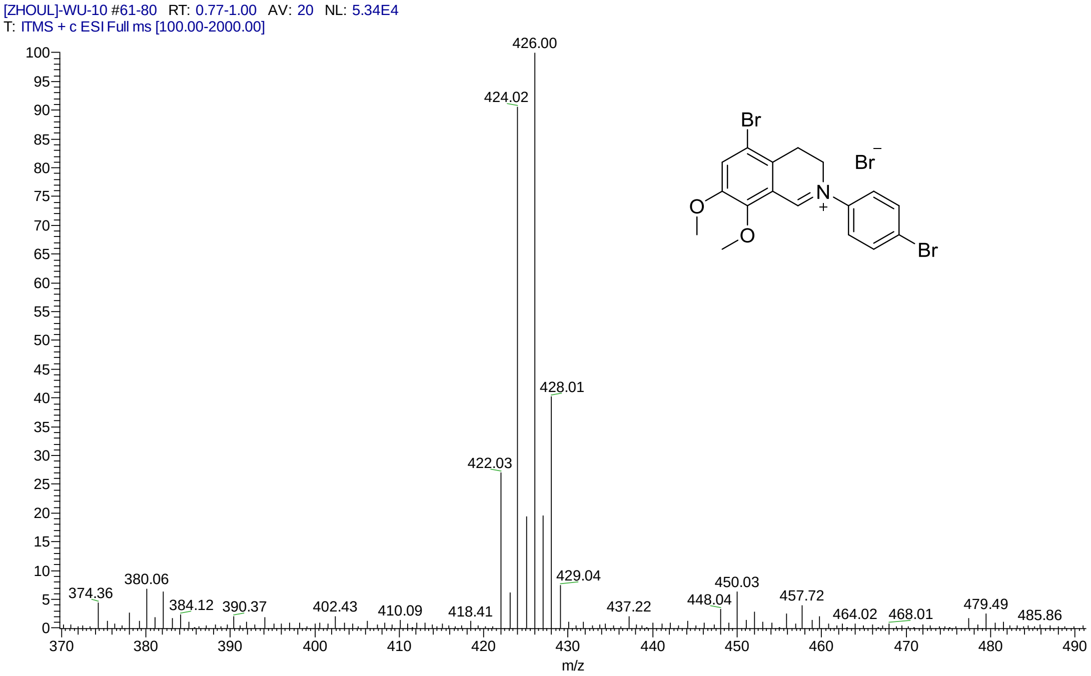

ESI-MS of compound **B8**

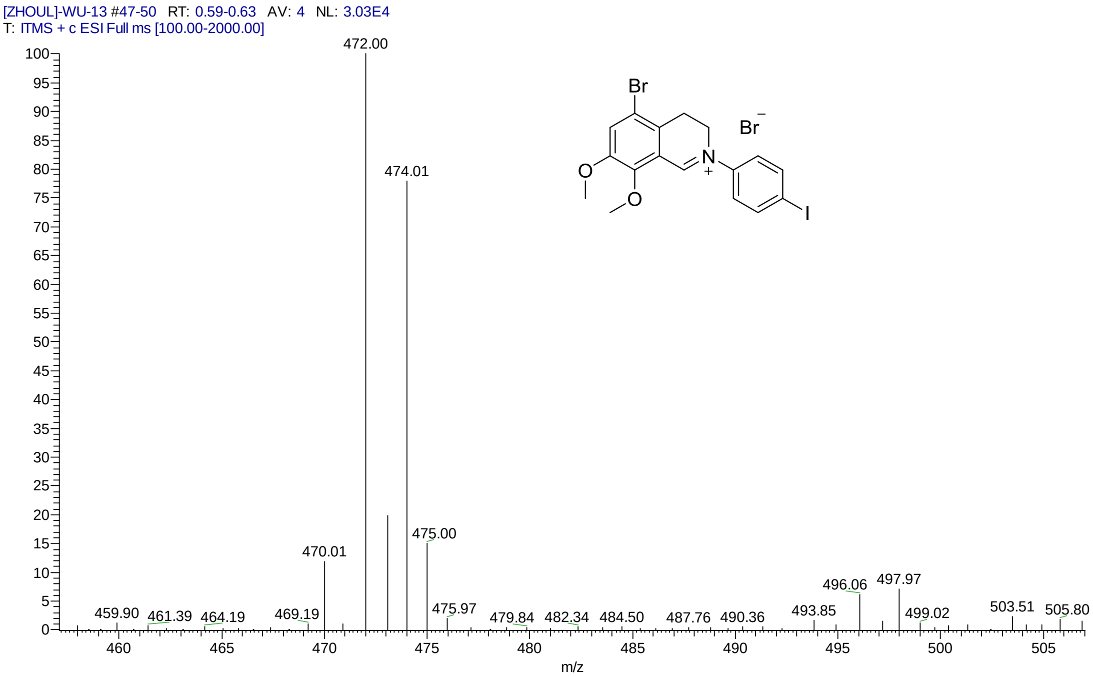

ESI-MS of compound **B9**

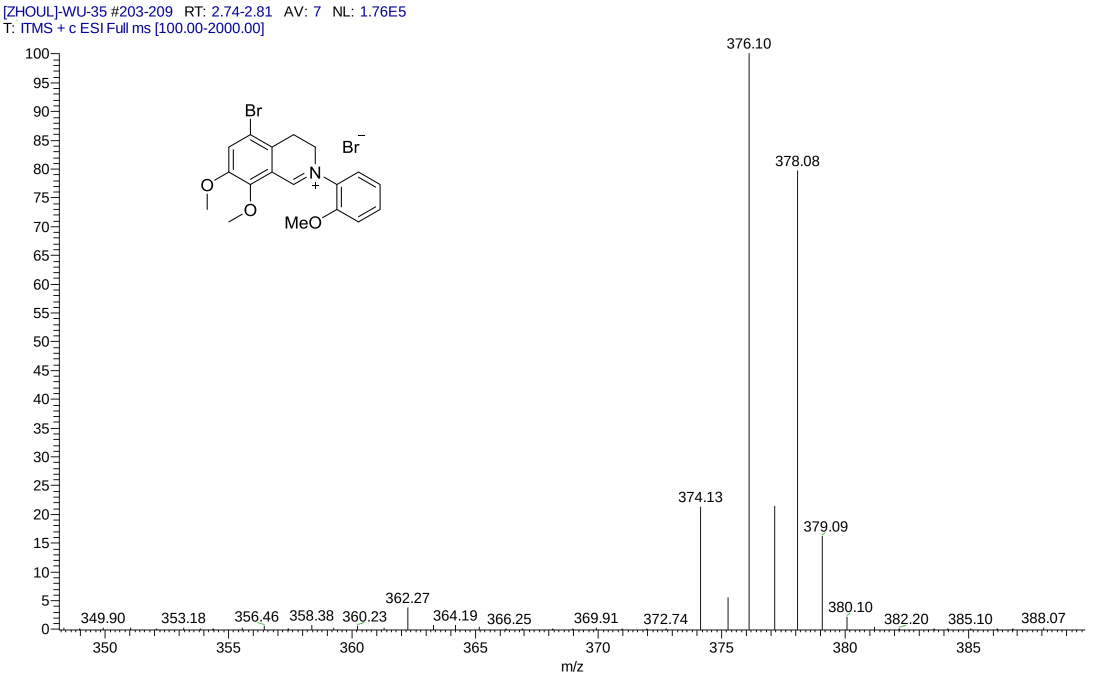

ESI-MS of compound **B10**
